# Total Synthesis of Dimeric HPI Alkaloids

**DOI:** 10.1007/s13659-016-0092-8

**Published:** 2016-03-11

**Authors:** Xianfu Shen, Yongyun Zhou, Yongkai Xi, Jingfeng Zhao, Hongbin Zhang

**Affiliations:** Key Laboratory of Medicinal Chemistry for Natural Resource (Yunnan University), Ministry of Education, School of Chemical Science and Technology, Yunnan University, Kunming, 650091 People’s Republic of China

**Keywords:** Copper catalyzed reaction, Oxidative dimerization, Total synthesis, Hexahydropyrroloindole, Alkaloids

## Abstract

**Abstract:**

In this paper, we report a full account of the synthesis of dimeric hexahydropyrroloindole alkaloids and its analogues. The key feature of our new strategy is the novel catalytic copper (10 %) mediated intramolecular arylations of *o*-haloanilides followed by intermolecular oxidative dimerization of the resulting oxindoles in one pot. This sequential reaction leads to the key intermediates for the synthesis of (+)-chimonanthine, (+)-folicanthine, (−)-calycanthine and (−)-ditryptophenaline.

**Graphical Abstract:**

In the presence of catalytic amount of cuprous iodide (10 %), an intramolecular arylation of *o*-haloanilides followed by an intermolecular oxidative dimerization of the resulting oxindoles leads to a common intermediate for the synthesis of (+)-chimonanthine, (+)-folicanthine and (−)-calycanthine. Based on this cascade sequence, we also developed a flexible strategy towards the asymmetric syntheses of dimeric HPI alkaloids (−)-ditryptophenaline and its analogues.
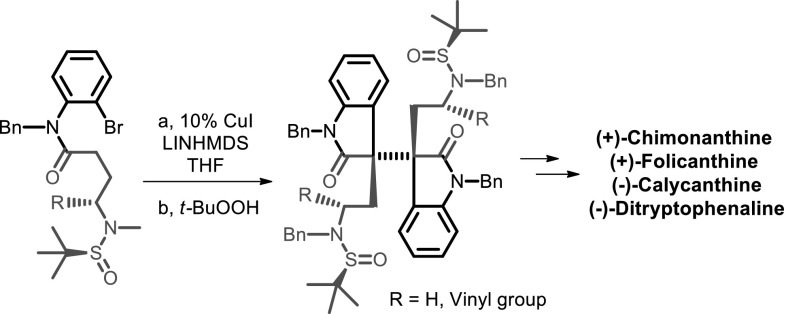

**Electronic supplementary material:**

The online version of this article (doi:10.1007/s13659-016-0092-8) contains supplementary material, which is available to authorized users.

## Introduction

**Fig. 1:**
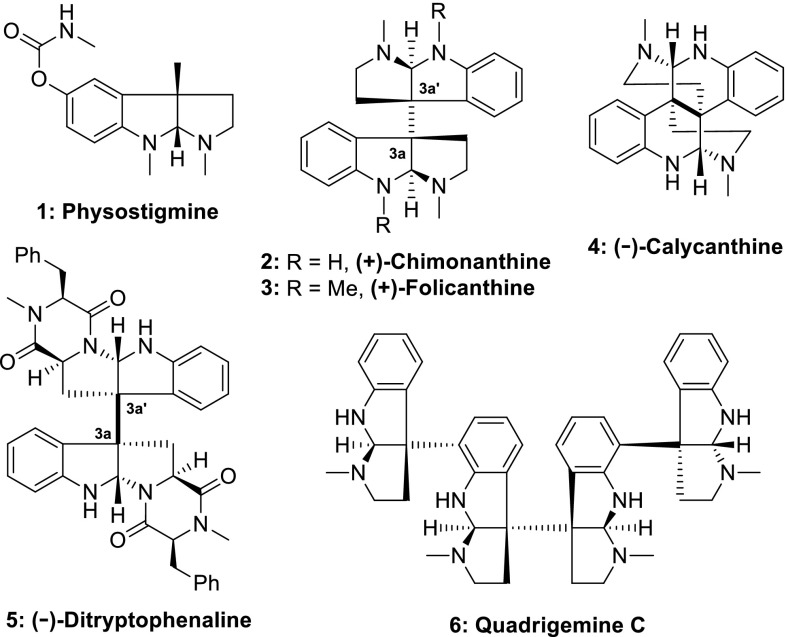
Natural HPI alkaloids

The hexahydropyrroloindole (HPI) structure-unit presents in a large class of natural products isolated from plants, microorganisms and fungi (for selected reviews and book chapters, see: [1–8]). Representative natural product of this family is physostigmine (Fig. 1, **1**) which was isolated from the seeds of the calabar bean plant and is currently used to treat myasthenia gravis, glaucoma, Alzheimers disease, delayed gastric emptying and orthostatic hypotension [5, 7]. There are a number of alkaloids containing more than one HPI unit and some of them contain a unique vicinal C3a–${\text{C}}{{3{\text{a}}}}^{'}$ quaternary carbon center [1, 4]. The stereocontrol synthesis of the congested all-carbon quaternary stereocenters in these alkaloids presents a formidable challenge [9–16]. In 1999, Overman and his team completed the first enantioselective synthesis of dimeric alkaloid (+)-calycanthine (Fig. 1, **4**) and (−)-chimonanthine (Fig. 1, **2**) [17]. Utilization of the same strategy, they also successfully synthesized a number of other dimeric and oligomeric HPI alkaloids [17–27]. In 2007, Mavassaghi reported a reductive radical dimerization strategy for the syntheses of dimeric HPI alkaloids (Fig. 1) [28, 29]. Based on this reductive dimerization reaction, a number of important works have been published towards the syntheses of dimeric HPI alkaloids (for selected syntheses of dimeric HPI alkaloids employed Movassaghi’s reductive dimerization, see: [30–37]). Prompted by the success of reductive dimerization strategies, studies on the oxidative dimerization of tryptamine and tryptophan derivatives have been revived and syntheses of natural HPI alkaloids, especially chimonanthine, folicanthine and ditryptophenaline, have been achieved by a number of research groups (oxidative dimerization of tryptamine and tryptophan derivatives as the key steps for the syntheses of dimeric HPI alkaloids before 2007: [38–58]).

Careful examination of literature related to the total syntheses of dimeric HPI alkaloids, we found that tryptamine or tryptophan derivatives were used frequently as starting materials [38–58], few examples had been documented by application of non-indole and/or non-oxindole starting materials [59]. To some extent, utilization of tryptamine and tryptophan derivatives as starting materials might limited the access of structurally diverse dimeric analogues. Therefore, it is of importance to develop alternative approaches towards the synthesis of the target dimeric HPI natural molecules as well as its analogues for the interests of medicinal chemistry.

**Scheme 1:**
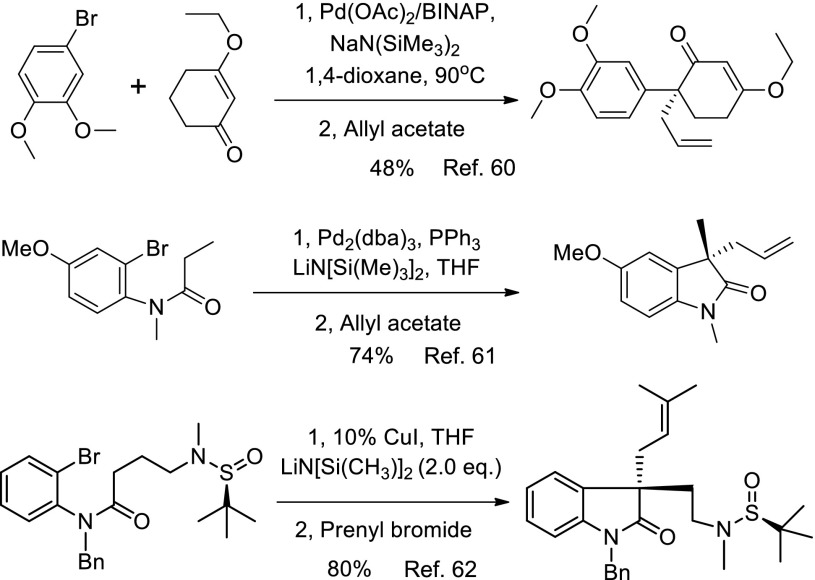
Sequential process for natural product synthesis

A major focus of our research group is the use of metal mediated sequential reactions to assemble the key structure units of the target molecules [60–63]. Our synthesis of the HPI alkaloids initiated in the early 2008. We successfully developed a sequential reaction for the synthesis of mesembrine [60] and esermethole [61] using palladium chemistry. In 2012, we developed a copper catalyzed arylation of *o*-bromoanilides assisted by a remote sulfinylamide or carbamate auxiliary [62]. Very recently, we established a novel copper catalyzed asymmetric arylation-oxidative dimerization of *o*-haloanilide derivatives (Scheme 1) to construct the vicinal C3a–${\text{C}}{{3{\text{a}}}}^{'}$ all carbon quaternary stereocenters required for the synthesis of dimeric HPI compounds [64]. In this paper, we report our full accounts of new strategy towards the synthesis of (+)-chimonanthine, (+)-folicanthine, (−)-calycanthine, (−)-ditryptophenaline and its analogues.

## Results and Discussion

The syntheses of dimeric HPI alkaloids began in the early 1960, and after endeavours of many research groups, the bio-inspired oxidative dimerization reaction of tryptamine and tryptophan derivatives has been developed to be a powerful method for the construction of dimeric HPI alkaloids [38–58, 64]. In comparison with oxidative dimerization of tryptamine and tryptophan derivatives, however, few oxidative dimerizations of oxindole derivatives have been reported [65–74]. To the best of our knowledge, only five papers reported direct oxidative dimerization of oxindoles derivatives. Except for the method established by Rodrigo which provided the dimeric intermediate in 61 % overall yield and good diastereoselectivity (*dl*:*meso* isomers = 53:8, Scheme 2) [67], other methods unfortunately suffered from low yields [65] or poor diastereoselectivity [66, 68, 69]. Before we conducted this research, no asymmetric oxidative dimerization of oxindoles had been developed to form the vicinal C3–${\text{C}}{3}^{'}$ all-carbon quaternary centers.

**Scheme 2:**
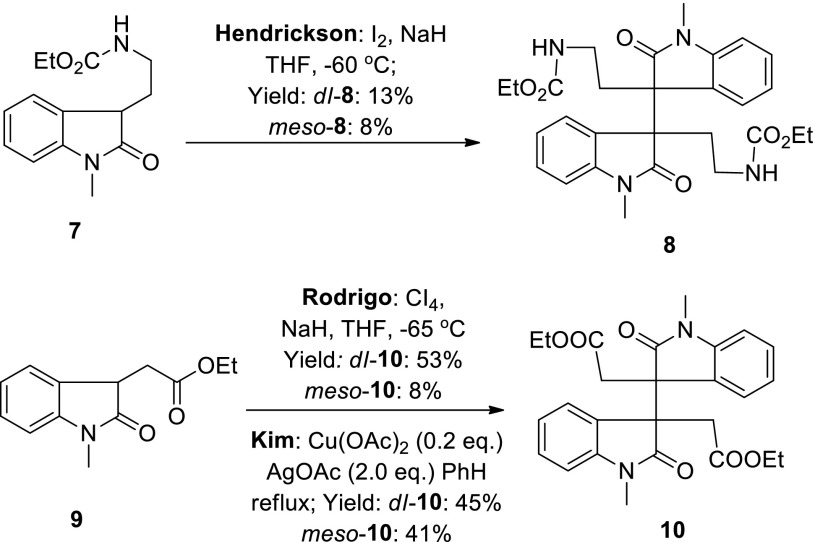
Oxidative dimerization of oxindoles in the literature

**Scheme 3:**
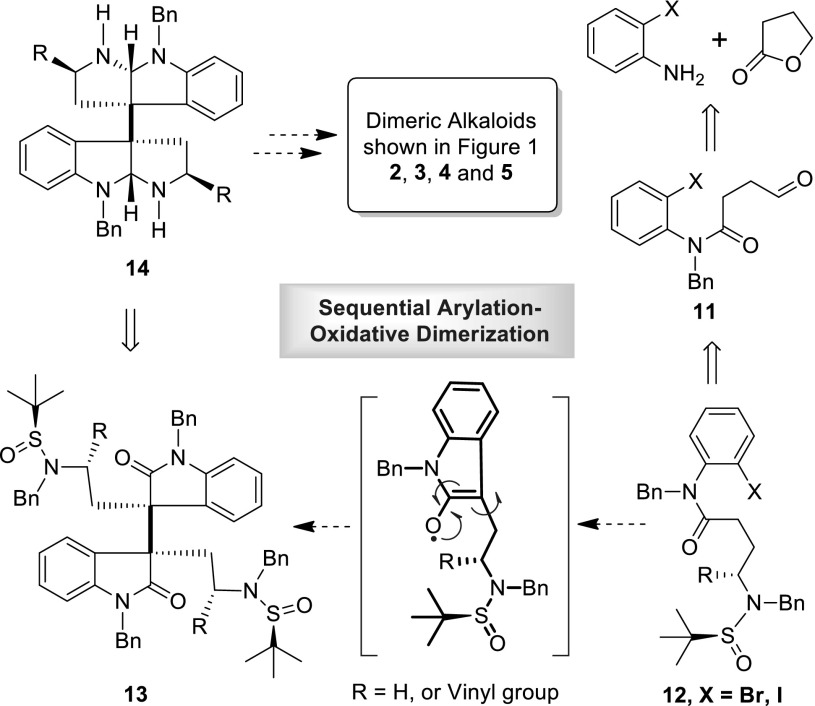
Synthetic analysis of dimeric HPI alkaloids

Recently, we disclosed an asymmetric synthesis of debromoflustramine and its analogues through a copper mediated arylation followed by oxindole-alkylation [62]. We envisioned that this copper catalysed cyclization process (Scheme 1) might be applied to the synthesis of dimeric HPI alkaloids indicated in Fig. 1. A retrosynthetic analysis is outlined in Scheme 3. Key to our new strategy is to develop a sequential reaction that combines metal catalysed arylation with an in situ oxidative dimerization of the resulting oxindole intermediates (Scheme 3, converting **12** to **13**). Based on this new sequential process, we would be able to synthesize the dimeric HPI alkaloids starting from *o*-haloanilide **12**. The amides (**12**) bearing a chiral sulfinyl amide unit [(*S*) or (*R*)-*tert*-butanesulfinamide] could be synthesized according to our previous procedure. We were curious to know whether the key intermediates (**13**, Scheme 3), containing the vicinal C3–${\text{C}}{3}^{'}$ all-carbon quaternary centers required for dimeric HPI alkaloids, could be formed diastereoselectively in a one-pot manner by a copper catalyzed arylation of *o*-haloanilide (**12**) followed by an oxidative dimerization of the newly generated oxindole intermediates in the presence of a suitable oxidant.

**Scheme 4:**
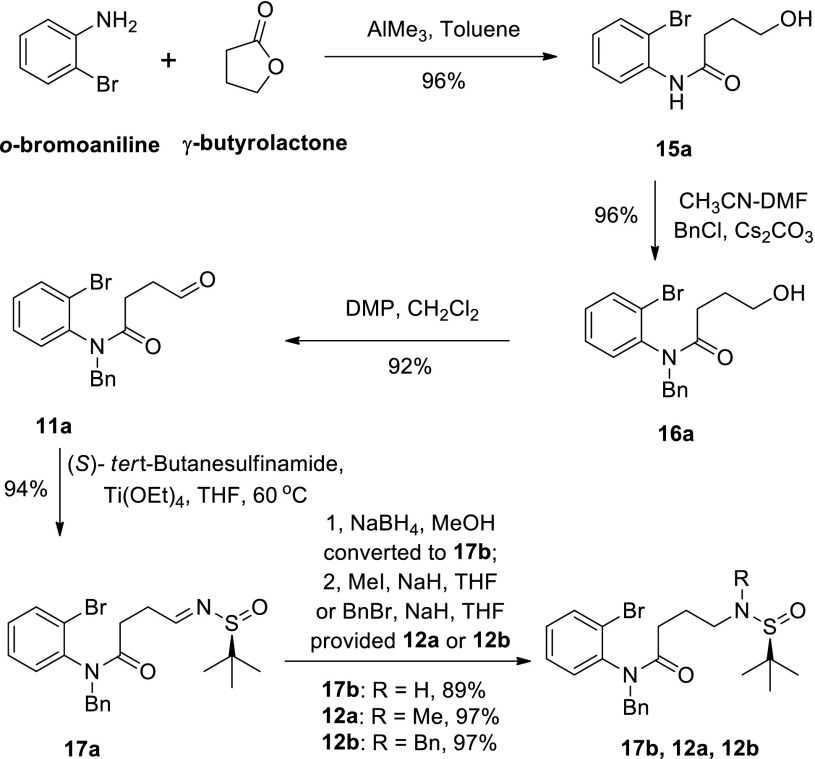
Syntheses of amides **12a** and **12b**

Our synthesis started from commercially available *o*-bromoaniline and γ-butyrolactone, and (*S*)-(−)-*tert*-butanesulfin-amide was used to introduce the nitrogen atom and also served as a chiral auxiliary. Amide **12a** was synthesized in 68 % overall yield in six steps according to our previous procedure [62] (Scheme 4). Amide **12b** was also prepared from **17a** in a 97 % yield. With amides (**12a** and **12b**) in hand, we next began to explore the key copper mediated sequential arylation-dimerization of *o*-bromoanilides, in the hope that the oxidative dimerization might also be effected by copper salts in an efficient and economic “one pot” operation.

The initial experiment was conducted with amide **12b** under our previous optimized reaction condition [62], namely LiN(SiMe_3_)_2_ and CuI in THF at reflux (Table 1, entry 1). This condition unfortunately gave a complex mixture, with the isolated product being *N*-benzyl-*o*-bromoaniline (**18**, 16 % yield). After several unsuccessful experiments, we finally found that the desired sequential reaction could be realized smoothly in toluene (Table 1, entry 6). Arylation of sulfinamide **12b** with catalytic amount of cuprous iodide (10 % eq.) and lithium bis(trimethylsilyl)amide (2.0 eq.) in toluene, followed by oxidation with anhydrous *tert*-butylhydroperoxide resulted in dimeric diastereomers (**13a** + **13b**, as a 5:1 mixture of diastereoisomers in 78 % yield, and **13c**: the *meso* isomer in 7 % yield). We also isolated small amount of the C3-hydroxy-oxindole (**19**). Although a number of oxidants could be used to generate the desired dimeric product (**13a** + **13b**), anhydrous *tert*-butyl hydroperoxide proved to be the best additive for this sequential arylation–oxidative dimerization. Next we carried out the sequential procedure with amide **12a** under the optimized reaction condition. The dimeric diastereomers (**13d** + **13e**, as a mixture of diastereoisomers) were obtained in 71 % yield, we also isolated the *meso* isomer **13f** in 8 % yield (Table 1, entry 10).

**Table 1:** Attempted conditions for copper catalyzed sequential arylation-oxidative dimerization of *o*-bromoanilide **12a** and **12b**

Entries	Metal salts, solvents	Oxidants	Products: yields (%)
1	CuI, THF	AIR	**18**: 16; Complex mixture^a^
2	CuI, toluene	AIR	**19**: 62; **13a + b**: 18; **13c**: 7^a^
3	CuI, toluene	I_2_	**19**: 5; **13a + b**: 53; **13c**: 8^a^
4	CuI, toluene	CI_4_	**19**: 6; **13a + b**: 43; **13c**: 9^a^
5	CuI, toluene	Mn(OAc)_3_–2H_2_O	**19**: 4; **13a + b**: 53; **13c**: 10^a^
6	CuI, toluene	*t*-BuOOH	**19**: 2; **13a + b**: 78; **13c**: 7^a^
7	CuI, benzene	*t*-BuOOH	**19**: 2; **13a + b**: 62; **13c**: 9^a^
8	CuI, mesitylene	*t*-BuOOH	**19**: 2; **13a + b**: 68; **13c**: 8^a^
9	CuOAc, toluene	*t*-BuOOH	**19**: 7; **13a + b**: 58; **13c**: 10^a^
10	CuI, toluene	*t*-BuOOH	**13d + e**: 71; **13f**: 8^a^
11	Pd_2_(dba)_3_, toluene	*t*-BuOOH	**19**: 51; **13a + b** and **13c**: none^b^

Yields represent isolated yields, average of two runs. All arylation reactions were conducted at 2 mmol scale under argon for 5–6 h, then addition of oxidants (2.2 mmol) or open to air

^a^Copper salts (0.1 eq.), LiN(SiMe_3_)_2_ (2.0 eq.), designated solvent, 80 °C

^b^Pd_2_(dba)_3_ (0.05 eq.), Ph_3_P (0.2 eq.), LiN(SiMe_3_)_2_ (2.0 eq.), toluene, 80 °C

**Scheme 5:**
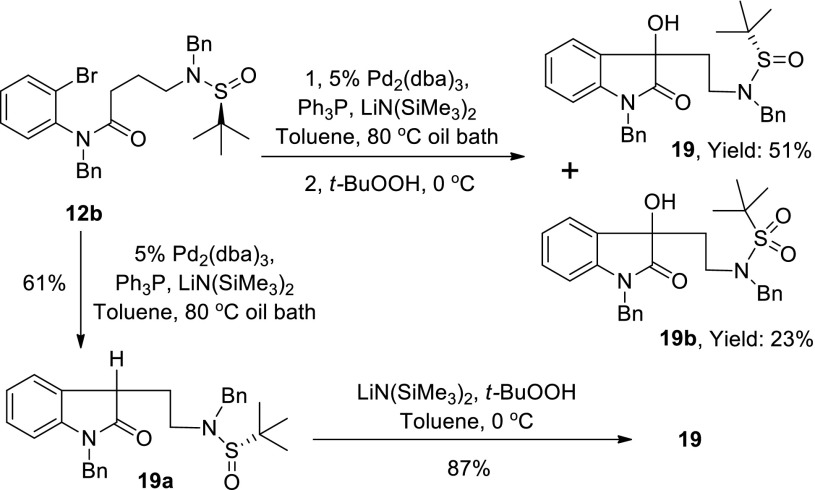
Palladium catalyzed arylation of *o*-bromoanilide **12b**

In order to know whether the oxidative dimerization was promoted solely by *tert*-butyl hydroperoxide, we conducted an arylation with tris(dibenzylideneacetone)dipalladium [Pd_2_(dba)_3_] [61] followed by oxidation with *t*-BuOOH. It was noteworthy that only C3-hydroxy-2-oxindole product (**19**, Table 1, entry 11, as a mixture of diastereomers at C3 position, Scheme 5) was obtained. We next carried out arylation with Pd_2_(dba)_3_ without addition of *t*-BuOOH and successfully obtained oxindole **19a** in 61 % yield. The oxindole (**19a**) was then subjected to oxidation in the presence of LiN(SiMe_3_)_2_ and *t*-BuOOH (see Scheme 5) and provided C3-hydroxy-2-oxindole (**19**) as the sole product.

The fact that no dimerization products (**13a**, **13b** or **13c**) formed in the absence of a copper salt suggests that the copper(II) ion, rather than *tert*-butyl hydroperoxide, plays the role of oxidizing the carbanion to a radical in the dimerization process. *Tert*-butyl hydroperoxide just serves as an oxidant to convert copper(I) to copper(II) in the oxidative dimerization process.

**Scheme 6:**
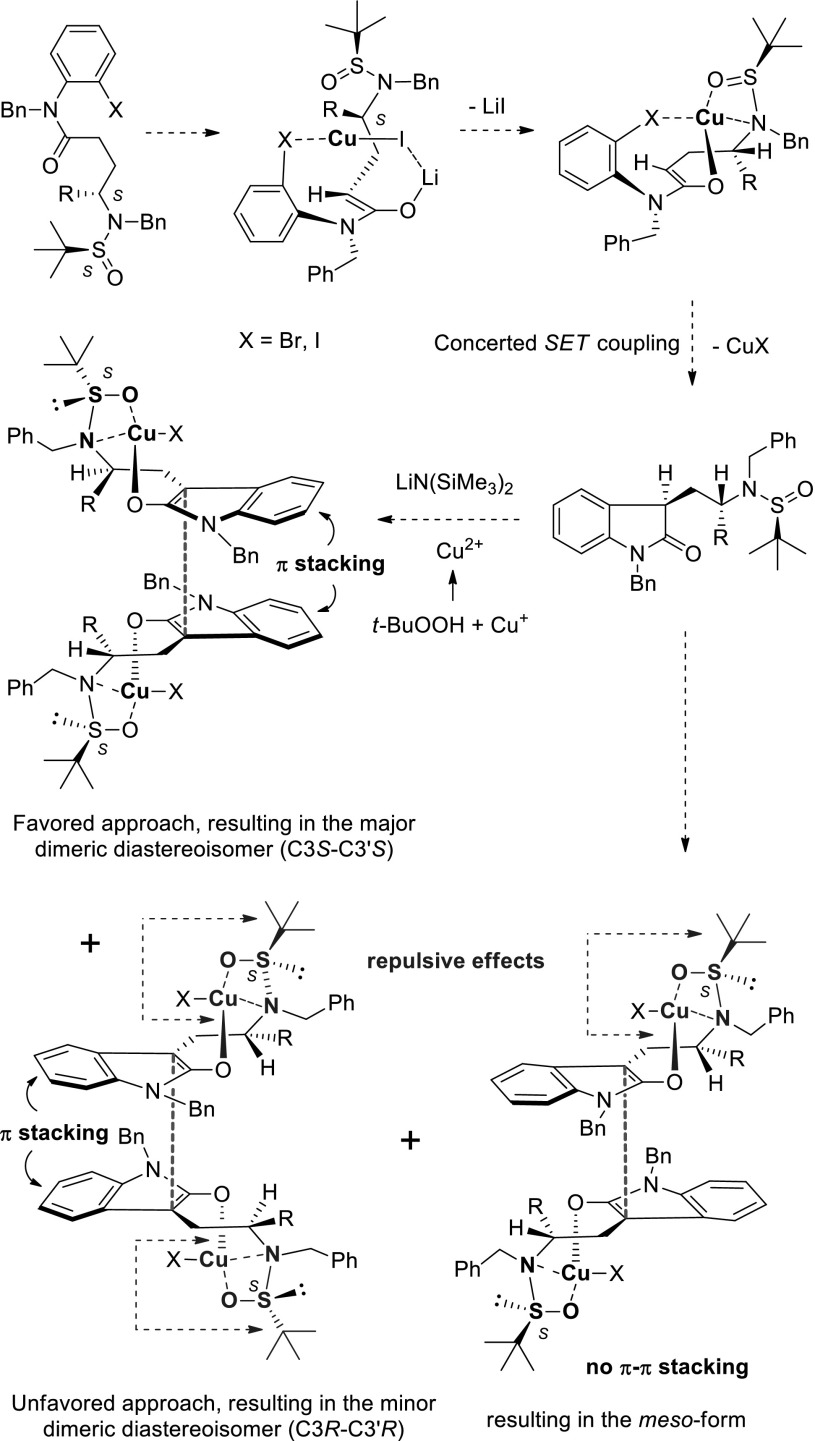
Working hypothesis for copper catalyzed arylation-oxidative dimerization of *o*-haloanilides

Next, we came to the issue of determining diastereoselectivity induced by remote *tert*-butanesulfinamide moiety. Although the major product (**13a** and **13b**) of this reaction was an unseparatable mixture of diastereoisomers (C3*S*–${\text{C}}{3}^{'}$*S* and C3*R*–${\text{C}}{3}^{'}$*R*) by silica gel column chromatography, the ratio could be readily determined by proton nuclear magnetic resonance (^1^H-NMR; 84:16, see Electronic supplementary material). The enantioselectivity for the formation of vicinal C3–${\text{C}}{3}^{'}$ quaternary carbon center was also determined after oxidation of the *tert*-butylsulfinyl group with 3-chloroperbenzoic acid (*m*-CPBA) in dichloromethane. A 66 % enantioselective excess was recorded (see Electronic supplementary material for chiral HPLC analysis) under our optimum reaction condition (Table 1, entry 6). The enantioselectivity for compounds **13d** and **13e** was also determined after oxidation of the *tert*-butylsulfinyl group with 3-chloroperbenzoic acid (*m*-CPBA) and relatively low enantioselective excess (35 %) was observed. To the best of our knowledge, this is the first example of copper catalyzed sequential arylation–dimerization of an *o*-bromoanilide, a high-yield procedure and also the first asymmetric oxidative dimerization of an oxindole derivative with good diastereoselectivity (*dr* >10:1) and enantioselectivity (*ee* = 66 %). The absolute stereochemistry was late confirmed by the total synthesis of (+)-chimonanthine (Scheme 10). A working hypothesis was proposed for prediction of C3–${\text{C}}{3}^{'}$ configuration in Scheme 6. π–π Stack (for a review for π–π.stack in asymmetric synthesis, see: [75, 76]) might play important role for the enantioselectivity as well as diastereoselectivity.

**Scheme 7:**
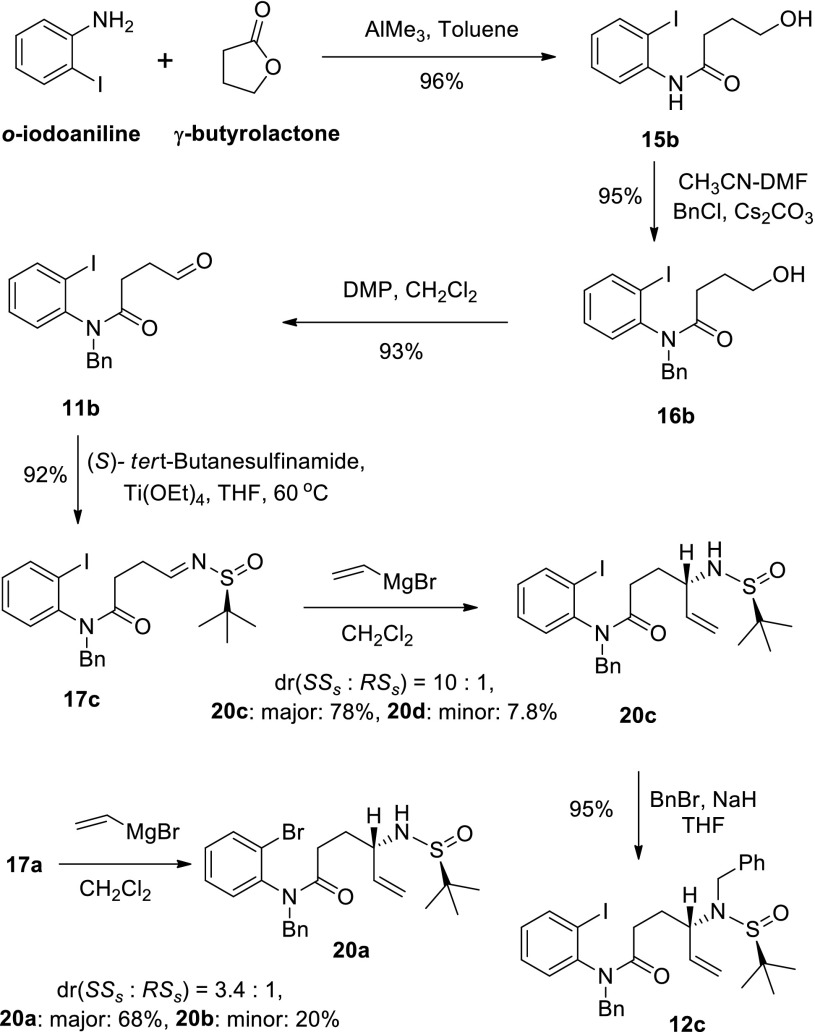
Synthesis of amide **12c**

**Scheme 8:**
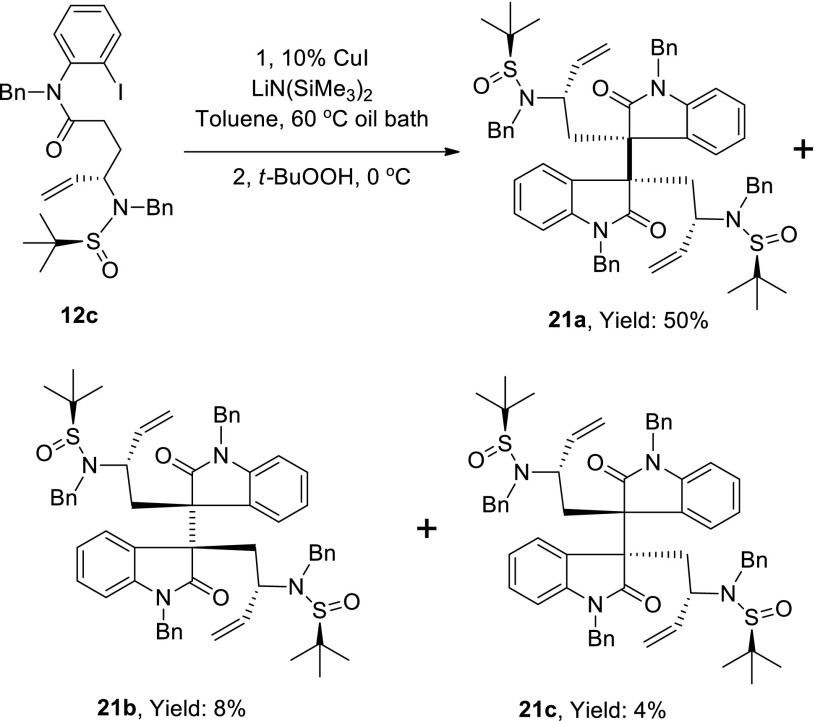
Copper catalyzed sequential arylation-oxidative dimerization of *o*-bromoanilide **12c**

**Scheme 9:**
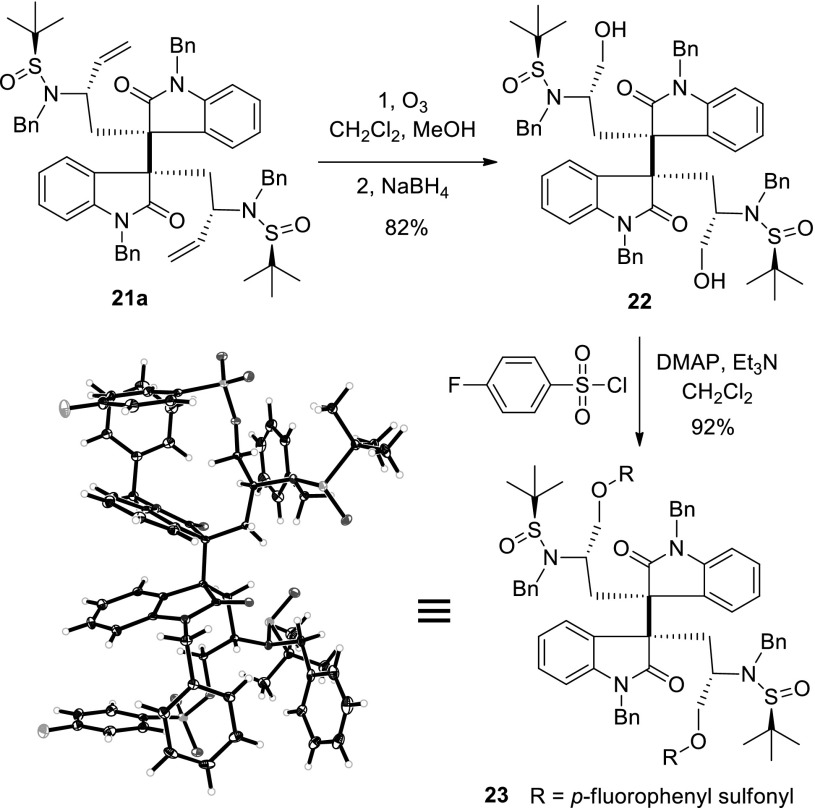
Determination of the absolute configuration of **21a** by X-ray crystallography with Mo Kα radiation

In order to access the starting material for the synthesis of ditryptophenaline, we started the synthesis of amide **12c** (Scheme 7). Nucleophilic addition of vinyl magnesium bromide to aldimine **17a** unfortunately provided two isolatable diastereisomers (**20a** and **20b**) in a ratio of 3.4–1 (see Scheme 7). This problem was soon fixed by addition of Grignard reagent to aldimine **17c**, a surrogate prepared from *o*-iodoaniline. Better diastereoselectivity (*dr* = 10:1, see Scheme 7) was obtained by 1,2-addition of vinyl magnesium bromide to *o*-iodoanilide **17c**. After benzylation, we obtained **12c** in 58 % yield in six steps from iodoaniline. The absolute configuration for the newly generated chiral center of compound **12c** was deduced by Cram’s chelation model [77] and late confirmed by X-ray crystallography (Schemes 8, 9) and our total synthesis of (−)-ditryptophenaline (Scheme 11).

We next conducted the arylation–oxidative dimerization reaction with substrate **12c** under the optimized condition. This reaction afforded the dimerization products in good yield, and all dimeric diastereomers were isolated by silica gel column chromatography, with the desired isomer (**21a**) being obtained as the major product and in a 50 % yield (Scheme 8). It was noteworthy that this sequential arylation–oxidative dimerization process could be conducted on multi-gram scale in the presence of only catalytic amount of cuprous iodide (10 %). In order to determine the absolute configuration of compound **21a**, we converted it to sulfonyl ester **23** and successfully obtained a suitable crystal for X-ray crystallography. The absolute configuration was confirmed by X-ray crystallography with Mo Kα radiation (Scheme 9).

**Scheme 10:**
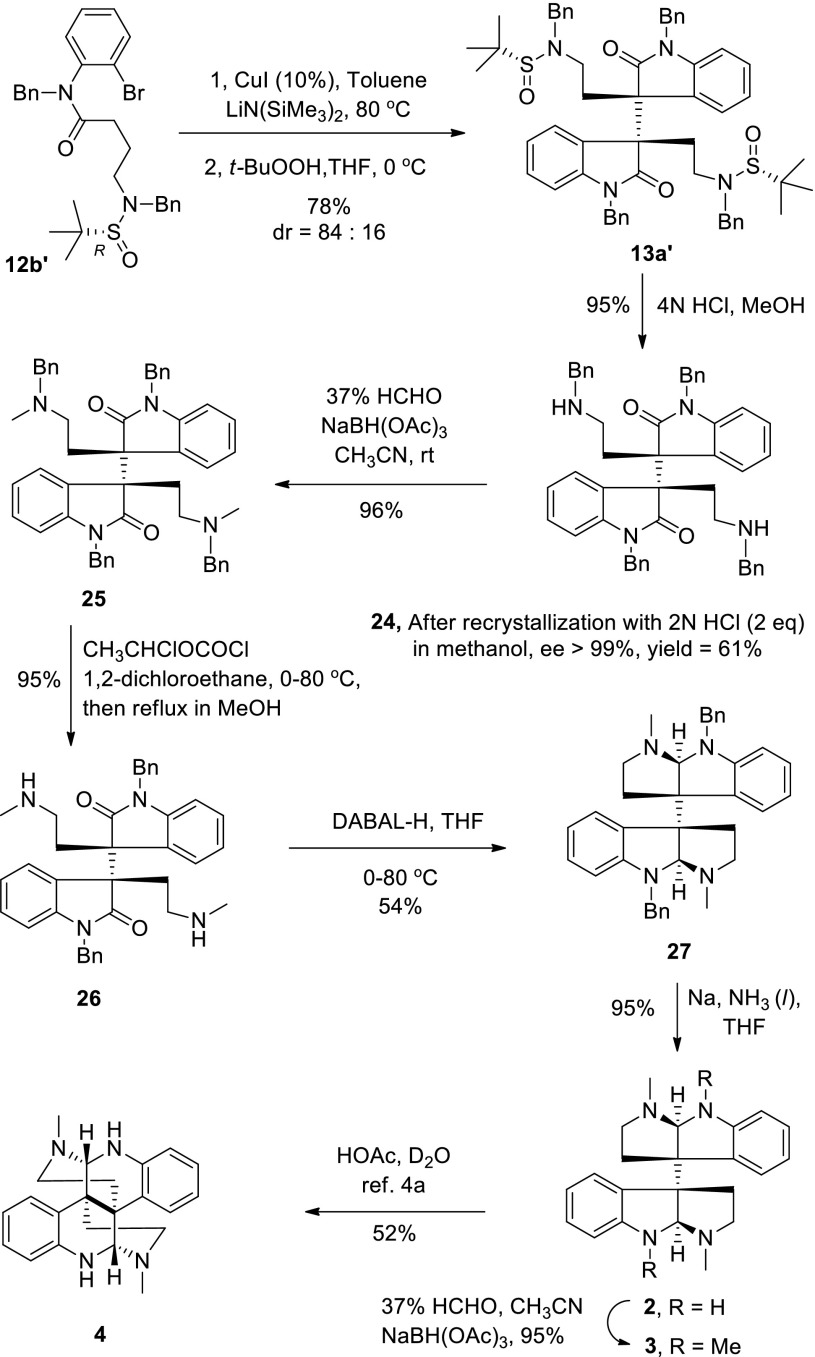
Total synthesis of (+)-chimonanthine, (+)-folicanthine and (−)-calycanthine

Having successfully established the cascade process, we next initiated the synthesis of (+)-chimonanthine and its related natural dimeric HPI alkaloids. Based on our working hypothesis (Scheme 6), we used (*R*)-(+)-*tert*-butanesulfinamide as a chiral auxiliary. Treatment of **11a** with (*R*)-(+)-*tert*-butanesulfinamide in the presence of titanium ethoxide [Ti(OEt)_4_] in THF afforded **17a′** in 94 % yield. After reduction and benzylation, amide **12b′** was synthesized in 68 % overall yield from *o*-bromoaniline. The key arylation–oxidative dimerization of **12b′** (at multi-gram scale, 5.42 g, 10 mmol, see Scheme 10) afforded **13a′** + **13b′** in 78 % yield. Treatment of this diastereomeric mixture with 4 *N* HCl in methanol gave diamine **24** in a 95 % yield. After recrystallization in methanol with 2 *N* aqueous solution of HCl (2.0 eq.), the isomeric purity of di-amine **24** was greater than 99 %, as determined by HPLC analysis (Scheme 10, see supporting information). The reductive amination of **24** with formaldehyde and NaBH(OAc)_3_ provided diamide **25** (96 %) [19, 28]. Selective debenzylation of the amine benzyl group in **25** by treatment with α-chloroethyl chloroformate (ACE-Cl) [77] followed by reflux in methanol afforded diamine **26** (95 %). Reductive aminocyclization of diamine **26** in THF in the presence of diisobutylaluminum hydride (DIBAL-H) provided desired HPI precursor (**27**) in 54 % yield. Finally, removal of the benzyl protecting groups by a Birch reduction [19] afforded (+)-chimonanthine in 95 % yield ([α]_D_ = +285, *c* 0.12, EtOH, lit. [71], [α]_D_ = +279, *c* 0.1, EtOH, lit. [28], [α]_D_ = +254, *c* 1.0, EtOH). The NMR spectra of our synthetic sample were in fully agreement with those reported in the literature [19, 20, 28].

Treatment of chimonanthine with formaldehyde in the presence of sodium triacetoxyborohydride [28] then furnished (+)-folicanthine (95 %, [α]_D_ = +315, *c* 0.10, MeOH, lit. [70], [α]_D_ = +318, *c* 0.11, MeOH, lit. [71], [α]_D_ = +314, *c* 0.25, MeOH). Upon exposure of chimonanthine to acid at reflux for 8 h [19, 28] (−)-calycanthine ([α]_D_ = −615, *c* 0.15, EtOH, lit. [28], [α]_D_ = −612, *c* 0.18, EtOH) was obtained in 52 % isolated yield (Scheme 10).

**Scheme 11:**
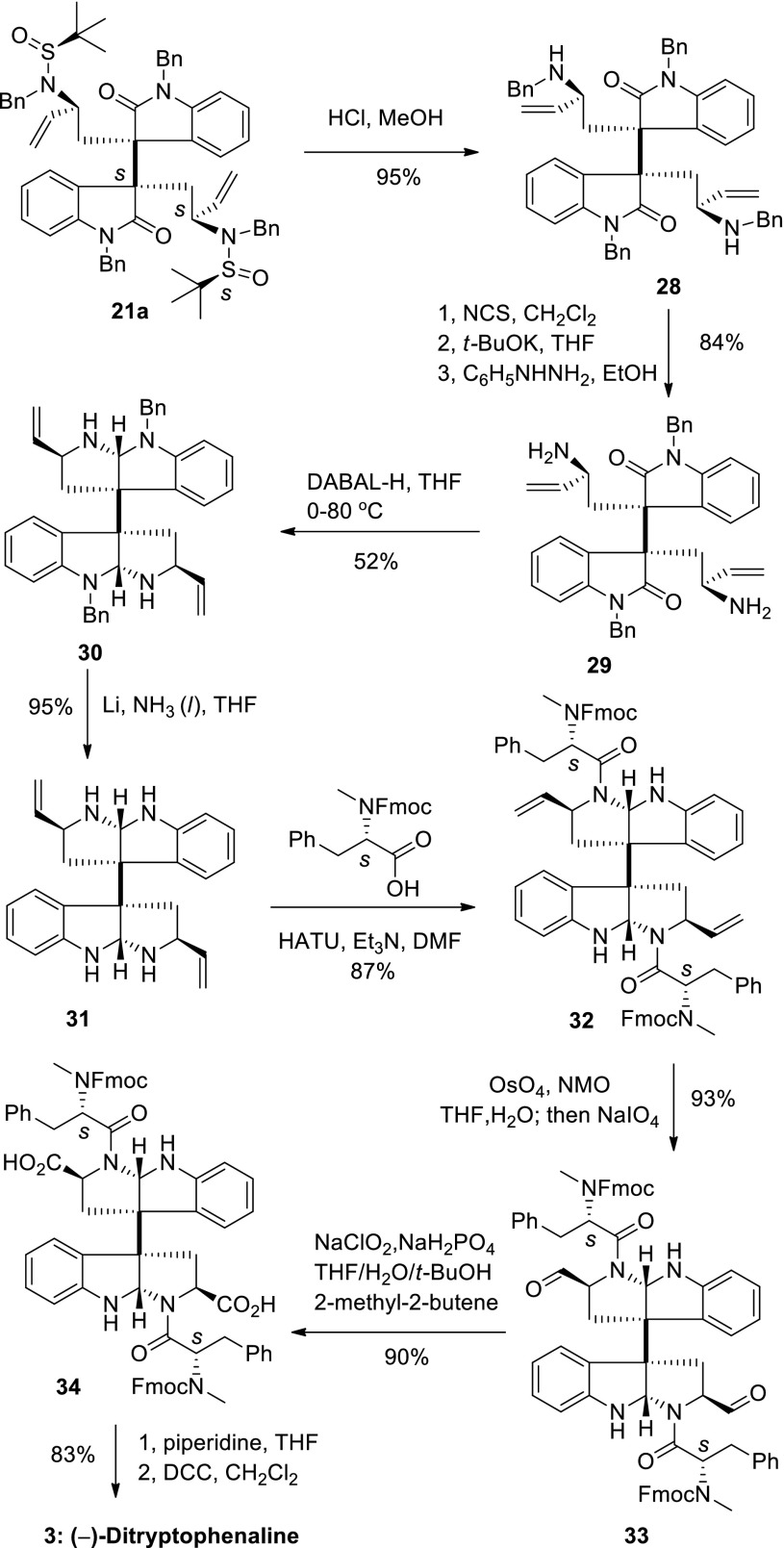
Total synthesis of (−)-ditryptophenaline

Starting from intermediate **21a**, synthesis of (−)-ditryptophenaline was also investigated (Scheme 11). Removal of the *tert*-butanesulfinyl group by treatment of **21a** with 4 *N* HCl in methanol afforded diamine **28**. Selectively deprotection of the benzyl groups attaching to amines was difficult due to the presence of double bonds but finally achieved after many experiments. Treatment of diamine **28** with *N*-chlorosuccinamide, followed by deprotonation with *t*-BuOK provided imine [79], which, upon exposure to phenylhydrazine in ethanol [80], gave the primary diamine **29** in 84 % overall yield. After reductive aminocyclization with DABAL-H and debenzylation with metal lithium in liquid ammonia (Scheme 11), intermediate **31** was obtained in 49 % yield over two steps. Condensation of intermediate **31** with Fmoc-methylphenaline afforded compound **32** (FMOC-(*S*)-MePhe–OH, HATU and Et_3_N in DMF) in 87 % yield [33]. Cleavage of the double bond led to aldehyde **33**, and oxidation with buffered NaClO_2_ provided diacid **34** [21]. Finally the diacid (**34**) was converted to (−)-ditryptophenaline ([α]_D_ = −292, *c* 0.40, CH_2_Cl_2_, lit. [29], [α]_D_ = −292, *c* 0.97, CH_2_Cl_2_, lit. [49], [α]_D_ = −291, *c* 0.41, CH_2_Cl_2_) in 83 % overall yield by following Overman’s procedure, deprotection with piperidine in THF and cyclization with DCC in dichloromethane (Scheme 11) [21]. The NMR spectra of our synthetic sample were in complete agreement with the reported data.

In conclusion, we have developed the first copper catalyzed arylation–oxidative dimerization of *o*-haloanilides with a remote assistance of an intramolecular sulfinyl amide unit. Based on this method, a general synthetic strategy has been successfully established for the total synthesis of chimonanthine, folicanthine, calycanthine and ditryptophenaline. This copper catalyzed sequential arylation–oxidative dimerization should find further application in the synthesis of HPI alkaloids as well as its medicinally interesting analogues.

## Experimental Section

### General Experimental

^1^H-NMR spectra were recorded on a Bruker Avance 300 or 400 spectrometer at 300 or 400 MHz. Carbon-13 nuclear magnetic resonance (^13^C-NMR) was recorded on Bruker Avance 300 or 400 spectrometer at 75 or 100 MHz. Chemical shifts are reported as δ values in parts per million (ppm) relative to tetramethylsilane for all recorded NMR spectra. Low-resolution mass spectra were recorded on a VG Auto Spec-3000 magnetic sector MS spectrometer. High resolution mass spectra were taken on AB QSTAR Pulsar mass spectrometer or Agilent G6230 TOF MS spectrometer. Chiral HPLC analyses were performed on Agilent 1100 series with a tunable UV detector at wavelength λ = 254 nm. Melting points were determined on a capillary melting point apparatus and are uncorrected. Optical rotations were obtained on a UV-210A spectrometer. Starting materials and reagents used in reactions were obtained commercially from Acros, Aldrich, J&K and were used without purification, unless otherwise indicated. THF and toluene used in the reactions were dried by distillation over metallic sodium; dichloromethane were distilled over P_2_O_5_. Unless otherwise stated, all reactions were conducted in dried glassware under a positive pressure of dry nitrogen or argon. Silica gel (Qingdao, 200–300 mesh) was used for column chromatography.

### Synthesis of Compound **15a**

2-Bromoaniline (12.04 g, 70.0 mmol) was dissolved in toluene (200 mL) under nitrogen. To this mixture, a solution of trimethylaluminum in toluene (2.0 M, 49 mL, 98.0 mmol, 1.4 eq.) was added dropwise at 0 °C. The resulting mixture was then stirred at room temperature for 45 min. γ-Butyrolactone (7.5 mL, 98 mmol, 1.4 eq.) was added via syringe and the reaction mixture was stirred at room temperature overnight. The solidified mixture was then cooled to 0 °C, and HCl (1 N, 150 mL) was added slowly. After 30 min, the resulting mixture was extracted with ethyl acetate (4 × 80 mL). The combined organic phases were washed with brine (50 mL) and dried over anhydrous sodium sulfate. After removal of the solvent, the crude products were chromatographed on silica gel (petroleum ether 60–90 °C:ethyl acetate = 1:2) to provide amide **15a** (17.34 g, 96 %) as a white solid.

m.p. 65–67 °C. *R*_f_ 0.55 (petroleum ether:ethyl acetate = 1:2). *FTIR* (KBr, thin film) cm^−1^ 3271, 2943, 2875, 1660, 1527, 1430, 1285, 1051, 755, 673. ^*1*^*H-NMR* (300 MHz, CDCl_3_), δ (ppm) 8.27 (1H, *d*, *J* = 7.8 Hz), 7.81 (1H, *brs*), 7.53 (1H, *d*, *J* = 7.8 Hz), 7.30 (1H, *t*, *J* = 7.8 Hz), 6.98 (1H, *t*, *J* = 7.8 Hz), 3.75 (2H, *t*, *J* = 5.7 Hz), 2.59 (3H, *t*, *J* = 6.9 Hz), 2.05–1.91 (2H, *m*). ^*13*^*C-NMR* (75 MHz, CDCl_3_) δ (ppm) 171.73, 135.72, 132.41, 128.48, 125.48, 122.42, 113.71, 62.06, 34.80, 28.04. +TOF-MS *m*/*z* (%) 282 (M^+^+1+Na, 100 %), 281 (M^+^+Na, 8 %), 280 (M^+^+Na, 96 %), 260 (42), 258 (40), 240 (20), 200 (9), 174 (29), 172 (30). *HRMSm*/*z* Found 258.0124, Calcd. for C_10_H_13_NO_2_Br [M+1]^+^ 258.0129.

### Synthesis of Compound **16a**

To a mixture of cesium carbonate (17.43 g, 53.5 mmol, 1.5 eq.) in acetonitrile (90 mL) and *N*,*N*-dimethylformamide (DMF, 45 mL) at 0 °C was added dropwise a solution of amide **15a** (9.20 g, 35.7 mmol) in acetonitrile (10 mL) and DMF (5 mL). Benzyl bromide (6.4 mL, 53.5 mmol, 1.5 eq.) was then added. The resulting mixture was allowed to stir at room temperature for 8 h. After filtration through a short column of silica gel and washed with ethyl acetate (180 mL), the combined organic phases were concentrated under reduced pressure and the residue was chromatographed on silica gel (petroleum ether 60–90 °C:ethyl acetate = 1:1) to afford alcohol **16a** (11.86 g, 96 %) as a colorless oil.

*R*_f_ 0.60 (petroleum ether:ethyl acetate = 1:1). *FTIR* (KBr, thin film) cm^−1^ 3417, 2963, 1651, 1402, 1270, 1203, 1050, 728. ^*1*^*H-NMR* (300 MHz, CDCl_3_), δ (ppm) 7.68 (1H, *dd*, *J* = 2.7, 7.8 Hz), 7.37–7.12 (7H, *m*), 6.77 (1H, *dd*, *J* = 3.3, 7.8 Hz), 5.64 (1H, *d*, *J* = 14.4 Hz), 4.01 (1H, *d*, *J* = 14.4 Hz), 3.71–3.55 (2H, *m*), 2.89–2.68 (1H, *m*), 2.14 (2H, *t*, *J* = 6.6 Hz), 1.98–1.78 (2H, *m*). ^*13*^*C-NMR* (75 MHz, CDCl_3_) δ (ppm) 173.43, 140.71, 136.99, 134.07, 131.52, 130.03, 129.46, 128.55, 127.72, 123.86, 62.78, 51.77, 32.06, 27.85. +TOF-MS *m*/*z* (%) 372 (M^+^+1+Na, 99 %), 371 (M^+^+Na, 9 %), 370 (M^+^+Na, 100 %), 350 (32), 348 (34), 330 (2), 264 (3). *HRMSm*/*z* Found 370.0410, Calcd. for C_17_H_18_NO_2_NaBr [M+23]^+^ 370.0418.

### Synthesis of Compound **11a**

Alcohol **16a** (11.86 g, 34.1 mmol) was dissolved in dichloromethane (120 mL). To this solution, a powder of Dess-Martin periodinane (21.67 g, 51.1 mmol, 1.5 eq.) was added. The resulting mixture was then stirred at room temperature for 6 h. After filtration through a short column of silica gel and washed with ethyl acetate (150 mL), the combined organic phases were concentrated under reduced pressure and the residue was chromatographed on silica gel (petroleum ether 60–90 °C:ethyl acetate = 3:1) to afford aldehyde **11a** (10.85 g, 92 %) as white plates.

m.p. 52–54 °C. *R*_f_ 0.53 (petroleum ether:ethyl acetate = 3:1). *FTIR* (KBr, thin film) cm^−1^ 3419, 3060, 2921, 2827, 2730, 1657, 1400, 1265, 1196, 1020, 727. ^*1*^*H-NMR* (300 MHz, CDCl_3_), δ (ppm) 9.81 (1H, *s*), 7.72–7.62 (1H, *m*), 7.38–7.12 (7H, *m*), 6.92–6.82 (1H, *m*), 5.62 (1H, *d*, *J* = 14.4 Hz), 4.03 (1H, *d*, *J* = 14.4 Hz), 2.91 (1H, *ddd*, *J* = 6.9, 7.8, 18.6 Hz), 2.68 (1H, *dt*, *J* = 6.3, 18.6 Hz), 2.42–2.16 (2H, *m*). ^*13*^*C-NMR* (75 MHz, CDCl_3_), δ (ppm) 200.99, 171.12, 140.45, 136.88, 133.99, 131.60, 130.08, 129.29, 128.62, 128.50, 127.64, 123.87, 51.75, 38.98, 27.18. +TOF-MS *m*/*z* (%) 370 (M^+^+1+Na, 98 %), 369 (M^+^+Na, 13 %), 368 (M^+^+Na, 100 %), 348 (81), 346 (90), 332 (30), 330 (31), 262 (3). *HRMSm*/*z* Found 368.0266, Calcd. for C_17_H_16_NO_2_NaBr [M+Na]^+^ 368.0262.

### Synthesis of Compound **17b**

To a solution of aldehyde **11a** (5.85 g, 16.8 mmol) in toluene (60 mL) was added a powder of (*S*)-(−)-*tert*-butanesulfinamide (4.1 g, 33.7 mmol, 2.0 eq.) and KHSO_4_ (4.56 g, 33.7 mmol, 2.0 eq.). The resulting mixture was stirred at 45 °C for 3 h. After filtration through a short column of silica gel and washed with ethyl acetate (80 mL), the combined organic phases were concentrated under reduced pressure and the residue was flash chromatographed on silica gel (petroleum ether 60–90 °C:ethyl acetate = 3:1) to afford the sulfinyl imine (**17a**: 7.36 g, 97 %) as colorless syrup. The sulfinyl imine was re-dissolved in anhydrous methanol (60 mL) and the resulting solution was cooled to 0 °C. A powder of sodium borohydride (1.87 g, 49.2 mmol, 3.0 eq.) was added in small portion over a period of 30 min. The resulting mixture was then allowed to stir at 0 °C for 4 h. After which, a saturated solution of NH_4_Cl (20 mL) was introduced and the resulting mixture was concentrated (ca. 20–30 mL). The mixture was diluted with water (100 mL) and extracted with ethyl acetate (4 × 50 mL). The combined organic phases were washed with brine (60 mL) and dried over anhydrous sodium sulfate. After removal of the solvent, the crude products were chromatographed on silica gel (petroleum ether 60–90 °C:ethyl acetate = 1:2) to provide sulfinamide **17b** (6.6 g, 89 %) as a white solid.

m.p. 102–103 °C. $[\alpha ]_{\text{D}}^{20}$ +71 (c 0.90, CHCl_3_). *R*_f_ 0.45 (petroleum ether:ethyl acetate = 1:2). *FTIR* (KBr, thin film) cm^−1^ 3244, 3063, 2950, 2868, 1662, 1559, 1468, 1398, 1266, 1203, 1063, 947, 767, 732, 628. ^*1*^*H-NMR* (as a mixture of rotamers, 300 MHz, CDCl_3_), δ (ppm) 7.71–7.61 (1H, *m*), 7.31–7.12 (7H, *m*), 6.81–6.70 (1H, *m*), 5.58 (1H, *d*, *J* = 14.1 Hz), 4.00 (0.5H, *d*, *J* = 14.1 Hz) [3.99 (0.5H, *d*, *J* = 14.1 Hz)], 3.42–3.23 (1H, *m*), 3.22–3.10 (1H, *m*), 3.10–2.94 (1H, *m*), 2.11–1.97 (2H, *m*), 1.93–1.78 (2H, *m*), 1.13 (9H, *s*). ^*13*^*C-NMR* (rotamer in brackets, 75 MHz, CDCl_3_), δ (ppm) 172.16, 140.67, 137.02, 134.00 (133.94), 131.57 (131.50), 129.94, 129.41, 128.47, 127.63, 123.85, 55.64, 51.58, 45.07 (44.99), 31.62, 26.15, 22.71. +TOF-MS *m*/*z* (%) 475 (M^+^+1+Na, 60 %), 474 (M^+^+Na, 8 %), 473 (M^+^+Na, 65 %), 453 (98), 452 (12), 451 (100), 435 (25), 433 (23), 402 (4), 400 (4), 294 (2), 282 (6), 280 (5), 262 (2). *HRMSm*/*z* Found 473.0878, Calcd. for C_21_H_27_N_2_O_2_NaSBr [M+Na]^+^ 473.0874.

### Synthesis of Compound **12a**

To a mixture of sodium hydride (60 % in mineral oil, 1.22 g, 30.5 mmol, freshly washed with anhydrous hexane three times under nitrogen) in anhydrous THF (50 mL) at 0 °C was added a solution of sulfonamide **17b** (10.6 g, 23.5 mmol) in THF (50 mL) via syringe. After stirring at 0 °C for 30 min, methyl iodide (1.9 mL, 30.6 mmol) was added. The resulting mixture was then stirred at room temperature for 15 h under nitrogen. A powder of NH_4_Cl (1.62 g, 30.0 mmol) was added and the mixture was stirred for 10 min. After concentrated under reduced pressure, the residue was diluted with water (100 mL) and extracted with ethyl acetate (3 × 50 mL). The combined organic phases were washed with brine (20 mL) and dried over anhydrous Na_2_SO_4_. After filtration, the solvent was removed under reduced pressure and the residue was chromatographed on silica gel (petroleum ether 60–90 °C:ethyl acetate = 1:1) to afford the product (**12a**: 10.03 g, 92 %) as a white solid.

m.p. 60–62 °C. *R*_f_ 0.52 (petroleum ether:ethyl acetate = 1:1). ^*1*^*H-NMR* (300 MHz, CDCl_3_), δ (ppm) 7.65–7.58 (1H, *m*), 7.28–7.08 (7H, *m*), 6.78–6.68 (1H, *m*), 5.57 (1H, *dd*, *J* = 2.1, 14.4 Hz), 3.95 (1H, *d*, *J* = 14.4 Hz), 3.01–2.82 (2H, *m*), 2.52 (3H, *s*), 2.01–1.78 (4H, *m*), 1.09 (9H, *s*). ^*13*^*C-NMR* (75 MHz, CDCl_3_), δ (ppm) 171.77, 140.51, 136.93, 133.86, 131.42, 129.86, 129.24, 128.43, 128.36, 127.51, 123.76, 58.11, 53.94 (53.57), 51.41, 32.65 (32.48), 23.64, 23.43. +TOF-MS *m*/*z* (%) 489 (M^+^+1+Na, 100 %), 488 (M^+^+Na, 12 %), 487 (M^+^+Na, 98 %), 467 (18), 465 (16), 432 (2), 383 (1), 375 (2), 361 (21), 359 (20), 276 (3), 274 (2). *HRMSm*/*z* Found 487.1023, Calcd. for C_22_H_29_N_2_O_2_NaSBr [M+Na]^+^ 487.1030.

### Synthesis of Compound **12b**

To a mixture of sodium hydride (60 % in mineral oil, 1.32 g, 33 mmol, 1.5 eq., freshly washed with anhydrous hexane three times under nitrogen) in anhydrous THF (30 mL) at 0 °C was added a solution of sulfonamide **17b** (9.9 g, 22 mmol) in THF (80 mL) via syringe. After stirring at 0 °C for 30 min, benzyl bromide (3.9 mL, 33 mmol, 1.5 eq.) was added. The resulting mixture was then stirred at 0 °C for 2 h, then at room temperature for 12 h under nitrogen. A powder of NH_4_Cl (1.62 g, 30.0 mmol) was added and the mixture was stirred for 10 min. After concentrated under reduced pressure, the residue was diluted with water (60 mL) and extracted with ethyl acetate (3 × 50 mL). The combined organic phases were washed with brine (30 mL) and dried over anhydrous Na_2_SO_4_. After filtration, the solvent was removed under reduced pressure and the residue was chromatographed on silica gel (petroleum ether 60–90 °C:ethyl acetate = 2:1) to afford the product (**12b**: 11.2 g, 94 %) as an off-yellow oil.

$[\alpha ]_{\text{D}}^{20}$ −3.9 (c 0.40, CHCl_3_). *R*_f_ 0.51 (petroleum ether:ethyl acetate = 2:1). *FTIR* (KBr, thin film) cm^−1^ 3421, 2960, 1665, 1467, 1396, 1278, 1204, 1070, 1024, 928, 700. ^*1*^*H-NMR* (as a mixture of rotamers, 300 MHz, CDCl_3_), δ (ppm) 7.71–7.61 (1H, *m*), 7.37–7.11 (12H, *m*), 6.77–6.67 (1H, *m*), 5.59 (0.5H, *d*, *J* = 14.4 Hz) [ 5.59 (0.5H, *d*, *J* = 14.1 Hz)], 4.26 (1H, *d*, *J* = 15.6 Hz), 4.10 (0.5H, *d*, *J* = 15.6 Hz) [ 4.08 (0.5H, *d*, *J* = 15.3 Hz)], 3.98 (0.5H, *d*, *J* = 14.1 Hz) [3.97 (0.5H, *d*, *J* = 14.4 Hz)], 3.03–2.85 (1H, *m*), 2.84–2.68 (1H, *m*), 1.97–1.76 (4H, *m*), 1.16 (9H, *s*). ^*13*^*C-NMR* (rotamer in brackets, 75 MHz, CDCl_3_), δ (ppm) 171.69, 140.56, 137.24, 137.02, 133.92, 131.52, 131.45, 129.88, 129.32, 128.54, 128.44, 127.57, 127.35, 123.85, 58.22, 51.47 (51.07), 48.23 (47.92), 31.79 (31.71), 23.93 (23.81), 23.39. EI–MS *m*/*z* (%) 543 (M^+^+2, 6 %), 541 (M^+^+1, 6 %), 486 (10), 486 (34), 484 (30), 430 (25), 395 (26), 330 (16), 263 (31), 261 (30), 212 (17), 174 (16), 147 (11), 91 (48), 90 (100), 85 (4), 76 (12), 57 (29). *HRMSm*/*z* Found 540.1448, Calcd. for C_28_H_33_N_2_O_2_SBr [M]^+^ 540.1446.

### Synthesis of Compound **15b**

2-Iodoaniline (21.9 g, 100.0 mmol) was dissolved in toluene (300 mL) under nitrogen. To this mixture, a solution of trimethylaluminum in toluene (2.0 M, 60 mL, 120 mmol, 1.2 eq.) was added dropwise at 0 °C. The resulting mixture was then stirred at room temperature for 45 min, after which, γ-butyrolactone (9.2 mL, 120 mmol, 1.2 eq.) was added via syringe and the reaction mixture was stirred at room temperature overnight. The solidified mixture was then cooled to 0 °C and HCl (1 N, 360 mL) was added slowly. After 1 h, the resulting mixture was extracted with ethyl acetate (4 × 150 mL). The combined organic phases were washed with brine (100 mL) and dried over anhydrous sodium sulfate. After removal of the solvent, the crude products were chromatographed on silica gel (petroleum ether 60–90 °C:ethyl acetate = 1:2) to provide amide **15b** (29.30 g, 96 %) as a white solid.

m.p. 65–66 °C. *R*_f_ 0.59 (petroleum ether:ethyl acetate = 1:2). *FTIR* (KBr, thin film) cm^−1^ 3268, 2935, 1658, 1528, 1430, 1287, 1059, 749, 664. ^*1*^*H-NMR* (400 MHz, CDCl_3_), δ (ppm) 8.02 (1H, *d*, *J* = 8.0 Hz), 7.76 (1H, *s*), 7.75 (1H, *d*, *J* = 8.0 Hz), 7.30 (1H, *t*, *J* = 7.6 Hz), 6.83 (1H, *t*, *J* = 7.6 Hz), 3.72 (2H, *t*, *J* = 6.0 Hz), 3.32 (1H, *brs*), 2.56 (3H, *t*, *J* = 6.9 Hz), 2.05–1.91 (2H, *m*). ^*13*^*C-NMR* (100 MHz, CDCl_3_) δ (ppm) 171.92, 138.90, 138.16, 129.16, 126.43, 123.11, 91.14, 61.76, 34.52, 28.06. EI–MS *m*/*z* (%) 304 (M^+^, 28 %), 268 (89 %), 224 (12 %), 182 (30), 180 (40), 152 (10), 128 (6), 104 (12), 91 (100). *HRMSm*/*z* Found 304.9969, Calcd. for C_10_H_12_NO_2_I [M]^+^ 304.9913.

### Synthesis of Compound **16b**

To a mixture of amide **15b** (29.3 g, 96 mmol), cesium carbonate (46.9 g, 144 mmol, 1.5 eq.) in acetonitrile (200 mL) and *N*,*N*-dimethylformamide (DMF, 100 mL) at 0 °C was added dropwise a solution of benzyl bromide (17.1 mL, 144 mmol, 1.5 eq.) was then added. The resulting mixture was then stirred at room temperature for 12 h. After filtration through a short column of silica gel and washed with ethyl acetate (200 mL), the combined organic phases were concentrated under reduced pressure and the residue was chromatographed on silica gel (petroleum ether 60–90 °C:ethyl acetate = 1:1) to afford alcohol **16b** (36.03 g, 95 %) as a white powder.

m.p. 72–74 °C. *R*_f_ 0.55 (petroleum ether:ethyl acetate = 1:1). *FTIR* (KBr, thin film) cm^−1^ 3500, 2940, 2871, 1631, 1405, 1322, 1051, 1008, 731, 616, 491. ^*1*^*H-NMR* (400 MHz, CDCl_3_), δ (ppm) 7.93 (1H, *dd*, *J* = 1.2, 8.0 Hz), 7.28–7.15 (6H, *m*), 7.03 (1H, *ddd*, *J* = 1.2, 7.8, 8.0 Hz), 6.71 (1H, *dd*, *J* = 1.2, 7.8 Hz), 5.67 (1H, *d*, *J* = 14.4 Hz), 3.91 (1H, *d*, *J* = 14.4 Hz), 3.70–3.53 (2H, *m*), 3.12–3.04 (1H, *m*), 2.10 (2H, *t*, *J* = 6.6 Hz), 1.92–1.78 (2H, *m*). ^*13*^*C-NMR* (100 MHz, CDCl_3_) δ (ppm) 173.18, 143.80, 140.31, 136.83, 130.83, 130.00, 129.43, 129.35, 128.47, 127.65, 100.36, 62.51, 51.81, 32.23, 27.79. EI–MS *m*/*z* (%) 395 (M^+^, 10 %), 351 (3 %), 309 (20 %), 224 (4), 203 (5), 182 (15), 180 (30), 152 (6), 134 (9), 119 (17), 104 (7), 91 (100), 77 (14). *HRMSm*/*z* Found 395.0378, Calcd. for C_17_H_18_NO_2_I [M]^+^ 395.0382.

### Synthesis of Compound **11b**

Alcohol **16b** (36.0 g, 91.1 mmol) was dissolved in dichloromethane (300 mL). To this solution, a powder of Dess-Martin periodinane (46.4 g, 109.3 mmol, 1.2 eq.) was added. The resulting mixture was then stirred at room temperature for 3 h. After filtration through a short column of silica gel and washed with ethyl acetate (150 mL), the combined organic phases were concentrated and the residue was chromatographed on silica gel (petroleum ether 60–90 °C:ethyl acetate = 3:1) to afford aldehyde **11b** (32.94 g, 92 %) as pale yellow plates.

m.p. 76–78 °C. *R*_f_ 0.53 (petroleum ether:ethyl acetate = 3:1). *FTIR* (KBr, thin film) cm^−1^ 3433, 3056, 2826, 1713, 1651, 1400, 1268, 1017, 771, 724. ^*1*^*H-NMR* (400 MHz, CDCl_3_), δ (ppm) 9.77 (1H, *s*), 7.93 (1H, *dd*, *J* = 1.2, 8.0 Hz), 7.29–7.13 (6H, *m*), 7.01 (1H, *ddd*, *J* = 1.2, 7.8, 8.0 Hz), 6.83 (1H, *dd*, *J* = 1.2, 7.8 Hz), 5.65 (1H, *d*, *J* = 14.4 Hz), 3.92 (1H, *d*, *J* = 14.4 Hz), 2.86 (1H, *ddd*, *J* = 6.0, 7.6, 18.7 Hz), 2.65 (1H, *dt*, *J* = 6.0, 18.7 Hz), 2.32 (1H, *ddd*, *J* = 6.0, 7.6, 17.1 Hz), 2.18 (1H, *dt*, *J* = 6.0, 17.1 Hz). ^*13*^*C-NMR* (100 MHz, CDCl_3_), δ (ppm) 200.64, 170.60, 143.34, 139.98, 136.49, 130.65, 129.86, 129.21, 128.99, 128.18, 127.33, 100.11, 51.54, 38.57, 27.26. EI–MS *m*/*z* (%) 393 (M^+^, 12 %), 369 (3 %), 351 (3 %), 309 (28), 282 (5), 268 (70), 230 (4), 224 (4), 203 (9), 182 (23), 180 (63), 152 (12), 104 (7), 91 (100), 77 (13). *HRMSm*/*z* Found 393.0265, Calcd. for C_17_H_16_NO_2_I [M]^+^ 393.0226.

### Synthesis of Compound **17c**

To a solution of aldehyde **11b** (19.7 g, 50 mmol) in THF (200 mL) was added a powder of (*S*)-(−)-*tert*-butanesulfinamide (12.1 g, 100 mmol, 2.0 eq.) and Ti(OEt)_4_ (22.8 g, 100 mmol, 2.0 eq.). The resulting mixture was stirred at 60 °C under nitrogen for 12 h. The reaction mixture was then cooled to room temperature and treated with saturated NaCl aqueous solution (100 mL) for 1 h. After filtration through a short column of Celite and washed with ethyl acetate (80 mL), the combined organic phases were dried over anhydrous Na_2_SO_4_. After removal of the solvent under reduced pressure, the residue was flash chromatographed on silica gel (petroleum ether 60–90 °C:ethyl acetate = 3:1) to afford the sulfinyl imine (**17c**: 22.85 g, 92 %) as a pale yellow syrup.

$[\alpha ]_{\text{D}}^{20}$ +52 (c 0.19, CHCl_3_). *R*_f_ 0.51 (petroleum ether:ethyl acetate = 3:1). *FTIR* (KBr, thin film) cm^−1^ 3445, 2964, 1663, 1466, 1400, 1079. ^*1*^*H-NMR* (as a mixture of rotamers, 400 MHz, CDCl_3_), δ (ppm) 8.09 (1H, *brs*), 7.93 (1H, *d*, *J* = 8.0 Hz), 7.28–7.14 (6H, *m*), 7.08–7.01 (1H, *m*), 6.75 (0.5H, *d*, *J* = 7.6 Hz) [ 6.74 (0.5H, *d*, *J* = 7.6 Hz)], 5.67 (0.5H, *d*, *J* = 14.4 Hz) [5.59 (0.5H, *d*, *J* = 14.0 Hz)], 3.97 (0.5H, *d*, *J* = 14.0 Hz) [3.92 (0.5H, *d*, *J* = 14.4 Hz)], 3.00–2.87 (1H, *m*), 2.81–2.68 (1H, *m*), 2.46–2.18 (2H, *m*), 1.14 (4.5H, *s*) [1.12 (4.5H, *s*)]. ^*13*^*C-NMR* (rotamer in brackets, 100 MHz, CDCl_3_), δ (ppm) 170.51 (170.39), 168.11 (168.06), 143.54, 140.20 (140.13), 136.69 (136.60), 130.76 (130.65), 129.89, 129.45 (129.24), 129.13, 128.24, 127.45 (127.42), 100.24, 56.57 (56.53), 51.71 (51.55), 31.13 (30.99), 29.88 (29.83), 22.25 (22.22). EI–MS *m*/*z* (%) 496 (M^+^, 10 %), 395 (3), 378 (16), 309 (10), 298 (15), 268 (20), 253 (4), 224 (2), 201 (7), 182 (11), 180 (20), 152 (5), 133 (21), 119 (46), 103 (6), 91 (100), 77 (14). *HRMSm*/*z* Found 496.0663, Calcd. for C_21_H_25_N_2_O_2_SI [M]^+^ 496.0682.

### Synthesis of Compound **20c**

A solution of imine **17c** (19.8 g, 40 mmol) in dichloromethane (200 mL) was stirred at −78 °C for 10 min. To this mixture was added slowly a solution of vinyl magnesium bromide in THF (1.0 M, 60 mL, 60 mmol, 1.5 eq.). The reaction mixture was then stirred at −78 °C for 5 h before warming up to room temperature. Saturated NH_4_Cl aqueous solution (80 mL) was introduced and the resulting mixture was stirred at room temperature for 1 h. The mixture was diluted with water (150 mL) and extracted with dichloromethane (3 × 100 mL) and the combined organic phases were dried over anhydrous Na_2_SO_4_. After filtration and concentrated, the residue was chromatographed on silica gel (petroleum ether 60–90 °C:ethyl acetate = 2:1) to afford the major amide (**20c**: 16.35 g, 78 %) as a yellow syrup. Further elution with solvents (petroleum ether 60–90 °C:ethyl acetate = 1:2) provided the minor sulfinamide **20d** (1.64 g, 7.8 %) as a pale yellow oil.

#### Major: **20c**

$[\alpha ]_{\text{D}}^{20}$ +20 (c 0.50, CHCl_3_). *R*_f_ 0.56 (petroleum ether:ethyl acetate = 1:1). *FTIR* (KBr, thin film) cm^−1^ 3223, 2963, 1640, 1462, 1397, 1269, 1199, 1061. ^*1*^*H-NMR* (400 MHz, CDCl_3_), δ (ppm) 7.92 (1H, *d*, *J* = 8.0 Hz), 7.30–7.13 (6H, *m*), 7.03 (1H, *t*, *J* = 7.6 Hz), 6.68 (1H, *d*, *J* = 8.0 Hz), 5.78 (1H, *ddd*, *J* = 6.4, 10.8, 17.2 Hz), 5.65 (1H, *d*, *J* = 14.4 Hz), 5.18 (1H, *dd*, *J* = 10.8, 17.2 Hz), 5.09 (1H, *d*, *J* = 10.4 Hz), 3.91 (1H, *d*, *J* = 14.4 Hz), 3.73–3.63 (1H, *m*), 3.37 (1H, *t*, *J* = 8.4 Hz), 2.12–1.88 (4H, *m*), 1.11 (9H, *s*). ^*13*^*C-NMR* (rotamer in brackets, 100 MHz, CDCl_3_), δ (ppm) 171.89, 143.84, 140.27, 139.42, 136.90, 130.84 (130.81), 129.92, 129.45 (129.43), 129.29, 128.42, 127.60, 116.67 (116.56), 100.44, 58.62 (58.39), 55.97, 51.69, 31.07 (30.93), 30.68 (30.46), 22.65. EI–MS *m*/*z* (%) 524 (M^+^, 36 %), 467 (100), 419 (6), 404 (47), 351 (14), 309 (77), 278 (12), 238 (13), 224 (17), 182 (21), 180 (30), 146 (5), 140 (9), 110 (8), 91 (88), 77 (9). *HRMSm*/*z* Found 524.1001, Calcd. for C_23_H_29_N_2_O_2_SI [M]^+^ 524.0995.

#### Minor: **20d**

$[\alpha ]_{\text{D}}^{20}$ +41 (c 0.20, CHCl_3_). *R*_f_ 0.53 (petroleum ether:ethyl acetate = 1:1). ^*1*^*H-NMR* (300 MHz, CDCl_3_), δ (ppm) 7.37–7.13 (7H, *m*), 6.99–6.90 (2H, *m*), 5.76 (1H, *ddd*, *J* = 6.0, 9.0, 18.0 Hz), 5.17 (1H, *d*, *J* = 18.0 Hz), 5.08 (1H, *d*, *J* = 9.0 Hz), 4.87 (2H, *s*), 3.66 (1H, *dt*, *J* = 6.0, 15.0 Hz), 3.50 (1H, *d*, *J* = 9.0 Hz), 2.20–2.05 (2H, *m*), 1.96–1.84 (2H, *m*), 1.11 (9H, *s*). ^*13*^*C-NMR* (75 MHz, CDCl_3_), δ (ppm) 172.35, 142.19, 139.47, 137.42, 129.68, 128.91, 128.43, 128.14, 127.46, 116.62, 58.79, 56.07, 53.13, 30.92, 30.75, 22.65. EI–MS *m*/*z* (%) 525 (M^+^+1, 10 %), 511 (5), 467 (30), 404 (10), 341 (100), 309 (21), 278 (37), 250 (4), 238 (6), 225 (36), 183 (79), 180 (15), 140 (11), 112 (6), 110 (9), 91 (78), 77 (12). *HRMSm*/*z* Found 524.0996, Calcd. for C_23_H_29_N_2_O_2_SI [M]^+^ 524.0995.

### Synthesis of Compound **12c**

To a mixture of sodium hydride (60 % in mineral oil, 1.87 g, 46.7 mmol, 1.5 eq., freshly washed with anhydrous hexane three times under nitrogen) in anhydrous THF (50 mL) at 0 °C was added a solution of sulfonamide **20c** (16.3 g, 31.1 mmol) in THF (150 mL) via syringe. After stirring at 0 °C for 10 min, benzyl bromide (5.5 mL, 46.7 mmol, 1.5 eq.) was added. The resulting mixture was then stirred at 0 °C for 2 h, then at room temperature for 12 h under nitrogen. A powder of NH_4_Cl (2.7 g, 50.0 mmol) was added and the mixture was stirred for 10 min. After concentrated under reduced pressure, the residue was diluted with water (150 mL) and extracted with ethyl acetate (3 × 100 mL). The combined organic phases were washed with brine (50 mL) and dried over anhydrous Na_2_SO_4_. After filtration, the solvent was removed under reduced pressure and the residue was chromatographed on silica gel (petroleum ether 60–90 °C:ethyl acetate = 2:1) to afford the product (**11b**: 18.1 g, 95 %) as a pale yellow oil.

$[\alpha ]_{\text{D}}^{20}$ −37 (c 0.12, CHCl_3_). *R*_f_ 0.54 (petroleum ether:ethyl acetate = 2:1). ^*1*^*H-NMR* (as a mixture of rotamers, 400 MHz, CDCl_3_), δ (ppm) 7.90 (1H, *d*, *J* = 8.0 Hz), 7.34–7.06 (11H, *m*), 7.00 (1H, *t*, *J* = 7.6 Hz), 6.64 (0.5H, *dd*, *J* = 1.2, 7.6 Hz), 6.60 (0.5H, *dd*, *J* = 1.2, 7.6 Hz), 5.74–5.59 (2H, *m*), 5.06 (1H, *dd*, *J* = 6.8, 10.0 Hz), 4.97 (1H, *t*, *J* = 18.0 Hz), 4.41 (1H, *dd*, *J* = 10.0, 16.4 Hz), 4.02 (1H, *d*, *J* = 16.4 Hz), 3.85 (1H, *t*, *J* = 14.0 Hz), 3.59–3.38 (1H, *m*), 2.35–2.17 (1H, *m*), 2.03–1.80 (3H, *m*), 1.12 (9H, *s*). ^*13*^*C-NMR* (rotamer in brackets, 100 MHz, CDCl_3_), δ (ppm) 171.61 (171.44), 143.81, 140.21 (140.19), 138.64 (138.54), 137.54, 137.08 (137.02), 131.00, 130.87, 129.83, 129.38, 129.35, 129.21, 128.44 (128.39), 128.11 (128.08), 127.51, 127.00, 117.75 (117.69), 100.49 (100.45), 63.29 (63.08), 58.14 (58.09), 51.56, 46.27, 31.72 (31.59), 26.94 (26.88), 23.57. EI–MS *m*/*z* (%) 614 (M^+^, 4 %), 509 (7), 482 (3), 458 (1), 420 (4), 405 (5), 309 (5), 278 (4), 236 (2), 182 (8), 180 (13), 146 (7), 128 (5), 106 (12), 91 (100). *HRMSm*/*z* Found 614.1480, Calcd. for C_30_H_35_N_2_O_2_SI [M]^+^ 614.1464.

### Synthesis of Compound **13a** + **13b**

A mixture of copper iodide (CuI, 133.3 mg, 0.7 mmol, 0.1 eq.) and bromoanilide **12b** (3.78 g, 7.0 mmol) in anhydrous toluene (140 mL) was degassed and purged with argon (three times). A solution of lithium bis(trimethylsilyl)amide (1.0 M in THF, 14 mL, 14 mmol, 2.0 eq.) was added and the resulting mixture was stirred at 80 °C (oil bath) under argon for 5 h. After cooling to room temperature then to 0 °C, a solution of anhydrous *t*-BuOOH (degassed and purged with argon, ~3.0 M in toluene, 3.5 mL, 10.5 mmol, 1.5 eq.) was added. The reaction mixture was allowed to stir at 0 °C under argon for 3 h. Saturated aqueous solution of NH_4_Cl (8 mL) was added. After 30 min, the mixture was diluted with water (100 mL). The aqueous phase was extracted with ethyl acetate (3 × 60 mL). The combined organic phases were dried over anhydrous Na_2_SO_4_. After removal of the solvents, the residue was chromatographed on silica gel (petroleum ether 60–90 °C:ethyl acetate = 2:1 → 1:1 → 1:2) to afford the major product (**13a** + **13b**, as a mixture of C3–C3a diastereomers, 2.51 g, 78.1 %) as pale yellow syrup. Further elution afforded the minor product (**13c**: *meso*-isomer, 0.23 g, 7.2 %) as a pale yellow oil, which was characterized after removal of the *tert*-butylsulfinyl group (**13d**). *The anhydrous *tert*-butylhydroperoxide (*t*-BuOOH) in toluene (ca. ~3.0 M) was prepared by the following procedure: 70 % aqueous solution of *t*-BuOOH (40.6 mL, density = 0.93 g/mL) was added to toluene (46 mL) and the resulting water (ca. 10 mL) was separated and back-extracted with toluene (2 × 10 mL). The combined organic phases were then dried over anhydrous sodium sulfate. After filtration, the resulting solution was kept with 4 Å molecular sieve and could be used for this reaction without further purification.

#### **13a** + **13b**

An 83:17 mixture of diastereomers at C3–${\text{C}}{3}^{'}$ position: *R*_f_ 0.45 (petroleum ether:ethyl acetate = 1:1). *FTIR* (KBr, thin film) cm^−1^ 3396, 3057, 2957, 1705, 1609, 1460, 1360, 1176, 1072, 982, 929, 746, 703. ^*1*^*H-NMR* (400 MHz, CDCl3, major C3*S*–${\text{C}}{3}^{'}$*S*-isomer reported), δ (ppm) 7.34–7.18 (2 × 8H, *m*), 7.11–7.03 (2 × 2H, *m*), 6.81 (2 × 1H, *t*, *J* = 7.6 Hz), 6.68 (2 × 1H, *d*, *J* = 7.2 Hz), 6.49 (2 × 1H, *t*, *J* = 7.6 Hz), 6.27 (2 × 1H, *d*, *J* = 7.6 Hz), 4.92 (2 × 1H, *d*, *J* = 15.6 Hz), 4.36 (2 × 1H, *d*, *J* = 15.6 Hz), 4.21 (2 × 2H, *s*), 3.10 (2 × 1H, *ddd*, *J* = 4.8, 12.4, 12.8 Hz), 2.51 (2 × 1H, *ddd*, *J* = 3.6, 12.4, 12.8 Hz), 2.37 (2 × 1H, *ddd*, *J* = 4.0, 13.2, 13.6 Hz), 2.21–2.11 (2 × 1H, *m*), 1.17 (2 × 9H, *s*). ^*13*^*C-NMR* (100 MHz, CDCl_3_, major C3*S*–${\text{C}}{3}^{'}$*S*-isomer reported), δ (ppm) 176.69, 142.50, 137.07, 135.38, 129.02, 128.87, 128.54, 128.39, 127.75, 127.69, 127.50, 126.79, 123.58, 122.17, 108.69, 58.37, 54.28, 52.40, 44.02, 43.58, 27.78, 23.45. EI–MS *m*/*z* (%) 919 (M^+^, 1%), 862 (5), 813 (3), 756 (50), 709 (12), 662 (25), 647 (24), 629 (12), 601 (16), 575 (49), 551 (51), 537 (39), 523 (56), 404 (18), 354 (28), 313 (22), 261 (18), 236 (25), 195 (17), 118 (27), 106 (27), 91 (100), 65 (18). *HRMSm*/*z* Found 941.4114, Calcd. for C_56_H_62_N_4_O_4_NaS_2_ [M+Na]^+^ 941.4110.

#### **13c** → **13d**

*Meso*-isomer: sulfinamide (**13c**: 257 mg, 0.28 mmol) was dissolved in methanol (6 mL). To this mixture was added an aqueous solution of HCl (4 N, 0.21 mL, 0.84 mmol, 3 eq.). The resulting mixture was allowed to stir at room temperature under nitrogen for 1 h. The reaction mixture was then treated with saturated aqueous solution of sodium bicarbonate (~3 mL) and concentrated under reduced pressure. The mixture was diluted with water (10 mL) and extracted with dichloromethane (3 × 5 mL), the combined organic phases were dried over anhydrous Na_2_SO_4_. After removal of the solvent, the residue was chromatographed on silica gel (dichloromethane:methanol = 20:1) to afford the amine (**13d**) (175 mg, 88 %) as pale yellow syrup.

#### **13d**

*R*_f_ 0.35 (CH_2_Cl_2_:MeOH:Et_3_N = 20:1:0.01). *FTIR* (KBr, thin film) cm^−1^ 3475, 2921, 1704, 1614, 1454, 1362, 1102, 745. ^*1*^*H-NMR* (as a mixture of rotamers, 400 MHz, CDCl_3_), δ (ppm) 7.32–6.29 (2 × 14H, *m*), 5.00–4.89 (2 × 1H, *m*), 4.76 (2 × 0.17H, *d*, *J* = 16.0 Hz), 4.72 (2 × 0.17H, *d*, *J* = 16.0 Hz), 4.43 (2 × 0.17H, *d*, *J* = 15.2 Hz), 4.33 (2 × 0.34H, *d*, *J* = 15.2 Hz), 4.19 (2 × 0.17H, *d*, *J* = 15.2 Hz), 3.62–3.45 (2 × 2H, *m*), 3.24–3.12 (2 × 0.66H, *m*), 2.63–2.10 (2 × 3.34H, *m*), 1.18 (2 × 1H, *brs*). ^*13*^*C-NMR* (as a mixture of rotamers, 100 MHz, CDCl_3_), δ (ppm) 178.20 (177.90), 143.07 (142.89), 140.82 (140.61), 140.33 (140.21), 136.12 (136.03), 135.78 (135.62), 131.83 (131.45), 128.87 (128.84), 128.65 (128.46), 128.31 (128.19), 128.15 (128.02), 127.91, 127.86 (127.72), 127.52 (127.45), 127.32, 127.00, 126.73, 124.85 (124.80), 123.92, 122.62 (122.56), 121.71 (121.59), 120.06 (119.86), 109.40, 108.50 (108.31), 107.32 (107.01), 55.19, 53.97 (53.64), 45.44, 44.19 (44.02), 29.10 (28.96). *HRMSm*/*z* Found 711.3658, Calcd. for C_48_H_47_N_4_O_2_ [M+H]^+^ 711.3699.

### Synthesis of Compound **19**

A mixture of tris(dibenzylideneacetone)dipalladium [Pd_2_(dba)_3_, FW 915.72, 46.0 mg, 0.05 mmol, 0.025 eq.] and triphenylphosphine (FW 262.29, 52 mg, 0.2 mmol, 0.1 eq.) and bromoanilide **12b** (1.08 g, 2.0 mmol) in anhydrous toluene (40 mL) was degassed and purged with argon (three times). A solution of lithium bis(trimethylsilyl)amide (1.0 M in THF, 4 mL, 4 mmol, 2.0 eq.) was added and the resulting mixture was stirred at 80 °C (oil bath) under argon for 6 h. After cooling to room temperature then 0 °C, a solution of anhydrous *t*-BuOOH (~3.0 M in toluene, 1.0 mL, 3 mmol, 1.5 eq.) was added and the reaction mixture was stirred at 0 °C (ca. 5 h). Saturated aqueous solution of NH_4_Cl (0.5 mL) was added. After 10 min, anhydrous sodium sulfate (ca. 2–3 g) was added. The resulting mixture was then filtered and washed with ethyl acetate (3 × 5 mL). After removal of the solvents, the residue was chromatographed on silica gel (petroleum ether 60–90 °C:ethyl acetate = 2:1 → 1:1) to afford the minor product (**19b**, 276 mg, 28 %) as white plates. Further elution afforded the major product (**19**, 489 mg, 51 %) as a yellow solid.

#### **19**

m.p. 125–127 °C. *R*_f_ 0.54 (petroleum ether:ethyl acetate = 1:1). *FTIR* (KBr, thin film) cm^−1^ 3321, 3057, 2958, 2866, 1722, 1611, 1460, 1359, 1273, 1174, 1068, 928, 743, 704, 633, 596, 464. ^*1*^*H-NMR* (as a mixture of rotamers, 400 MHz, CDCl_3_), δ (ppm) 7.30–7.11 (12H, *m*), 7.08 (0.5H, *t*, *J* = 7.6 Hz), 7.07 (0.5H, *t*, *J* = 7.6 Hz), 6.92 (0.5H, *t*, *J* = 7.6 Hz), 6.91 (0.5H, *t*, *J* = 7.6 Hz), 6.61 (0.5H, *d*, *J* = 7.6 Hz), 6.60 (0.5H, *d*, *J* = 7.6 Hz), 5.07 (0.5H, *s*), 5.04 (0.5H, *s*), 4.86 (0.5H, *d*, *J* = 15.6 Hz), 4.83 (0.5H, *d*, *J* = 15.6 Hz), 4.61 (0.5H, *d*, *J* = 15.6 Hz), 4.59 (0.5H, *d*, *J* = 15.6 Hz), 4.19 (0.5H, *d*, *J* = 15.2 Hz), 4.17 (0.5H, *d*, *J* = 15.2 Hz), 4.02 (0.5H, *d*, *J* = 15.2 Hz), 4.01 (0.5H, *d*, *J* = 15.2 Hz), 3.10–2.95 (1H, *m*), 2.93–2.70 (1H, *m*), 2.38–2.18 (2H, *m*), 1.10 (4.5H, *s*), 1.09 (4.5H, *s*). ^*13*^*C-NMR* (rotamer in brackets, 100 MHz, CDCl_3_), δ (ppm) 177.54, 141.86, 136.62 (136.56), 135.29, 129.86 (129.80), 129.21, 128.60, 128.36 (128.30), 127.41 (127.20), 127.03 (127.00), 123.73 (123.68), 122.88, 109.24, 74.75 (74.71), 57.97, 51.24, 43.46, 42.72, 36.55 (36.43), 23.09. *HRMSm*/*z* Found 499.2028, Calcd. for C_28_H_32_N_2_NaO_3_S [M+Na]^+^ 499.2031.

#### **19b**

m.p. 113–115 °C. *R*_f_ 0.52 (petroleum ether:ethyl acetate = 2:1). *FTIR* (KBr, thin film) cm^−1^ 3415, 3059, 2980, 2930, 1718, 1612, 1462, 1360, 1310, 1172, 1124, 996, 934, 743, 702, 645, 521. ^*1*^*H-NMR* (as a mixture of rotamers, 400 MHz, CDCl_3_), δ (ppm) 7.39–7.10 (12H, *m*), 6.95 (1H, *t*, *J* = 7.6 Hz), 6.63 (1H, *t*, *J* = 7.6 Hz), 4.90 (0.4H, *d*, *J* = 16.0 Hz), 4.89 (0.6H, *d*, *J* = 16.0 Hz), 4.62 (0.4H, *d*, *J* = 15.6 Hz), 4.61 (0.6H, *d*, *J* = 15.6 Hz), 4.51–4.37 (1H, *brs*), 4.05 (0.4H, *s*), 3.99 (0.6H, *s*), 3.32–3.11 (2H, *m*), 2.27–2.15 (2H, *m*), 1.40 (4.5H, *s*), 1.39 (4.5H, *s*). ^*13*^*C-NMR* (100 MHz, CDCl_3_), δ (ppm) 177.44, 141.95, 136.04, 135.31, 129.61, 129.37, 128.82, 128.57, 127.79, 127.66, 127.15, 123.77, 123.18, 109.52, 74.81, 61.62, 52.20, 43.69, 42.52, 36.79, 24.79. *HRMSm*/*z* Found 515.1974, Calcd. for C_28_H_32_N_2_NaO_4_S [M+Na]^+^ 515.1975.

### Synthesis of Compound **21a**

To a mixture of copper iodide (CuI, 190 mg, 1.0 mmol, 0.1 eq.) and *o*-iodoanilide **12c** (6.14 g, 10.0 mmol) in anhydrous toluene (200 mL) was added a solution of lithium bis(trimethylsilyl)amide (1.0 M in THF, 20 mL, 20 mmol, 2.0 eq.). The resulting mixture was degassed and purged with argon (three times). The reaction mixture was then allowed to stir at 60 °C (oil bath) under argon for 5 h. After cooling to room temperature then to 0 °C, a solution of anhydrous *t*-BuOOH (degassed and purged with argon, ~3.0 M in toluene, 5.0 mL, 15 mmol, 1.5 eq.) was added. The reaction mixture was then stirred at 0 °C under argon for 3 h. Saturated aqueous solution of Na_2_S_2_O_3_ (10 mL) was added followed by saturated aqueous solution of NH_4_Cl (10 mL). After 30 min, the resulting mixture was diluted with water (200 mL) and extracted with ethyl acetate (3 × 100 mL). The combined organic phases were dried over anhydrous Na_2_SO_4_. After removal of the solvents, the residue was chromatographed on silica gel (petroleum ether 60–90 °C:ethyl acetate = 2:1 → 1:1 → 1:2) to afford the major product (**21a**, 2.42 g, 50 %) as a pale yellow syrup. Further elution afforded the *meso*-isomer (**21c**, characterized after removal of *tert*-butylsulfinyl group, 0.205 g, 4.2 %), followed by minor product (**21b**, characterized after removal of the *tert*-butylsulfinyl group, 0.40 g, 8.2 %) as a pale yellow oil.

#### **21a** (Major Isomer)

$[\alpha ]_{\text{D}}^{20}$ −211 (c 0.14, CHCl_3_). *R*_f_ 0.55 (petroleum ether:ethyl acetate = 1:1). *FTIR* (KBr, thin film) cm^−1^ 3440, 2960, 1702, 1609, 1465, 1361, 1177, 1071, 924, 745, 701. ^*1*^*H-NMR* (400 MHz, CDCl_3_), δ (ppm) 7.45 (2 × 2H, *d*, *J* = 8.0 Hz), 7.42 (2 × 2H, *d*, *J* = 8.0 Hz), 7.32–7.25 (2 × 1H, *m*), 7.21–7.11 (2 × 3H, *m*), 7.01 (2 × 2H, *d*, *J* = 7.2 Hz), 6.85 (2 × 1H, *d*, *J* = 7.6 Hz), 6.79 (2 × 1H, *t*, *J* = 7.6 Hz), 6.52 (2 × 1H, *t*, *J* = 7.6 Hz), 6.21 (2 × 1H, *d*, *J* = 8.0 Hz), 5.21 (2 × 1H, *ddd*, *J* = 9.6, 10.4, 16.8 Hz), 4.80 (2 × 1H, *d*, *J* = 15.2 Hz), 4.58 (2 × 1H, *d*, *J* = 16.8 Hz), 4.14 (2 × 1H, *d*, *J* = 15.2 Hz), 4.13 (2 × 1H, *d*, *J* = 10.4 Hz), 3.90 (2 × 1H, *d*, *J* = 17.2 Hz), 3.84 (2 × 1H, *d*, *J* = 17.2 Hz), 3.29 (2 × 1H, *dd*, *J* = 11.2, 13.0 Hz), 3.02–2.89 (2 × 2H, *m*), 1.18 (2 × 9H, *s*). ^*13*^*C-NMR* (100 MHz, CDCl_3_), δ (ppm) 176.36, 142.92, 138.63, 137.51, 135.60, 128.65, 128.57, 128.24, 128.11, 127.93, 127.59, 127.29, 126.97, 125.34, 121.21, 115.89, 108.09, 62.93, 58.10, 54.96, 45.28, 43.83, 33.08, 23.35. *HRMSm*/*z* Found 971.4619, Calcd. for C_60_H_67_N_4_O_4_S_2_ [M+H]^+^ 971.4604.

#### **21b** → **21d** (Minor-Isomer)

Sulfinamide (**21b**: 400 mg, 0.41 mmol) was dissolved in methanol (6 mL). To this mixture was added an aqueous solution of HCl (4 N, 0.31 mL, 1.23 mmol, 3 eq.). The resulting mixture was allowed to stir at room temperature under nitrogen for 1 h. The reaction mixture was then treated with saturated aqueous solution of sodium bicarbonate (~8 mL) and concentrated under reduced pressure. The mixture was diluted with water (10 mL) and extracted with dichloromethane (3 × 10 mL), the combined organic phases were dried over anhydrous Na_2_SO_4_. After removal of the solvent, the residue was chromatographed on silica gel (petroleum ether 60–90 °C:ethyl acetate = 2:1) to afford the diamine (**21d**) (290 mg, 92 %) as yellow oil.

#### **21d** (Minor Isomer)

$[\alpha ]_{\text{D}}^{20}$ −153 (c 0.18, CHCl_3_). *R*_f_ 0.42 (petroleum ether:ethyl acetate = 1:1). *FTIR* (KBr, thin film) cm^−1^ 3420, 3061, 2925, 2843, 2357, 1712, 1608, 1482, 1460, 1358, 1175, 1110, 991, 921, 743, 701. ^*1*^*H-NMR* (as a mixture of rotamers, 400 MHz, CDCl_3_), δ (ppm) 7.50–6.31 (2 × 14H, *m*), 5.62–5.46 (2 × 1H, *m*), 5.10–4.72 (2 × 2.35H, *m*), 4.56 (2 × 0.33H, *d*, *J* = 16.0 Hz), 4.44 (2 × 0.33H, *d*, *J* = 15.6 Hz), 4.33 (2 × 0.67H, *dd*, *J* = 11.6, 15.6 Hz), 4.03 (2 × 0.33H, *d*, *J* = 15.6 Hz), 3.66 (2 × 0.33H, *d*, *J* = 13.2 Hz), 3.55 (2 × 0.33H, *d*, *J* = 13.2 Hz), 3.49 (2 × 0.33H, *d*, *J* = 12.8 Hz), 3.43–3.31 (2 × 0.67H, *m*), 3.14 (2 × 0.33H, *d*, *J* = 13.2 Hz), 3.02 (2 × 0.33H, *d*, *J* = 13.2 Hz), 2.91–2.80 (2 × 0.33H, *m*), 2.77 (2 × 0.33H, *d*, *J* = 12.8 Hz), 2.67–2.50 (2 × 1H, *m*), 2.50–2.38 (2 × 0.67H, *m*), 2.22–2.14 (2 × 0.33H, *m*), 0.97 (2 × 1H, *brs*). ^*13*^*C-NMR* (rotamer in brackets, 100 MHz, CDCl_3_), δ (ppm) 179.54 (179.21), 143.82 (143.70), 141.22, 140.53 (140.42), 140.35, 140.14 (140.09), 136.39, 136.06 (135.86), 130.91, 128.87 (128.61), 128.49 (128.45), 128.36, 128.21 (128.13), 128.04, 127.86 (127.83), 127.52, 127.39 (127.34), 127.24, 126.76 (126.59), 126.42 (126.35), 125.26, 124.76, 121.94 (121.29), 119.45, 115.73, 115.44 (115.21), 109.33, 108.16 (107.18), 58.37, 58.09 (57.68), 54.81, 54.74 (54.59), 50.81, 50.75 (50.42), 44.57 (44.42), 44.11 (44.06), 34.89. *HRMSm*/*z* Found 763.4008, Calcd. for C_52_H_51_N_4_O_2_ [M+H]^+^: 763.4012.

#### **21c** → **21e** (*Meso*-isomer)

Sulfinamide (**21c**: 205 mg, 0.21 mmol) was dissolved in methanol (6 mL). To this mixture was added an aqueous solution of HCl (4 N, 0.16 mL, 0.63 mmol, 3 eq.). The resulting mixture was allowed to stir at room temperature under nitrogen for 1 h. The reaction mixture was then treated with saturated aqueous solution of sodium bicarbonate (~4 mL) and concentrated under reduced pressure. The mixture was diluted with water (10 mL) and extracted with dichloromethane (3 × 5 mL), the combined organic phases were dried over anhydrous Na_2_SO_4_. After removal of the solvent, the residue was chromatographed on silica gel (petroleum ether 60–90 °C:ethyl acetate = 1:2) to afford the diamine (**21e**) (145 mg, 91 %) as yellow syrup.

#### **21e** (*Meso*-isomer)

$[\alpha ]_{\text{D}}^{20}$ −29 (c 0.18, CHCl_3_). *R*_f_ 0.41 (petroleum ether:ethyl acetate = 1:3). *FTIR* (KBr, thin film) cm^−1^ 3311, 3062, 2975, 2924, 2846, 1704, 1613, 1492, 1455, 1354, 1257, 1182, 1113, 995, 921, 742, 703, 634. ^*1*^*H-NMR* (as a mixture of rotamers, 400 MHz, CDCl_3_), δ (ppm) 7.23–7.01 (2 × 9H, *m*), 6.96–6.60 (2 × 4H, *m*), 6.48 (2 × 0.5H, *d*, *J* = 8.0 Hz), 6.45 (2 × 0.5H, *d*, *J* = 8.0 Hz), 5.56–5.35 (2 × 1H, *m*), 4.98 (2 × 0.5H, *d*, *J* = 16.0 Hz), 4.87 (2 × 0.5H, *d*, *J* = 10.0 Hz), 4.87 (2 × 0.5H, *d*, *J* = 10.0 Hz), 4.76 (2 × 0.5H, *d*, *J* = 15.6 Hz), 4.68 (2 × 0.5H, *d*, *J* = 16.8 Hz), 4.54 (2 × 0.5H, *d*, *J* = 16.8 Hz), 4.32 (2 × 0.5H, *brs*), 3.88 (2 × 0.5H, *brs*), 3.55 (2 × 0.5H, *d*, *J* = 13.2 Hz), 3.49 (2 × 0.5H, *d*, *J* = 13.2 Hz), 3.13 (2 × 0.5H, *d*, *J* = 13.2 Hz), 3.01 (2 × 0.5H, *d*, *J* = 13.2 Hz), 3.00 (2 × 0.5H, *brs*), 2.92 (2 × 1H, *brs*), 2.68–2.50 (2 × 2H, *m*), 0.97 (2 × 1H, *brs*). ^*13*^*C-NMR* (rotamer in brackets, 100 MHz, CDCl_3_), δ (ppm) 178.05 (176.96), 145.05 (144.20), 140.49 (140.39), 140.31 (140.19), 136.17 (135.85), 128.56 (128.44), 128.22 (128.00), 127.55, 127.25 (127.21), 126.95, 126.69 (126.56), 125.10, 124.54, 121.74 (121.45), 115.99 (115.44), 109.41 (109.30), 58.75 (58.56), 55.83 (55.34), 50.92 (50.85), 44.37, 37.69 (36.62). *HRMSm*/*z* Found 763.4003, Calcd. for C_52_H_51_N_4_O_2_ [M+H]^+^ 763.4012.

### Synthesis of Compound **22**

A solution of **21a** (485 mg, 0.5 mmol) in dichloromethane and methanol (20 mL, 1:1 mixture) was cooled to −78 °C (dry ice–acetone bath). Ozone was then passed through the solution for 10 min. The reaction progress was monitored by TLC. Sodium borohydride (189 mg, 5 mmol, 10 eq.) was added. The reaction mixture was then gradually warmed up to room temperature under argon at stirration overnight. Saturated aqueous solution of NH_4_Cl (10 mL) was added. The resulting mixture was diluted with water (20 mL) and extracted with dichloromethane (3 × 20 mL). The combined organic phases were dried over anhydrous Na_2_SO_4_. After removal of the solvents, the residue was chromatographed on silica gel (petroleum ether 60–90 °C:ethyl acetate = 1:2) to afford the product (**22**, 401 mg, 82 %) as a pale yellow syrup.

$[\alpha ]_{\text{D}}^{20}$ −332 (c 0.14, CHCl_3_). *R*_f_ 0.45 (petroleum ether:ethyl acetate = 1:2). *FTIR* (KBr, thin film) cm^−1^ 3730, 3436, 2921, 2351, 1703, 1609, 1460, 1364, 1174, 1046, 745. ^*1*^*H-NMR* (400 MHz, CDCl_3_), δ (ppm) 7.51 (2 × 2H, *d*, *J* = 7.6 Hz), 7.35 (2 × 2H, *t*, *J* = 7.6 Hz), 7.25–7.16 (2 × 4H, *m*), 7.04 (2 × 2H, *dd*, *J* = 1.6, 7.6 Hz), 6.92 (2 × 1H, *d*, *J* = 6.8 Hz), 6.88 (2 × 1H, *d*, *J* = 8.0 Hz), 6.63 (2 × 1H, *t*, *J* = 7.6 Hz), 6.32 (2 × 1H, *d*, *J* = 8.0 Hz), 4.84 (2 × 1H, *d*, *J* = 15.6 Hz), 4.52 (2 × 1H, *d*, *J* = 16.8 Hz), 4.35 (2 × 1H, *d*, *J* = 15.6 Hz), 3.90 (2 × 1H, *d*, *J* = 16.8 Hz), 3.12 (2 × 1H, *ddd*, *J* = 3.6, 11.0, 12.8 Hz), 3.03 (2 × 1H, *dd*, *J* = 9.2, 14.0 Hz), 2.92 (2 × 1H, *d*, *J* = 14.0 Hz), 2.59 (2 × 1H, *ddd*, *J* = 3.2, 9.2, 12.8 Hz), 2.05–1.97 (2 × 1H, *m*), 1.20 (2 × 9H, *s*). ^*13*^*C-NMR* (100 MHz, CDCl_3_), δ (ppm) 176.31, 142.80, 138.33, 135.12, 129.09, 128.92, 128.78, 128.38, 127.78, 127.63, 127.35, 124.16, 122.28, 109.11, 63.92, 63.28, 58.37, 55.42, 45.36, 44.02, 30.25, 23.75. *HRMSm*/*z* Found 979.4513, Calcd. for C_58_H_67_N_4_O_6_S_2_ [M+H]^+^ 979.4502.

### Synthesis of Compound **23**

To a mixture of diol **22** (49 mg, 0.05 mmol), trimethylamine (20 mg, 0.027 mL, 0.2 mmol) and DMAP (3 mg, 0.025 mmol) in dichloromethane (5 mL) was added 4-fluorobenzene-1-sulfonyl chloride (39 mg, 0.2 mmol, 4.0 eq.). The resulting mixture was then allowed to stir at room temperature for 6 h. A solution of saturated aqueous solution of NaHCO_3_ (2 mL) was added and diluted with water (5 mL). The mixture was extracted with dichloromethane (3 × 4 mL). The combined organic phases were dried over anhydrous Na_2_SO_4_. After removal of the solvents, the residue was chromatographed on silica gel (petroleum ether 60–90 °C:ethyl acetate = 1:1) to afford the product (**23**, 60 mg, 92 %) as a plate.

m.p. 127–129 °C. $[\alpha ]_{\text{D}}^{20}$ −160 (c 0.72, CHCl_3_). *R*_f_ 0.55 (petroleum ether:ethyl acetate = 1:1). *FTIR* (KBr, thin film) cm^−1^ 3730, 3456, 2921, 2351, 1703, 1459, 1368, 1016, 752. ^*1*^*H-NMR* (400 MHz, CDCl_3_), δ (ppm) 7.46 (2 × 2H, *d*, *J* = 7.6 Hz), 7.42–7.31 (2 × 4H, *m*), 7.28–7.20 (2 × 2H, *m*), 7.16 (2 × 2H, *t*, *J* = 7.6 Hz), 6.96–6.76 (2 × 6H, *m*), 6.52 (2 × 1H, *m*), 6.23 (2 × 1H, *d*, *J* = 7.2 Hz), 4.64 (2 × 1H, *d*, *J* = 15.6 Hz), 4.63 (2 × 1H, *d*, *J* = 17.2 Hz), 4.13 (2 × 1H, *d*, *J* = 15.6 Hz), 3.83 (2 × 1H, *d*, *J* = 17.2 Hz), 3.82 (2 × 1H, *d*, *J* = 10.8 Hz), 3.20–3.01 (2 × 2H, *m*), 2.84 (2 × 1H, *dd*, *J* = 2.8, 10.8 Hz), 2.71–2.61 (2 × 1H, *m*), 1.27 (2 × 9H, *s*). ^*13*^*C-NMR* (100 MHz, CDCl_3_), δ (ppm) 176.36, 166.80, 164.25, 142.70, 137.16, 135.09, 131.64, 130.51, 130.41, 129.61, 129.14, 128.95, 128.70, 128.61, 128.33, 127.75, 127.53, 127.32, 126.55, 124.09, 122.18, 116.52, 116.29, 109.37, 70.58, 58.80, 58.48, 55.14, 46.39, 44.03, 30.62, 23.60. *HRMSm*/*z* Found 1317.3994, Calcd. for C_70_H_72_N_4_O_10_S_4_F_2_Na [M+Na]^+^ 1317.3997.

### Synthesis of Compound **13a′** + **13b′**

To a mixture of copper iodide (CuI, 190.4 mg, 1.0 mmol, 0.1 eq.) and bromoanilide **12b′** (5.41 g, 10 mmol) in anhydrous toluene (200 mL) was added a solution of lithium bis(trimethylsilyl)amide (1.0 M in THF, 20 mL, 20 mmol, 2.0 eq.). The resulting mixture was degassed and purged with argon (three times). After which, the reaction mixture was stirred at 80 °C (oil bath) under argon for 5 h. After cooling to room temperature then to 0 °C, a solution of anhydrous *t*-BuOOH (~3 M in toluene, 5.0 mL, 15 mmol, 1.5 eq.) was added. The reaction mixture was allowed to stir at 0 °C under argon for 3 h. Saturated aqueous solution of NH_4_Cl (10 mL) was added. After 30 min, the mixture was diluted with water (200 mL). The resulting mixture was then extracted with ethyl acetate (3 × 100 mL). The combined organic phases were dried over anhydrous Na_2_SO_4_. After removal of the solvents, the residue was chromatographed on silica gel (petroleum ether 60–90 °C:ethyl acetate = 1:1 → 1:2 → 1:3) to afford the major product (**13a′** + **13b′**, 3.59 g, 78 %) as a pale yellow syrup. Further elution afforded the minor product (**13c′**, 0.32 g, 7 %) as a pale yellow oil, which was characterized after removal of *tert*-butylsulfinyl group (see **13d**).

#### **13a′** + **13b′**

A mixture of diastereomers at C3–${\text{C}}{3}^{'}$ position (84:16): *R*_f_ 0.45 (petroleum ether:ethyl acetate = 1:1). *FTIR* (KBr, thin film) cm^−1^ 3429, 2969, 2352, 1702, 1612, 1456, 1365, 1052. ^*1*^*H-NMR* (400 MHz, CDCl_3_, major C3*R–*${\text{C}}{3}^{'}$*R*-isomer reported), δ (ppm) 7.37–7.19 (2 × 8H, *m*), 7.10–7.03 (2 × 2H, *m*), 6.81 (2 × 1H, *t*, *J* = 7.6 Hz), 6.68 (2 × 1H, *d*, *J* = 7.6 Hz), 6.49 (2 × 1H, *t*, *J* = 7.6 Hz), 6.26 (2 × 1H, *d*, *J* = 8.0 Hz), 4.91 (2 × 1H, *d*, *J* = 15.6 Hz), 4.36 (2 × 1H, *d*, *J* = 15.6 Hz), 4.21 (2 × 2H, *s*), 3.11 (2 × 1H, *ddd*, *J* = 4.4, 12.4, 12.8 Hz), 2.48 (2 × 1H, *ddd*, *J* = 3.6, 12.4, 12.8 Hz), 2.37 (2 × 1H, *ddd*, *J* = 3.6, 13.2, 13.6 Hz), 2.22–2.10 (2 × 1H, *m*), 1.17 (2 × 9H, *s*). ^*13*^*C-NMR* (100 MHz, CDCl_3_, major C3*R–*${\text{C}}{3}^{'}$*R*-isomer reported), δ (ppm) 176.63, 142.44, 137.01, 135.32, 128.96, 128.81, 128.49, 128.34, 127.70, 127.64, 127.45, 126.80, 126.72, 123.52, 122.11, 108.63, 58.31, 54.22, 52.40, 43.96, 43.51, 27.70, 23.40. EI–MS *m*/*z* (%) 919 (M^+^, 1 %), 918 (1 %), 706 (2), 588 (3), 575 (1), 354 (3), 249 (2), 236 (4), 223 (4), 132 (10), 118 (19), 106 (7), 91 (100), 65 (5). *HRMSm*/*z* Found 918.4224, Calcd. for C_56_H_62_N_4_O_4_S_2_ (M)^+^ 918.4213; Found 919.4307, Calcd. for C_56_H_63_N_4_O_4_S_2_ [M+H]^+^ 919.4291.

## Synthesis of Compound **24**

Sulfinamide (**13a′** + **13b′**: 3.59 g, 3.9 mmol) was dissolved in methanol (60 mL). To this mixture was added an aqueous solution of HCl (4 N, 2.9 mL, 11.7 mmol, 3 eq.). The resulting mixture was allowed to stir at room temperature under nitrogen for 1 h. The reaction mixture was then treated with saturated aqueous solution of sodium bicarbonate (~50 mL) and concentrated under reduced pressure. The mixture was diluted with water (100 mL) and extracted with dichloromethane (3 × 50 mL), the combined organic phases were dried over anhydrous Na_2_SO_4_. After removal of the solvent, the residue was chromatographed on silica gel (dichloromethane:methanol = 20:1) to afford the amine (**24**) (2.63 g, 95 %) as white foam. The diamine was dissolved in methanol (20 mL) and HCl (2 N, 5.6 mL, 11.1 mmol, 3.0 eq.) was added. This solution was allowed to crystallize at room temperature. The needle-like crystals were collected and subjected to HPLC analysis (a 1:1 mixture of C3*R–*${\text{C}}{3}^{'}$*R* and C3*S–*${\text{C}}{3}^{'}$*S* enantiomers). The mother liquid was then treated with saturated aqueous solution of sodium bicarbonate to pH 8, and extracted with dichloromethane (3). The combined organic phases were dried over anhydrous Na_2_SO_4_. After removal of the solvents, the enantiomeric pure product **24** was obtained (1.60 g, 61 %) as a pale yellow syrup.

$[\alpha ]_{\text{D}}^{20}$ +171 (c 0.12, MeOH). *ee* = 99.1 %. *R*_f_ 0.40 (CH_2_Cl_2_:MeOH:Et_3_N = 20:1:0.01). *FTIR* (KBr, thin film) cm^−1^ 3426, 2967, 1701, 1611, 1456, 1365, 1174, 1047, 746, 700. ^*1*^*H-NMR* (400 MHz, CDCl_3_), δ (ppm) 7.32–7.16 (2 × 9H, *m*), 7.10 (2 × 2H, *d*, *J* = 7.2 Hz), 7.04 (2 × 1H, *d*, *J* = 7.2 Hz), 6.97 (2 × 1H, *t*, *J* = 7.6 Hz), 6.71 (2 × 1H, *t*, *J* = 7.6 Hz), 6.39 (2 × 1H, *d*, *J* = 7.6 Hz), 5.03 (2 × 1H, *d*, *J* = 15.6 Hz), 4.45 (2 × 1H, *d*, *J* = 15.6 Hz), 3.60 (2 × 1H, *d*, *J* = 13.6 Hz), 3.53 (2 × 1H, *d*, *J* = 13.6 Hz), 3.26 (2 × 1H, *ddd*, *J* = 5.6, 6.0, 13.2 Hz), 2.64 (2 × 1H, *ddd*, *J* = 7.6, 8.0, 13.2 Hz), 2.27–2.17 (2 × 2H, *m*). ^*13*^*C-NMR* (100 MHz, CDCl_3_), δ (ppm) 178.03, 143.09, 140.31, 135.85, 128.68, 128.32, 128.06, 128.01, 127.85, 127.58, 126.74, 124.12, 121.76, 108.52, 55.14, 53.65, 45.41, 44.12, 29.22. EI–MS *m*/*z* (%) 710 (M^+^, 2 %), 577 (1), 356 (4), 344 (2), 262 (3), 236 (4), 223 (4), 134 (8), 118 (12), 106 (21), 91 (100). *HRMSm*/*z* Found 710.3622, Calcd. for C_48_H_46_N_4_O_2_ [M]^+^ 710.3621.

### Synthesis of Compound **25**

To diamine **24** (0.82 g, 1.15 mmol) in acetonitrile (10 mL) was added a solution of formaldehyde (37 % aqueous solution, 0.44 mL, 5.75 mmol, 5.0 eq.). Sodium triacetoxyborohydride (1.22 g, 5.75 mmol, 5.0 eq.) was added and the resulting mixture was stirred at room temperature under argon for 2 h. A solution of methanol in dichloromethane (MeOH:CH_2_Cl_2_ = 5:95, 10 mL) saturated with ammonia was added. The mixture was then allowed to stir at room temperature for 5 min. After removal of the solvent under reduced pressure, the residue was chromatographed on silica gel (dichloromethane:MeOH:NH_3_–H_2_O = 300:10:1) to afford the product (**25**, 0.82 g, 96 % yield) as a pale yellow syrup.

$[\alpha ]_{\text{D}}^{20}$ +171 (c 0.10, MeOH). *R*_f_ 0.45 (CH_2_Cl_2_:MeOH = 30:1). *FTIR* (KBr, thin film) cm^−1^ 3436, 2930, 2789, 1702, 1608, 1462, 1361, 1177, 1036, 744, 700. ^*1*^*H-NMR* (300 MHz, CDCl_3_), δ (ppm) 7.18–7.01 (2 × 10H, *m*), 6.89 (2 × 1H, *d*, *J* = 7.5 Hz), 6.79 (2 × 1H, *t*, *J* = 7.5 Hz), 6.55 (2 × 1H, *t*, *J* = 7.5 Hz), 6.25 (2 × 1H, *d*, *J* = 7.5 Hz), 4.92 (2 × 1H, *d*, *J* = 15.0 Hz), 4.32 (2 × 1H, *d*, *J* = 15.0 Hz), 3.26 (2 × 1H, *d*, *J* = 12.9 Hz), 3.15 (2 × 1H, *d*, *J* = 12.9 Hz), 3.18–3.07 (2 × 1H, *m*), 2.67–2.53 (2 × 1H, *m*), 1.94 (2 × 3H, *s*), 1.98–1.86 (2 × 1H, *m*), 1.79–1.67 (2 × 1H, *m*). ^*13*^*C-NMR* (75 MHz, CDCl_3_), δ (ppm) 177.53, 142.97, 138.34, 135.84, 129.27, 128.62, 128.05, 128.01, 127.86, 127.56, 126.83, 124.02, 121.75, 108.35, 61.81, 55.00, 53.04, 43.99, 41.80, 26.11. EI–MS *m*/*z* (%) 738 (M^+^, 9 %), 647 (21), 591 (43), 444 (4), 370 (23), 293 (2), 277 (13), 235 (12), 148 (17), 134 (93), 120 (25), 91 (100), 65 (10). *HRMSm*/*z* Found 738.3931, Calcd. for C_50_H_50_N_4_O_2_ [M]^+^ 738.3934.

### Synthesis of Compound **26**

Diamine **25** (369 mg, 0.5 mmol) in anhydrous 1,2-dichloroethane (15 mL) was stirred with a solution of α-chloroethyl chloroformate (ACE-Cl, 0.54 mL, 5 mmol, 10.0 eq.) at 0 °C for 2 h, then at room temperature for 1 h. After which, the reaction mixture was allowed to stir at 80 °C (oil bath) for 12 h. After removal of the solvents, the residue was diluted with methanol (15 mL) and stirred at 70 °C (oil bath) for 3 h. The resulting mixture was concentrated under reduced pressure and diluted with dichloromethane (5 mL), ice (~10 g) and saturated aqueous solution of NaHCO_3_ (10 mL). The mixture was then extracted with dichloromethane (3 × 15 mL), and the combined organic phases was dried over anhydrous sodium sulfate. After filtration and removal of the solvent under reduced pressure, the residue was chromatographed on silica gel (dichloromethane:MeOH:NH_3_–H_2_O = 100:100:1) to afford the product (**26**, 265 mg, 95 % yield) as a pale yellowish syrup.

$[\alpha ]_{\text{D}}^{20}$ +203 (c 0.10, MeOH). *R*_f_ 0.44 (CH_2_Cl_2_:MeOH = 1:1). *FTIR* (KBr, thin film) cm^−1^ 3431, 2970, 2352, 1628, 1397, 1089. ^*1*^*H-NMR* (400 MHz, CDCl_3_), δ (ppm) 7.32–7.11 (2 × 5H, *m*), 7.03 (2 × 1H, *d*, *J* = 7.2 Hz), 6.92 (2 × 1H, *dt*, *J* = 0.8, 7.6 Hz), 6.70 (2 × 1H, *t*, *J* = 7.6 Hz), 6.35 (2 × 1H, *d*, *J* = 7.6 Hz), 5.10 (2 × 1H, *d*, *J* = 15.6 Hz), 4.40 (2 × 1H, *d*, *J* = 15.6 Hz), 3.25–3.13 (2 × 1H, *m*), 2.58–2.49 (2 × 1H, *m*), 2.21 (2 × 3H, *s*), 2.12-2.03 (2 × 3H, *m*). ^*13*^*C-NMR* (100 MHz, CDCl_3_), δ (ppm) 177.73, 142.98, 135.88, 128.74, 128.20, 128.00, 127.86, 127.66, 124.02, 121.84, 108.54, 55.04, 47.81, 44.05, 36.12, 29.00. EI–MS *m*/*z* (%) 558 (M^+^, 21 %), 526 (5), 501 (100), 470 (12), 444 (43), 280 (52), 248 (7), 236 (38), 223 (28), 187 (7), 158 (13), 91 (56). *HRMSm*/*z* Found 558.2989, Calcd. for C_36_H_38_N_4_O_2_ [M]^+^ 558.2995.

### Synthesis of Compound **27**

Diamine **26** (558 mg, 1.0 mmol) in THF (25 mL) was degassed and purged with argon (three times). A solution of diisobutylaluminium hydride (DIBAL-H, 1.1 M in THF, 10 mL, 10 mmol, 10.0 eq.) was added and the resulting mixture was stirred at 0 °C for 1 h, then at room temperature for 2 h and finally at 80 °C (oil bath) under argon for 15 h. After cooling to room temperature, a saturated aqueous solution of potassium sodium tartrate (10 mL) was added and the resulting mixture was stirred at room temperature for 2 h. The mixture was diluted with water (30 mL) and extracted with ethyl acetate (3 × 30 mL). The combined organic phases were dried over anhydrous sodium sulfate. After filtration and removal of the solvent under reduced pressure, the residue was chromatographed on silica gel (petroleum ether 60–90 °C:ethyl acetate = 1:4) to afford the product (**27**, 284 mg, 54 % yield) as white foam.

$[\alpha ]_{\text{D}}^{20}$ +248 (c 0.11, CHCl_3_). *R*_f_ 0.54 (petroleum ether:ethyl acetate = 1:4). *FTIR* (KBr, thin film) cm^−1^ 3419, 3029, 2919, 2791, 1598, 1488, 1350, 1258, 1147, 1039, 736. ^*1*^*H-NMR* (400 MHz, CDCl_3_), δ (ppm) 7.31–7.21 (2 × 4H, *m*), 7.20–7.13 (2 × 1H, *m*), 6.90 (2 × 1H, *d*, *J* = 6.8 Hz), 6.82 (2 × 1H, *t*, *J* = 7.6 Hz), 6.46 (2 × 1H, *t*, *J* = 7.6 Hz), 6.10 (2 × 1H, *d*, *J* = 7.6 Hz), 4.49 (2 × 1H, *brs*), 4.42 (2 × 1H, *d*, *J* = 16.4 Hz), 4.36 (2 × 1H, *d*, *J* = 16.4 Hz), 2.57–2.50 (2 × 2H, *m*), 2.42–2.33 (2 × 1H, *m*), 2.18 (2 × 3H, *s*), 1.94–1.87 (2 × 1H, *m*). ^*13*^*C-NMR* (100 MHz, CDCl_3_), δ (ppm) 152.69, 139.44, 133.08, 128.54, 128.09, 127.39, 126.87, 126.73, 124.13, 117.29, 106.80, 92.96, 63.27, 53.21, 52.61, 38.99, 35.74. EI–MS *m*/*z* (%) 526 (M^+^, 10%), 482 (6), 439 (26), 392 (3), 309 (12), 263 (49), 262 (100), 220 (15), 172 (26), 171 (19), 130 (8), 91 (42). *HRMSm*/*z* Found 526.3099, Calcd. for C_36_H_38_N_4_ [M]^+^ 526.3096.

### Synthesis of (+)-Chimonanthine

To a solution of liquid ammonia (freshly distilled and collected by Birch condenser, acetone–dry ice, 50–60 mL) at −78 °C was added sodium metal (ca. 124 mg, 5.4 mmol, 10 eq.). A solution of chimonanthine precursor (**27**, 284 mg, 0.54 mmol) in anhydrous THF (10 mL) was added to this dark blue solution of liquid ammonia. After stirring at −78 °C for 15 min, a powder of NH_4_Cl (433 mg, 8.1 mmol) was added in one portion followed by saturated aqueous solution of NH_4_Cl (5 mL). The resulting mixture was allowed to evaporate in fume hood. The residue was then diluted with water (20 mL) and extracted with dichloromethane (3 × 20 mL). The organic phases were combined and dried over anhydrous sodium sulfate. After filtration, the solvent was removed under reduced pressure and the crude product was chromatographed on silica gel (CH_2_Cl_2_:MeOH:NH_3_–H_2_O = 200:10:1) to afford the product (**2**, 177 mg, 95 %) as white plates.

m.p. 170–172 °C, $[\alpha ]_{\text{D}}^{20}$ +285 (c 0.12, EtOH). *R*_f_ 0.48 (CH_2_Cl_2_:MeOH:NH_3_–H_2_O = 200:10:1). *FTIR* (KBr, thin film) cm^−1^ 3404, 3219, 2930, 2856, 2797, 1601, 1480, 1252, 1161, 1031, 739, 648. ^*1*^*H-NMR* (300 MHz, CDCl_3_), δ (ppm) 7.19 (2 × 1H, *d*, *J* = 7.2 Hz), 6.99 (2 × 1H, *t*, *J* = 7.2 Hz), 6.67 (2 × 1H, *t*, *J* = 7.2 Hz), 6.54 (2 × 1H, *d*, *J* = 7.2 Hz), 4.34 (2 × 1H, *brs*), 4.12 (2 × 1H, *brs*), 2.63–2.48 (2 × 3H, *m*), 2.30 (2 × 3H, *s*), 2.18–2.01 (2 × 1H, *m*). ^*13*^*C-NMR* (75 MHz, CDCl_3_), δ (ppm) 150.74, 133.27, 128.27, 124.58, 118.78, 109.41, 85.40, 63.41, 52.83, 37.38, 35.66. EI–MS *m*/*z* (%) 346 (M^+^, 7 %), 302 (2), 259 (2), 245 (3), 231 (3), 190 (11), 173 (37), 172 (100), 157 (6), 143 (8), 130 (30), 117 (6), 103 (5), 85 (24), 83 (28). *HRMSm*/*z* Found 346.2151, Calcd. for C_22_H_26_N_4_ [M]^+^ 346.2157.

### Synthesis of (+)-Folicanthine

To a solution of amine **2** (35 mg, 0.1 mmol) in acetonitrile (3 mL) was added a solution of formalin (37 % HCHO in water, 39 μL, 0.52 mmol, 5.2 eq.) and sodium triacetoxyborohydride [NaBH(OAc)_3_, 110 mg, 0.52 mmol]. The resulting mixture was then stirred at room temperature under argon for 1 h. The mixture was then treated with a solution of methanol in dichloromethane saturated with ammonia (ca. 5 mL, CH_2_Cl_2_:MeOH = 95:5). After stirring for 5 min, the mixture was concentrated and the residue was chromatographed on silica gel (dichloromethane:methanol:NH_3_–H_2_O = 500:10:1) to afford the product (**3**, 35.5 mg, 95 %) as white plates.

m.p. 183–185 °C, $[\alpha ]_{\text{D}}^{20}$ +315 (c 0.10, MeOH). *R*_f_ 0.48 (CH_2_Cl_2_:MeOH:NH_3_–H_2_O = 200:10:1). *FTIR* (KBr, thin film) cm^−1^ 3434, 2944, 2784, 1601, 1488,1345, 1156, 1034, 730. ^*1*^*H-NMR* (300 MHz, CDCl_3_), δ (ppm) 7.02–6.88 (2 × 2H, *m*), 6.50 (2 × 1H, *t*, *J* = 7.5 Hz), 6.26 (2 × 1H, *d*, *J* = 7.5 Hz), 4.38 (2 × 1H, *brs*), 3.00 (2 × 3H, *s*), 2.70–2.58 (2 × 1H, *m*), 2.51–2.33 (2 × 2H, *m*), 2.41 (2 × 3H, *s*), 2.04–1.91 (2 × 1H, *m*). ^*13*^*C-NMR* (75 MHz, CDCl_3_), δ (ppm) 153.00, 132.95, 128.14, 123.70, 116.71, 105.90, 92.03, 62.74, 52.72, 38.03, 35.52, 35.39. EI–MS *m*/*z* (%) 374 (M^+^, 11 %), 273 (2), 187 (45), 186 (100), 172 (9), 157 (8), 145 (14), 144 (31), 130 (7), 115 (5), 85 (19), 83 (22). *HRMSm*/*z* Found 374.2480, Calcd. for C_24_H_30_N_4_ [M]^+^ 374.2470.

### Synthesis of (−)-Calycanthine

To a solution of acetic acid in D_2_O (0.43 M, 4 mL) was added chimonanthine **2** (35 mg, 0.1 mmol). The resulting mixture was then stirred at 95 °C for 18 h under an atmosphere of argon. After cooling down to room temperature, the mixture was diluted with dichloromethane (10 mL) and treated with a saturated aqueous solution of sodium bicarbonate (until pH 8). The combined aqueous phases were back-extracted with dichloromethane (3 × 8 mL). The organic phases were combined and dried over anhydrous sodium sulfate. After filtration and removal of the solvent, the residue was chromatographed on silica gel (dichloromethane:methanol:NH_3_–H_2_O = 500:10:1) to afford the product (**4**, 18 mg, 52 %) as a white solid.

m.p. 232–235 °C, $[\alpha ]_{\text{D}}^{20}$ −615 (c 0.15, EtOH). *FTIR* (KBr, thin film) cm^−1^ 3435, 2968, 1627, 1451, 1047, 744, 608. ^*1*^*H-NMR* (300 MHz, CDCl_3_), δ (ppm) 7.01 (2 × 1H, *d*, *J* = 7.5 Hz), 6.82 (2 × 1H, *t*, *J* = 7.5 Hz), 6.55 (2 × 1H, *t*, *J* = 7.5 Hz), 6.28 (2 × 1H, *d*, *J* = 7.5 Hz), 4.69 (2 × 1H, *brs*), 4.44 (2 × 1H, *s*), 3.17 (2 × 1H, *ddd*, *J* = 5.4, 13.2, 13.2 Hz), 2.70 (2 × 1H, *dd*, *J* = 4.6, 11.4 Hz), 2.46 (2 × 3H, *s*), 2.29 (2 × 1H, *ddd*, *J* = 3.6, 11.4, 11.4 Hz), 1.33 (2 × 1H, *dd*, *J* = 3.6, 13.2 Hz). ^*13*^*C-NMR* (75 MHz, CDCl_3_), δ (ppm) 145.00, 126.88, 124.58, 116.90, 112.36, 71.37, 46.63, 42.50, 35.96, 31.55. EI–MS *m*/*z* (%) 347 (M^+^+H, 31 %), 346 (M^+^, 100 %), 314 (5), 302 (18), 288 (29), 270 (23), 259 (13), 245 (28), 231 (59), 219 (11), 199 (9), 185 (13), 172 (21), 149 (30), 143 (27), 130 (27), 115 (14), 87 (69), 83 (72), 74 (91). *HRMSm*/*z* Found 346.2149, Calcd. for C_22_H_26_N_4_ [M]^+^ 346.2157.

### Synthesis of Compound **28**

To a solution of sulfinamide **21a** (2.42 g, 2.5 mmol) in methanol (50 mL) was added an aqueous solution of HCl (4 N, 1.88 mL, 7.5 mmol, 3 eq.). The resulting mixture was allowed to stir at room temperature for 1 h. The reaction mixture was then treated with saturated aqueous solution of sodium bicarbonate (until pH 8) and concentrated under reduced pressure. The mixture was diluted with water (80 mL) and extracted with dichloromethane (3 × 50 mL), the combined organic phases were dried over anhydrous Na_2_SO_4_. After removal of the solvent, the residue was chromatographed on silica gel (petroleum ether 60–90 °C:ethyl acetate = 2:1) to afford the amine (**28**) (1.81 g, 95 %) as a pale yellow syrup.

$[\alpha ]_{\text{D}}^{20}$ −241 (c 0.18, CHCl_3_). *ee* = 99.8 %, *R*_f_ 0.65 (petroleum ether:ethyl acetate = 2:1). *FTIR* (KBr, thin film) cm^−1^ 3454, 3061, 2921, 1699, 1609, 1485, 1361, 742. ^*1*^*H-NMR* (400 MHz, CDCl_3_), δ (ppm) 7.23–7.15 (2 × 5H, *m*), 7.11–7.04 (2 × 3H, *m*), 6.98 (2 × 1H, *t*, *J* = 6.8 Hz), 6.97 (2 × 1H, *d*, *J* = 7.2 Hz), 6.72–6.66 (2 × 2H, *m*), 6.61 (2 × 1H, *t*, *J* = 7.2 Hz), 6.35 (2 × 1H, *t*, *J* = 8.0 Hz), 5.52 (2 × 1H, *ddd*, *J* = 8.4, 10.0, 17.2 Hz), 5.00 (2 × 1H, *d*, *J* = 10.0 Hz), 4.75 (2 × 1H, *d*, *J* = 17.2 Hz), 4.48 (2 × 1H, *d*, *J* = 15.6 Hz), 4.42 (2 × 1H, *d*, *J* = 15.6 Hz), 3.50 (2 × 1H, *d*, *J* = 13.2 Hz), 3.33 (2 × 1H, *dd*, *J* = 4.0, 13.6 Hz), 3.01 (2 × 1H, *d*, *J* = 13.2 Hz), 2.54 (2 × 1H, *ddd*, *J* = 4.0, 8.6, 10.8 Hz), 2.40 (2 × 1H, *dd*, *J* = 10.8, 13.6 Hz), 0.91 (2 × 1H, *brs*). ^*13*^*C-NMR* (100 MHz, CDCl_3_), δ (ppm) 179.28, 143.86, 140.53, 140.01, 136.12, 128.44, 128.07, 128.02, 127.87, 127.70, 127.31, 126.32, 124.88, 121.36, 115.53, 108.44, 57.58, 54.78, 50.31, 44.45, 34.85. +TOF-MS *m*/*z* (%) 763 (M^+^+1, 100 %), 382 (15), 275 (2), 236 (20). *HRMSm*/*z* Found 763.4015, Calcd. for C_52_H_51_N_4_O_2_ [M+H]^+^ 763.4012.

### Synthesis of Compound **28a**

To diamine **28** (763 mg, 1 mmol) in dichloromethane (20 mL) was added a powder of *N*-chlorosuccinimide (NCS, 400 mg, 3 mmol, 3.0 eq.). The resulting mixture was stirred at room temperature for 1 h. After which, a solution of saturated Na_2_CO_3_ (5 mL) was added. The mixture was then allowed to stir at room temperature for 10 min. The aqueous phase was extracted with dichloromethane (3 × 15 mL) and the combined organic phases were dried over anhydrous Na_2_SO_4_. After removal of the solvent under reduced pressure, the residue was chromatographed on silica gel (petroleum ether 60–90 °C:ethyl acetate = 7:1) to afford the product (**28a**, 789 mg, 95 % yield) as a pale yellow syrup.

#### **28a**

$[\alpha ]_{\text{D}}^{20}$ −278 (c 0.50, CHCl_3_). *R*_f_ 0.45 (petroleum ether:ethyl acetate = 7:1). *FTIR* (KBr, thin film) cm^−1^ 3435, 2920, 2352, 1703, 1615, 1361, 1047, 745, 602. ^*1*^*H-NMR* (400 MHz, CDCl_3_), δ (ppm) 7.32–7.19 (2 × 5H, *m*), 7.10 (2 × 1H, *t*, *J* = 7.2 Hz), 7.03 (2 × 1H, *d*, *J* = 7.6 Hz), 7.02 (2 × 1H, *t*, *J* = 7.2 Hz), 6.89 (2 × 1H, *t*, *J* = 7.6 Hz), 6.87 (2 × 1H, *d*, *J* = 7.6 Hz), 6.69 (2 × 2H, *d*, *J* = 7.2 Hz), 6.50 (2 × 1H, *t*, *J* = 7.2 Hz), 6.34 (2 × 1H, *d*, *J* = 7.6 Hz), 5.95 (2 × 1H, *ddd*, *J* = 7.6, 10.4, 17.2 Hz), 5.19 (2 × 1H, *dd*, *J* = 1.2, 10.4 Hz), 4.86 (2 × 1H, *dd*, *J* = 1.2, 17.2 Hz), 4.76 (2 × 1H, *d*, *J* = 15.6 Hz), 4.38 (2 × 1H, *d*, *J* = 15.6 Hz), 3.80 (2 × 1H, *d*, *J* = 12.8 Hz), 3.50–3.40 (2 × 2H, *m*), 3.30–2.89 (2 × 2H, *m*). ^*13*^*C-NMR* (100 MHz, CDCl_3_), δ (ppm) 176.99, 143.66, 136.37, 136.12, 134.58, 129.59, 128.56, 128.45, 128.10, 128.05, 127.60, 127.37, 127.29, 124.74, 121.43, 119.58, 108.44, 67.79, 64.75, 54.63, 44.29, 31.92. *HRMSm*/*z* Found 831.3222, Calcd. for C_52_H_49_Cl_2_N_4_O_2_ [M+H]^+^ 831.3233.

### Synthesis of Compound **29**

To a solution of dichloroamine **28a** (789 mg, 0.95 mmol) in anhydrous THF (20 mL) was added a powder of potassium *tert*-butoxide (*t*-BuOK, 320 mg, 2.85 mmol, 3.0 eq.). The resulting mixture was stirred at 0 °C for 1 h. After which, the reaction mixture was treated with a solution of saturated NH_4_Cl (5 mL). After removal of the solvents, the residue was diluted with water (25 mL) and extracted with dichloromethane (3 × 25 mL), and the combined organic phases was dried over anhydrous sodium sulfate. After filtration and removal of the solvent under reduced pressure, the crude product was obtained (670 mg, 93 % yield) as pale yellowish oil. The crude imine was dissolved in ethanol (20 mL). To this solution was added phenylhydrazine (0.26 mL, 2.64 mmol, 3.0 eq.). The resulting mixture was allowed to stir at room temperature for 3 h. After removal of the solvent under reduced pressure, the residue was chromatographed on silica gel (dichloromethane:methanol:NH_3_–H_2_O = 400:10:1 → 100:10:1) to afford the product (**29**, 489 mg, 88 % yield over two steps) as a pale yellow syrup.

$[\alpha ]_{\text{D}}^{20}$ −225 (c 0.28, CHCl_3_). *R*_f_ 0.44 (CH_2_Cl_2_:MeOH = 10:1). *FTIR* (KBr, thin film) cm^−1^ 3362, 3055, 1702, 1607, 1486, 1360, 1170, 997, 916, 751. ^*1*^*H-NMR* (400 MHz, CD_3_OD), δ (ppm) 7.51 (2 × 2H, *d*, *J* = 7.6 Hz), 7.43–7.33 (2 × 3H, *m*), 7.05 (2 × 1H, *d*, *J* = 8.0 Hz), 7.02 (2 × 1H, *t*, *J* = 7.6 Hz), 6.77 (2 × 1H, *t*, *J* = 7.6 Hz), 6.61 (2 × 1H, *d*, *J* = 8.0 Hz), 5.50 (2 × 1H, *ddd*, *J* = 7.2, 10.4, 17.2 Hz), 5.18 (2 × 1H, *d*, *J* = 15.6 Hz), 4.64–4.53 (2 × 3H, *m*), 3.31 (2 × 1H, *dd*, *J* = 6.8, 13.6 Hz), 2.93 (2 × 1H, *dd*, *J* = 6.4, 13.2 Hz), 2.59 (2 × 1H, *dd*, *J* = 6.8, 13.6 Hz). ^*13*^*C-NMR* (100 MHz, CD_3_OD), δ (ppm) 179.10, 144.37, 142.14, 137.21, 129.64, 129.46, 128.76, 128.60, 125.45, 122.76, 114.37, 109.83, 56.14, 52.99, 45.11, 36.86. EI–MS *m*/*z* (%) 583 (M^+^+1, 11 %), 513 (20), 496 (7), 444 (4), 292 (76), 291 (24), 275 (12), 261 (6), 236 (100), 235 (58), 223 (7), 206 (4), 158 (15), 130 (7), 91 (100). *HRMSm*/*z* Found 582.2991, Calcd. for C_38_H_38_N_4_O_2_ [M]^+^ 582.2995.

### Synthesis of Compound **30**

Diamine **29** (291 mg, 0.5 mmol) in THF (20 mL) was degassed and purged with argon (three times). A solution of diisobutylaluminium hydride (DIBAL-H, 1.0 M in THF, 5 mL, 5 mmol, 10.0 eq.) was added at 0 °C and the resulting mixture was stirred at 0 °C for 1 h, then at room temperature for 2 h and finally at 80 °C (oil bath) under argon for 15 h. After cooling to room temperature, a saturated aqueous solution of potassium sodium tartrate (5 mL) was added and the resulting mixture was stirred at room temperature for 2 h. The mixture was diluted with water (20 mL) and extracted with ethyl acetate (3 × 20 mL). The combined organic phases were dried over anhydrous sodium sulfate. After filtration and removal of the solvent, the residue was chromatographed on silica gel (petroleum ether 60–90 °C:ethyl acetate = 3:1) to afford the product (**30**, 143 mg, 52 % yield) as a yellow oil.

$[\alpha ]_{\text{D}}^{20}$*−*210 (c 0.18, MeOH). *R*_f_ 0.56 (petroleum ether:ethyl acetate = 3:1). *FTIR* (KBr, thin film) cm^−1^ 3437, 2919, 1601, 1491, 1349, 1088, 909, 738. ^*1*^*H-NMR* (400 MHz, CDCl_3_), δ (ppm) 7.30–7.12 (2 × 5H, *m*), 7.03 (2 × 1H, *d*, *J* = 7.2 Hz), 6.92 (2 × 1H, *t*, *J* = 7.2 Hz), 6.48 (2 × 1H, *t*, *J* = 7.2 Hz), 6.20 (2 × 1H, *d*, *J* = 8.0 Hz), 5.64 (2 × 1H, *ddd*, *J* = 6.8, 10.4, 17.2 Hz), 5.00 (2 × 1H, *d*, *J* = 17.2 Hz), 4.92 (2 × 1H, *d*, *J* = 10.4 Hz), 4.76 (2 × 1H, *s*), 4.38 (2 × 1H, *d*, *J* = 16.0 Hz), 4.31 (2 × 1H, *d*, *J* = 16.0 Hz), 3.40–3.30 (2 × 1H, *m*), 2.10–1.97 (2 × 2H, *m*), 1.70 (2 × 1H, *brs*). ^*13*^*C-NMR* (100 MHz, CDCl_3_), δ (ppm) 152.27, 139.11, 139.00, 131.43, 128.71, 128.60, 127.54, 127.20, 124.61, 116.56, 115.58, 105.21, 85.91, 62.82, 59.74, 49.15, 44.84. EI–MS *m*/*z* (%) 550 (M^+^, 10 %), 308 (3), 274 (100), 259 (3), 236 (3), 220 (19), 183 (18), 169 (5), 130 (7), 118 (6), 91 (62). *HRMSm*/*z* Found 550.3096, Calcd. for C_38_H_38_N_4_ [M]^+^ 550.3096.

### Synthesis of Compound **31**

To a solution of liquid ammonia (freshly distilled and collected by Birch condenser, acetone–dry ice, 20–25 mL) at −78 °C was added sodium metal (ca. 60 mg, 2.6 mmol, 10 eq.). A solution of diamine **30** (143 mg, 0.26 mmol) in anhydrous THF (5 mL) was added to this dark blue solution of liquid ammonia. After stirring at −78 °C for 15 min, a powder of NH_4_Cl (214 mg, 4 mmol) was added in one portion followed by saturated aqueous solution of NH_4_Cl (5 mL). The resulting mixture was allowed to evaporate in fume hood. The residue was then diluted with water (20 mL) and extracted with dichloromethane (3 × 20 mL). The organic phases were combined and dried over anhydrous sodium sulfate. After filtration, the solvent was removed under reduced pressure and the crude product was chromatographed on silica gel (CH_2_Cl_2_:MeOH:NH_3_–H_2_O = 300:10:1) to afford the product (**31**, 92 mg, 95 %) as a yellow syrup.

$[\alpha ]_{\text{D}}^{20}$ −310 (c 0.30, MeOH). *R*_f_ 0.48 (CH_2_Cl_2_:MeOH:NH_3_–H_2_O = 30:1:0.1). *FTIR* (KBr, thin film) cm^−1^ 3404, 2922, 2352, 1605, 1474, 1094, 928, 744. ^*1*^*H-NMR* (400 MHz, CD_3_OD), δ (ppm) 7.19 (2 × 1H, *d*, *J* = 7.2 Hz), 6.96 (2 × 1H, *t*, *J* = 7.6 Hz), 6.62 (2 × 1H, *t*, *J* = 7.2 Hz), 6.52 (2 × 1H, *d*, *J* = 7.6 Hz), 5.82 (2 × 1H, *ddd*, *J* = 7.2, 10.4, 17.2 Hz), 5.16 (2 × 1H, *d*, *J* = 17.2 Hz), 5.10 (2 × 1H, *d*, *J* = 10.4 Hz), 5.09 (2 × 1H, *s*), 3.45–3.36 (2 × 1H, *m*), 2.29–2.17 (2 × 2H, *m*). ^*13*^*C-NMR* (100 MHz, CD_3_OD), δ (ppm) 152.62, 138.09, 131.63, 129.70, 125.36, 119.32, 117.74, 109.82, 81.39, 65.04, 61.28, 43.61. EI–MS *m*/*z* (%) 370 (M^+^, 22 %), 314 (4), 260 (5), 245 (11), 232 (4), 219 (3), 184 (100), 183 (22), 169 (13), 158 (6), 131 (27), 130 (30), 117 (4), 103 (4), 91 (3). *HRMSm*/*z* Found 370.2155, Calcd. for C_24_H_26_N_4_ [M]^+^ 370.2157.

### Synthesis of Compound **33**

To a solution of amine **31** (74 mg, 0.2 mmol) and FMOC-(*S*)-MePhe–OH (200 mg, 0.5 mmol, 2.5 eq.) in anhydrous DMF (3 mL) was added Et_3_N (0.14 mL, 1 mmol, 5.0 eq.) and *O*-(7-azabenzotriazol-1-yl)-*N*,*N*,*N′*,*N′*-tetramethyluronium hexafluoro-phosphate (HATU, 190 mg, 0.5 mmol, 2.5 eq.) at 0 °C. The resulting mixture was then stirred at room temperature for 12 h. The mixture was then treated with an aqueous solution of LiCl (5 %, ca. 10 mL). After stirring for 5 min, the mixture was diluted with water (10 mL) and extracted with ethyl acetate (3 × 20 mL). The combined organic phases were washed with 5 % aqueous solution of LiCl (5 mL) and dried over anhydrous sodium sulfate. After filtration, the solvent was removed under reduced pressure to afford the product (**32**, 198 mg, 87 %) which was used directly in the next step without further purification. To a solution of olefin **32** (114 mg, 0.1 mmol) in THF (3 mL) and H_2_O (1 mL) was added 4-methylmorpholine-*N*-oxide (47 mg, 0.4 mmol, 4.0 eq.) and OsO_4_ (20 mg/mL in water, 0.13 mL, 0.01 mmol, 0.1 eq.). The resulting mixture was then stirred at room temperature for 20 h under nitrogen. After which, NaIO_4_ (86 mg, 0.4 mmol, 4.0 eq.) was added and the mixture was allowed to stir at room temperature for 3 h. The resulting mixture was then diluted with water (10 mL) and extracted with ethyl acetate (3 × 15 mL). The organic phases were combined and dried over anhydrous sodium sulfate. After filtration and removal of the solvent, the residue was chromatographed on silica gel (petroleum ether 60–90 °C:ethyl acetate = 2:1) to afford the dialdehyde (**33**, 106 mg, 93 %) as white plates.

m.p. 155–157 °C, $[\alpha ]_{\text{D}}^{20}$ −318 (c 0.35, CHCl_3_). *R*_f_ 0.41 (petroleum ether:ethyl acetate = 3:1). *FTIR* (KBr, thin film) cm^−1^ 3425, 2920, 1730, 1666, 1450, 1321, 1147, 1071, 744. ^*1*^*H-NMR* (400 MHz, CDCl_3_), δ (ppm) 8.95 (2 × 1H, *d*, *J* = 4.4 Hz), 7.78 (2 × 2H, *d*, *J* = 7.6 Hz), 7.58–7.00 (2 × 12H, *m*), 6.94 (2 × 1H, *t*, *J* = 7.6 Hz), 6.63 (2 × 1H, *t*, *J* = 7.6 Hz), 6.32 (2 × 1H, *d*, *J* = 7.6 Hz), 5.12 (2 × 1H, *t*, *J* = 8.0 Hz), 4.92 (2 × 1H, *s*), 4.48 (2 × 1H, *dd*, *J* = 7.2, 10.4 Hz), 4.36 (2 × 1H, *dd*, *J* = 7.2, 10.4 Hz), 4.25 (2 × 1H, *t*, *J* = 6.8 Hz), 3.81–3.74 (2 × 1H, *m*), 3.12 (2 × 3H, *s*), 2.97–2.85 (2 × 2H, *m*), 2.08–2.00 (2 × 1H, *m*), 1.92–1.82 (2 × 1H, *m*). ^*13*^*C-NMR* (100 MHz, CDCl_3_), δ (ppm) 195.77, 170.75, 157.86, 149.38, 143.84, 143.66, 141.47, 135.72, 130.06, 129.08, 128.99, 128.03, 127.99, 127.71, 127.29, 126.22, 125.17, 125.08, 123.80, 120.24, 118.77, 109.18, 79.14, 68.60, 64.42, 62.14, 56.28, 47.23, 36.83, 32.84, 30.40. *HRMSm*/*z* Found 1141.4830, Calcd. for C_72_H_65_N_6_O_8_ (M+H)^+^ 1141.4864; Found 1163.4662, Calcd. for C_72_H_64_N_6_NaO_8_ [M+Na]^+^ 1163.4683.

### Synthesis of (−)-Ditryptophenaline

To a solution of dialdehyde **33** (106 mg, 0.093 mmol) in THF (2 mL), water (2 mL) and *t*-BuOH (0.5 mL) was added 2-methyl-2-butene (1 mL), NaClO_2_ (80% purity, 105 mg, 0.93 mmol, 10 eq.) and KH_2_PO_4_ (253 mg, 1.86 mmol, 20 eq.). The resulting mixture was then stirred at room temperature for 15 h. After which, the mixture was diluted with a saturated aqueous solution of NH_4_Cl (40 mL) and extracted with ethyl acetate (3 × 20 mL). The combined organic phases were washed with brine (10 mL) and dried over anhydrous sodium sulfate. After filtration and removal of the solvent, the crude product was obtained (101 mg, 93 %) and used in next step without further purification. The diacid (101 mg) was dissolved in THF (3 mL) and treated with piperidine (0.5 mL) at room temperature for 4 h. After removal of solvent and excess piperidine, the crude yellowish oil (60 mg) was obtained and used in next step without further purification. To a solution of aminoacid (60 mg, ~0.082 mmol) in dichloromethane (4 mL) was added DCC (169 mg, 0.82 mmol, 10 eq.). The resulting mixture was stirred at 40 °C for 15 h. After removal of the solvent, the residue was chromatographed on silica gel (petroleum ether 60–90 °C:ethyl acetate = 3:1 → 1:5) to afford the product (**5**, 50 mg, 78 % over three steps) as white plates.

m.p. 201–202 °C, $[\alpha ]_{\text{D}}^{20}$ −292 (c 0.40, CH_2_Cl_2_). *R*_f_ 0.41 (petroleum ether:ethyl acetate = 1:5). *FTIR* (KBr, thin film) cm^−1^ 3426, 2921, 1651, 1452, 1075, 749. ^*1*^*H-NMR* (400 MHz, CDCl_3_), δ (ppm) 7.55 (2 × 2H, *t*, *J* = 7.6 Hz), 7.50 (2 × 1H, *t*, *J* = 7.2 Hz), 7.13 (2 × 2H, *d*, *J* = 7.2 Hz), 7.06 (2 × 1H, *dt*, *J* = 0.8, 7.6 Hz), 6.96 (2 × 1H, *d*, *J* = 7.6 Hz), 6.69 (2 × 1H, *t*, *J* = 7.2 Hz), 6.54 (2 × 1H, *d*, *J* = 7.6 Hz), 4.80 (2 × 1H, *s*), 4.68 (2 × 1H, *s*), 4.27–4.22 (2 × 1H, *m*), 3.65 (2 × 1H, *dd*, *J* = 4.8, 12.0 Hz), 3.52 (2 × 1H, *dd*, *J* = 3.0, 14.4 Hz), 3.24 (2 × 1H, *dd*, *J* = 4.4, 14.4 Hz), 3.02 (2 × 3H, *s*), 2.01 (2 × 1H, *dd*, *J* = 4.8, 12.4 Hz), 1.56 (2 × 1H, *t*, *J* = 12.4 Hz). ^*13*^*C-NMR* (100 MHz, CDCl_3_), δ (ppm) 165.55, 164.10, 150.30, 134.64, 129.78, 129.52, 129.46, 128.07, 126.62, 125.89, 119.08, 109.78, 63.27, 59.09, 58.70, 36.34, 36.14, 32.69. *HRMSm*/*z* Found 693.3178, Calcd. for C_42_H_41_N_6_O_4_ [M+H]^+^ 693.3189.

## Electronic supplementary material

Supplementary material 1 (PDF 3458 kb)
