# Supplementary material for: Total Synthesis of Dimeric HPI Alkaloids
Source: Nat Prod Bioprospect. 2016 Mar 11;6(2):117–39. doi: 10.1007/s13659-016-0092-8 (PMC4805652; doi:10.1007/s13659-016-0092-8)
Supplement: Supplementary file 1 — Supplementary material 1 (PDF 3458 kb) [file 13659_2016_92_MOESM1_ESM.pdf]

Total Synthesis of Dimeric HPI Alkaloids

Xianfu Shen, Yongyun Zhou, Yongkai Xi, Jingfeng Zhao, and Hongbin Zhang*

Key Laboratory of Medicinal Chemistry for Natural Resource, Ministry of Education, School of Chemical Science and Technology, Yunnan University, Kunming, Yunnan 650091, P. R. China.

Email: zhanghb@ynu.edu.cn

Supporting Information

Copies of ^{1}H and ^{13}C spectra of new compounds

Corresponding author:

Professor Hongbin Zhang
Key Laboratory of Medicinal Chemistry for Natural Resource
Ministry of Education
School of Chemical Science and Technology
Yunnan University
Kunming, Yunnan 650091
P. R. China.
Fax: 86-871-5035538

Supporting information for

Total Synthesis of Dimeric HPI Alkaloids

Xianfu Shen, Yongyun Zhou, Yongkai Xi, Jingfeng Zhao, and Hongbin Zhang*

Key Laboratory of Medicinal Chemistry for Natural Resource, Ministry of Education, School of Chemical Science and Technology, Yunnan University, Kunming, Yunnan 650091, P. R. China

Fax: 86-871-5035538. E-mail: zhanghb@ynu.edu.cn

Crystal data for compound **23**: $C_{70}H_{72}F_2N_4O_{10}S_4$•$C_4H_8O_2$, M = 1383.66, orthorhombic, a = 10.1404(9) Å, b = 25.087(2) Å, c = 27.663(3) Å, α = 90.00°, β = 90.00°, γ = 90.00°, V = 7037.4(11) Å^3, T = 100(2) K, space group P212121, Z = 4, μ(MoKα) = 0.205 mm^{-1}, 70275 reflections measured, 17497 independent reflections (R_{int} = 0.0645). The final R_1 values were 0.0434 ($I > 2\sigma(I)$). The final $wR(F^2)$ values were 0.0969 ($I > 2\sigma(I)$). The final R_1 values were 0.0596 (all data). The final $wR(F^2)$ values were 0.1063 (all data). The goodness of fit on F^2 was 1.030. Flack parameter = 0.03(4).

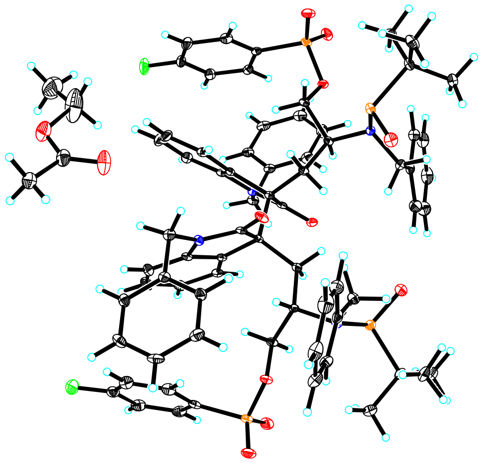

View of the molecules in an asymmetric unit.

Displacement ellipsoids are drawn at the 30% probability level.

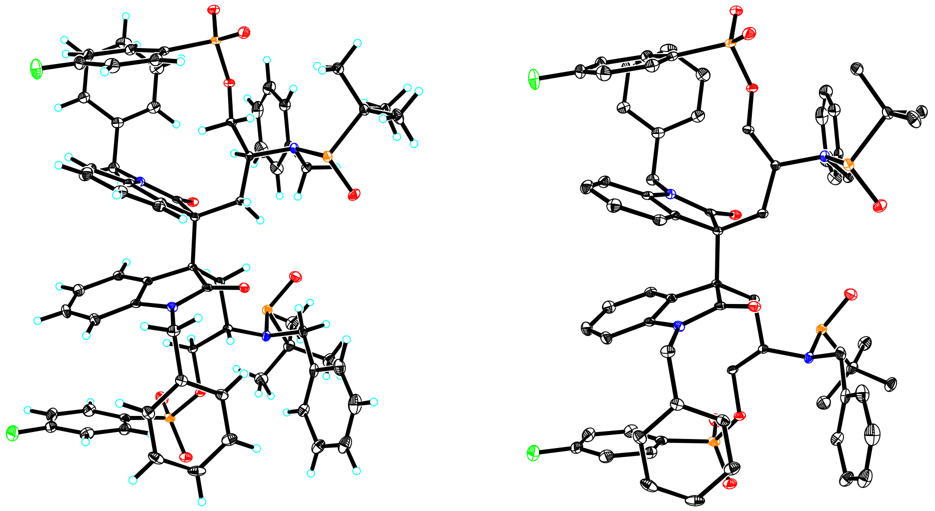

View of a molecule of zhb_s3 with the atom-labelling scheme.
Displacement ellipsoids are drawn at the 30% probability level.

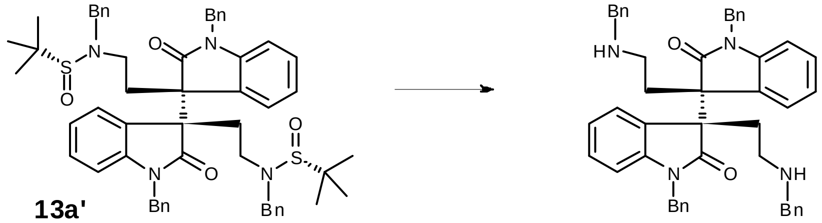

13a'

24 $C_{48}H_{46}N_4O_2$
Mol. Wt.: 710.90

24: (3R,3'R)-1,1'-dibenzyl-3,3'-bis(2-(benzylamino)ethyl)-[3,3'-biindoline]-2,2(3R,3'R)-1,1'-dibenzyl-3,3'-bis(2-(benzylamino)ethyl)-[3,3'-biindoline]-2,2'-dione

Sulfinamide (**13a'** + **13b'**: 3.59g, 3.9 mmol) was dissolved in methanol (60 mL). To this mixture was added an aqueous solution of HCl (4N, 2.9 mL, 11.7 mmol, 3 eq.). The resulting mixture was allowed to stir at room temperature under nitrogen for 1 h. The reaction mixture was then treated with saturated aqueous solution of sodium bicarbonate (~50 mL) and concentrated under reduced pressure. The mixture was diluted with water (100 mL) and extracted with dichloromethane (3 × 50 mL), the combined organic phases were dried over anhydrous Na_2SO_4. After removal of the solvent, the residue was chromatographed on silica gel (Dichloromethane : Methanol = 20 : 1) to afford the amine (**24**) (2.63g, 95%) as white foam.*[1] The diamine was dissolved in methanol (20 mL) and HCl (2N, 5.6 mL, 11.1 mmol, 3.0 eq.) was added. This solution was allowed to crystallize at room temperature. The needle-like crystals were collected and subjected to HPLC analysis (a 1:1 mixture of C3*R*-C3'*R* and C3*S*-C3'*S* enantiomers). The mother liquid was then treated with saturated aqueous solution of sodium bicarbonate to pH = 8,

and extracted with dichloromethane (3). The combined organic phases were dried over anhydrous Na_2SO_4. After removal of the solvents, the enantiomeric pure product **24** was obtained (1.60 g, 61%) as a pale yellow syrup.*[2]

24: $[\alpha]^{20}_D$ +171 (c 0.12, MeOH). *ee* = 99.1%. R_f: 0.40 (CH_2Cl_2: MeOH: Et_3N= 20: 1 : 0.01). **FTIR** (KBr, thin film) cm^{-1}: 3426, 2967, 1701, 1611, 1456, 1365, 1174, 1047, 746, 700. **^{1}H-NMR** (400 MHz, $CDCl_3$), δ (ppm): 7.32-7.16 (2×9H, *m*), 7.10 (2×2H, *d*, *J* = 7.2 Hz), 7.04 (2×1H, *d*, *J* = 7.2 Hz), 6.97 (2×1H, *t*, *J* = 7.6 Hz), 6.71 (2×1H, *t*, *J* = 7.6 Hz), 6.39 (2×1H, *d*, *J* = 7.6 Hz), 5.03 (2×1H, *d*, *J* = 15.6 Hz), 4.45 (2×1H, *d*, *J* = 15.6 Hz), 3.60 (2×1H, *d*, *J* = 13.6 Hz), 3.53 (2×1H, *d*, *J* = 13.6 Hz), 3.26 (2×1H, *ddd*, *J* = 5.6, 6.0, 13.2 Hz), 2.64 (2×1H, *ddd*, *J* = 7.6, 8.0, 13.2 Hz), 2.27-2.17 (2×2H, *m*). **^{13}C-NMR** (100 MHz, $CDCl_3$), δ (ppm): 178.03, 143.09, 140.31, 135.85, 128.68, 128.32, 128.06, 128.01, 127.85, 127.58, 126.74, 124.12, 121.76, 108.52, 55.14, 53.65, 45.41, 44.12, 29.22. EI-MS *m*/*z* (%) : 710 (M^+, 2%), 577 (1), 356 (4), 344 (2), 262 (3), 236 (4), 223 (4), 134 (8), 118 (12), 106 (21), 91 (100). **HRMS** *m*/*z* Found: 710.3622, Calcd. for $C_{48}H_{46}N_4O_2$ $(M)^+$: 710.3621.

(**13a'** racemic)*[1] Chiral HPLC analysis: 25 ºC; column: DAICEL Chiralcel OD-H (0.46 cm Φ × 25 cm); mobile phase: hexane / *iso*-propanol 85 / 15; flow rate: 0.500 mL/min; detection, UV 254 nm; tR_1 = 48.247 min, Area = 39.4094; tR_2 = 53.887 min, Area = 60.5906.
(**24**)*[2] Chiral HPLC analysis: 25 ºC; column: DAICEL Chiralcel OD-H (0.46 cm Φ × 25 cm); mobile phase: hexane / *iso*-propanol 85/ 15; flow rate: 0.500 mL/min; detection, UV 254 nm; tR_1 = 50.183 min, Area = 0.4883; tR_2 = 52.932 min, Area = 99.5117.

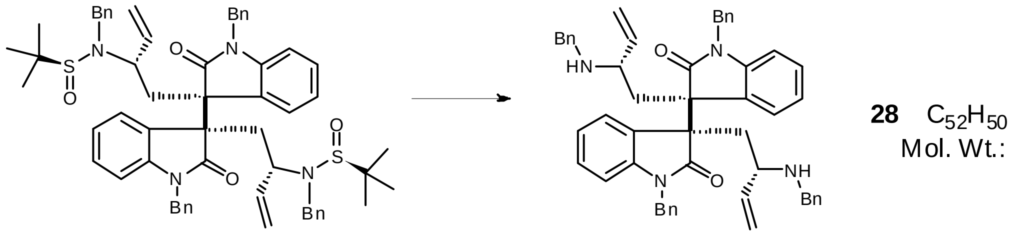

21b

28 $C_{52}H_{50}N_4O_2$
Mol. Wt.: 762.98

28: (3*S*,3'*S*)-1,1'-dibenzyl-3,3'-bis((*S*)-2-(benzylamino)but-3-en-1-yl)-[3,3'-biindoline]-2,2'-dione

To a solution of sulfinamide **21b** (2.42 g, 2.5 mmol) in methanol (50 mL) was added an aqueous solution of HCl (4N, 1.88 mL, 7.5 mmol, 3 eq.). The resulting mixture was allowed to stir at room temperature for 1 h. The reaction mixture was then treated with saturated aqueous solution of sodium bicarbonate (until pH = 8) and concentrated under reduced pressure. The mixture was diluted with water (80 mL) and extracted with dichloromethane (3 × 50 mL), the combined organic phases were dried over anhydrous Na_2SO_4. After removal of the solvent, the residue was chromatographed on silica gel (Petroleum ether 60-90 ºC: ethyl acetate = 2:1) to afford the amine (**28**) (1.81 g, 95%) as a pale yellow syrup.

$[\alpha]^{20}_{D}$ −241 (c 0.18, $CHCl_3$). *ee* = 99.8%, R_f: 0.65 (Petroleum ether: ethyl acetate = 2:1). **FTIR** (KBr, thin film) cm^{-1}: 3454, 3061, 2921, 1699, 1609, 1485, 1361, 742. **^{1}H-NMR** (400 MHz, $CDCl_3$), δ (ppm): 7.23-7.15 (2×5H, *m*), 7.11-7.04 (2×3H, *m*), 6.98 (2×1H, *t*, *J* = 6.8 Hz), 6.97 (2×1H, *d*, *J* = 7.2 Hz), 6.72-6.66 (2×2H, *m*), 6.61 (2×1H, *t*, *J* = 7.2 Hz), 6.35 (2×1H, *t*, *J* = 8.0 Hz), 5.52 (2×1H, *ddd*, *J* = 8.4, 10.0, 17.2 Hz), 5.00 (2×1H, *d*, *J* = 10.0 Hz), 4.75 (2×1H, *d*, *J* = 17.2 Hz), 4.48 (2×1H, *d*, *J* = 15.6 Hz), 4.42 (2×1H, *d*, *J* = 15.6 Hz), 3.50 (2×1H, *d*, *J* = 13.2 Hz), 3.33 (2×1H, *dd*, *J* = 4.0, 13.6 Hz), 3.01 (2×1H, *d*, *J* = 13.2 Hz), 2.54 (2×1H, *ddd*, *J* = 4.0, 8.6, 10.8 Hz), 2.40 (2×1H, *dd*, *J* = 10.8, 13.6 Hz), 0.91 (2×1H, *brs*). **^{13}C-NMR** (100 MHz, $CDCl_3$), δ (ppm): 179.28, 143.86, 140.53, 140.01, 136.12, 128.44, 128.07, 128.02, 127.87, 127.70, 127.31, 126.32, 124.88, 121.36, 115.53, 108.44, 57.58, 54.78, 50.31, 44.45, 34.85. +TOF-MS *m/z* (%) : 763 (M^++1, 100%), 382 (15), 275 (2), 236 (20). **HRMS** *m/z* Found: 763.4015, Calcd. for $C_{52}H_{51}N_4O_2$ $(M+H)^+$: 763.4012.

(**28** racemic) Chiral HPLC analysis: 25 ºC; column: DAICEL Chiralcel AD-H (0.46 cm Φ × 25 cm); mobile phase: hexane / iso-propanol 90 / 10; flow rate: 0.600 mL/min; detection, UV 254 nm; tR_1 = 11.693 min, Area = 49.2003; tR_2 = 24.015 min, Area = 50.7997.

(**28**) Chiral HPLC analysis: 25 ºC; column: DAICEL Chiralcel AD-H (0.46 cm Φ × 25 cm); mobile phase: hexane / iso-propanol 90 / 10; flow rate: 0.600 mL/min; detection, UV 254 nm; tR_1 = 11.769 min, Area = 0.1004; tR_2 = 24.349 min, Area = 99.8996.

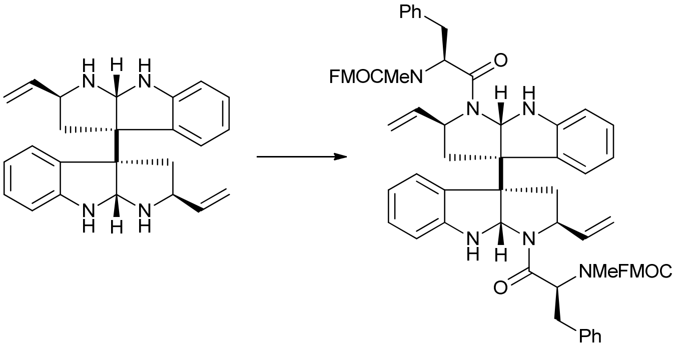

32: bis((9*H*-fluoren-9-yl)methyl) ((2*S*,2'*S*)-((2*S*,2'*S*,3a*S*,3'a*S*,8a*S*,8'a*S*)-2,2'-divinyl-2,2',3,3',8,8a,8',8'a-octahydro-1*H*,1'*H*-[3a,3'a-bipyrrolo[2,3-*b*]indole]-1,1'-diyl)bis(1-oxo-3-phenylpropane-2,1-diyl))bis(methylcarbamate)

32: $C_{74}H_{68}N_6O_6$
Mol. Wt.: 1137.37

To a solution of amine **31** (74 mg, 0.2 mmol) and FMOC-(*S*)-MePhe-OH (200 mg, 0.5 mmol, 2.5 eq.) in anhydrous DMF (3 ml) was added Et_3N (0.14 mL, 1 mmol, 5.0 eq.) and *O*-(7-azabenzotriazol-1-yl)-*N,N,N',N'*-tetramethyluronium hexafluorophosphate (HATU, 190 mg, 0.5 mmol, 2.5 eq.) at 0 ºC. The resulting mixture was then stirred at room temperature for 12 h. The mixture was then treated with an aqueous solution of LiCl (5%, ca. 10 mL). After stirring for 5 minutes, the mixture was diluted with water (10 mL) and extracted with ethyl acetate (3 × 20 mL). The combined organic phases were washed with 5% aqueous solution of LiCl (5 mL) and dried over anhydrous sodium sulfate. After filtration, the solvent was removed under reduced pressure to afford the product (**32**, 198 mg, 87%) which was used directly in the next step without further purification.

$[\alpha]^{20}_{D}$ −265 (c 0.40, $CHCl_3$). R_f: 0.48 (Petroleum ether: ethyl acetate = 3:1). **HRMS** *m/z* Found: 1137.5271, Calcd. for $C_{74}H_{69}N_4O_6$ $(M+H)^+$: 1137.5279.

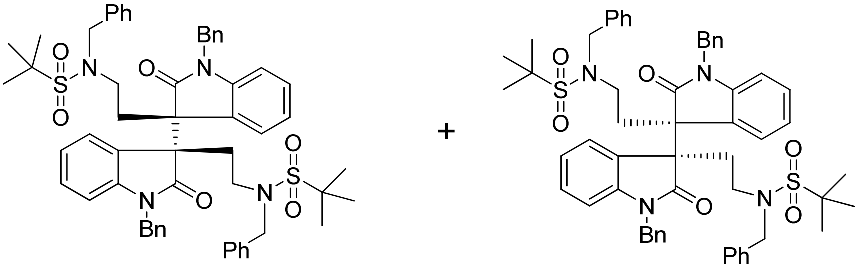

^{1}H-NMR (400 MHz, $CDCl_3$), δ (ppm): 7.45 (2×2H, *d*, *J* = 7.2 Hz), 7.36 (2×2H, *t*, *J* = 7.2 Hz), 7.33-7.24 (2×4H, *m*), 7.14-7.08 (2×2H, *m*), 6.81 (2×1H, *t*, *J* = 7.6 Hz), 6.59 (2×1H, *d*, *J* = 7.2 Hz), 6.49 (2×1H, *t*, *J* = 7.6 Hz), 6.25 (2×1H, *d*, *J* = 8.0 Hz), 5.02 (2×1H, *d*, *J* = 15.6 Hz), 4.72-4.55 (2×1H, *m*), 4.48-4.32 (2×1H, *m*), 4.27 (2×1H, *d*, *J* = 15.6 Hz), 3.27-3.14 (2×1H, *m*), 2.69-2.54 (2×1H, *m*), 2.46-2.31 (2×2H, *m*), 1.41 (2×9H, *s*). **^{13}C-NMR** (100 MHz, $CDCl_3$), δ (ppm): 176.32, 142.37, 136.18, 135.44, 129.09, 128.91, 128.65, 128.49, 127.87, 127.78, 127.65, 126.38, 123.56, 122.11, 108.57, 61.60, 54.13, 51.89, 43.90, 43.43, 27.04, 24.96.

Synthesis of compound **19a**: A mixture of tris(dibenzylideneacetone)dipalladium [$Pd_2(dba)_3$, FW 915.72, 46.0 mg, 0.05 mmol, 0.025 eq.], triphenylphosphine (FW 262.29, 52 mg, 0.2 mmol, 0.1 eq.) and bromoanilide **12b** (541 mg, 1.0 mmol) in anhydrous toluene (20 mL) was degassed and purged with argon (3 times). To this mixture, a solution of lithium bis(trimethylsilyl)amide (1.0 M in THF, 2 mL, 2 mmol, 2.0 eq.) was added and the resulting mixture was stirred at 80 °C (oil bath) under argon for 6 h. After cooling to room temperature then 0 °C, powdered NH_4Cl (107 mg, 2 mmol) was added. After stirring at 0 °C for 60 min, the mixture was diluted with water (5 mL). The aqueous phase was extracted with ethyl acetate (3 × 30 mL). The combined organic phases were dried over anhydrous Na_2SO_4. After removal of the solvents, the residue was chromatographed on silica gel (Petroleum ether 60-90 ºC: ethyl acetate = 2:1) to afford the major product (**19a**, 280 mg, 61%) as pale yellow syrup, Further elution afforded the minor product (**19**, 133 mg, 28%) as a yellow solid.

19a: R_f: 0.54 (Petroleum ether: ethyl acetate = 2:1). **^{1}H-NMR** (as a mixture of rotamers, 300 MHz, $CDCl_3$), δ (ppm): 7.39-6.87 (13H, m), 6.67 (1H, *d, J* = 7.8 Hz), 4.87 (1H, *d, J* = 15.6 Hz), 4.80 (1H, *d, J* = 15.6 Hz), 4.39-4.07 (2H, *m*), 3.45 (1H, *t*, *J* = 6.0 Hz), 3.31-3.10 (1H, *m*), 3.09-2.82 (1H, *m*), 2.33-2.04 (1H, *m*), 1.22 (4.5H, *s*), 1.20 (4.5H, *s*). **^{13}C-NMR** ((rotamer in brackets, 75 MHz, $CDCl_3$), δ (ppm):177.08, 143.26, 137.12, 135.85, 128.79, 128.63, 128.02, 127.62, 127.54, 127.30, 123.75, 122.48, 109.08, 58.46 (58.30), 51.34, 45.86 (45.29), 43.75, 43.34, 29.48 (29.30), 23.48 (23.40). **HRMS** *m/z* Found: 460.2168, Calcd. for $C_{28}H_{32}N_2NaO_3S$ $(M)^+$: 460.2200.

Synthesis of compound **19**: A solution of amine **19a** (184 mg, 0.4 mmol) in anhydrous toluene (5 mL) was degassed and purged with argon (3 times). To this solution, lithium bis(trimethylsilyl)amide (1.0 M in THF, 0.48 mL, 0.48

mmol, 1.2 eq.) was added and the resulting mixture was stirred at 0 °C under argon for 0.5 h. A solution of anhydrous *t*-BuOOH (~3.0 M in toluene, 0.16 mL, 0.48 mmol, 1.2 eq.) was added and the reaction mixture was stirred at 0 °C under argon for another 0.5 h. Saturated aqueous solution of NH_4Cl (1 mL) was then added. After 10 min, the resulting mixture was diluted with water (5 mL) and extracted with ethyl acetate (3 × 5 mL). After removal of the solvents, the residue was chromatographed on silica gel (Petroleum ether 60-90 ºC: ethyl acetate = 2:1) to afford the product (**19**, 166 mg, 87%) as a yellow solid.

19: m.p.: 125-127 ºC. R_f: 0.54 (Petroleum ether: ethyl acetate = 1:1). **FTIR** (KBr, thin film) cm^{-1}: 3321, 3057, 2958, 2866, 1722, 1611, 1460, 1359, 1273, 1174, 1068, 928, 743, 704, 633, 596, 464. **^{1}H-NMR** (as a mixture of rotamers, 400 MHz, $CDCl_3$), δ (ppm): 7.30-7.11 (12H, *m*), 7.08 (0.5H, *t*, *J* = 7.6 Hz), 7.07 (0.5H, *t*, *J* = 7.6 Hz), 6.92 (0.5H, *t*, *J* = 7.6 Hz), 6.91 (0.5H, *t*, *J* = 7.6 Hz), 6.61 (0.5H, *d*, *J* = 7.6 Hz), 6.60 (0.5H, *d*, *J* = 7.6 Hz), 5.07 (0.5H, *s*), 5.04 (0.5H, *s*), 4.86 (0.5H, *d*, *J* = 15.6 Hz), 4.83 (0.5H, *d*, *J* = 15.6 Hz), 4.61 (0.5H, *d*, *J* = 15.6 Hz), 4.59 (0.5H, *d*, *J* = 15.6 Hz), 4.19 (0.5H, *d*, *J* = 15.2 Hz), 4.17 (0.5H, *d*, *J* = 15.2 Hz), 4.02 (0.5H, *d*, *J* = 15.2 Hz), 4.01 (0.5H, *d*, *J* = 15.2 Hz), 3.10-2.95 (1H, *m*), 2.93-2.70 (1H, *m*), 2.38-2.18 (2H, *m*), 1.10 (4.5H, *s*), 1.09 (4.5H, *s*). **^{13}C-NMR** (rotamer in brackets, 100 MHz, $CDCl_3$), δ (ppm): 177.54, 141.86, 136.62 (136.56), 135.29, 129.86 (129.80), 129.21, 128.60, 128.36 (128.30), 127.41 (127.20), 127.03 (127.00), 123.73 (123.68), 122.88, 109.24, 74.75 (74.71), 57.97, 51.24, 43.46, 42.72, 36.55 (36.43), 23.09. **HRMS** *m/z* Found: 499.2028, Calcd. for $C_{28}H_{32}N_2NaO_3S$ $(M+Na)^+$: 499.2031.

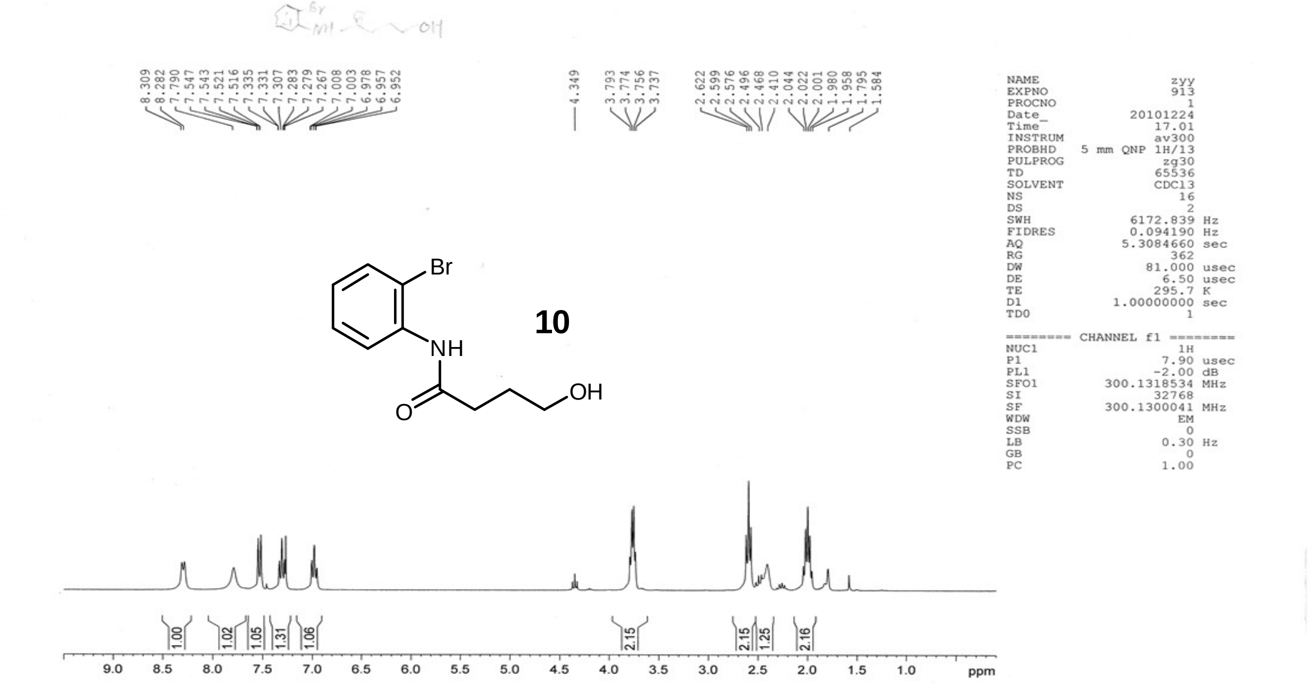
8.309
8.282
7.790
7.547
7.543
7.521
7.516
7.335
7.331
7.307
7.283
7.279
7.267
7.008
7.003
6.978
6.957
6.952
4.349
3.793
3.774
3.756
3.737
2.622
2.599
2.576
2.496
2.468
2.410
2.044
2.022
2.001
1.980
1.958
1.795
1.584
Br
NH
O
OH
10
NAME zyy
EXPNO 913
PROCNO 1
Date_ 20101224
Time 17.01
INSTRUM av300
PROBHD 5 mm QNP 1H/13
PULPROG zg30
TD 65536
SOLVENT CDCl3
NS 16
DS 2
SWH 6172.839 Hz
FIDRES 0.094190 Hz
AQ 5.3084660 sec
RG 362
DW 81.000 usec
DE 6.50 usec
TE 295.7 K
D1 1.00000000 sec
TD0 1
======== CHANNEL f1 ========
NUC1 1H
P1 7.90 usec
PL1 -2.00 dB
SFO1 300.1318534 MHz
SI 32768
SF 300.1300041 MHz
WDW EM
SSB 0
LB 0.30 Hz
GB 0
PC 1.00
1.00
1.02
1.05
1.31
1.06
2.15
2.15
1.25
2.16
9.0 8.5 8.0 7.5 7.0 6.5 6.0 5.5 5.0 4.5 4.0 3.5 3.0 2.5 2.0 1.5 1.0 ppm

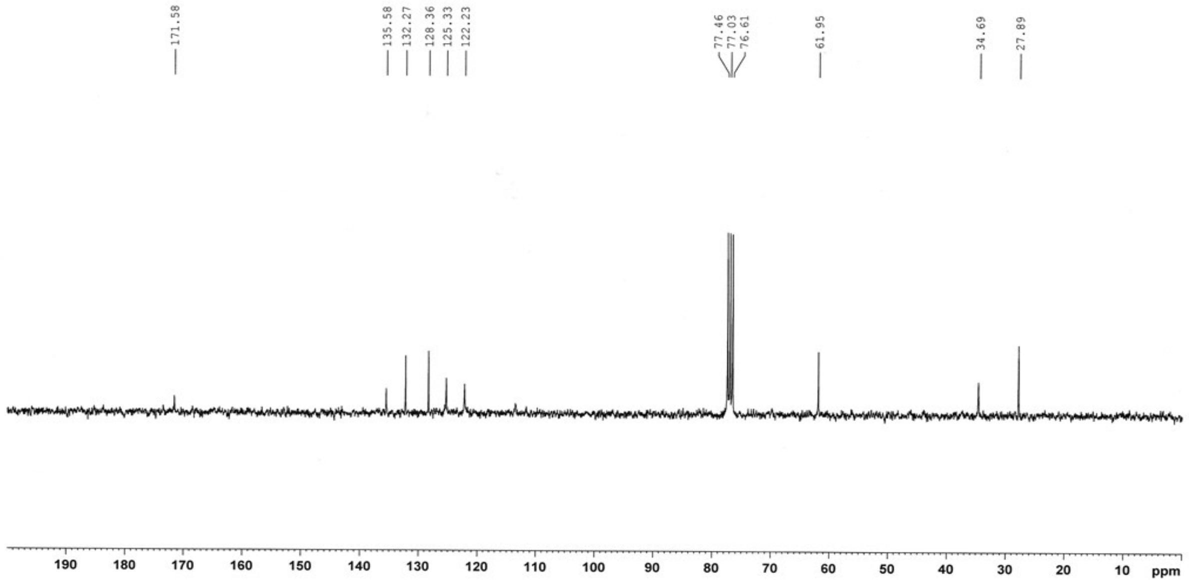
171.58
135.58
132.27
128.36
125.33
122.23
77.46
77.03
76.61
61.95
34.69
27.89
190 180 170 160 150 140 130 120 110 100 90 80 70 60 50 40 30 20 10 ppm

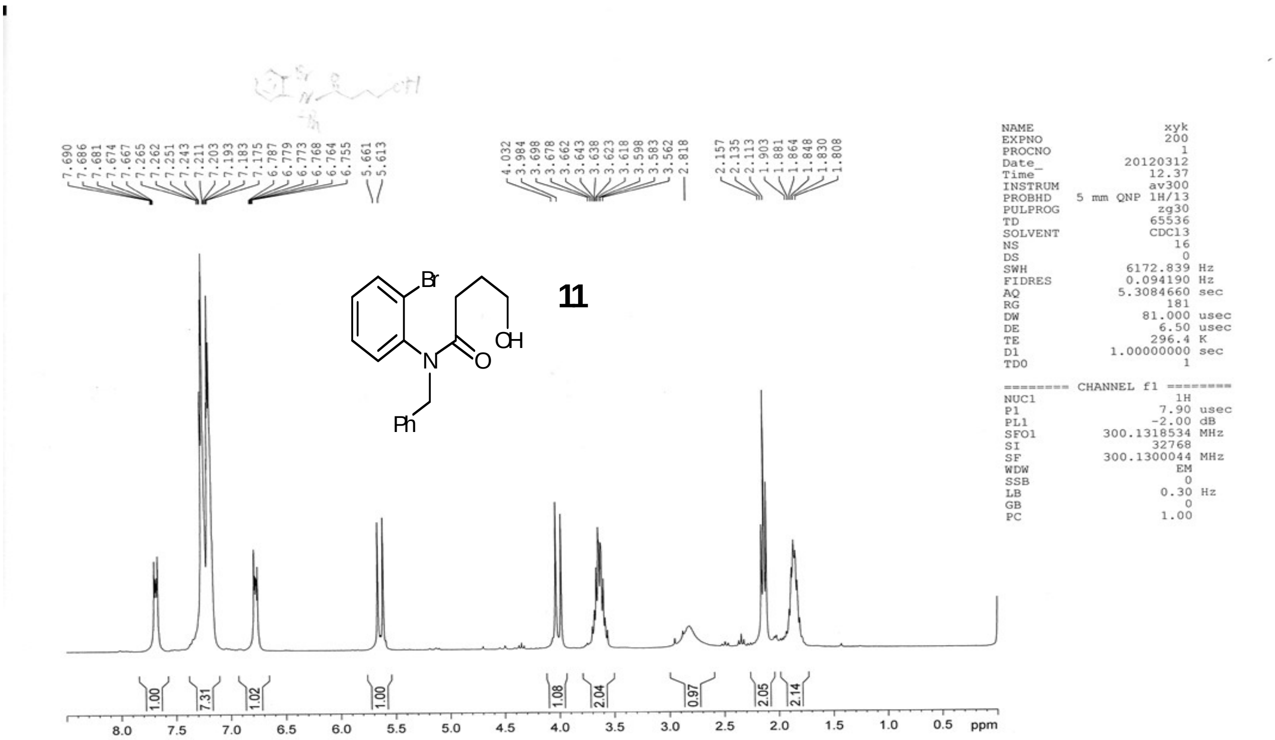

7.690 7.686 7.681 7.674 7.667 7.265 7.262 7.251 7.243 7.211 7.203 7.193 7.183 7.175 6.787 6.779 6.773 6.768 6.764 6.755 5.661 5.613
4.032 3.984 3.698 3.678 3.662 3.643 3.638 3.623 3.618 3.598 3.583 3.562 2.818
2.157 2.135 2.113 1.903 1.881 1.864 1.848 1.830 1.808
Br
N
O
OH
Ph
11
NAME xyk
EXPNO 200
PROCNO 1
Date_ 20120312
Time 12.37
INSTRUM av300
PROBHD 5 mm QNP 1H/13
PULPROG zg30
TD 65536
SOLVENT CDCl3
NS 16
DS 0
SWH 6172.839 Hz
FIDRES 0.094190 Hz
AQ 5.3084660 sec
RG 181
DW 81.000 usec
DE 6.50 usec
TE 296.4 K
D1 1.00000000 sec
TD0 1
======== CHANNEL f1 ========
NUC1 1H
P1 7.90 usec
PL1 -2.00 dB
SFO1 300.1318534 MHz
SI 32768
SF 300.1300044 MHz
WDW EM
SSB 0
LB 0.30 Hz
GB 0
PC 1.00
1.00 7.31 1.02 1.00 1.08 2.04 0.97 2.05 2.14
8.0 7.5 7.0 6.5 6.0 5.5 5.0 4.5 4.0 3.5 3.0 2.5 2.0 1.5 1.0 0.5 ppm

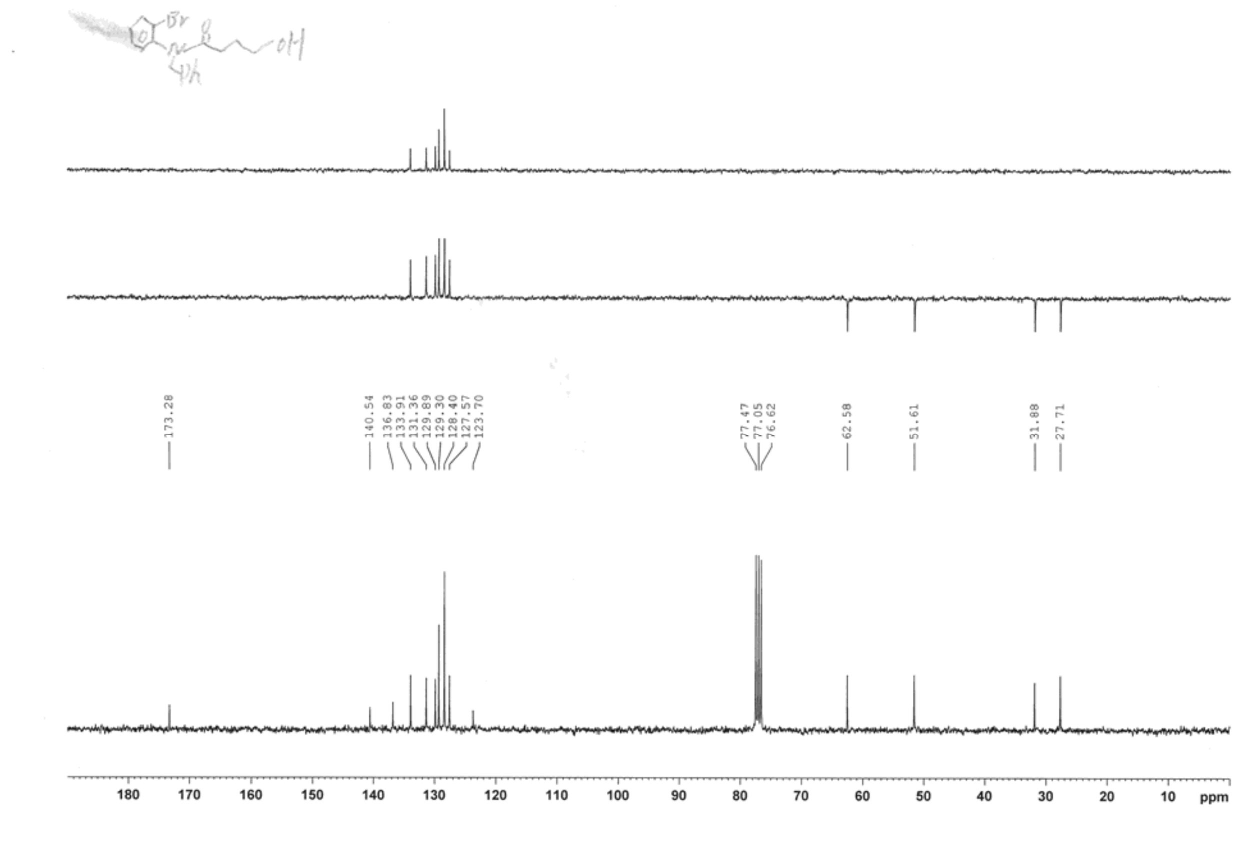

173.28
140.54 136.83 133.91 131.36 129.89 129.30 128.40 127.57 123.70
77.47 77.05 76.62
62.58
51.61
31.88 27.71
180 170 160 150 140 130 120 110 100 90 80 70 60 50 40 30 20 10 ppm

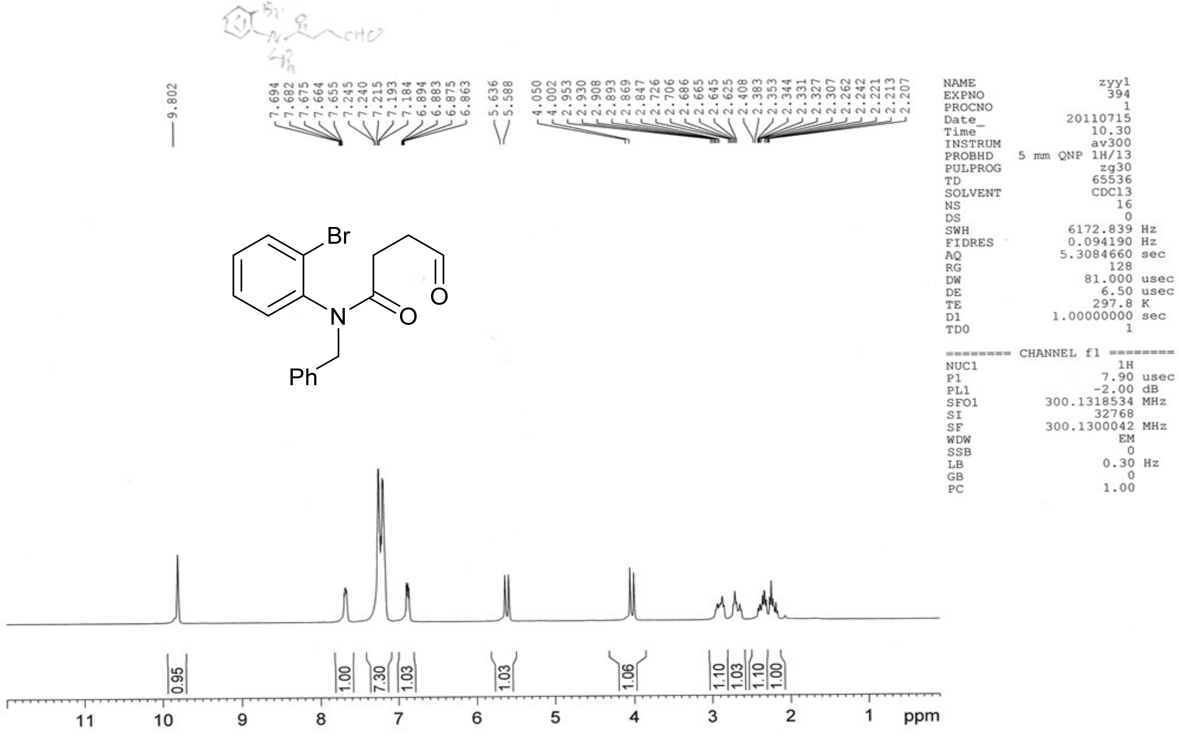
9.802
7.694 7.682 7.675 7.664 7.655 7.245 7.240 7.215 7.193 7.184 6.894 6.883 6.875 6.863
5.636 5.588
4.050 4.002 2.953 2.930 2.908 2.893 2.869 2.847 2.726 2.706 2.686 2.665 2.645 2.625 2.408 2.383 2.353 2.344 2.331 2.327 2.307 2.262 2.242 2.221 2.213 2.207
Br
N
O
O
Ph
NAME zyy1
EXPNO 394
PROCNO 1
Date_ 20110715
Time 10.30
INSTRUM av300
PROBHD 5 mm QNP 1H/13
PULPROG zg30
TD 65536
SOLVENT CDCl3
NS 16
DS 0
SWH 6172.839 Hz
FIDRES 0.094190 Hz
AQ 5.3084660 sec
RG 128
DW 81.000 usec
DE 6.50 usec
TE 297.8 K
D1 1.00000000 sec
TD0 1
======== CHANNEL f1 ========
NUC1 1H
P1 7.90 usec
PL1 -2.00 dB
SFO1 300.1318534 MHz
SI 32768
SF 300.1300042 MHz
WDW EM
SSB 0
LB 0.30 Hz
GB 0
PC 1.00
0.95 1.00 7.30 1.03 1.03 1.06 1.10 1.03 1.10 1.00
11 10 9 8 7 6 5 4 3 2 1 ppm

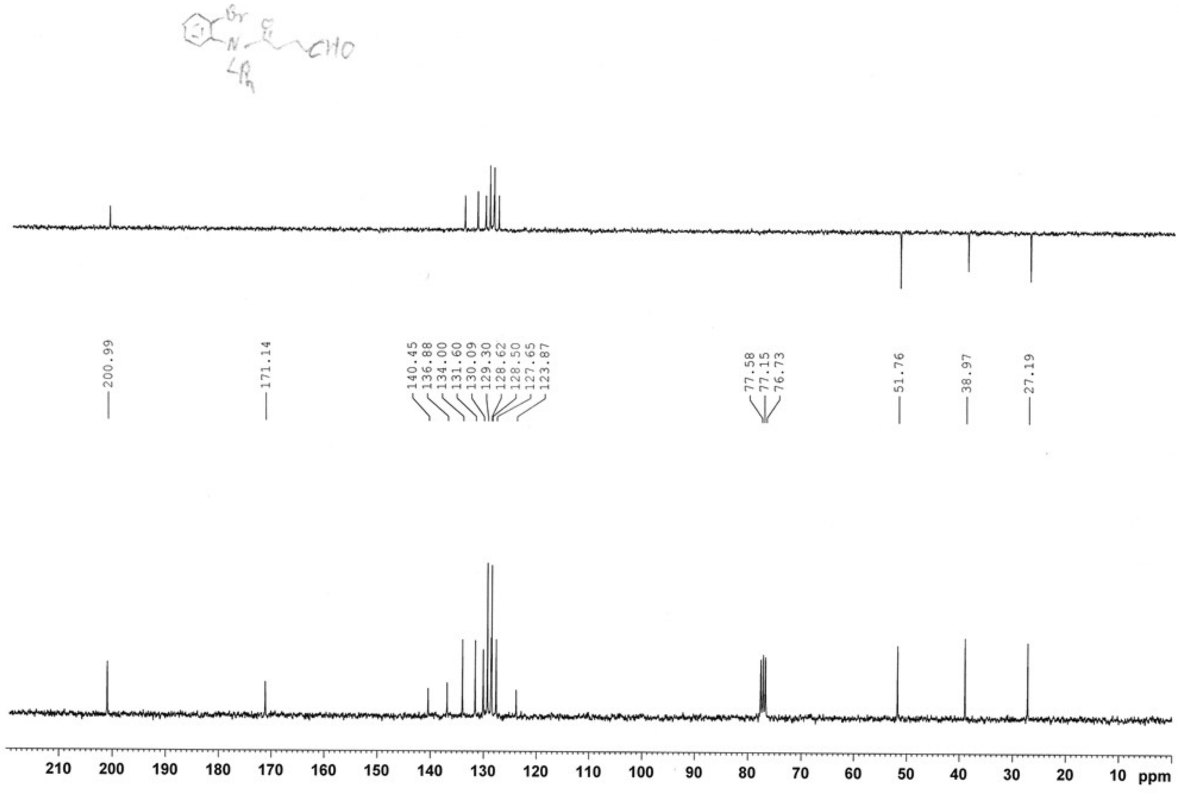
200.99
171.14
140.45 136.88 134.00 131.60 130.09 129.30 128.62 128.50 127.65 123.87
77.58 77.15 76.73
51.76
38.97
27.19
210 200 190 180 170 160 150 140 130 120 110 100 90 80 70 60 50 40 30 20 10 ppm

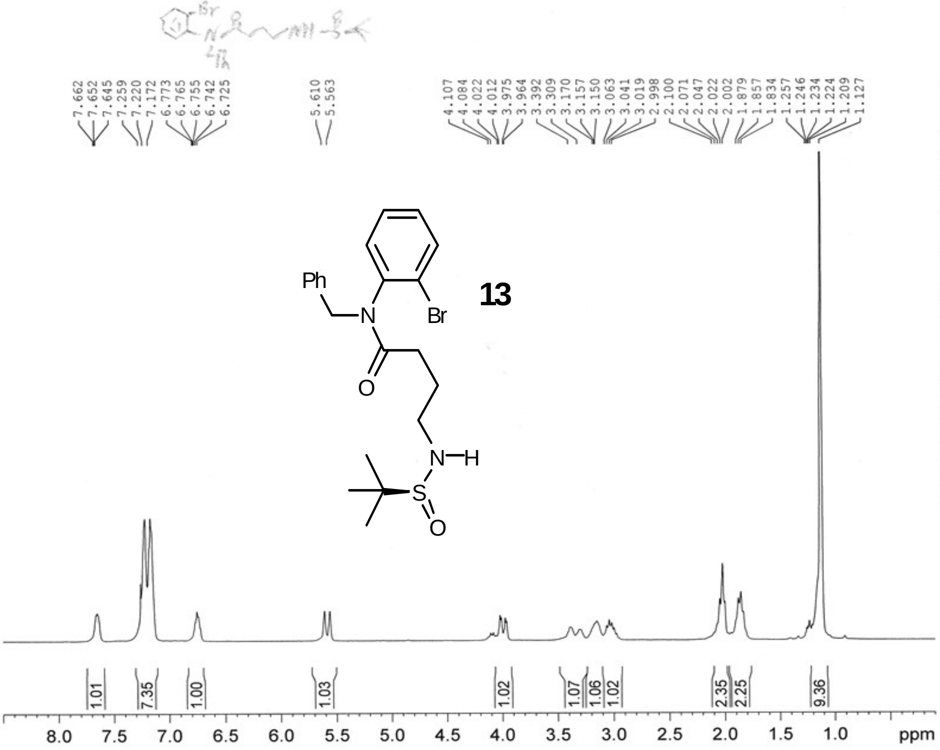

NAME	zyyl
EXPNO	29
PROCNO	1
Date_	20110228
Time	16.48
INSTRUM	av300
PROBHD	5 mm QNP 1H/13
PULPROG	zg30
TD	65536
SOLVENT	CDCl3
NS	16
DS	2
SWH	6172.839 Hz
FIDRES	0.094190 Hz
AQ	5.3084660 sec
RG	128
DW	81.000 usec
DE	6.50 usec
TE	295.9 K
D1	1.00000000 sec
TD0	1

======== CHANNEL f1 ========

NUC1	1H
P1	7.90 usec
PL1	-2.00 dB
SFO1	300.1318534 MHz
SI	32768
SF	300.1300065 MHz
WDW	EM
SSB	0
LB	0.30 Hz
GB	0
PC	1.00

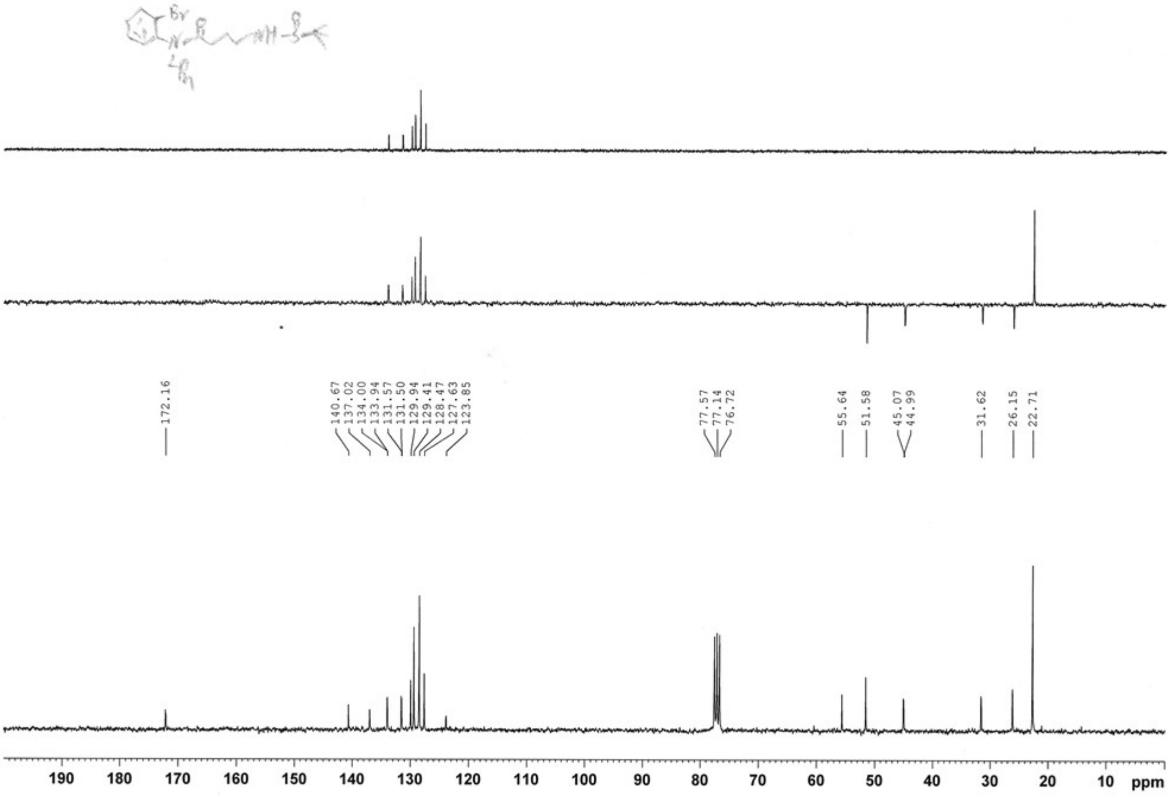

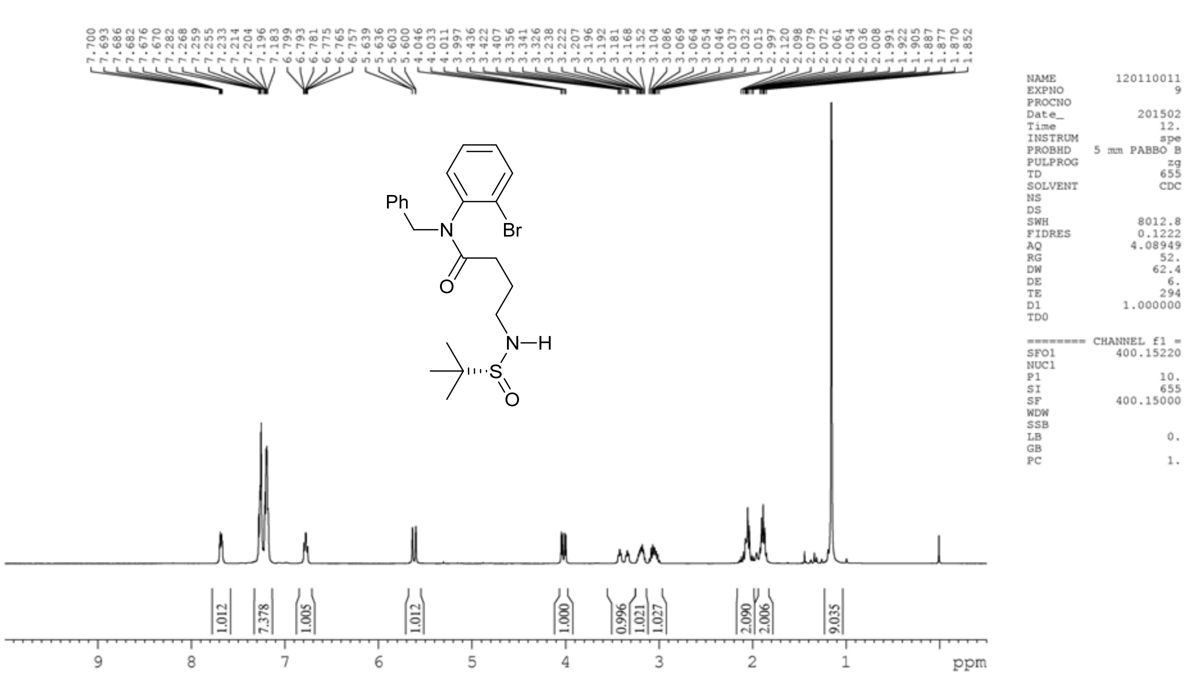
Ph
N
Br
O
N—H
S
O
NAME 120110011
EXPNO 9
PROCNO
Date_ 201502
Time 12.
INSTRUM spe
PROBHD 5 mm PABBO B
PULPROG zg
TD 655
SOLVENT CDC
NS
DS
SWH 8012.8
FIDRES 0.1222
AQ 4.08949
RG 52.
DW 62.4
DE 6.
TE 294
D1 1.000000
TD0
======== CHANNEL f1 =
SFO1 400.15220
NUC1
P1 10.
SI 655
SF 400.15000
WDW
SSB
LB 0.
GB
PC 1.
1.012
7.378
1.005
1.012
1.000
0.996
1.021
1.027
2.090
2.006
9.035
ppm

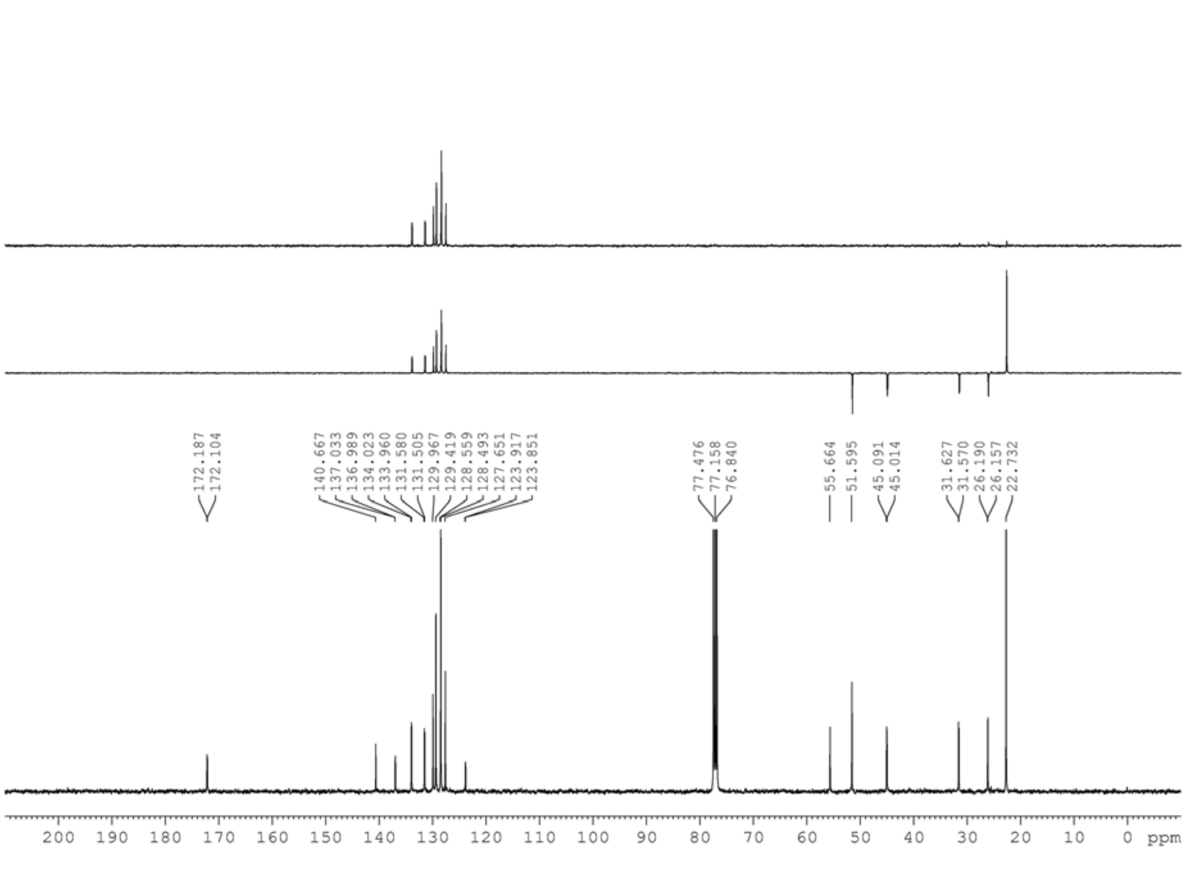
172.187
172.104
140.667
137.033
136.989
134.023
133.960
131.580
131.505
129.967
129.419
128.559
128.493
127.651
123.917
123.851
77.476
77.158
76.840
55.664
51.595
45.091
45.014
31.627
31.570
26.190
26.157
22.732
ppm

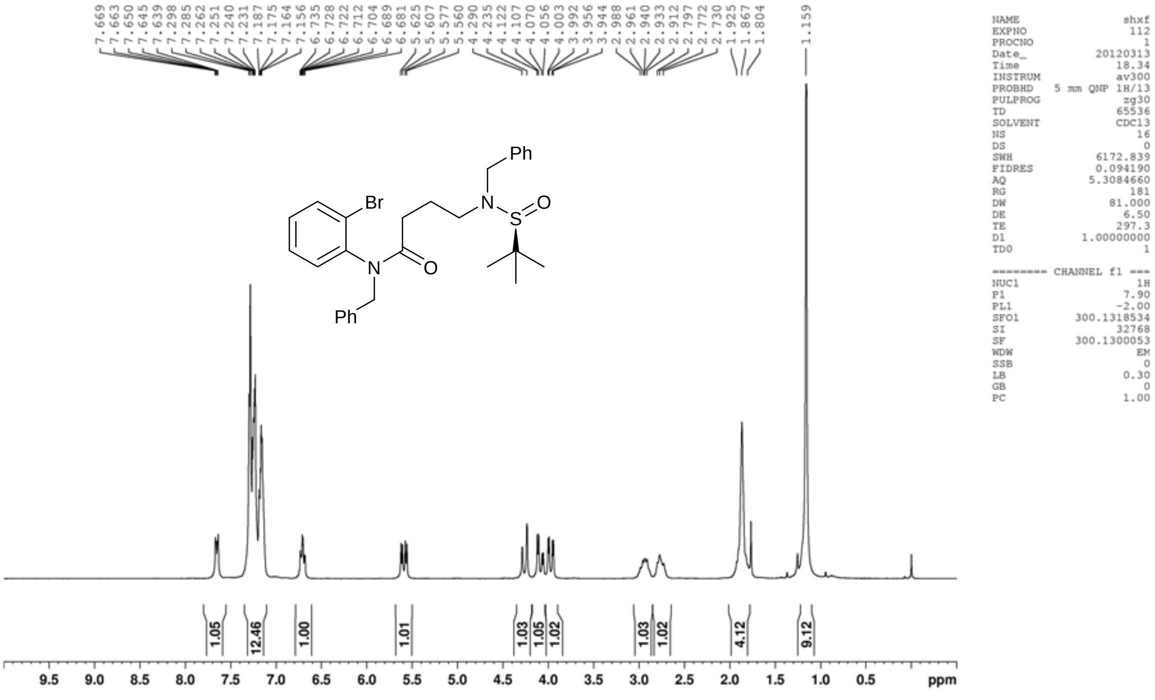
7.669 7.663 7.650 7.645 7.639 7.298 7.285 7.262 7.251 7.240 7.231 7.187 7.175 7.164 7.156 6.735 6.728 6.722 6.712 6.704 6.689 6.681 5.625 5.607 5.577 5.560 4.290 4.235 4.122 4.107 4.070 4.056 4.003 3.992 3.956 3.944 2.988 2.961 2.940 2.933 2.912 2.797 2.772 2.730 1.925 1.867 1.804 1.159
Br
Ph
N
O
S
Ph
1.05 12.46 1.00 1.01 1.03 1.05 1.02 1.03 1.02 4.12 9.12
9.5 9.0 8.5 8.0 7.5 7.0 6.5 6.0 5.5 5.0 4.5 4.0 3.5 3.0 2.5 2.0 1.5 1.0 0.5 ppm
NAME shxf
EXPNO 112
PROCNO 1
Date_ 20120313
Time 18.34
INSTRUM av300
PROBHD 5 mm QNP 1H/13
PULPROG zg30
TD 65536
SOLVENT CDCl3
NS 16
DS 0
SWH 6172.839
FIDRES 0.094190
AQ 5.3084660
RG 181
DW 81.000
DE 6.50
TE 297.3
D1 1.00000000
TD0 1
======== CHANNEL f1 ===
NUC1 1H
P1 7.90
PL1 -2.00
SFO1 300.1318534
SI 32768
SF 300.1300053
WDW EM
SSB 0
LB 0.30
GB 0
PC 1.00

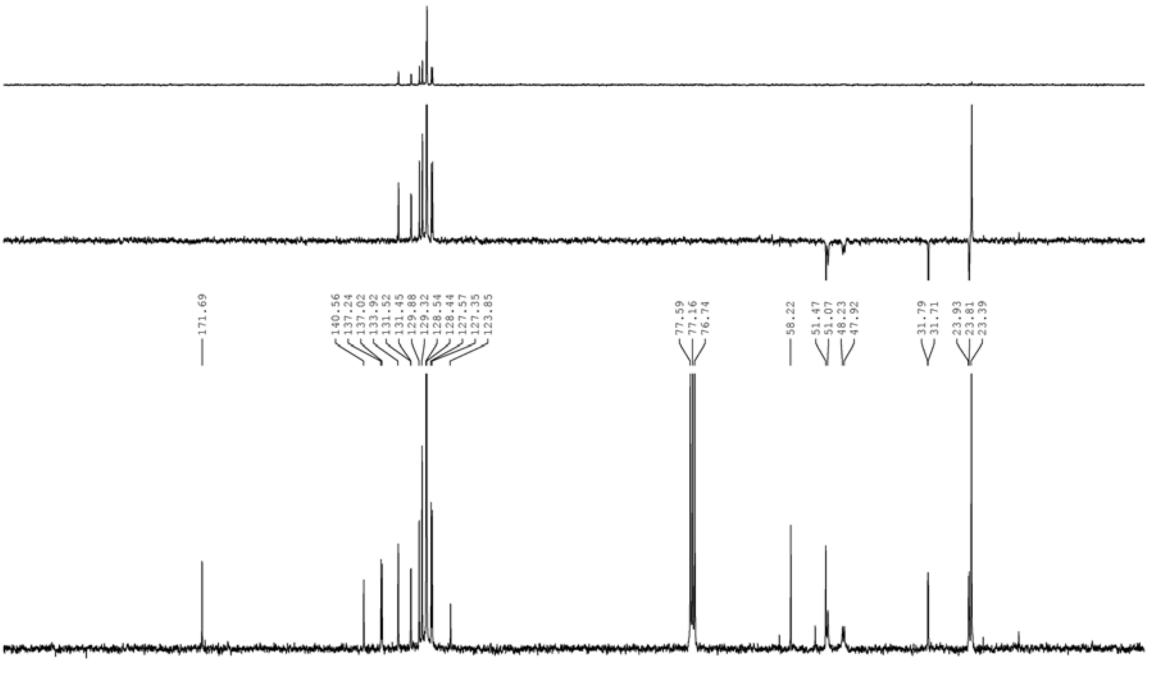
171.69
140.56 137.24 137.02 133.92 131.52 131.45 129.88 129.32 128.54 128.44 127.57 127.35 123.85
77.59 77.16 76.74
58.22
51.47 51.07 48.23 47.92
31.79 31.71
23.93 23.81 23.39
200 190 180 170 160 150 140 130 120 110 100 90 80 70 60 50 40 30 20 10 0 ppm

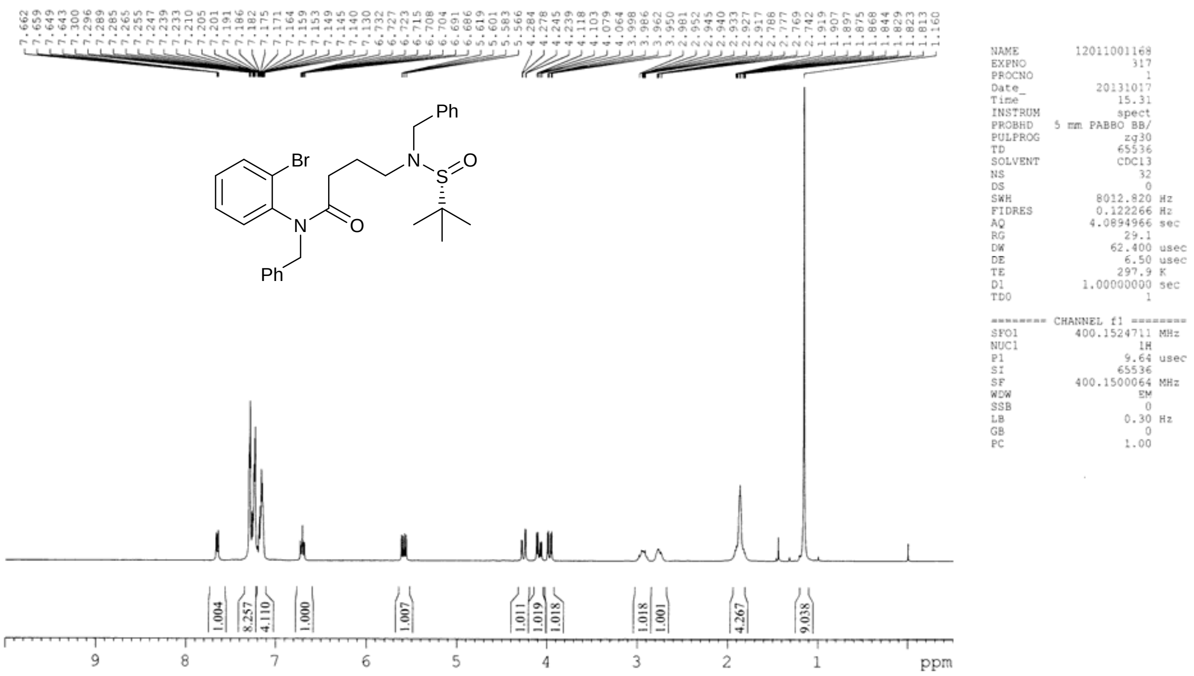
7.662 7.659 7.649 7.643 7.300 7.296 7.289 7.285 7.265 7.255 7.247 7.239 7.233 7.210 7.205 7.201 7.191 7.186 7.182 7.175 7.171 7.164 7.159 7.153 7.149 7.145 7.140 7.130 6.732 6.727 6.723 6.715 6.708 6.704 6.691 6.686 5.619 5.601 5.583 5.566 4.284 4.278 4.245 4.239 4.118 4.103 4.079 4.064 3.998 3.986 3.962 3.950 2.981 2.952 2.945 2.940 2.933 2.927 2.917 2.788 2.777 2.769 2.742 1.919 1.907 1.897 1.875 1.868 1.844 1.829 1.823 1.813 1.160
Br
N
O
Ph
N
S
O
Ph
1.004 8.257 4.110 1.000 1.007 1.011 1.019 1.018 1.018 1.001 4.267 9.038
9 8 7 6 5 4 3 2 1 ppm
NAME 12011001168
EXPNO 317
PROCNO 1
Date_ 20131017
Time 15.31
INSTRUM spect
PROBHD 5 mm PABBO BB/
PULPROG zg30
TD 65536
SOLVENT CDCl3
NS 32
DS 0
SWH 8012.820 Hz
FIDRES 0.122266 Hz
AQ 4.0894966 sec
RG 29.1
DW 62.400 usec
DE 6.50 usec
TE 297.9 K
D1 1.00000000 sec
TD0 1
======== CHANNEL f1 ========
SFO1 400.1524711 MHz
NUC1 1H
P1 9.64 usec
SI 65536
SF 400.1500064 MHz
WDW EM
SSB 0
LB 0.30 Hz
GB 0
PC 1.00

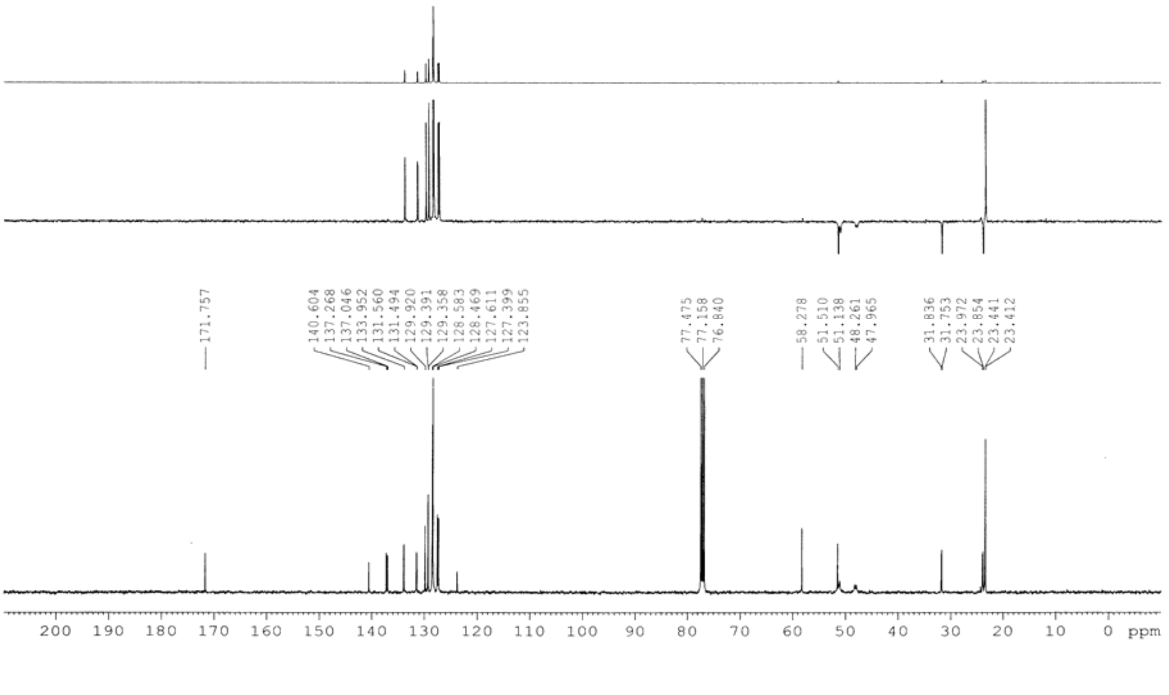
171.757
140.604 137.268 137.046 133.952 131.560 131.494 129.920 129.391 129.358 128.583 128.469 127.611 127.399 123.855
77.475 77.158 76.840
58.278 51.510 51.138 48.261 47.965
31.836 31.753 23.972 23.854 23.441 23.412
200 190 180 170 160 150 140 130 120 110 100 90 80 70 60 50 40 30 20 10 0 ppm

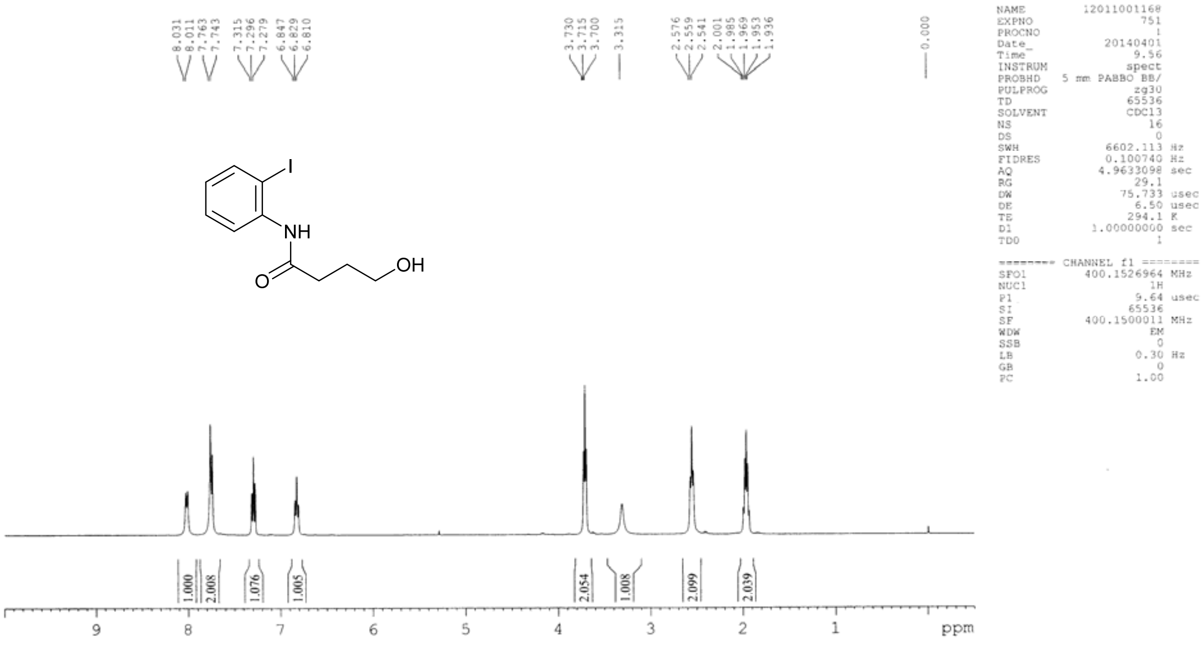

8.031
8.011
7.763
7.743
7.315
7.296
7.279
6.847
6.829
6.810
3.730
3.715
3.700
3.315
2.576
2.559
2.541
2.001
1.985
1.969
1.953
1.936
0.000
I
NH
O
OH
NAME 12011001168
EXPNO 751
PROCNO 1
Date_ 20140401
Time 9.56
INSTRUM spect
PROBHD 5 mm PABBO BB/
PULPROG zg30
TD 65536
SOLVENT CDCl3
NS 16
DS 0
SWH 6602.113 Hz
FIDRES 0.100740 Hz
AQ 4.9633098 sec
RG 29.1
DW 75.733 usec
DE 6.50 usec
TE 294.1 K
D1 1.00000000 sec
TD0 1
======== CHANNEL f1 ========
SFO1 400.1526964 MHz
NUC1 1H
P1 9.64 usec
SI 65536
SF 400.1500011 MHz
WDW EM
SSB 0
LB 0.30 Hz
GB 0
PC 1.00
1.000
2.008
1.076
1.005
2.054
1.008
2.099
2.039
9
8
7
6
5
4
3
2
1
ppm

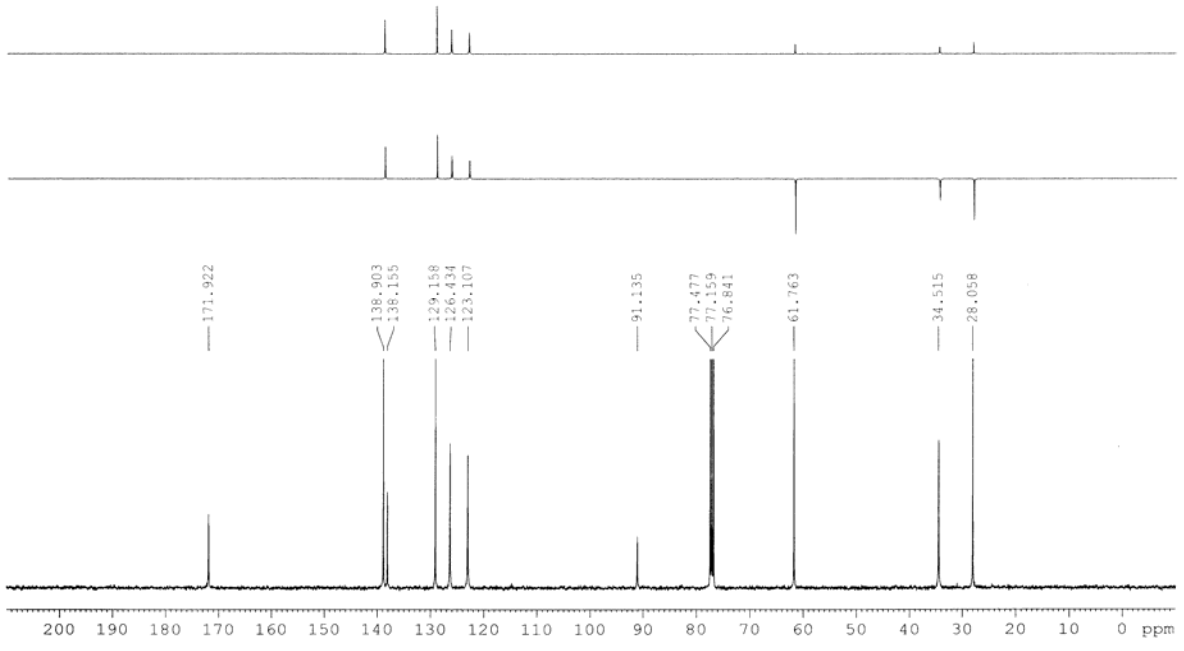

171.922
138.903
138.155
129.158
126.434
123.107
91.135
77.477
77.159
76.841
61.763
34.515
28.058
200
190
180
170
160
150
140
130
120
110
100
90
80
70
60
50
40
30
20
10
0
ppm

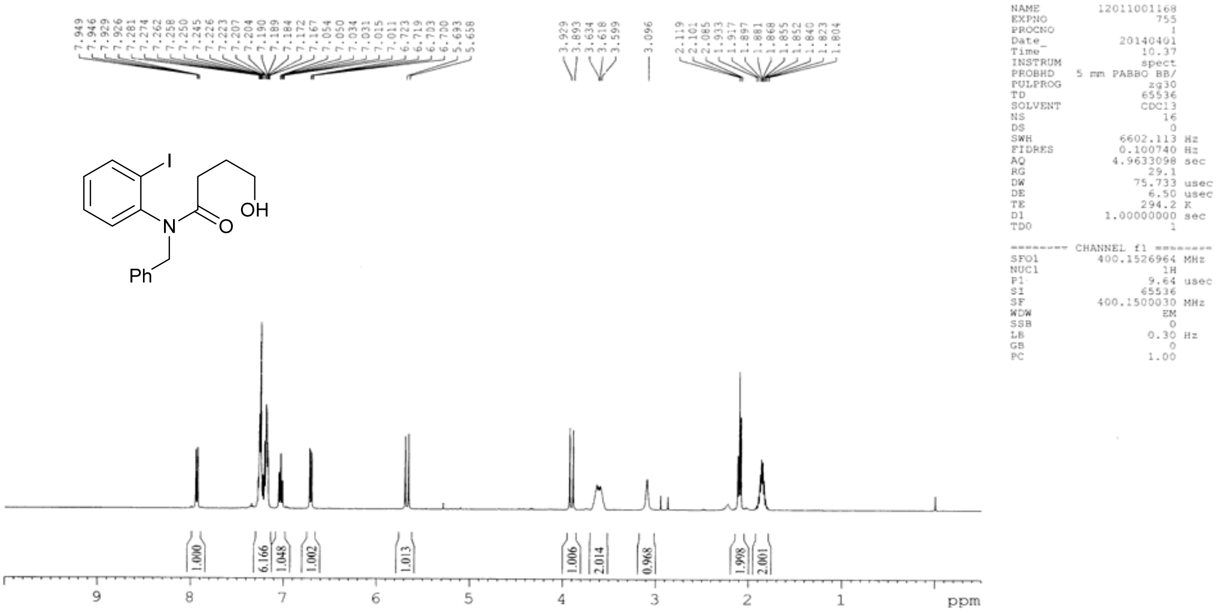
7.949
7.946
7.929
7.926
7.281
7.274
7.262
7.258
7.250
7.245
7.226
7.223
7.207
7.204
7.190
7.189
7.184
7.172
7.167
7.054
7.050
7.034
7.031
7.015
7.011
6.723
6.719
6.703
6.700
5.693
5.658
3.929
3.893
3.634
3.618
3.599
3.096
2.119
2.101
2.085
1.933
1.917
1.897
1.881
1.868
1.855
1.852
1.840
1.823
1.804
I
N
O
OH
Ph
1.000
6.166
1.048
1.002
1.013
1.006
2.014
0.968
1.998
2.001
9
8
7
6
5
4
3
2
1
ppm
NAME 12011001168
EXPNO 755
PROCNO 1
Date_ 20140401
Time 10.37
INSTRUM spect
PROBHD 5 mm PABBO BB/
PULPROG zg30
TD 65536
SOLVENT CDCl3
NS 16
DS 0
SWH 6602.113 Hz
FIDRES 0.100740 Hz
AQ 4.9633098 sec
RG 29.1
DW 75.733 usec
DE 6.50 usec
TE 294.2 K
D1 1.00000000 sec
TD0 1
======== CHANNEL f1 ========
SFO1 400.1526964 MHz
NUC1 1H
P1 9.64 usec
SI 65536
SF 400.1500030 MHz
WDW EM
SSB 0
LB 0.30 Hz
GB 0
PC 1.00

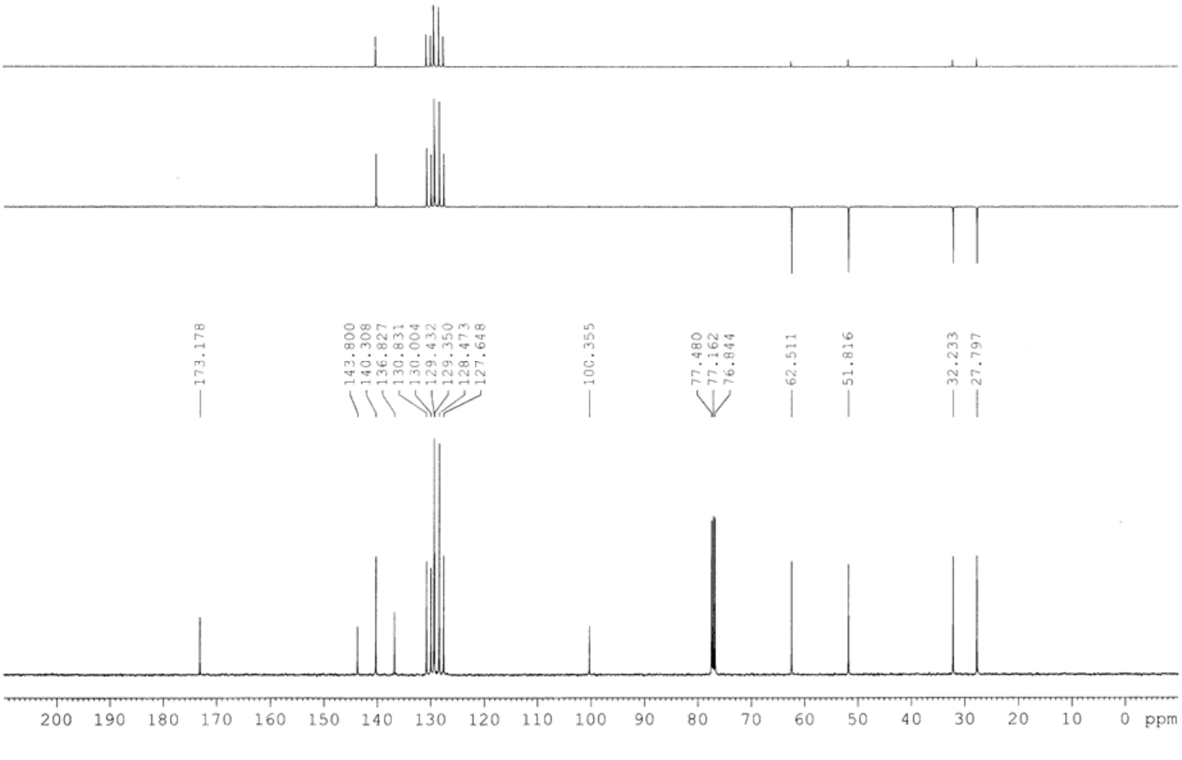
173.178
143.800
140.308
136.827
130.831
130.004
129.432
129.350
128.473
127.648
100.355
77.480
77.162
76.844
62.511
51.816
32.233
27.797
200
190
180
170
160
150
140
130
120
110
100
90
80
70
60
50
40
30
20
10
0
ppm

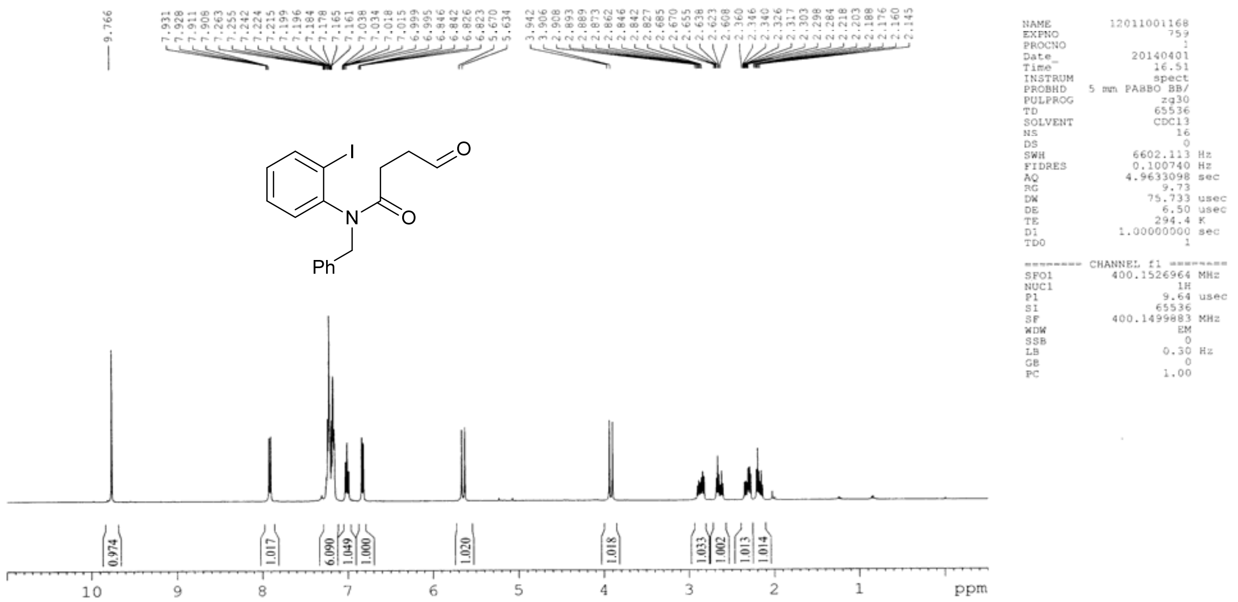
9.766
7.931 7.928 7.911 7.908 7.263 7.255 7.242 7.224 7.215 7.199 7.196 7.184 7.178 7.165 7.161 7.038 7.034 7.018 7.015 6.999 6.995 6.846 6.842 6.826 6.823 5.670 5.634
3.942 3.906 2.908 2.893 2.889 2.873 2.862 2.846 2.842 2.827 2.685 2.670 2.655 2.638 2.623 2.608 2.360 2.346 2.340 2.326 2.317 2.303 2.298 2.284 2.218 2.203 2.188 2.176 2.160 2.145
I
O
N
O
Ph
0.974 1.017 6.090 1.049 1.000 1.020 1.018 1.033 1.002 1.013 1.014
10 9 8 7 6 5 4 3 2 1 ppm
NAME 12011001168
EXPNO 759
PROCNO 1
Date_ 20140401
Time 16.51
INSTRUM spect
PROBHD 5 mm PABBO BB/
PULPROG zg30
TD 65536
SOLVENT CDCl3
NS 16
DS 0
SWH 6602.113 Hz
FIDRES 0.100740 Hz
AQ 4.9633098 sec
RG 9.73
DW 75.733 usec
DE 6.50 usec
TE 294.4 K
D1 1.00000000 sec
TD0 1
======== CHANNEL f1 ========
SFO1 400.1526964 MHz
NUC1 1H
P1 9.64 usec
SI 65536
SF 400.1499883 MHz
WDW EM
SSB 0
LB 0.30 Hz
GB 0
PC 1.00

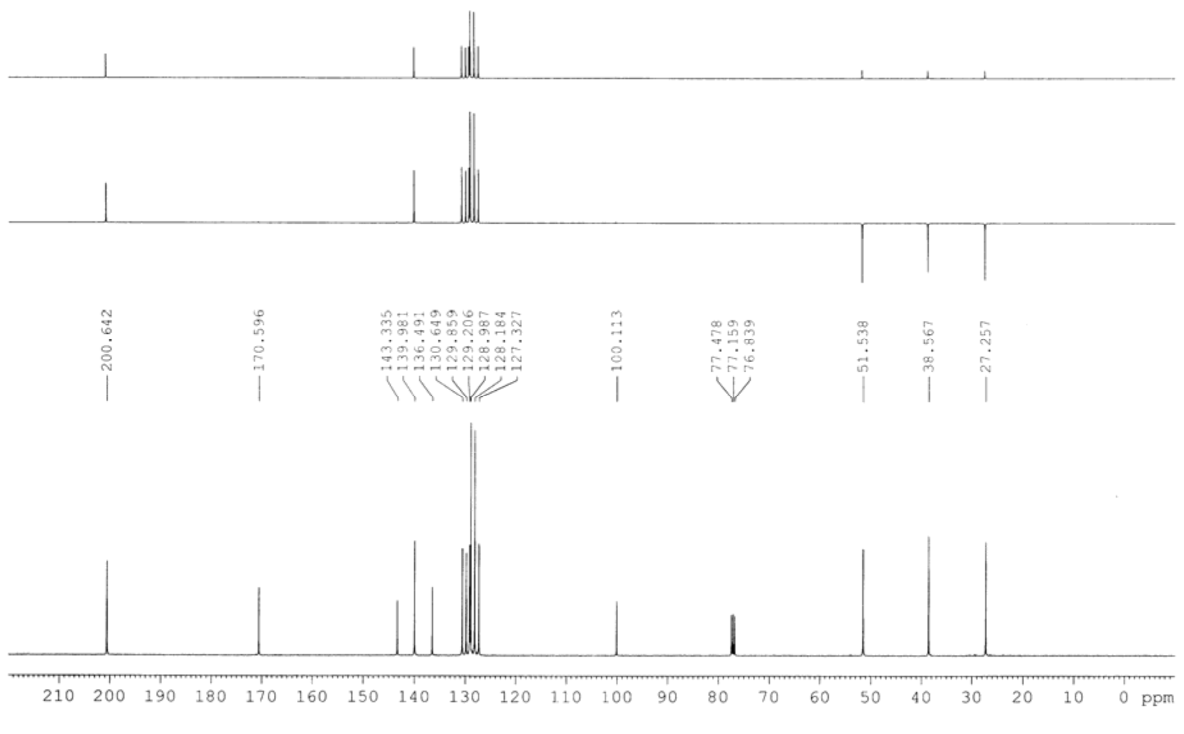
200.642
170.596
143.335 139.981 136.491 130.649 129.859 129.206 128.987 128.184 127.327
100.113
77.478 77.159 76.839
51.538
38.567
27.257
210 200 190 180 170 160 150 140 130 120 110 100 90 80 70 60 50 40 30 20 10 0 ppm

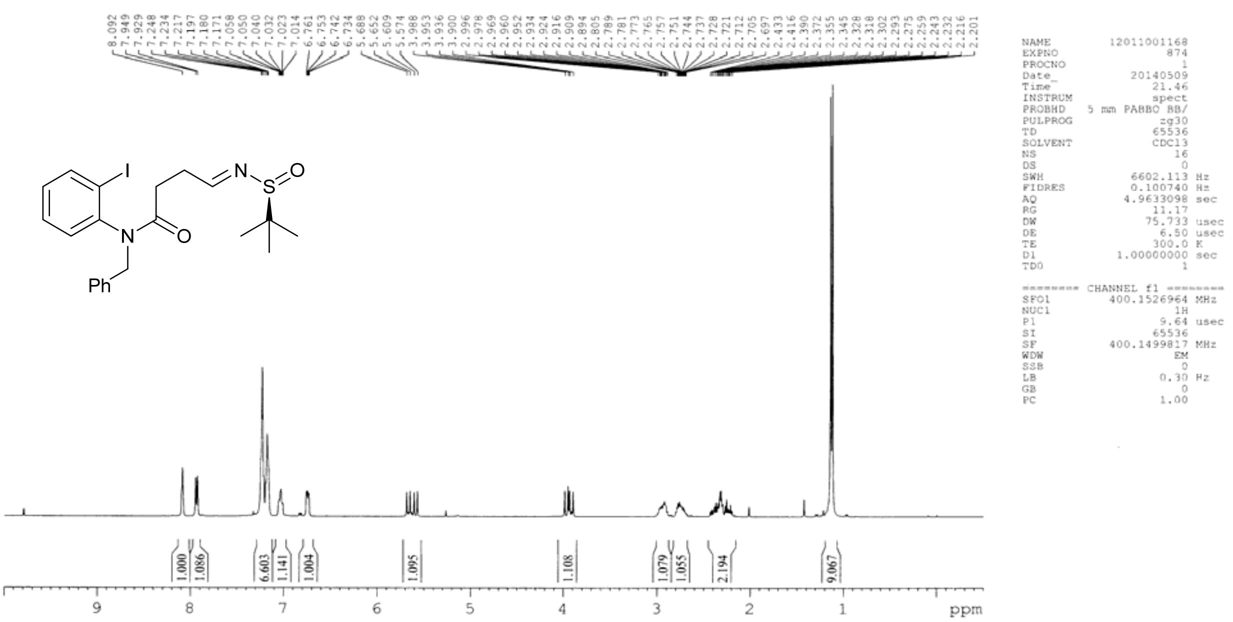
8.092 7.949 7.929 7.248 7.234 7.217 7.197 7.180 7.171 7.058 7.050 7.040 7.032 7.023 7.014 6.761 6.753 6.742 6.734 5.688 5.652 5.609 5.574 3.988 3.953 3.936 3.900 2.996 2.978 2.969 2.960 2.952 2.934 2.924 2.916 2.909 2.894 2.805 2.789 2.781 2.773 2.765 2.757 2.751 2.744 2.737 2.728 2.721 2.712 2.705 2.697 2.433 2.416 2.390 2.372 2.355 2.345 2.328 2.318 2.302 2.293 2.275 2.259 2.243 2.232 2.216 2.201
I
N
S
O
O
N
Ph
1.000 1.086 6.603 1.141 1.004 1.095 1.108 1.079 1.055 2.194 9.067
9 8 7 6 5 4 3 2 1 ppm
NAME 12011001168
EXPNO 874
PROCNO 1
Date_ 20140509
Time 21.46
INSTRUM spect
PROBHD 5 mm PABBO BB/
PULPROG zg30
TD 65536
SOLVENT CDCl3
NS 16
DS 0
SWH 6602.113 Hz
FIDRES 0.100740 Hz
AQ 4.9633098 sec
RG 11.17
DW 75.733 usec
DE 6.50 usec
TE 300.0 K
D1 1.00000000 sec
TD0 1
======== CHANNEL f1 ========
SFO1 400.1526964 MHz
NUC1 1H
P1 9.64 usec
SI 65536
SF 400.1499817 MHz
WDW EM
SSB 0
LB 0.30 Hz
GB 0
PC 1.00

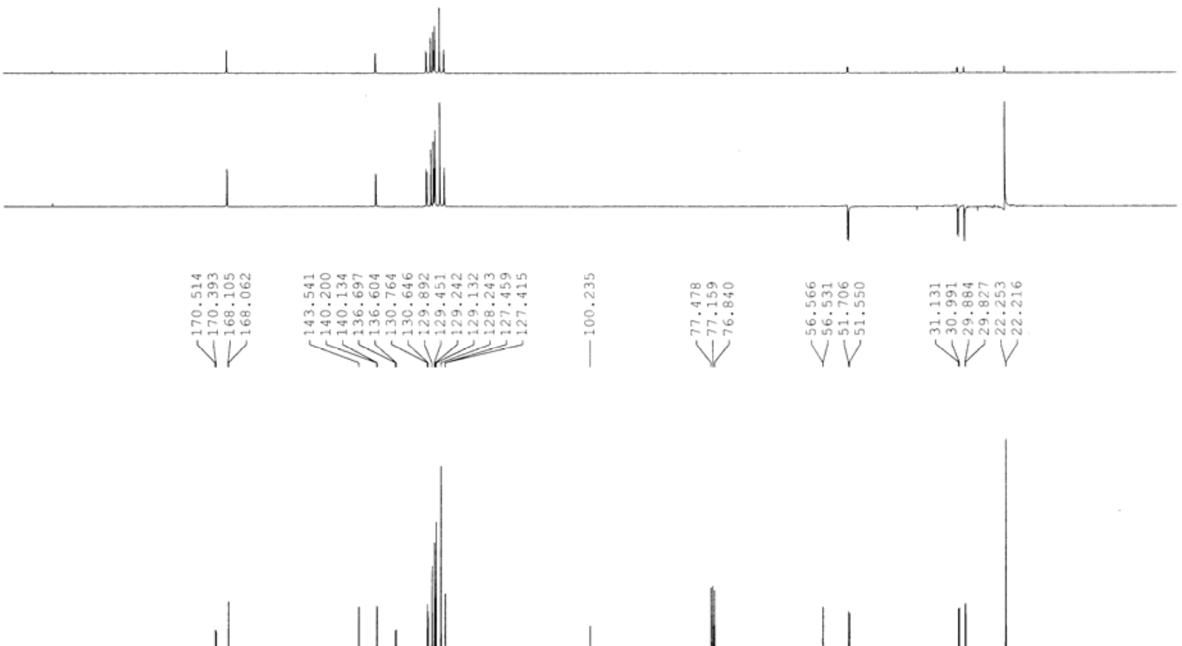
170.514 170.393 168.105 168.062
143.541 140.200 140.134 136.697 136.604 130.764 130.646 129.892 129.451 129.242 129.132 128.243 127.459 127.415
100.235
77.478 77.159 76.840
56.566 56.531 51.706 51.550
31.131 30.991 29.884 29.827 22.253 22.216
200 190 180 170 160 150 140 130 120 110 100 90 80 70 60 50 40 30 20 10 0 ppm

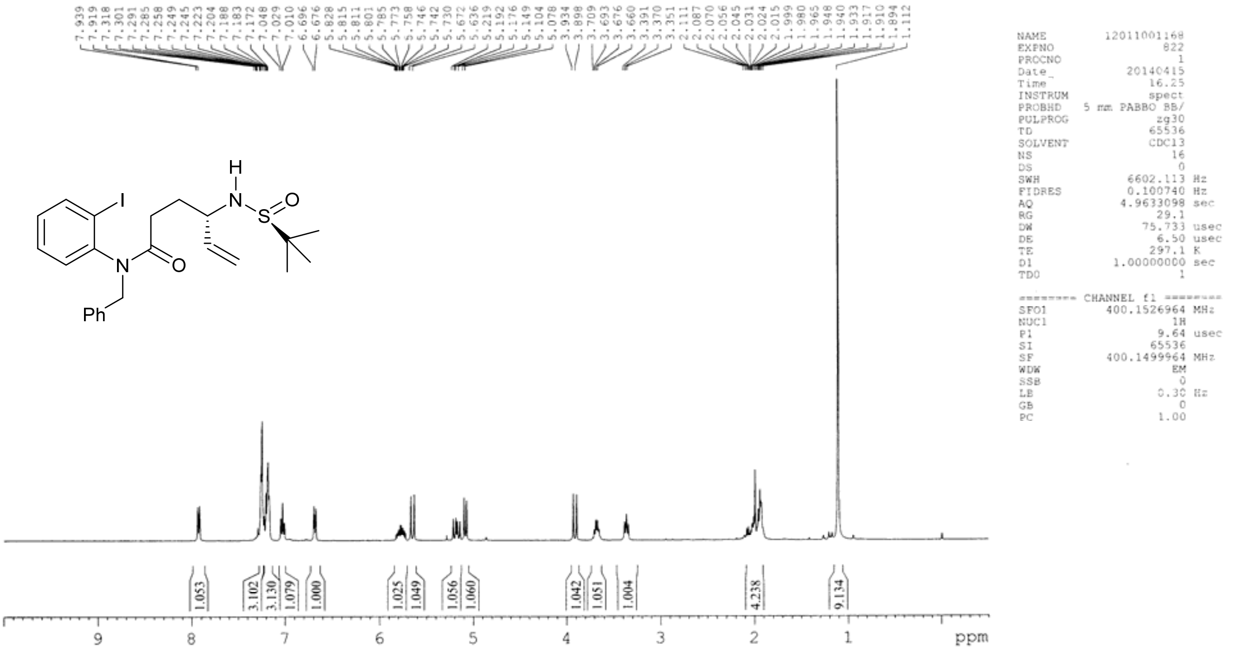
NAME 12011001168
EXPNO 822
PROCNO 1
Date_ 20140415
Time 16.25
INSTRUM spect
PROBHD 5 mm PABBO BB/
PULPROG zg30
TD 65536
SOLVENT CDCl3
NS 16
DS 0
SWH 6602.113 Hz
FIDRES 0.100740 Hz
AQ 4.9633098 sec
RG 29.1
DW 75.733 usec
DE 6.50 usec
TE 297.1 K
D1 1.00000000 sec
TD0 1
======== CHANNEL f1 ========
SFO1 400.1526964 MHz
NUC1 1H
P1 9.64 usec
SI 65536
SF 400.1499964 MHz
WDW EM
SSB 0
LB 0.30 Hz
GB 0
PC 1.00
ppm

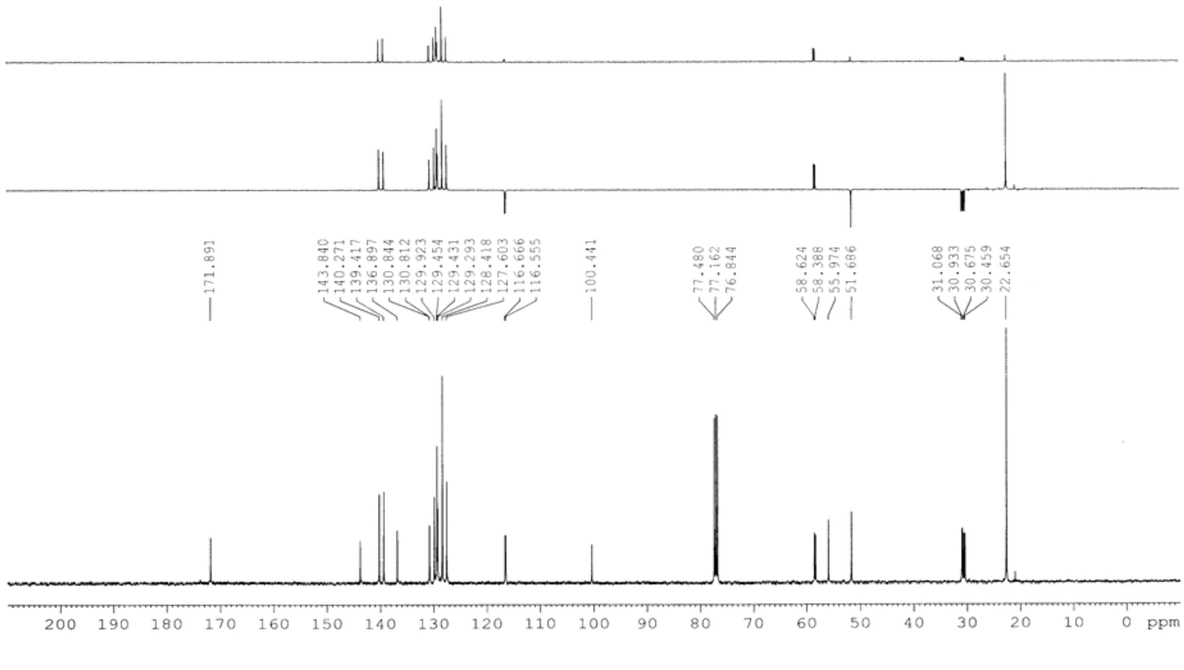
ppm

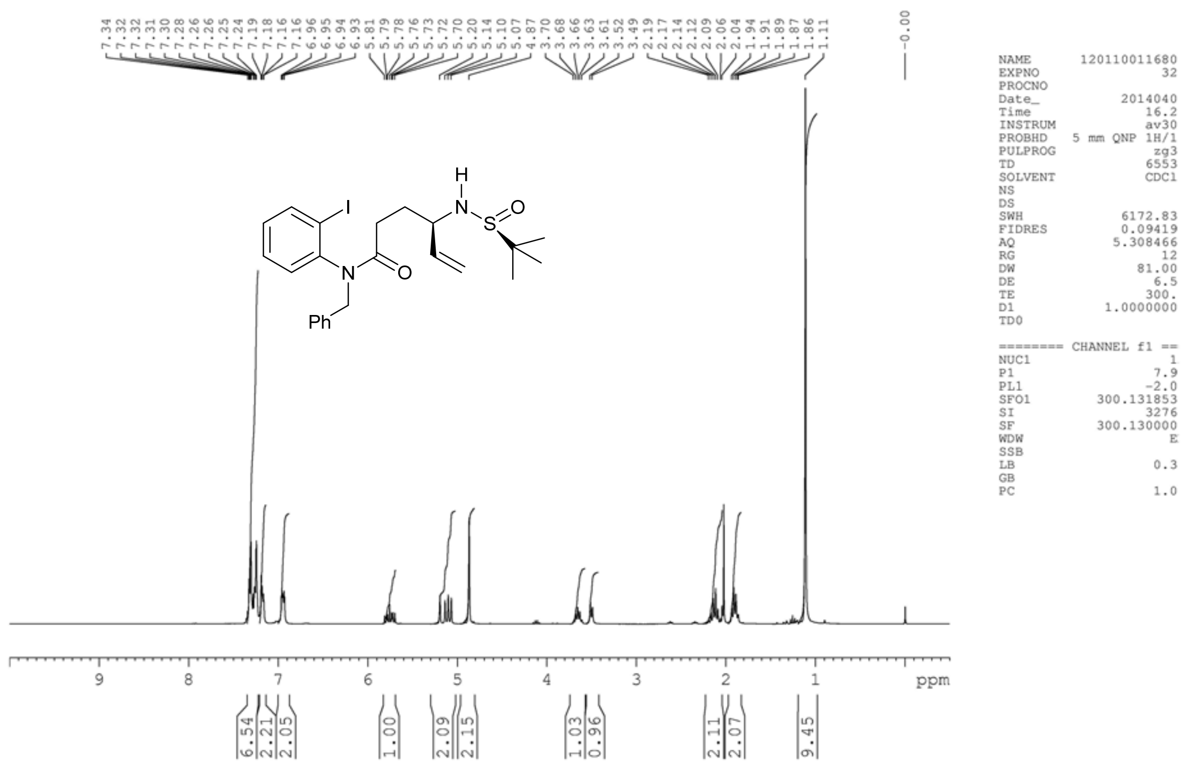
7.34 7.32 7.32 7.31 7.30 7.28 7.26 7.26 7.25 7.24 7.19 7.18 7.16 7.16 6.96 6.95 6.94 6.93 5.81 5.79 5.78 5.76 5.73 5.72 5.70 5.20 5.14 5.10 5.07 4.87 3.70 3.68 3.66 3.63 3.61 3.52 3.49 2.19 2.17 2.14 2.12 2.09 2.06 2.04 1.94 1.91 1.89 1.87 1.86 1.11
-0.00
NAME 120110011680
EXPNO 32
PROCNO
Date_ 2014040
Time 16.2
INSTRUM av30
PROBHD 5 mm QNP 1H/1
PULPROG zg3
TD 6553
SOLVENT CDCl
NS
DS
SWH 6172.83
FIDRES 0.09419
AQ 5.308466
RG 12
DW 81.00
DE 6.5
TE 300.
D1 1.0000000
TD0
======== CHANNEL f1 ==
NUC1 1
P1 7.9
PL1 -2.0
SFO1 300.131853
SI 3276
SF 300.130000
WDW E
SSB
LB 0.3
GB
PC 1.0
I
N
O
Ph
H
N
S
O
9 8 7 6 5 4 3 2 1 ppm
6.54 2.21 2.05 1.00 2.09 2.15 1.03 0.96 2.11 2.07 9.45

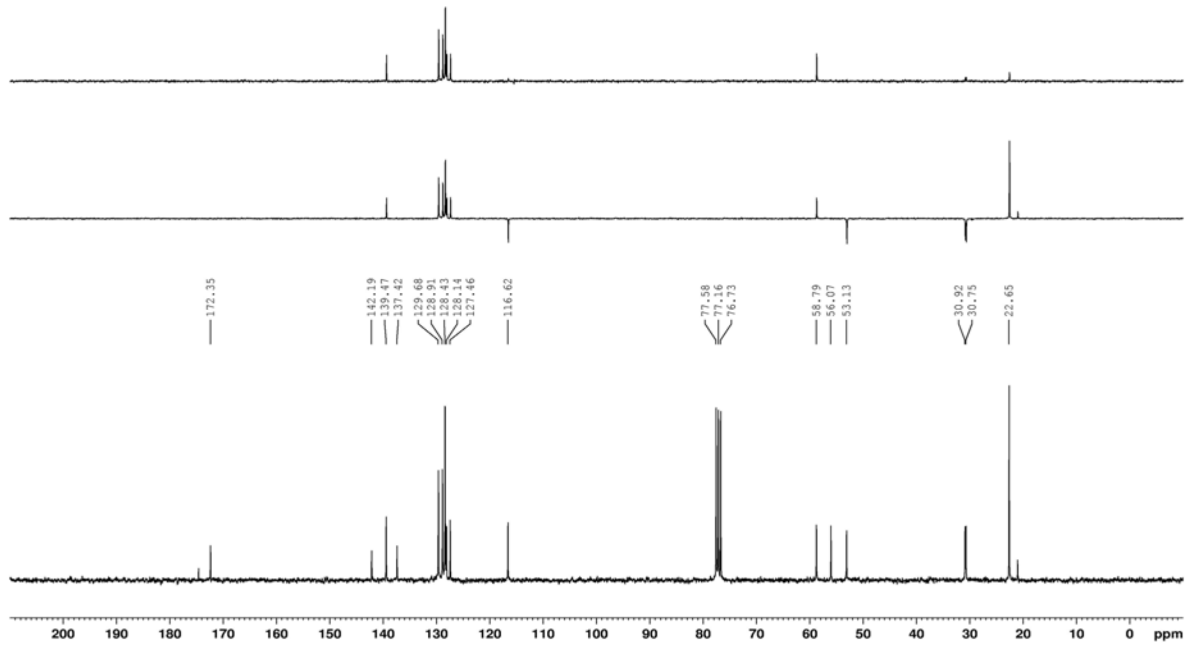
172.35
142.19 139.47 137.42 129.68 128.91 128.43 128.14 127.46
116.62
77.58 77.16 76.73
58.79 56.07 53.13
30.92 30.75
22.65
200 190 180 170 160 150 140 130 120 110 100 90 80 70 60 50 40 30 20 10 0 ppm

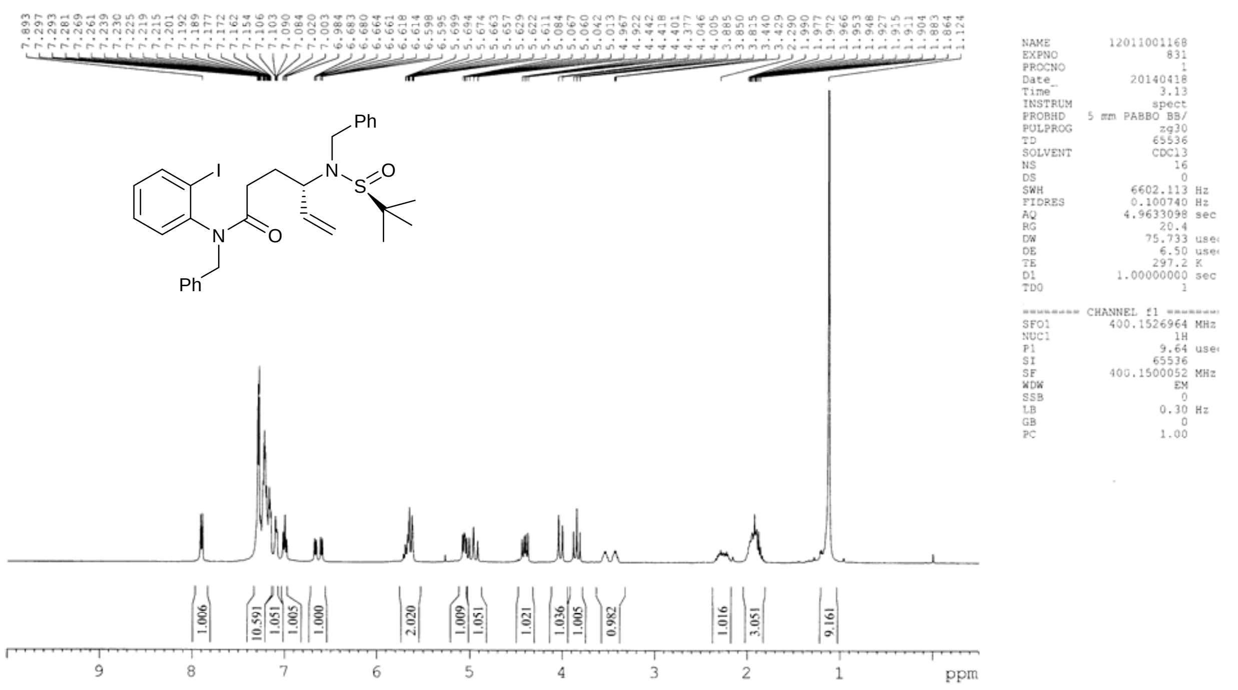
7.893
7.297
7.293
7.281
7.269
7.261
7.239
7.230
7.225
7.219
7.215
7.201
7.192
7.189
7.177
7.172
7.162
7.154
7.106
7.103
7.090
7.084
7.020
7.003
6.984
6.683
6.680
6.664
6.661
6.618
6.614
6.598
6.595
5.699
5.694
5.674
5.663
5.657
5.629
5.622
5.611
5.084
5.067
5.060
5.042
5.013
4.967
4.922
4.442
4.418
4.401
4.377
4.046
4.005
3.885
3.850
3.815
3.429
2.290
1.990
1.977
1.972
1.966
1.953
1.948
1.927
1.915
1.911
1.904
1.883
1.864
1.124
Ph
I
N
S
O
N
O
Ph
NAME 12011001168
EXPNO 831
PROCNO 1
Date_ 20140418
Time 3.13
INSTRUM spect
PROBHD 5 mm PABBO BB/
PULPROG zg30
TD 65536
SOLVENT CDCl3
NS 16
DS 0
SWH 6602.113 Hz
FIDRES 0.100740 Hz
AQ 4.9633098 sec
RG 20.4
DW 75.733 use
DE 6.50 use
TE 297.2 K
D1 1.00000000 sec
TD0 1
======== CHANNEL f1 ========
SFO1 400.1526964 MHz
NUC1 1H
P1 9.64 use
SI 65536
SF 400.1500052 MHz
WDW EM
SSB 0
LB 0.30 Hz
GB 0
PC 1.00
1.006
10.591
1.051
1.005
1.000
2.020
1.009
1.051
1.021
1.036
1.005
0.982
1.016
3.051
9.161
9
8
7
6
5
4
3
2
1
ppm

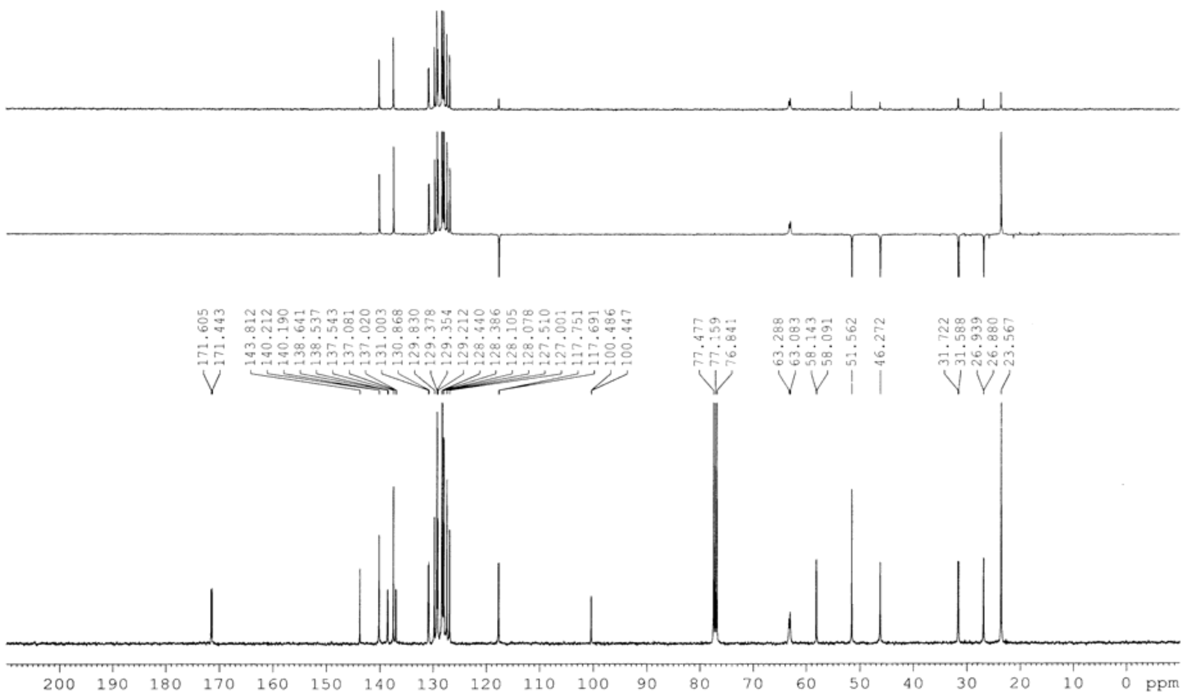
171.605
171.443
143.812
140.212
140.190
138.641
138.537
137.543
137.081
137.020
131.003
130.868
129.830
129.378
129.354
129.212
128.440
128.386
128.105
128.078
127.510
127.001
117.751
117.691
100.486
100.447
77.477
77.159
76.841
63.288
63.083
58.143
58.091
51.562
46.272
31.722
31.588
26.939
26.880
23.567
200
190
180
170
160
150
140
130
120
110
100
90
80
70
60
50
40
30
20
10
0
ppm

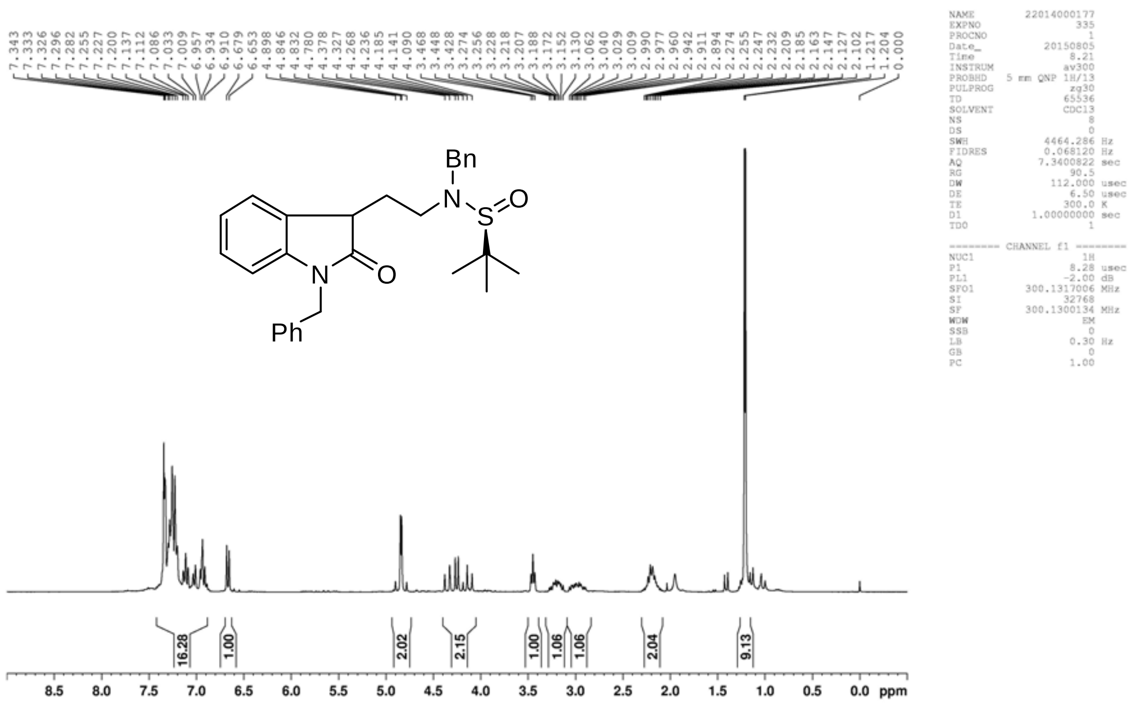
Bn
N
S
O
N
O
Ph
16.28
1.00
2.02
2.15
1.00
1.06
1.06
2.04
9.13
ppm

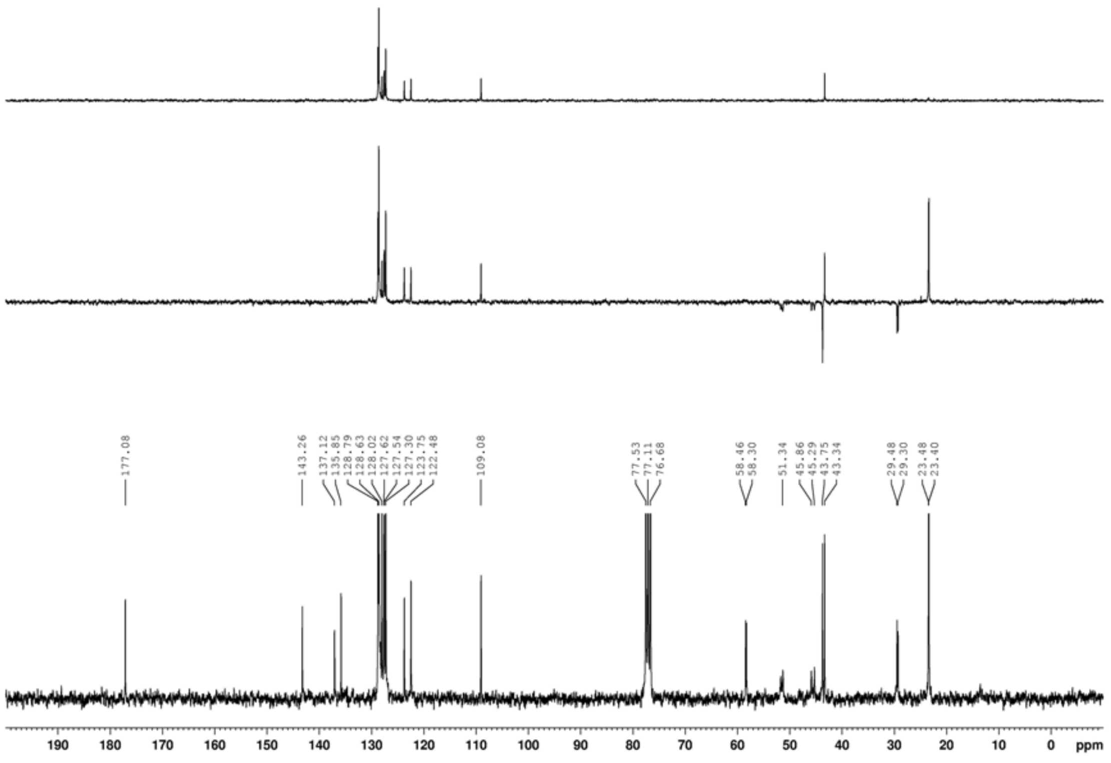
177.08
143.26
137.12
135.85
128.79
128.63
128.02
127.62
127.54
127.30
123.75
122.48
109.08
77.53
77.11
76.68
58.46
58.30
51.34
45.86
45.29
43.75
43.34
29.48
29.30
23.48
23.40
ppm

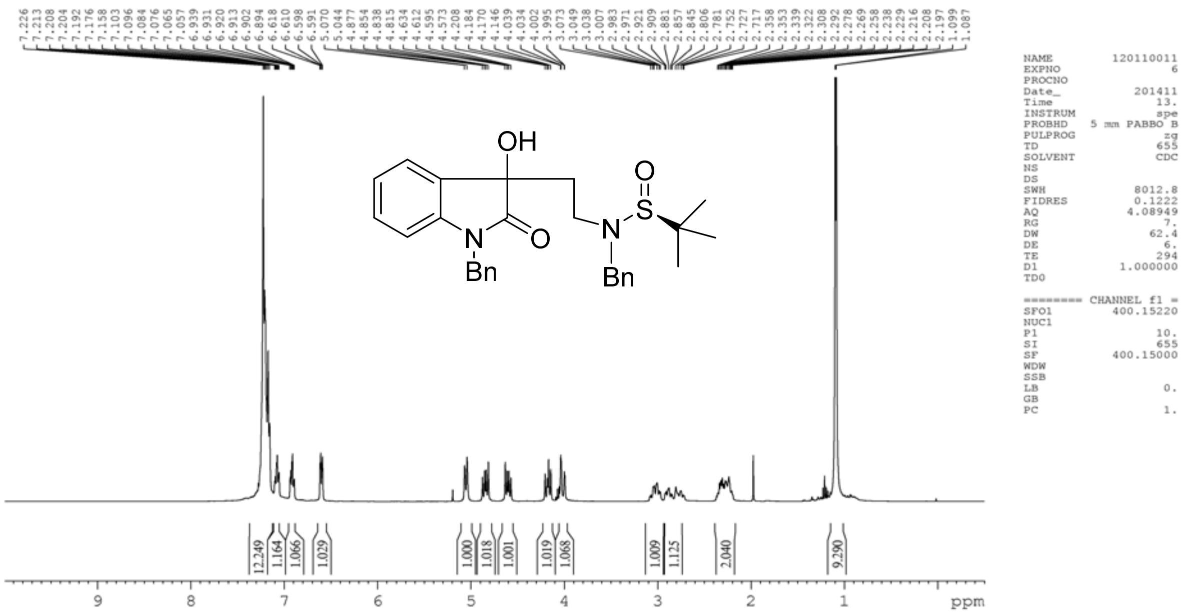
7.226 7.213 7.208 7.204 7.192 7.176 7.158 7.103 7.096 7.084 7.076 7.065 7.057 6.939 6.931 6.920 6.913 6.902 6.894 6.618 6.610 6.598 6.591 5.070 5.044 4.877 4.854 4.838 4.815 4.634 4.612 4.595 4.573 4.208 4.184 4.170 4.146 4.039 4.034 4.002 3.995 3.073 3.049 3.038 3.007 2.983 2.971 2.921 2.909 2.881 2.857 2.845 2.806 2.781 2.752 2.727 2.717 2.358 2.353 2.339 2.322 2.308 2.292 2.278 2.269 2.258 2.238 2.229 2.216 2.208 2.197 1.099 1.087
OH
O
S
N
O
N
Bn
Bn
NAME 120110011
EXPNO 6
PROCNO
Date_ 201411
Time 13.
INSTRUM spe
PROBHD 5 mm PABBO B
PULPROG zg
TD 655
SOLVENT CDC
NS
DS
SWH 8012.8
FIDRES 0.1222
AQ 4.08949
RG 7.
DW 62.4
DE 6.
TE 294
D1 1.000000
TD0
======== CHANNEL f1 =
SFO1 400.15220
NUC1
P1 10.
SI 655
SF 400.15000
WDW
SSB
LB 0.
GB
PC 1.
12.249 1.164 1.066 1.029 1.000 1.018 1.001 1.019 1.068 1.009 1.125 2.040 9.290
9 8 7 6 5 4 3 2 1 ppm

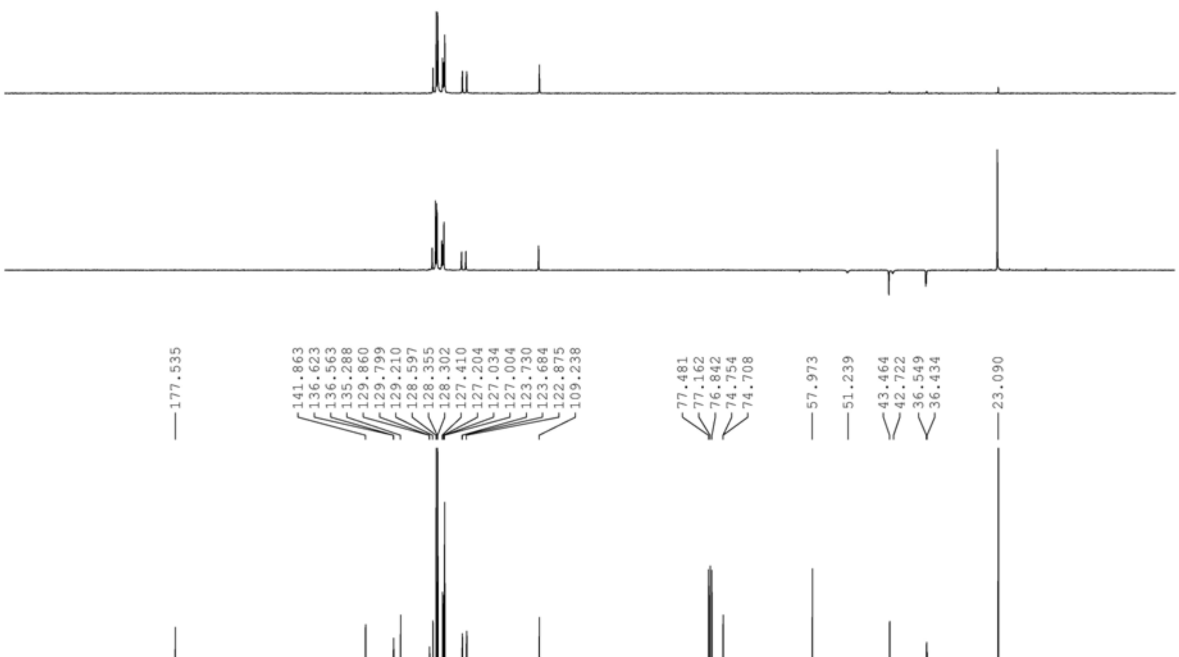
177.535
141.863 136.623 136.563 135.288 129.860 129.799 129.210 128.597 128.355 128.302 127.410 127.204 127.034 127.004 123.730 123.684 122.875 109.238
77.481 77.162 76.842 74.754 74.708
57.973
51.239
43.464 42.722 36.549 36.434
23.090
200 190 180 170 160 150 140 130 120 110 100 90 80 70 60 50 40 30 20 10 0 ppm

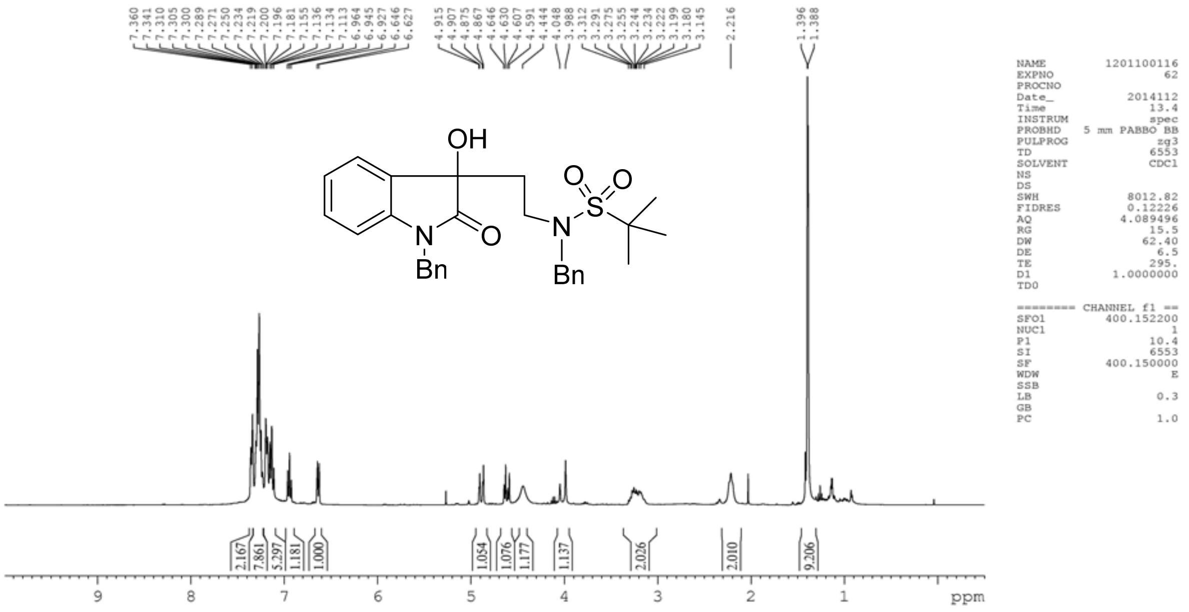
7.360
7.341
7.310
7.305
7.300
7.289
7.271
7.250
7.234
7.219
7.200
7.196
7.181
7.155
7.136
7.134
7.113
6.964
6.945
6.927
6.646
6.627
4.915
4.907
4.875
4.867
4.646
4.630
4.607
4.591
4.444
4.048
3.988
3.312
3.291
3.275
3.255
3.244
3.234
3.222
3.199
3.180
3.145
2.216
1.396
1.388
OH
O
S
O
N
O
N
Bn
Bn
NAME 1201100116
EXPNO 62
PROCNO
Date_ 2014112
Time 13.4
INSTRUM spec
PROBHD 5 mm PABBO BB
PULPROG zg3
TD 6553
SOLVENT CDCl
NS
DS
SWH 8012.82
FIDRES 0.12226
AQ 4.089496
RG 15.5
DW 62.40
DE 6.5
TE 295.
D1 1.0000000
TD0
======== CHANNEL f1 ==
SFO1 400.152200
NUC1 1
P1 10.4
SI 6553
SF 400.150000
WDW E
SSB
LB 0.3
GB
PC 1.0
2.167
7.861
5.297
1.181
1.000
1.054
1.076
1.177
1.137
2.026
2.010
9.206
9
8
7
6
5
4
3
2
1
ppm

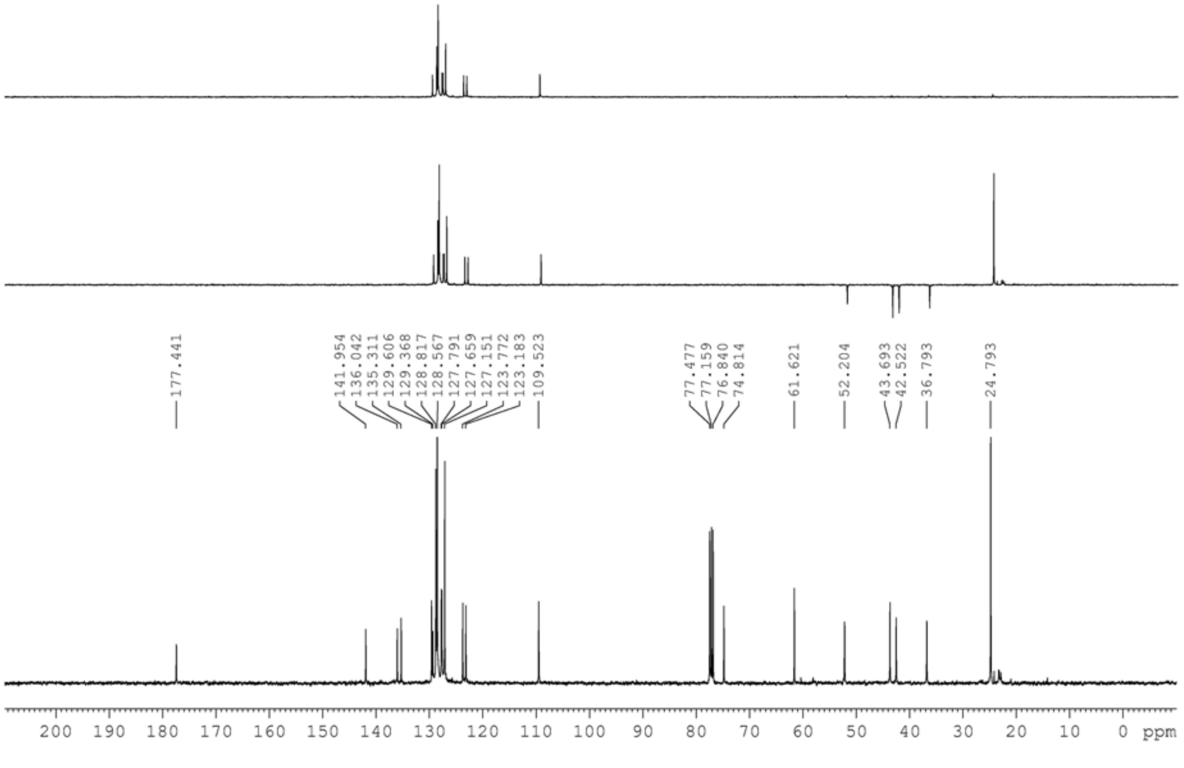
177.441
141.954
136.042
135.311
129.606
129.368
128.817
128.567
127.791
127.659
127.151
123.772
123.183
109.523
77.477
77.159
76.840
74.814
61.621
52.204
43.693
42.522
36.793
24.793
200
190
180
170
160
150
140
130
120
110
100
90
80
70
60
50
40
30
20
10
0
ppm

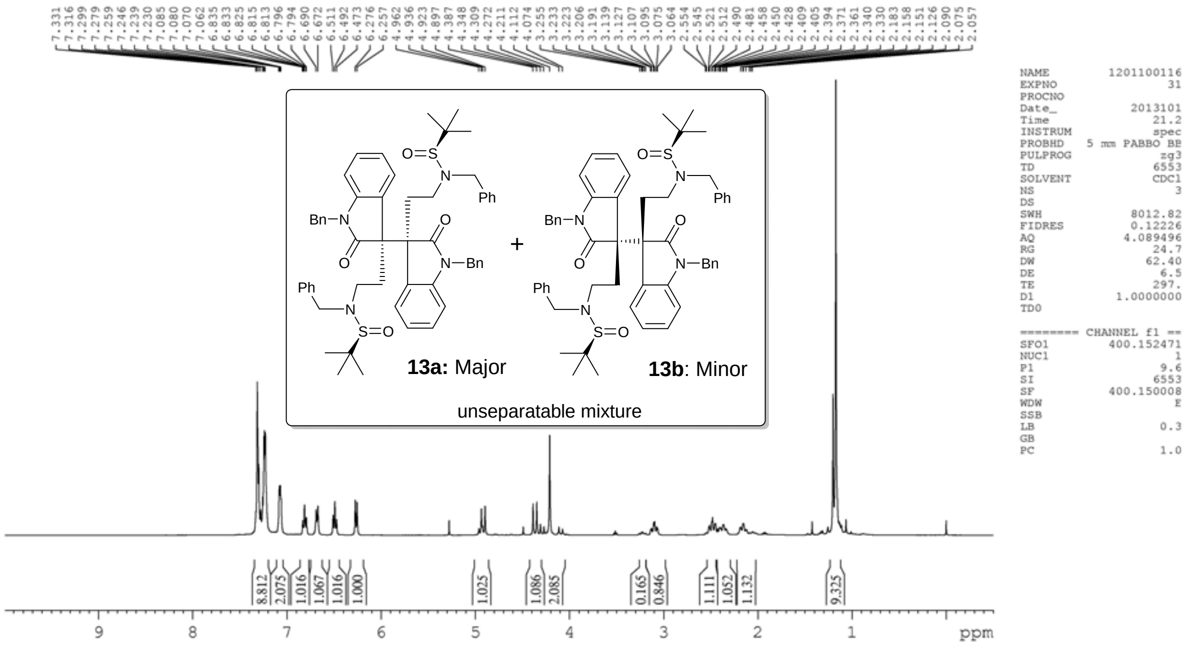
7.331 7.316 7.299 7.279 7.259 7.246 7.239 7.230 7.085 7.080 7.070 7.062 6.835 6.833 6.825 6.815 6.813 6.796 6.794 6.690 6.672 6.511 6.492 6.473 6.276 6.257 4.962 4.936 4.923 4.897 4.387 4.348 4.309 4.272 4.211 4.112 4.074 3.255 3.233 3.223 3.206 3.191 3.139 3.127 3.107 3.095 3.075 3.064 2.554 2.545 2.521 2.512 2.490 2.481 2.458 2.450 2.428 2.409 2.405 2.394 2.371 2.361 2.340 2.330 2.183 2.158 2.151 2.126 2.090 2.075 2.057
Bn−N
O
N−Bn
Ph
O=S
S=O
+
13a: Major
13b: Minor
unseparatable mixture
8.812 2.075 1.016 1.067 1.016 1.000 1.025 1.086 2.085 0.165 0.846 1.111 1.052 1.132 9.325
9 8 7 6 5 4 3 2 1 ppm
NAME 1201100116
EXPNO 31
PROCNO
Date_ 2013101
Time 21.2
INSTRUM spec
PROBHD 5 mm PABBO BB
PULPROG zg3
TD 6553
SOLVENT CDCl
NS 3
DS
SWH 8012.82
FIDRES 0.12226
AQ 4.089496
RG 24.7
DW 62.40
DE 6.5
TE 297.
D1 1.0000000
TD0
======== CHANNEL f1 ==
SFO1 400.152471
NUC1 1
P1 9.6
SI 6553
SF 400.150008
WDW E
SSB
LB 0.3
GB
PC 1.0

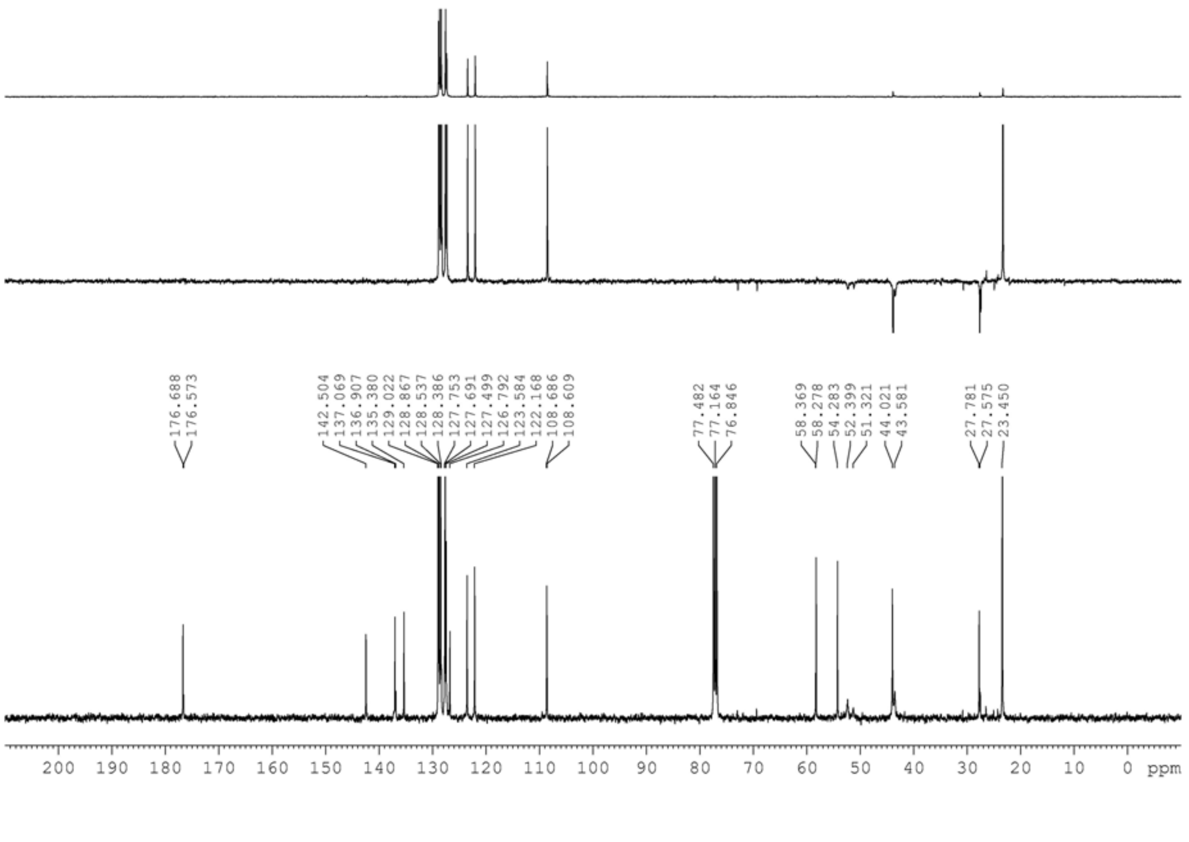
176.688 176.573
142.504 137.069 136.907 135.380 129.022 128.867 128.537 128.386 127.753 127.691 127.499 126.792 123.584 122.168 108.686 108.609
77.482 77.164 76.846
58.369 58.278 54.283 52.399 51.321 44.021 43.581
27.781 27.575 23.450
200 190 180 170 160 150 140 130 120 110 100 90 80 70 60 50 40 30 20 10 0 ppm

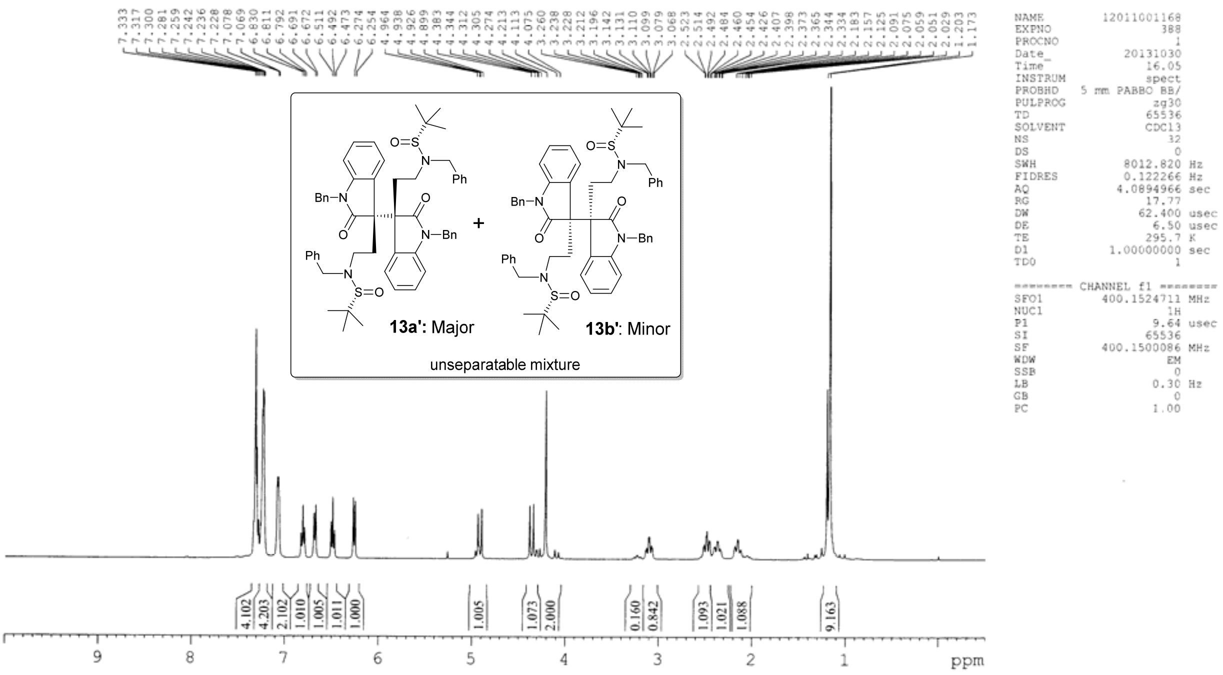
7.333
7.317
7.300
7.281
7.259
7.242
7.236
7.228
7.078
7.069
6.830
6.811
6.792
6.691
6.672
6.511
6.492
6.473
6.274
6.254
4.964
4.938
4.926
4.899
4.383
4.344
4.312
4.305
4.274
4.213
4.113
4.075
3.260
3.238
3.228
3.212
3.196
3.142
3.131
3.110
3.099
3.079
3.068
2.523
2.514
2.492
2.484
2.460
2.454
2.426
2.407
2.398
2.373
2.365
2.344
2.334
2.183
2.157
2.125
2.091
2.075
2.059
2.051
2.029
1.203
1.173
NAME 12011001168
EXPNO 388
PROCNO 1
Date_ 20131030
Time 16.05
INSTRUM spect
PROBHD 5 mm PABBO BB/
PULPROG zg30
TD 65536
SOLVENT CDCl3
NS 32
DS 0
SWH 8012.820 Hz
FIDRES 0.122266 Hz
AQ 4.0894966 sec
RG 17.77
DW 62.400 usec
DE 6.50 usec
TE 295.7 K
D1 1.00000000 sec
TD0 1
======== CHANNEL f1 ========
SFO1 400.1524711 MHz
NUC1 1H
P1 9.64 usec
SI 65536
SF 400.1500086 MHz
WDW EM
SSB 0
LB 0.30 Hz
GB 0
PC 1.00
13a': Major
13b': Minor
unseparatable mixture
4.102
4.203
2.102
1.010
1.005
1.011
1.000
1.005
1.073
2.000
0.160
0.842
1.093
1.021
1.088
9.163
ppm

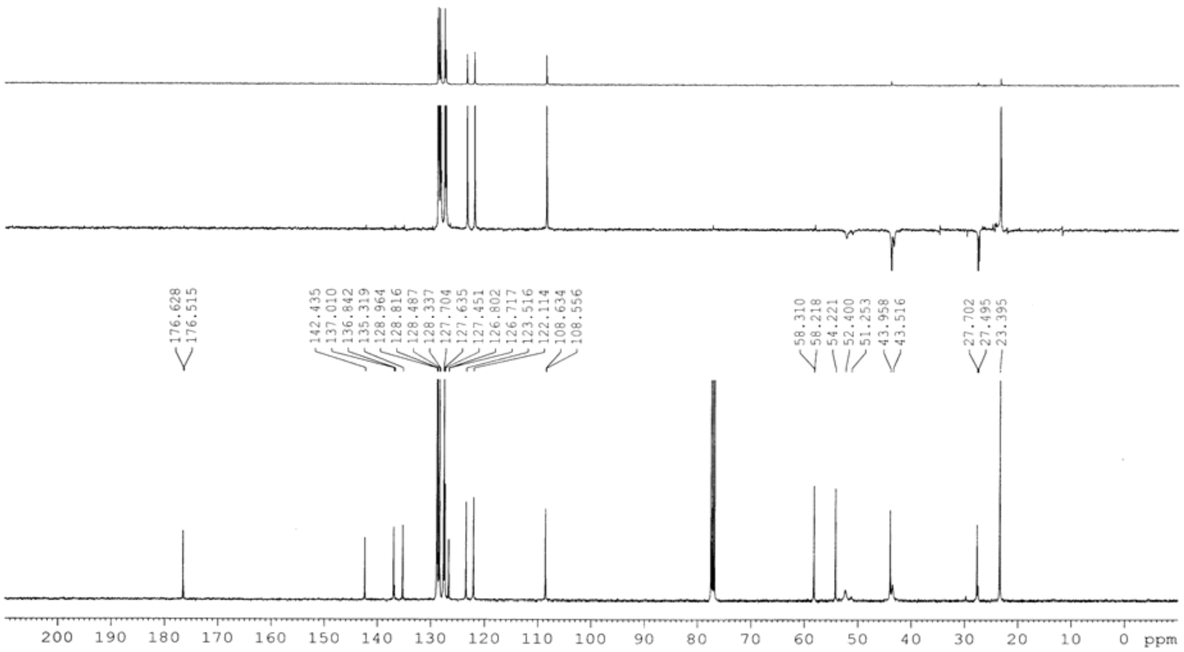
176.628
176.515
142.435
137.010
136.842
135.319
128.964
128.816
128.487
128.337
127.704
127.635
127.451
126.802
126.717
123.516
122.114
108.634
108.556
58.310
58.218
54.221
52.400
51.253
43.958
43.516
27.702
27.495
23.395
ppm

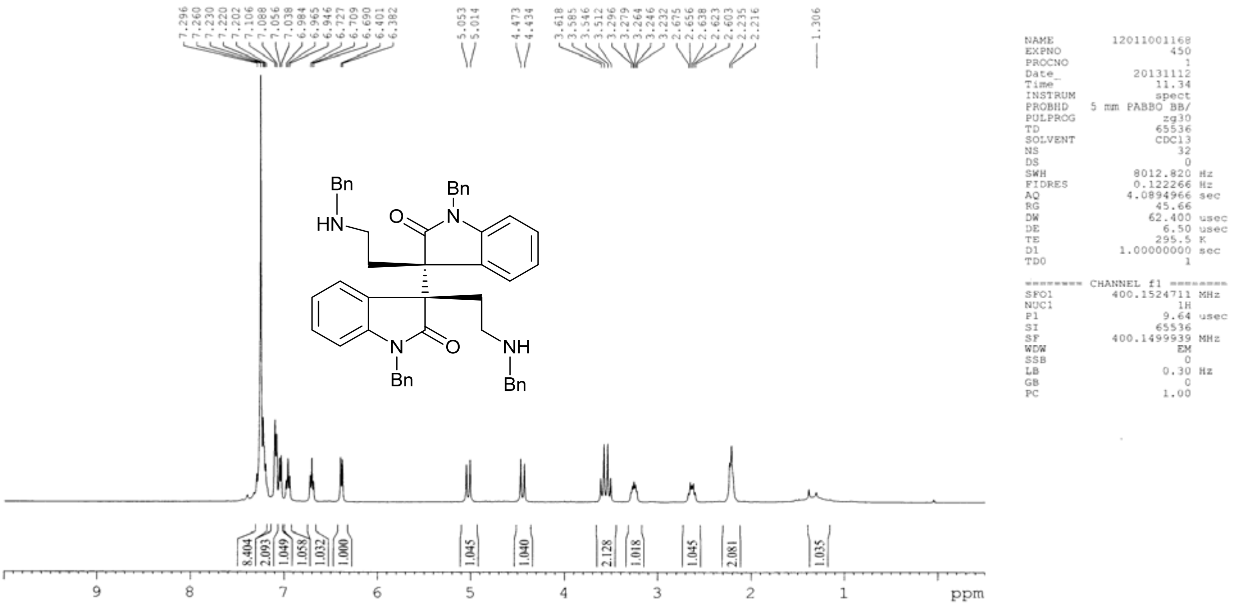
7.296 7.260 7.230 7.220 7.202 7.106 7.088 7.056 7.038 6.984 6.965 6.946 6.727 6.709 6.690 6.401 6.382
5.053 5.014
4.473 4.434
3.618 3.585 3.546 3.512 3.296 3.279 3.264 3.246 3.232 2.675 2.656 2.638 2.623 2.603 2.235 2.216
1.306
Bn
HN
Bn
O
N
N
O
NH
Bn
Bn
8.404 2.093 1.049 1.058 1.032 1.000 1.045 1.040 2.128 1.018 1.045 2.081 1.035
9 8 7 6 5 4 3 2 1 ppm
NAME 12011001168
EXPNO 450
PROCNO 1
Date_ 20131112
Time 11.34
INSTRUM spect
PROBHD 5 mm PABBO BB/
PULPROG zg30
TD 65536
SOLVENT CDCl3
NS 32
DS 0
SWH 8012.820 Hz
FIDRES 0.122266 Hz
AQ 4.0894966 sec
RG 45.66
DW 62.400 usec
DE 6.50 usec
TE 295.5 K
D1 1.00000000 sec
TD0 1
======== CHANNEL f1 ========
SFO1 400.1524711 MHz
NUC1 1H
P1 9.64 usec
SI 65536
SF 400.1499939 MHz
WDW EM
SSB 0
LB 0.30 Hz
GB 0
PC 1.00

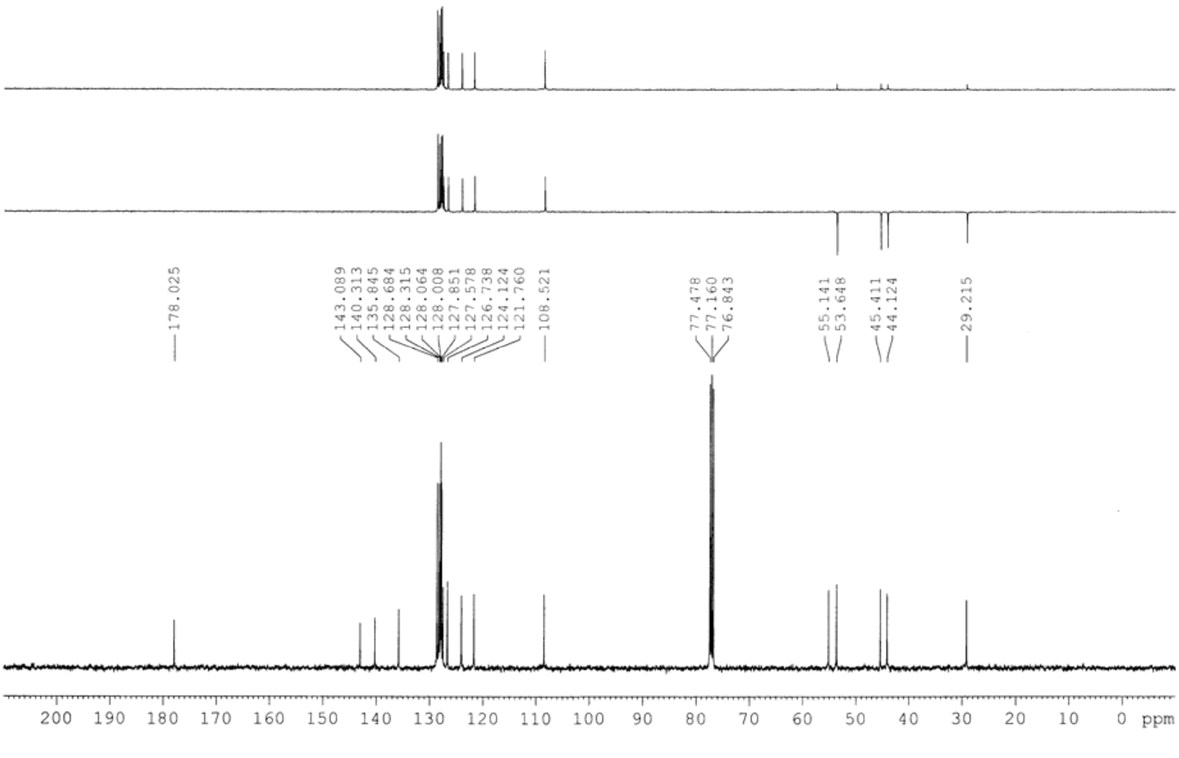
178.025
143.089 140.313 135.845 128.684 128.315 128.064 128.008 127.851 127.578 126.738 124.124 121.760
108.521
77.478 77.160 76.843
55.141 53.648
45.411 44.124
29.215
200 190 180 170 160 150 140 130 120 110 100 90 80 70 60 50 40 30 20 10 0 ppm

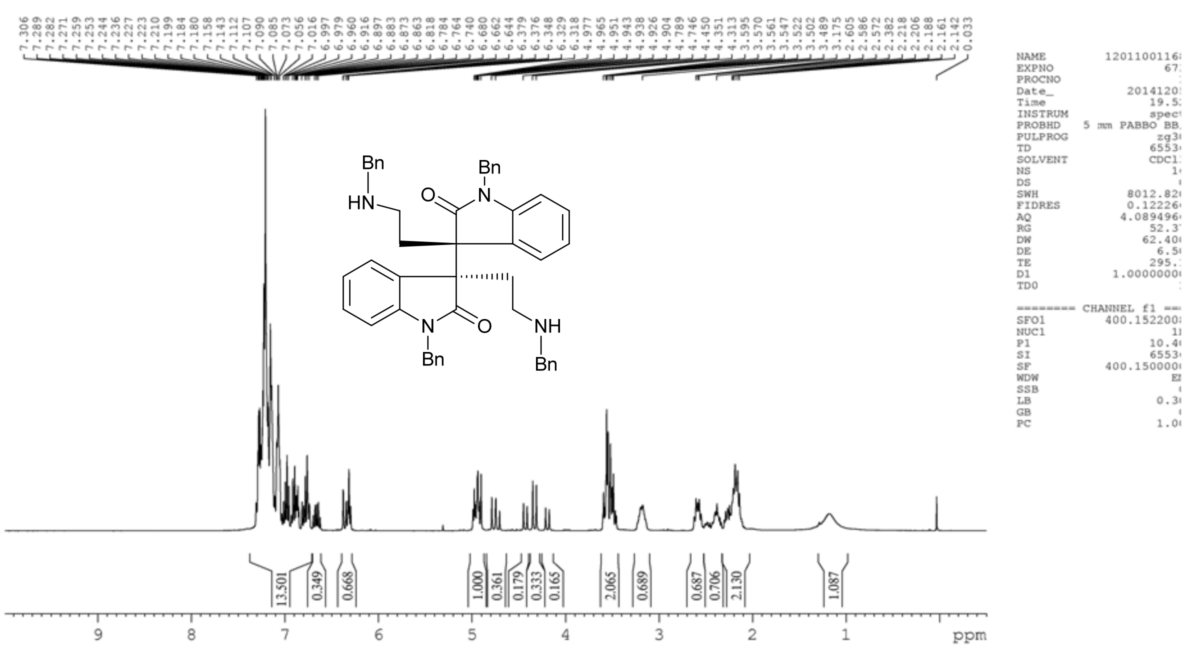
7.306 7.289 7.282 7.271 7.259 7.253 7.244 7.236 7.227 7.223 7.210 7.199 7.184 7.180 7.158 7.143 7.112 7.107 7.090 7.085 7.073 7.056 7.016 6.997 6.979 6.960 6.916 6.897 6.883 6.873 6.863 6.818 6.784 6.764 6.740 6.680 6.662 6.644 6.379 6.376 6.348 6.329 6.318 4.977 4.965 4.951 4.943 4.938 4.926 4.904 4.789 4.746 4.450 4.351 4.313 3.595 3.570 3.561 3.547 3.522 3.502 3.489 3.175 2.605 2.586 2.572 2.382 2.218 2.206 2.188 2.161 2.142 0.033
Bn
HN
O
N
Bn
N
O
NH
Bn
Bn
NAME
EXPNO
PROCNO
Date_
Time
INSTRUM
PROBHD 5 mm PABBO BB
PULPROG
TD
SOLVENT
NS
DS
SWH 8012.82
FIDRES 0.12226
AQ 4.089496
RG
DW
DE
TE
D1 1.0000000
TD0
======== CHANNEL f1 ==
SFO1 400.152200
NUC1
P1
SI
SF 400.150000
WDW
SSB
LB
GB
PC
13.501 0.349 0.668 1.000 0.361 0.179 0.333 0.165 2.065 0.689 0.687 0.706 2.130 1.087
9 8 7 6 5 4 3 2 1 ppm

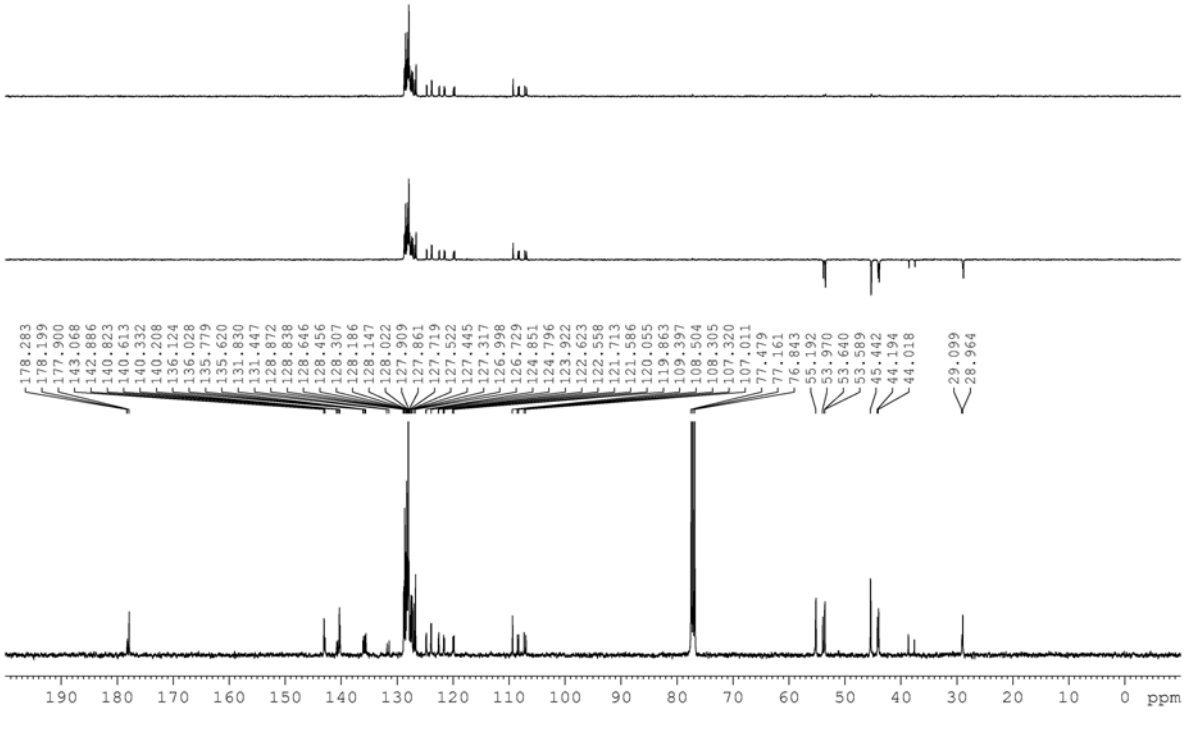
178.283 178.199 177.900 143.068 142.886 140.823 140.613 140.332 140.208 136.124 136.028 135.779 135.620 131.830 131.447 128.872 128.838 128.646 128.456 128.307 128.186 128.147 128.022 127.909 127.861 127.719 127.522 127.445 127.317 126.998 126.729 124.851 124.796 123.922 122.623 122.558 121.713 121.586 120.055 119.863 109.397 108.504 108.305 107.320 107.011 77.479 77.161 76.843 55.192 53.970 53.640 53.589 45.442 44.194 44.018 29.099 28.964
190 180 170 160 150 140 130 120 110 100 90 80 70 60 50 40 30 20 10 0 ppm

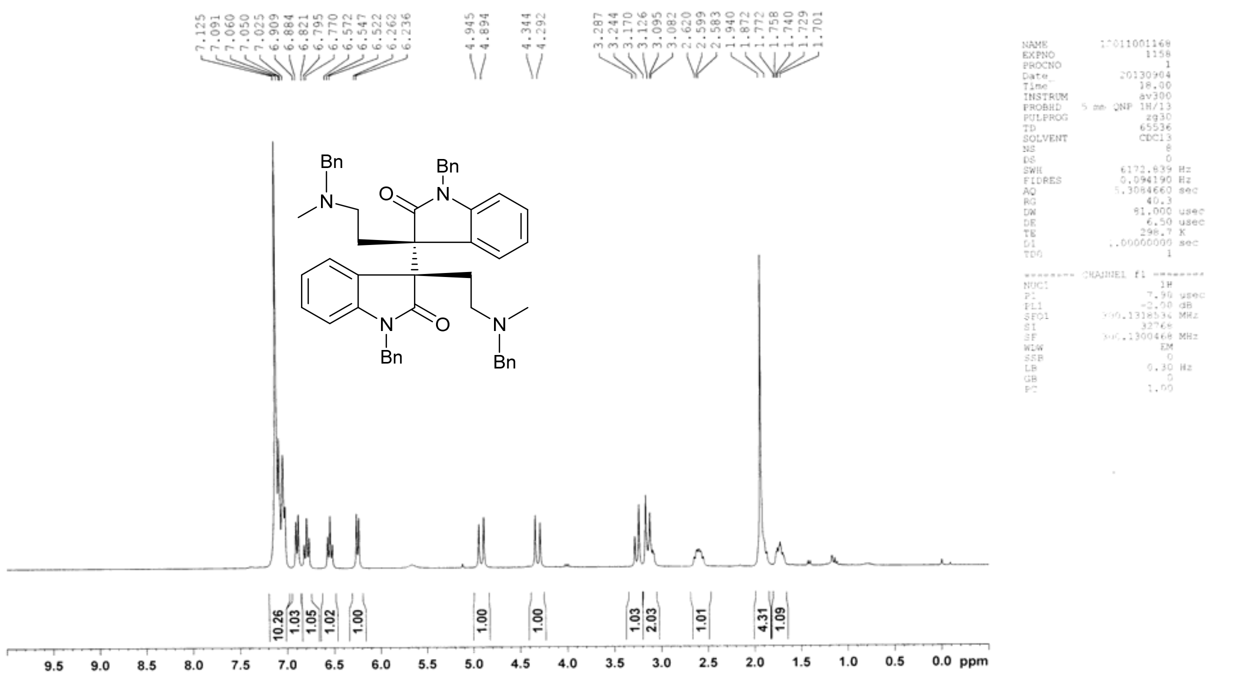
7.125
7.091
7.060
7.050
7.025
6.909
6.884
6.821
6.795
6.770
6.572
6.547
6.522
6.262
6.236
4.945
4.894
4.344
4.292
3.287
3.244
3.170
3.126
3.095
3.082
2.620
2.599
2.583
1.940
1.872
1.772
1.758
1.740
1.729
1.701
Bn
N
O
N
Bn
N
O
Bn
N
Bn
10.26
1.03
1.05
1.02
1.00
1.00
1.00
1.03
2.03
1.01
4.31
1.09
9.5 9.0 8.5 8.0 7.5 7.0 6.5 6.0 5.5 5.0 4.5 4.0 3.5 3.0 2.5 2.0 1.5 1.0 0.5 0.0 ppm

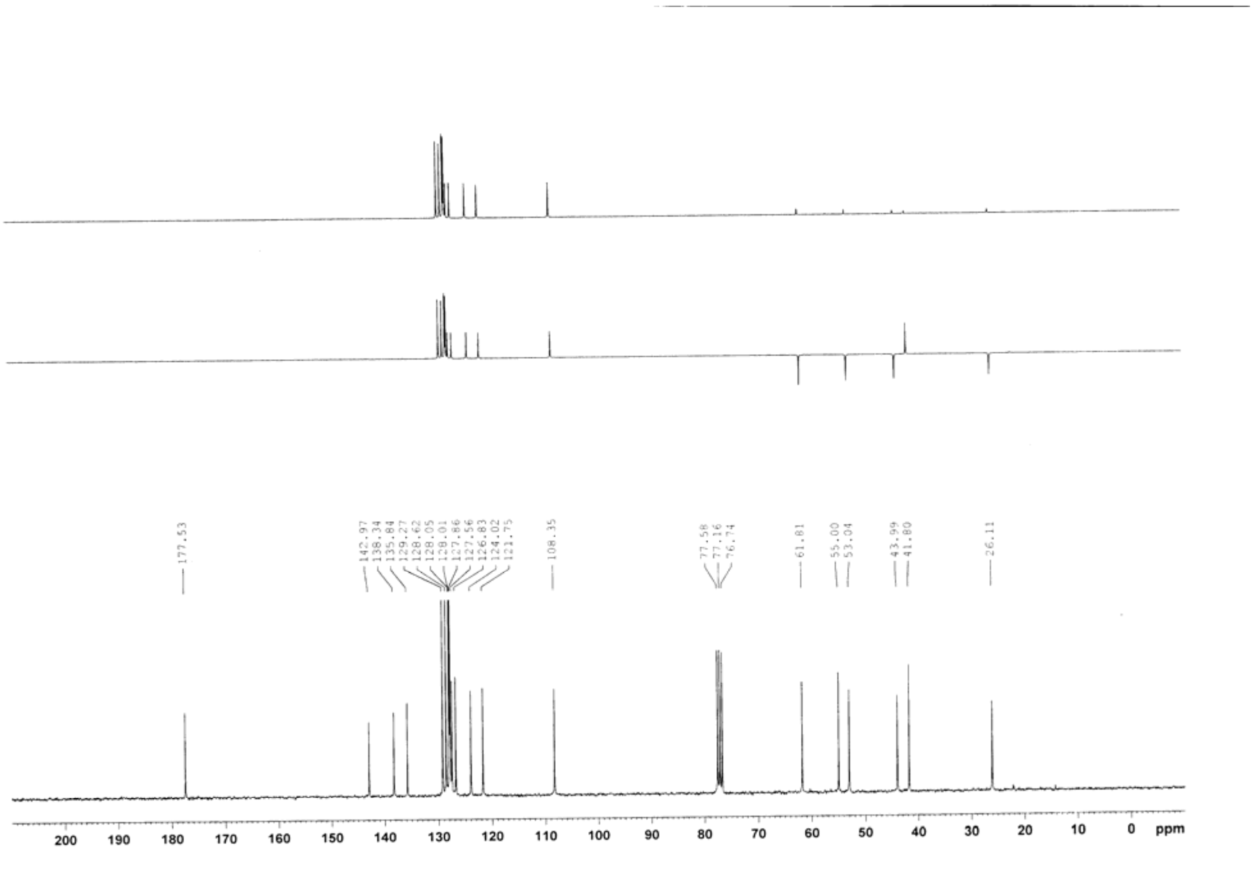
177.53
142.97
138.34
135.84
129.27
128.62
128.05
128.01
127.86
127.56
126.83
124.02
121.75
108.35
77.58
77.16
76.74
61.81
55.00
53.04
43.99
41.80
26.11
200 190 180 170 160 150 140 130 120 110 100 90 80 70 60 50 40 30 20 10 0 ppm

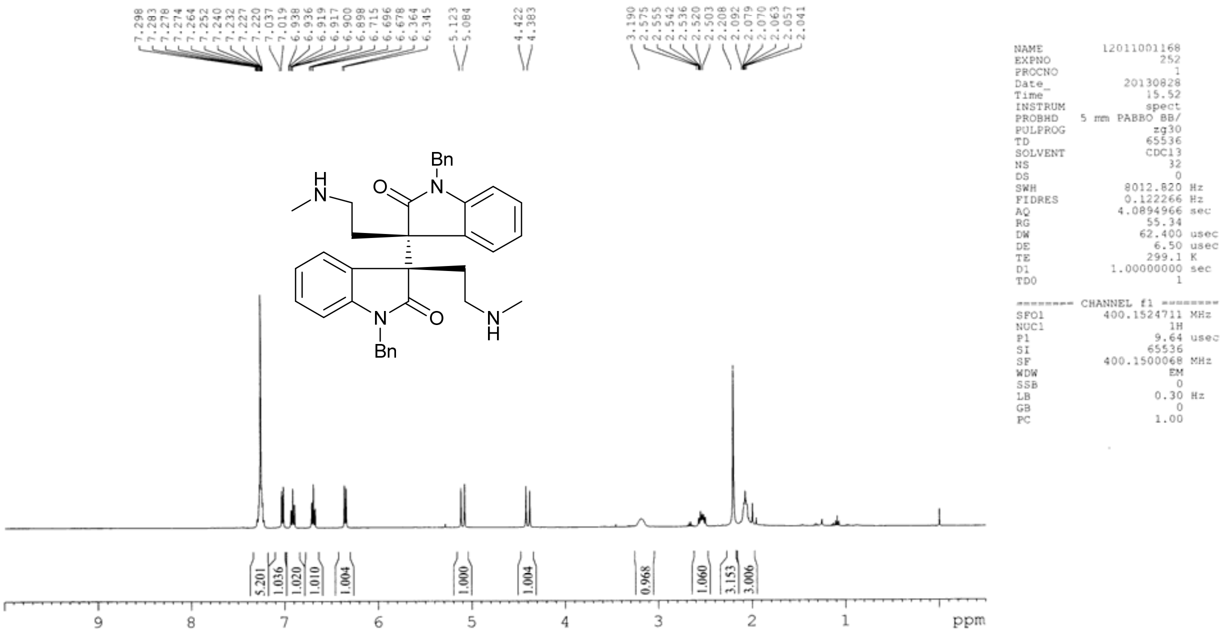
7.298 7.283 7.278 7.274 7.264 7.252 7.240 7.232 7.227 7.220 7.037 7.019 6.938 6.936 6.919 6.917 6.900 6.898 6.715 6.696 6.678 6.364 6.345 5.123 5.084 4.422 4.383
3.190 2.575 2.555 2.542 2.536 2.520 2.503 2.208 2.092 2.079 2.070 2.063 2.057 2.041
Bn
H
N
O
N
N
O
N
H
Bn
NAME 12011001168
EXPNO 252
PROCNO 1
Date_ 20130828
Time 15.52
INSTRUM spect
PROBHD 5 mm PABBO BB/
PULPROG zg30
TD 65536
SOLVENT CDCl3
NS 32
DS 0
SWH 8012.820 Hz
FIDRES 0.122266 Hz
AQ 4.0894966 sec
RG 55.34
DW 62.400 usec
DE 6.50 usec
TE 299.1 K
D1 1.00000000 sec
TD0 1
======== CHANNEL f1 ========
SFO1 400.1524711 MHz
NUC1 1H
P1 9.64 usec
SI 65536
SF 400.1500068 MHz
WDW EM
SSB 0
LB 0.30 Hz
GB 0
PC 1.00
5.201 1.036 1.020 1.010 1.004 1.000 1.004 0.968 1.060 3.153 3.006
9 8 7 6 5 4 3 2 1 ppm

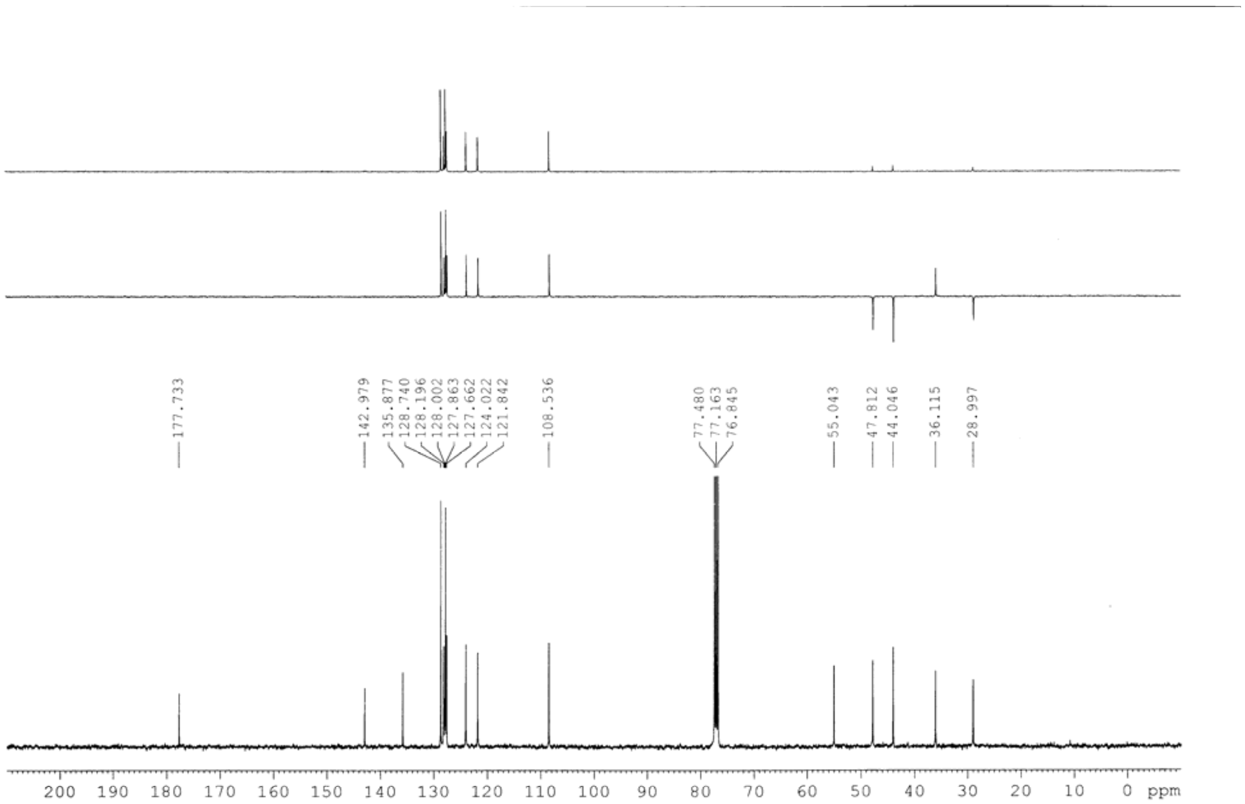
177.733
142.979 135.877 128.740 128.196 128.002 127.863 127.662 124.022 121.842
108.536
77.480 77.163 76.845
55.043
47.812 44.046
36.115
28.997
200 190 180 170 160 150 140 130 120 110 100 90 80 70 60 50 40 30 20 10 0 ppm

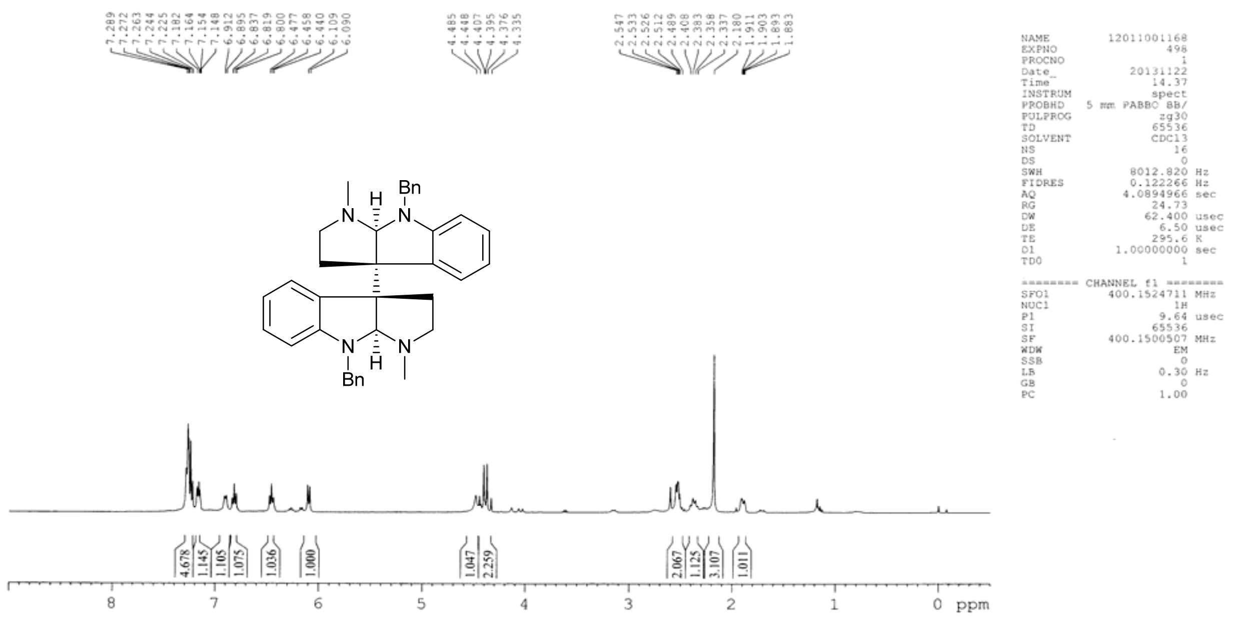

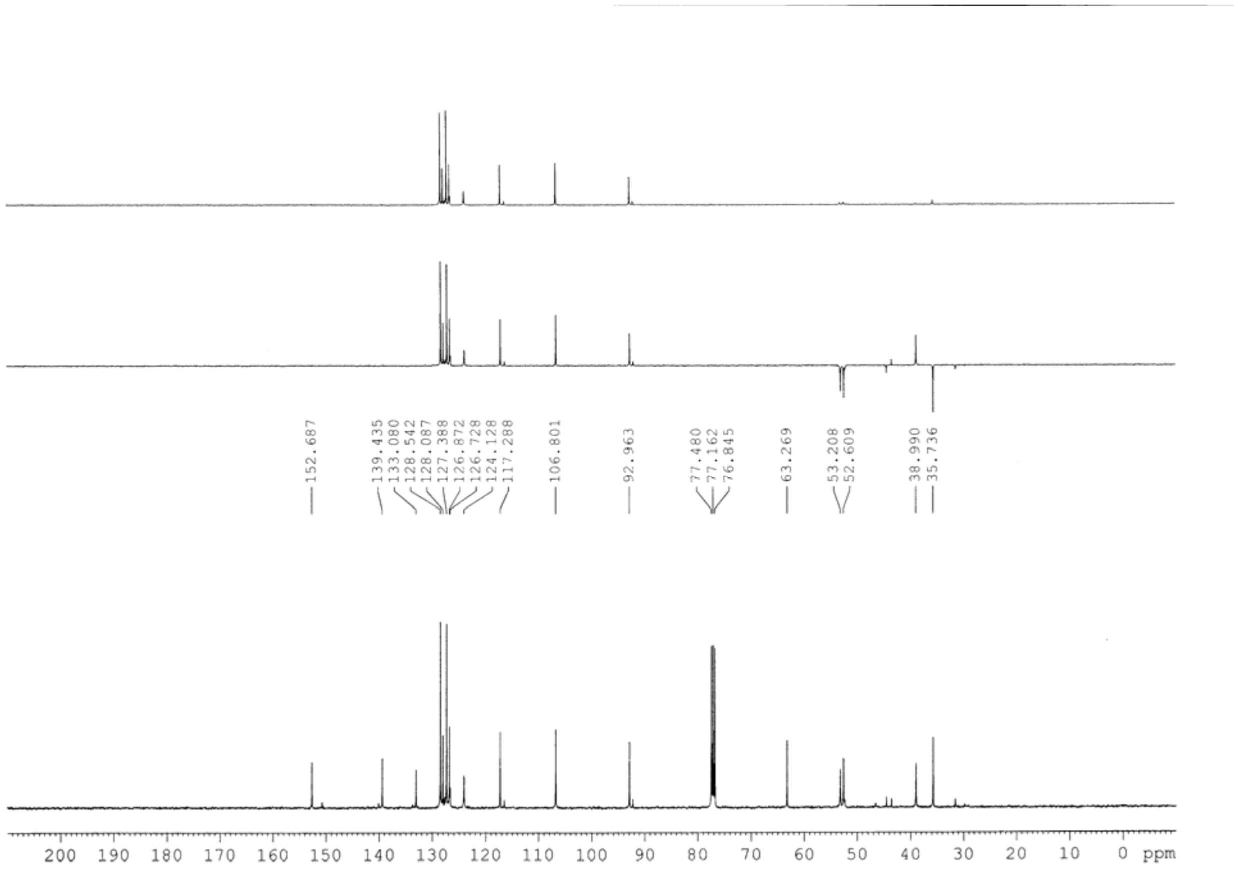

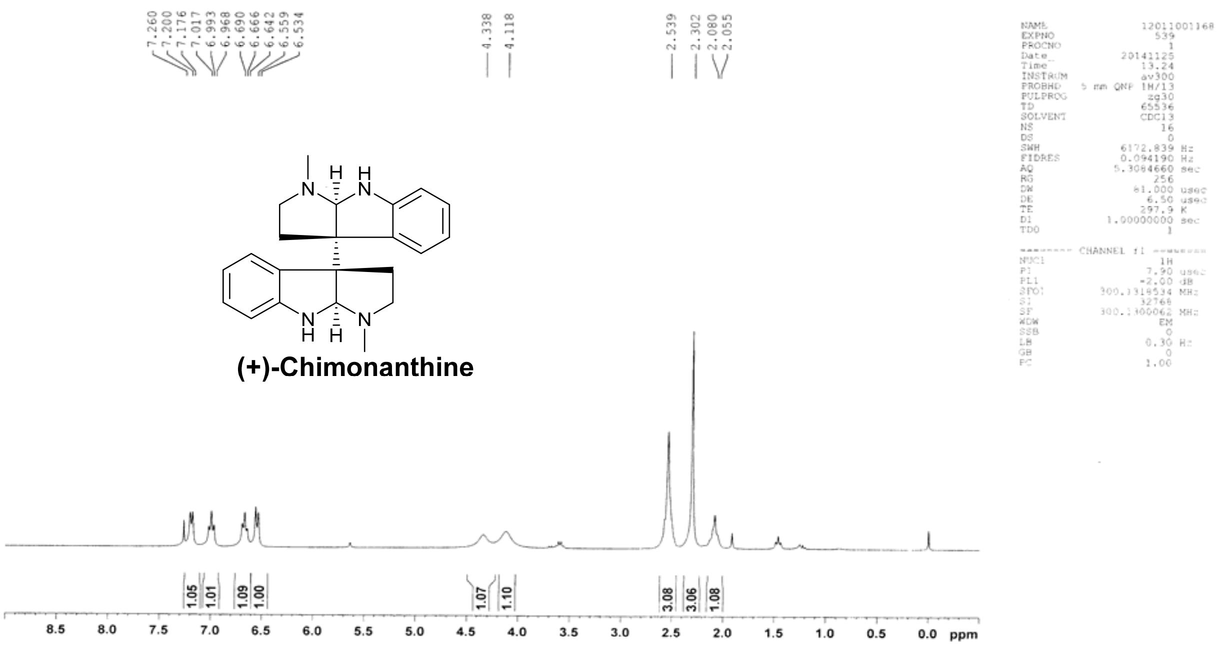
7.260
7.200
7.176
7.017
6.993
6.968
6.690
6.666
6.642
6.559
6.534
4.338
4.118
2.539
2.302
2.080
2.055
(+)-Chimonanthine
1.05
1.01
1.09
1.00
1.07
1.10
3.08
3.06
1.08
8.5 8.0 7.5 7.0 6.5 6.0 5.5 5.0 4.5 4.0 3.5 3.0 2.5 2.0 1.5 1.0 0.5 0.0 ppm

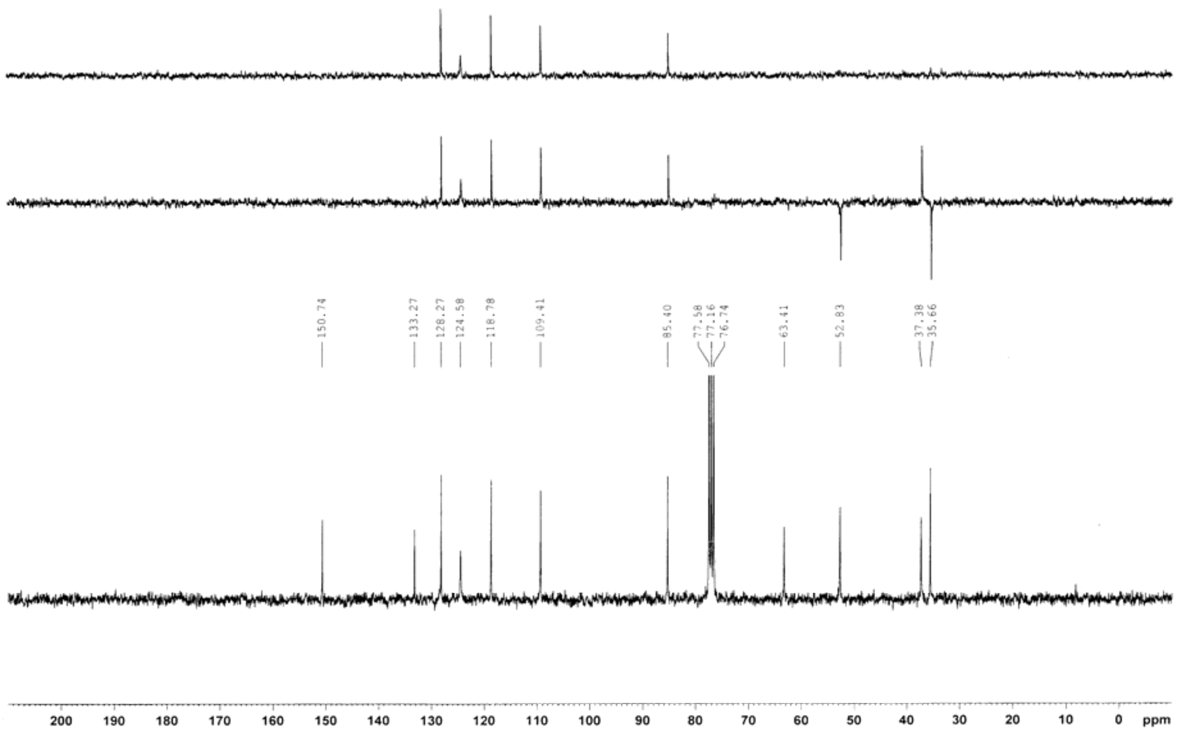
150.74
133.27
128.27
124.58
118.78
109.41
85.40
77.58
77.16
76.74
63.41
52.83
37.38
35.66
200 190 180 170 160 150 140 130 120 110 100 90 80 70 60 50 40 30 20 10 0 ppm

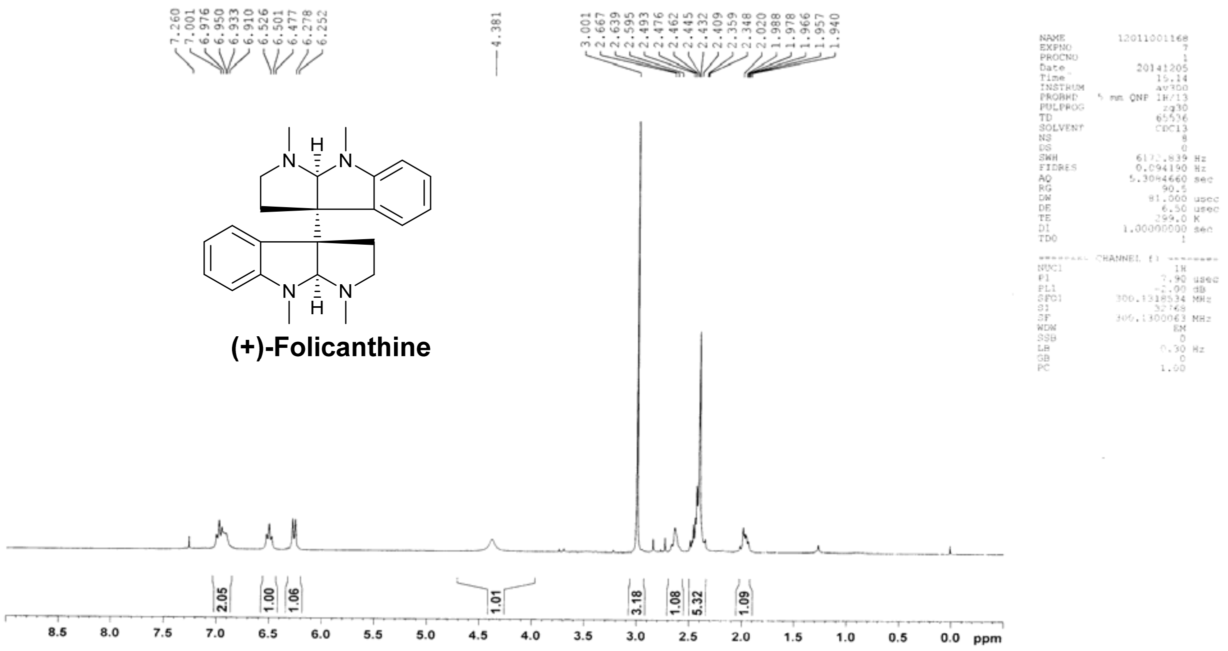

7.260
7.001
6.976
6.950
6.933
6.910
6.526
6.501
6.477
6.278
6.252
4.381
3.001
2.667
2.639
2.595
2.493
2.476
2.462
2.445
2.432
2.409
2.359
2.348
2.020
1.988
1.978
1.966
1.957
1.940
H
N
N
H
(+)-Folicanthine
2.05
1.00
1.06
1.01
3.18
1.08
5.32
1.09
8.5 8.0 7.5 7.0 6.5 6.0 5.5 5.0 4.5 4.0 3.5 3.0 2.5 2.0 1.5 1.0 0.5 0.0 ppm

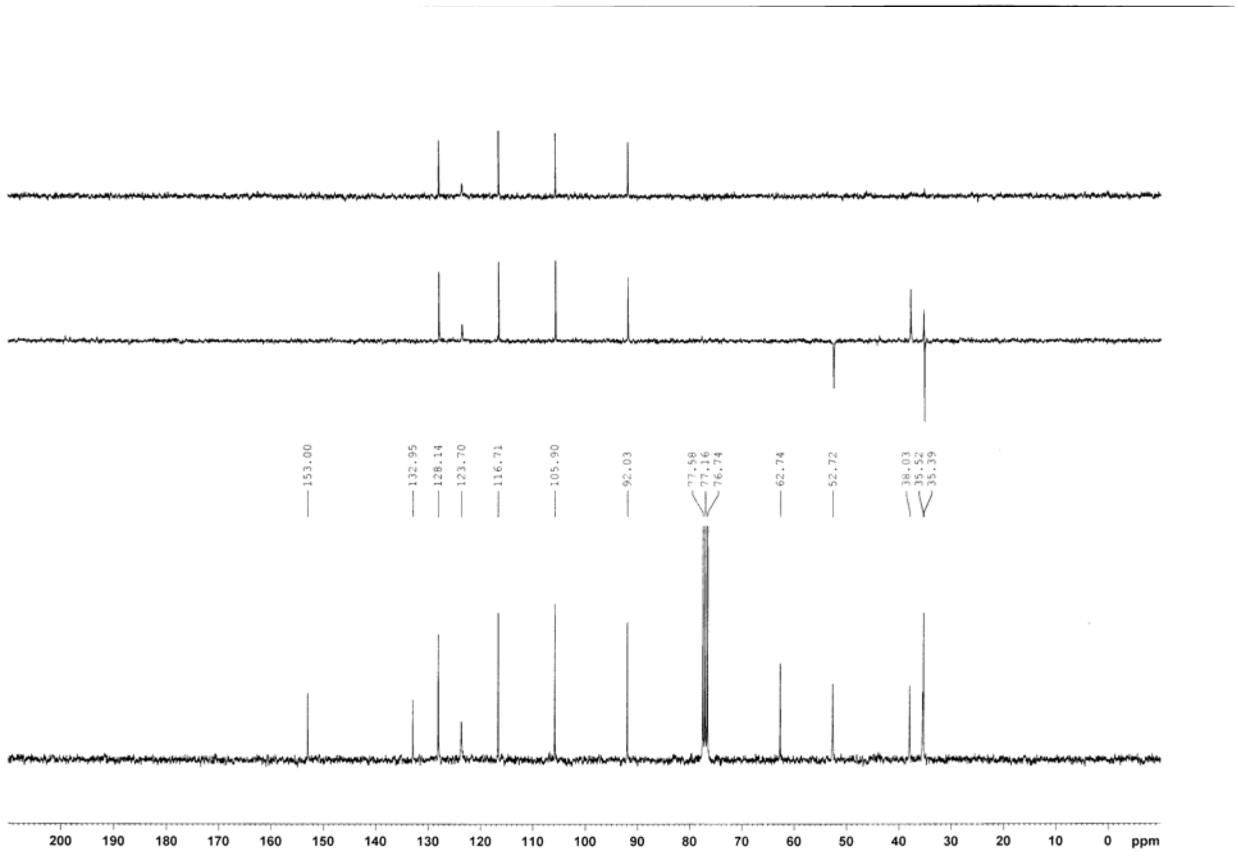

153.00
132.95
128.14
123.70
116.71
105.90
92.03
77.58
77.16
76.74
62.74
52.72
38.03
35.52
35.39
200 190 180 170 160 150 140 130 120 110 100 90 80 70 60 50 40 30 20 10 0 ppm

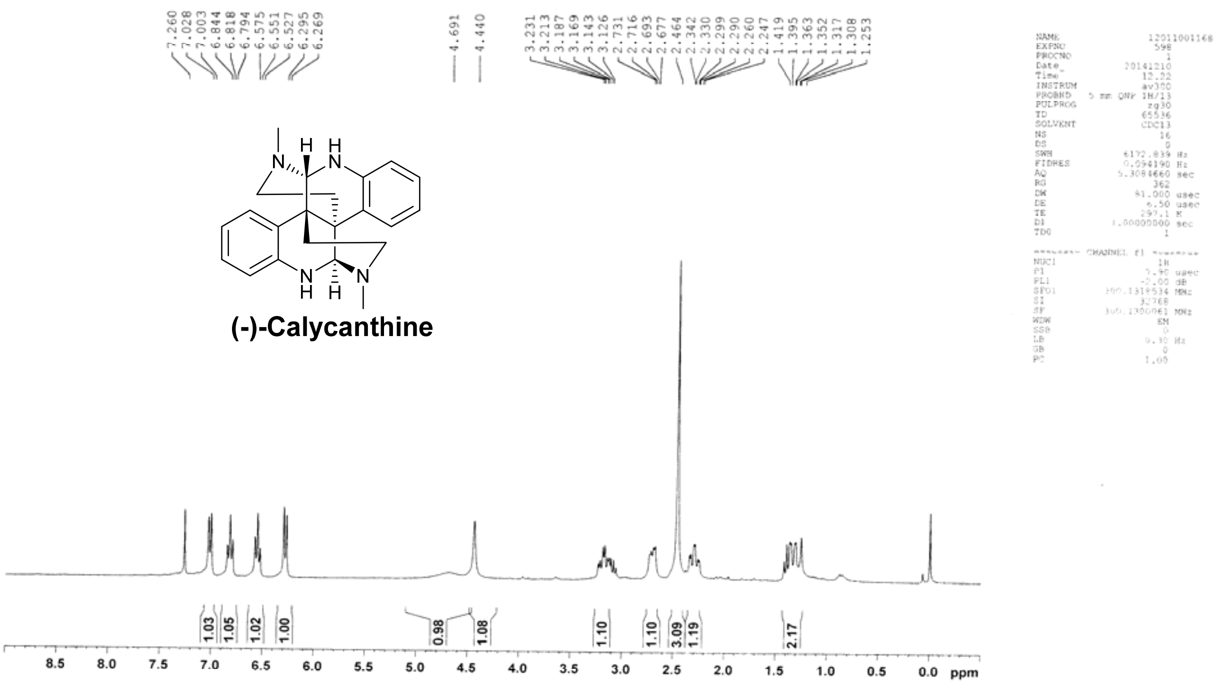
7.260
7.028
7.003
6.844
6.818
6.794
6.575
6.551
6.527
6.295
6.269
4.691
4.440
3.231
3.213
3.187
3.169
3.143
3.126
2.731
2.716
2.693
2.677
2.464
2.342
2.330
2.299
2.290
2.260
2.247
1.419
1.395
1.363
1.352
1.317
1.308
1.253
(-)-Calycanthine
ppm

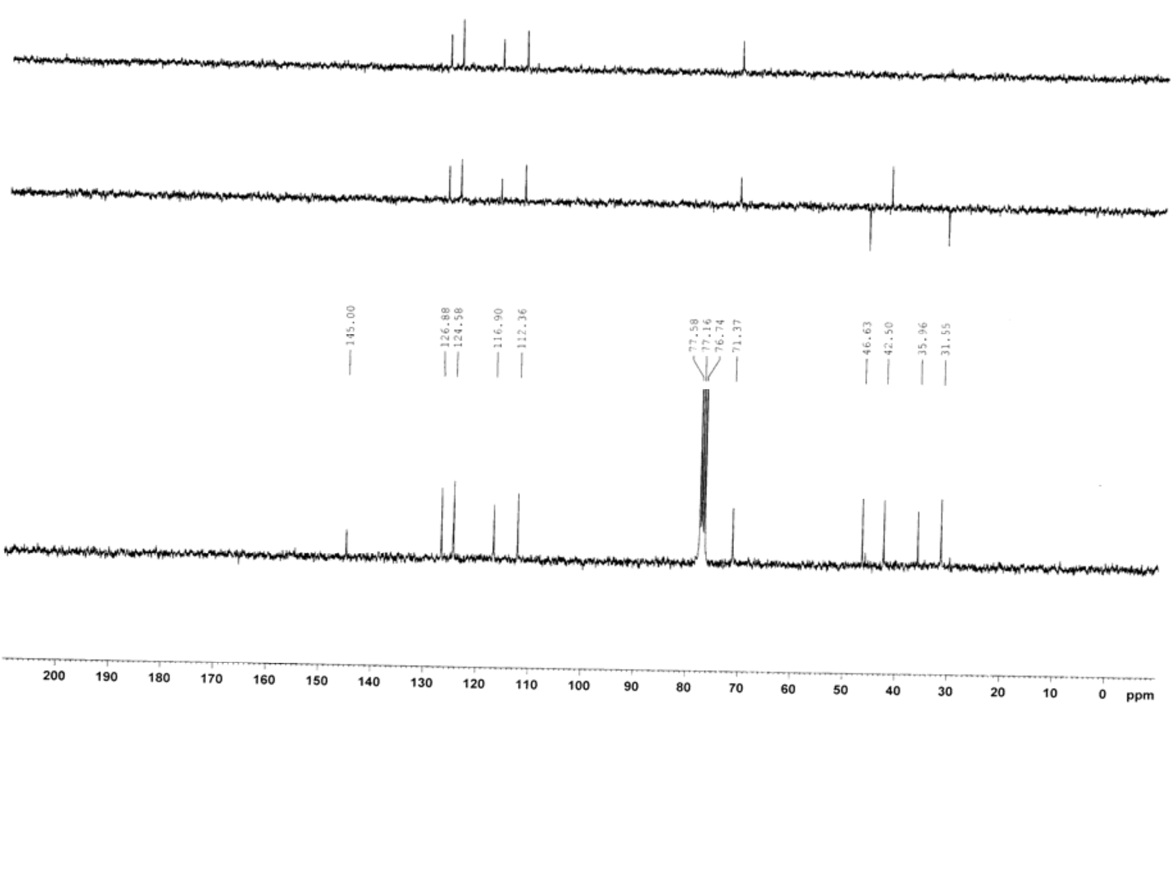
145.00
126.88
124.58
116.90
112.36
77.58
77.16
76.74
71.37
46.63
42.50
35.96
31.55
ppm

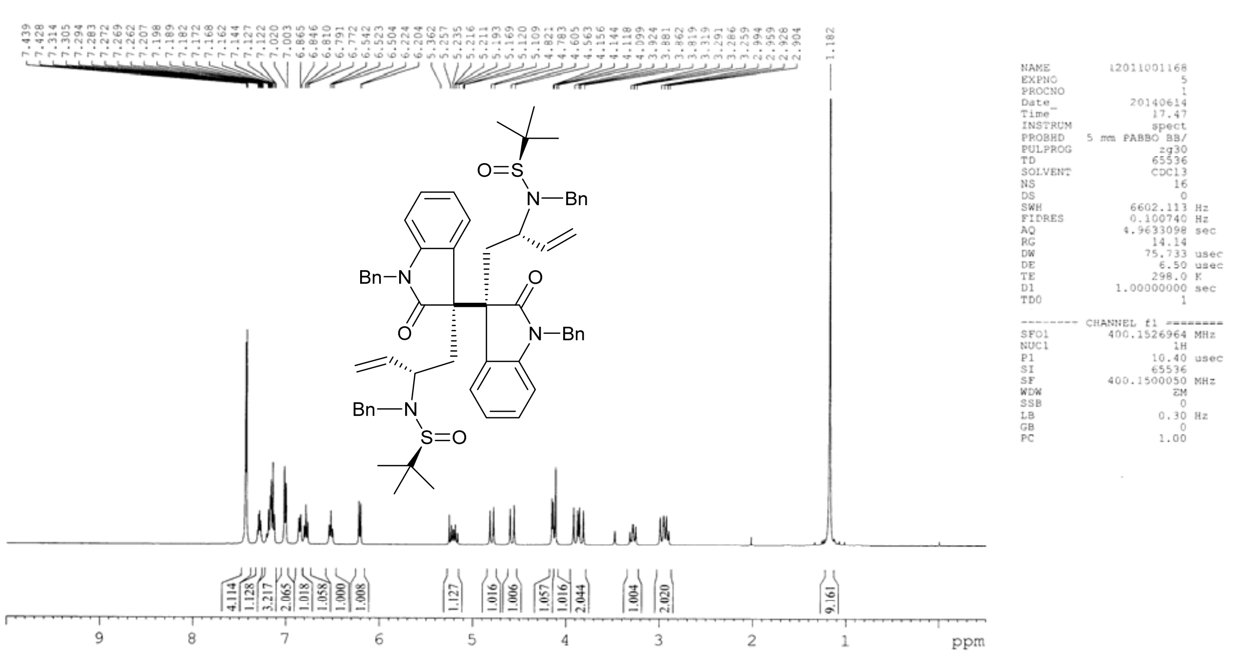
O=S
N—Bn
Bn—N
O
O
N—Bn
Bn—N
S=O
4.114
1.128
3.217
2.065
1.018
1.058
1.000
1.008
1.127
1.016
1.006
1.057
1.016
2.044
1.004
2.020
9.161
9 8 7 6 5 4 3 2 1 ppm
NAME 12011001168
EXPNO 5
PROCNO 1
Date_ 20140614
Time 17.47
INSTRUM spect
PROBHD 5 mm PABBO BB/
PULPROG zg30
TD 65536
SOLVENT CDCl3
NS 16
DS 0
SWH 6602.113 Hz
FIDRES 0.100740 Hz
AQ 4.9633098 sec
RG 14.14
DW 75.733 usec
DE 6.50 usec
TE 298.0 K
D1 1.00000000 sec
TD0 1
======== CHANNEL f1 ========
SFO1 400.1526964 MHz
NUC1 1H
P1 10.40 usec
SI 65536
SF 400.1500050 MHz
WDW EM
SSB 0
LB 0.30 Hz
GB 0
PC 1.00

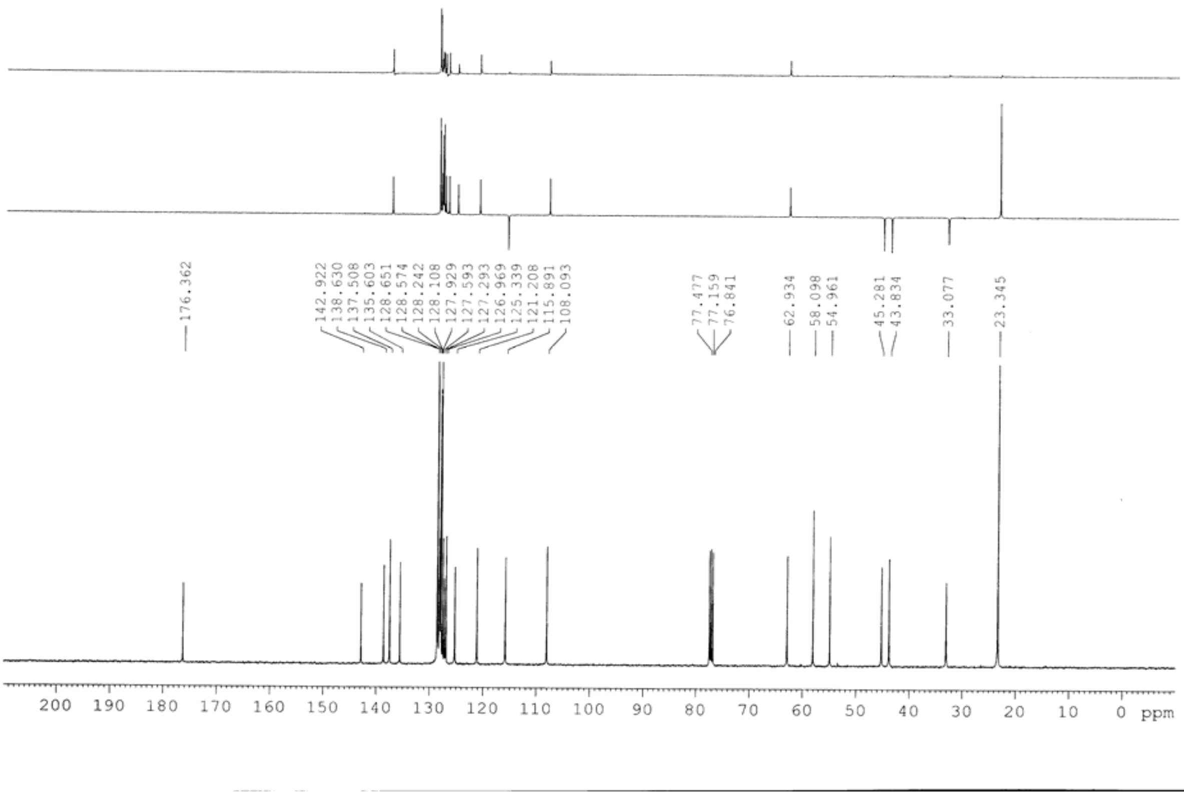
176.362
142.922
138.630
137.508
135.603
128.651
128.574
128.242
128.108
127.929
127.593
127.293
126.969
125.339
121.208
115.891
108.093
77.477
77.159
76.841
62.934
58.098
54.961
45.281
43.834
33.077
23.345
200 190 180 170 160 150 140 130 120 110 100 90 80 70 60 50 40 30 20 10 0 ppm

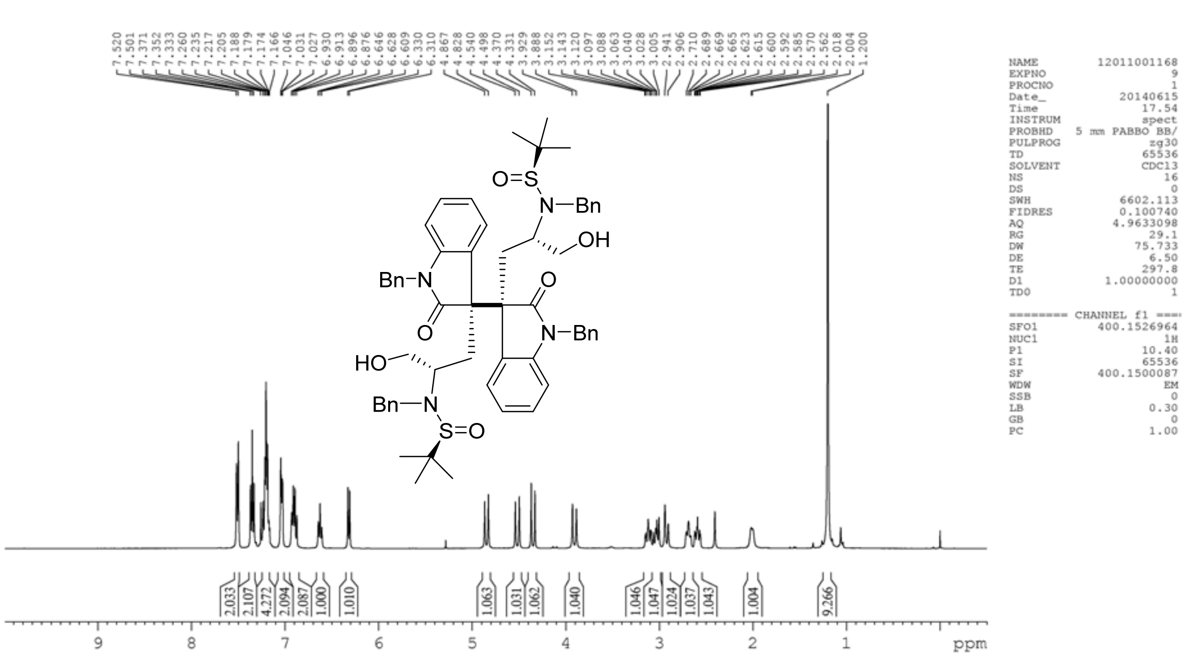
7.520 7.501 7.371 7.352 7.333 7.260 7.235 7.217 7.205 7.188 7.179 7.174 7.166 7.046 7.031 7.027 6.930 6.913 6.896 6.876 6.646 6.628 6.609 6.330 6.310 4.867 4.828 4.540 4.498 4.370 4.331 3.929 3.888 3.152 3.143 3.120 3.097 3.088 3.063 3.040 3.028 3.005 2.941 2.906 2.710 2.689 2.669 2.665 2.623 2.615 2.600 2.592 2.585 2.570 2.562 2.018 2.004 1.200
O=S
N−Bn
OH
Bn−N
O
O
N−Bn
HO
Bn−N
S=O
2.033 2.107 4.272 2.094 2.087 1.000 1.010 1.063 1.031 1.062 1.040 1.046 1.047 1.024 1.037 1.043 1.004 9.266
9 8 7 6 5 4 3 2 1 ppm
NAME 12011001168
EXPNO 9
PROCNO 1
Date_ 20140615
Time 17.54
INSTRUM spect
PROBHD 5 mm PABBO BB/
PULPROG zg30
TD 65536
SOLVENT CDCl3
NS 16
DS 0
SWH 6602.113
FIDRES 0.100740
AQ 4.9633098
RG 29.1
DW 75.733
DE 6.50
TE 297.8
D1 1.00000000
TD0 1
======== CHANNEL f1 ====
SFO1 400.1526964
NUC1 1H
P1 10.40
SI 65536
SF 400.1500087
WDW EM
SSB 0
LB 0.30
GB 0
PC 1.00

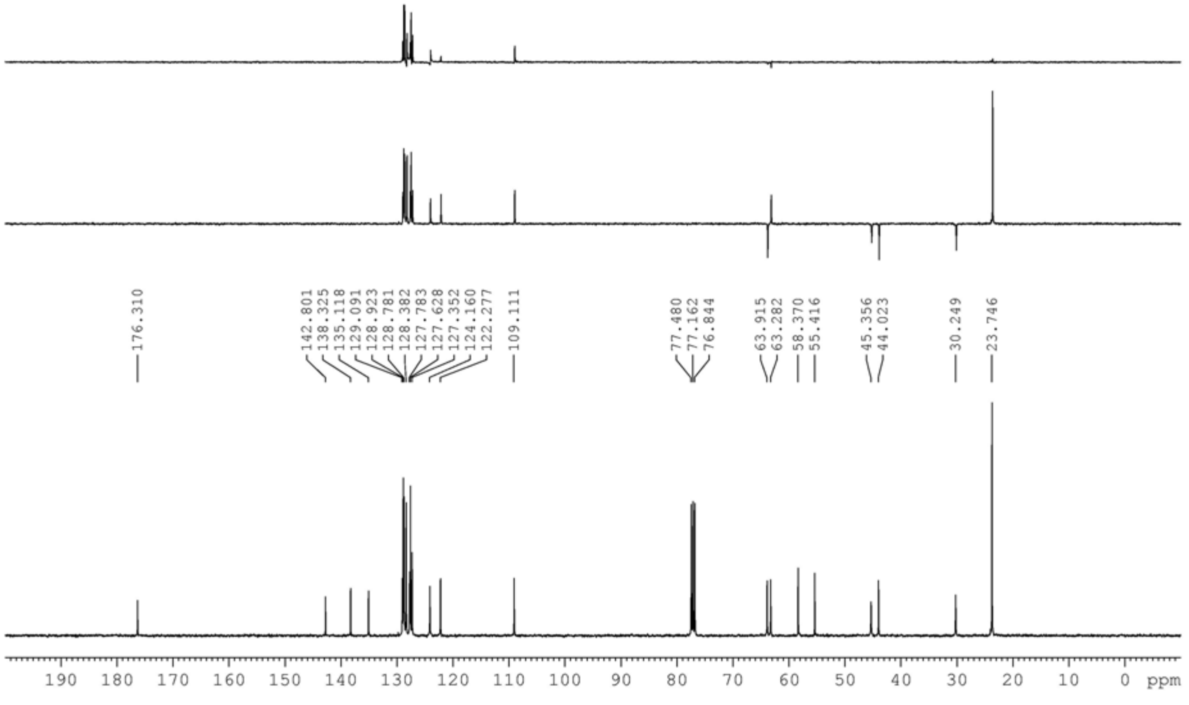
176.310
142.801 138.325 135.118 129.091 128.923 128.781 128.382 127.783 127.628 127.352 124.160 122.277
109.111
77.480 77.162 76.844
63.915 63.282 58.370 55.416
45.356 44.023
30.249 23.746
190 180 170 160 150 140 130 120 110 100 90 80 70 60 50 40 30 20 10 0 ppm

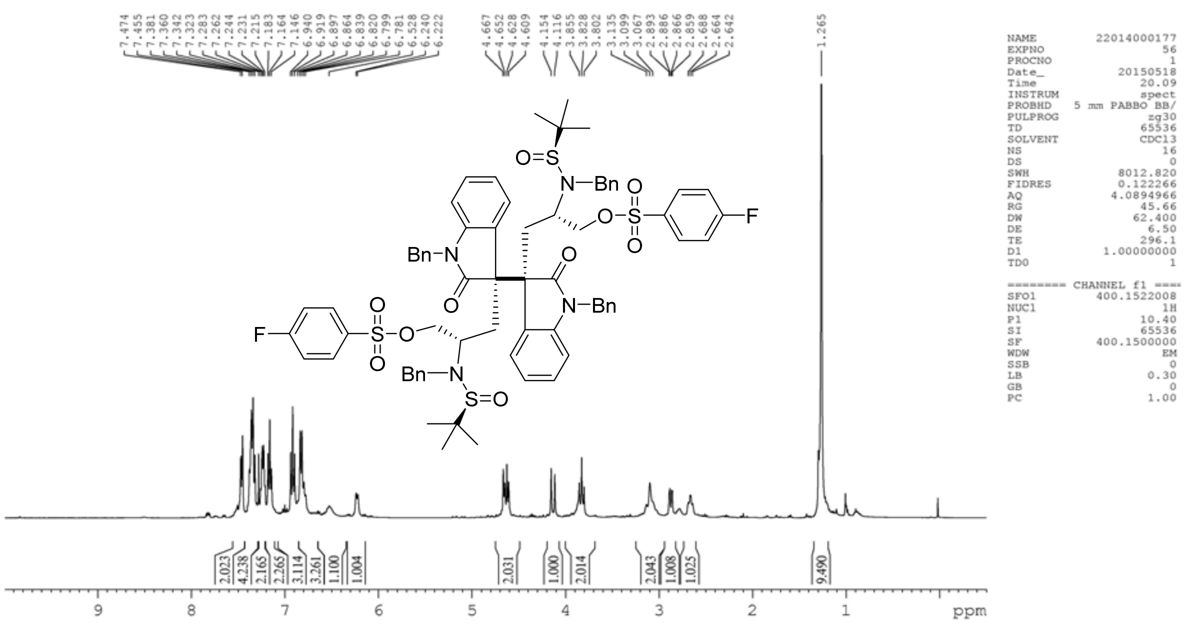
NAME 22014000177
EXPNO 56
PROCNO 1
Date_ 20150518
Time 20.09
INSTRUM spect
PROBHD 5 mm PABBO BB/
PULPROG zg30
TD 65536
SOLVENT CDCl3
NS 16
DS 0
SWH 8012.820
FIDRES 0.122266
AQ 4.0894966
RG 45.66
DW 62.400
DE 6.50
TE 296.1
D1 1.00000000
TD0 1
======== CHANNEL f1 ========
SFO1 400.1522008
NUC1 1H
P1 10.40
SI 65536
SF 400.1500000
WDW EM
SSB 0
LB 0.30
GB 0
PC 1.00
ppm

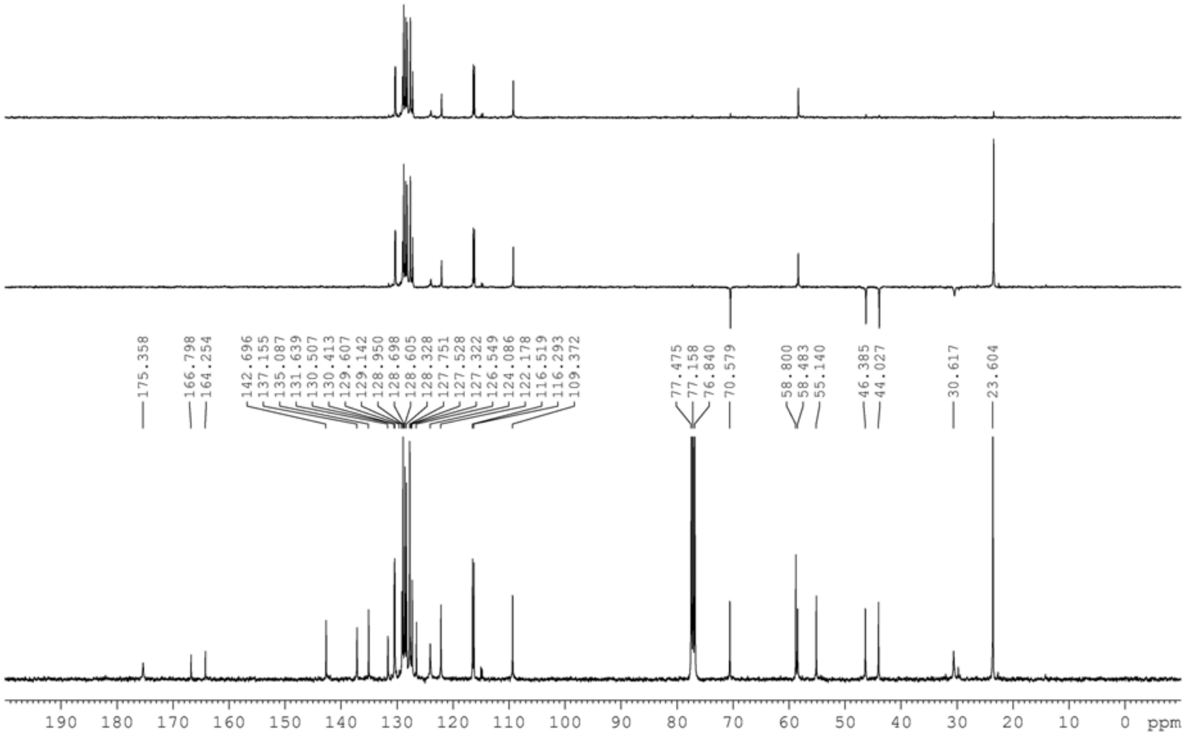
175.358
166.798
164.254
142.696
137.155
135.087
131.639
130.507
130.413
129.607
129.142
128.950
128.698
128.605
128.328
127.751
127.528
127.322
126.549
124.086
122.178
116.519
116.293
109.372
77.475
77.158
76.840
70.579
58.800
58.483
55.140
46.385
44.027
30.617
23.604
ppm

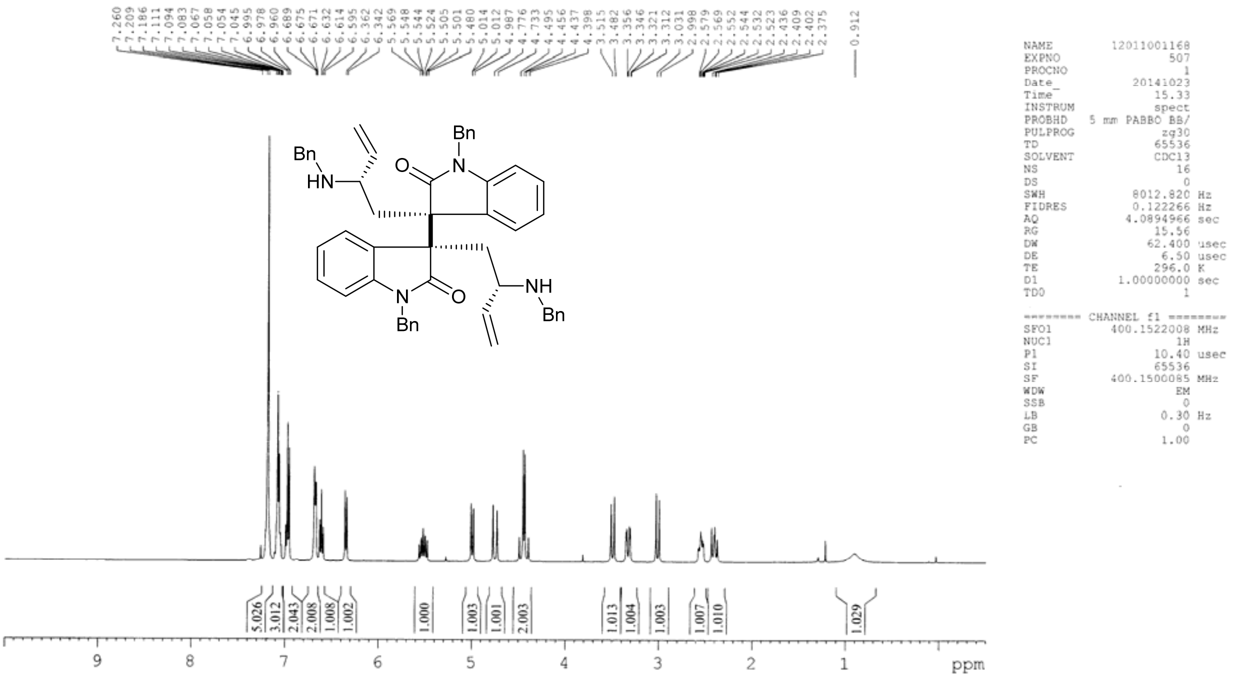

7.260 7.209 7.186 7.111 7.094 7.083 7.067 7.058 7.054 7.045 6.995 6.978 6.960 6.689 6.675 6.671 6.632 6.614 6.595 6.362 6.342 5.569 5.548 5.544 5.524 5.505 5.501 5.480 5.014 5.012 4.987 4.776 4.733 4.495 4.456 4.437 4.398 3.515 3.482 3.356 3.346 3.321 3.312 3.031 2.998 2.579 2.569 2.552 2.544 2.532 2.523 2.436 2.409 2.402 2.375 0.912
Bn
Bn
HN
O
N
NH
N
O
Bn
Bn
NAME 12011001168
EXPNO 507
PROCNO 1
Date_ 20141023
Time 15.33
INSTRUM spect
PROBHD 5 mm PABBO BB/
PULPROG zg30
TD 65536
SOLVENT CDCl3
NS 16
DS 0
SWH 8012.820 Hz
FIDRES 0.122266 Hz
AQ 4.0894966 sec
RG 15.56
DW 62.400 usec
DE 6.50 usec
TE 296.0 K
D1 1.00000000 sec
TD0 1
======== CHANNEL f1 ========
SFO1 400.1522008 MHz
NUC1 1H
P1 10.40 usec
SI 65536
SF 400.1500085 MHz
WDW EM
SSB 0
LB 0.30 Hz
GB 0
PC 1.00
5.026 3.012 2.043 2.008 1.008 1.002 1.000 1.003 1.001 2.003 1.013 1.004 1.003 1.007 1.010 1.029
9 8 7 6 5 4 3 2 1 ppm

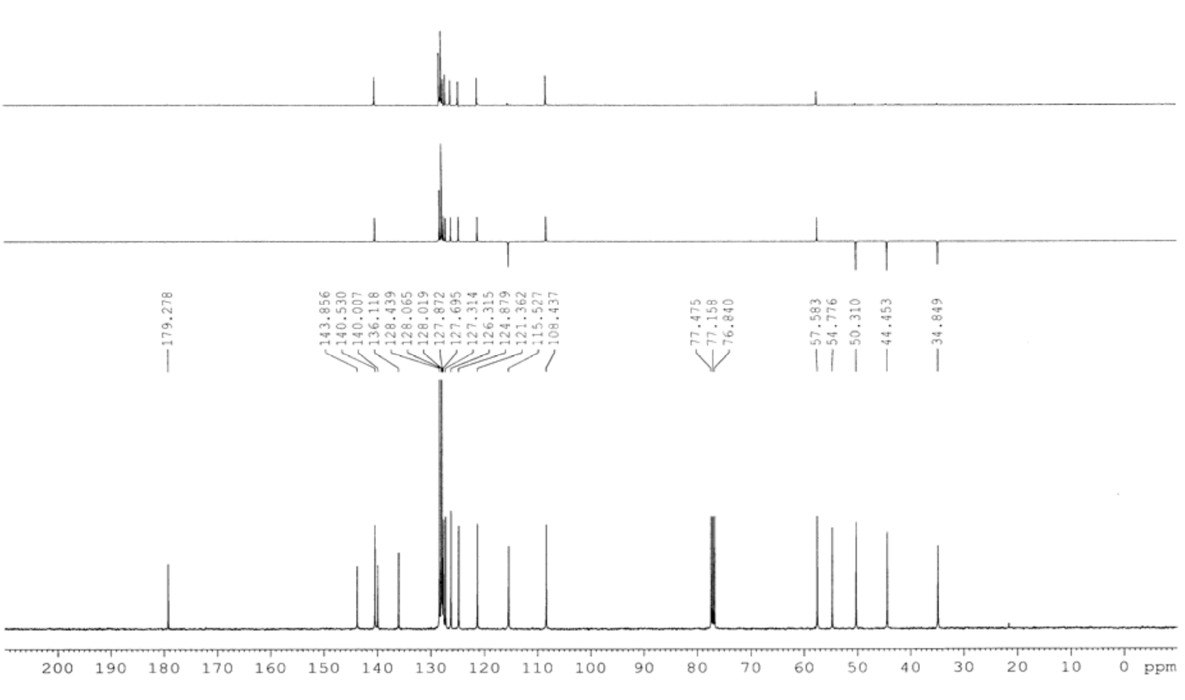

179.278
143.856 140.530 140.007 136.118 128.439 128.065 128.019 127.872 127.695 127.314 126.315 124.879 121.362 115.527 108.437
77.475 77.158 76.840
57.583 54.776 50.310 44.453 34.849
200 190 180 170 160 150 140 130 120 110 100 90 80 70 60 50 40 30 20 10 0 ppm

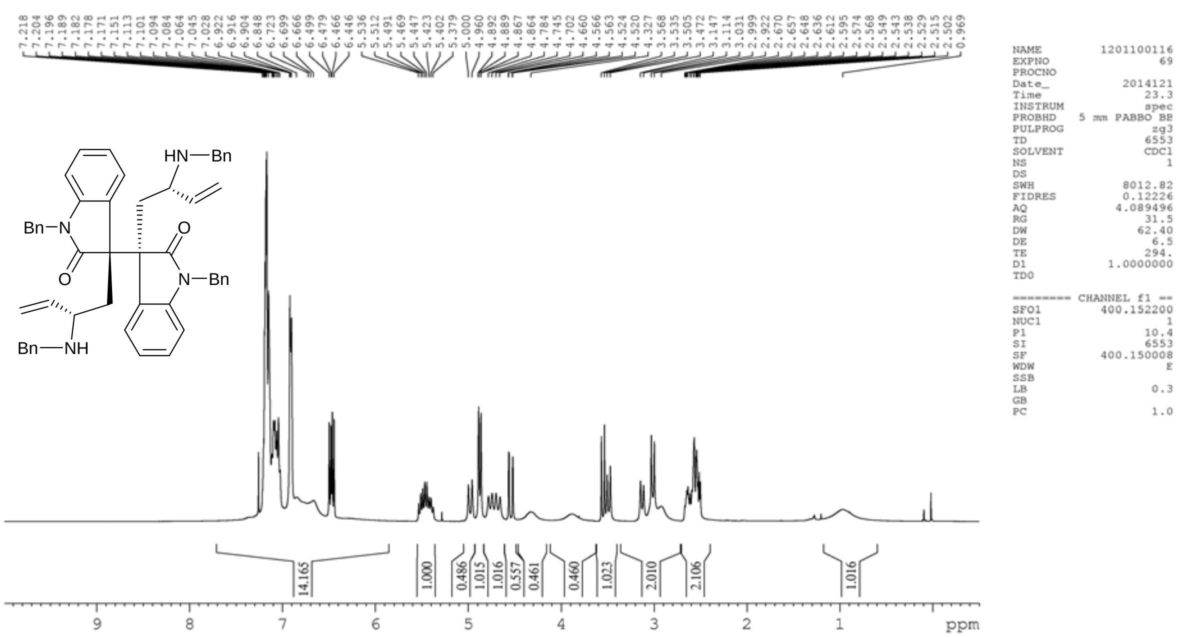
7.218 7.204 7.196 7.189 7.182 7.178 7.171 7.151 7.113 7.101 7.094 7.084 7.064 7.045 7.028 6.922 6.916 6.904 6.848 6.723 6.699 6.666 6.499 6.479 6.466 6.446 5.536 5.512 5.491 5.469 5.447 5.423 5.402 5.379 5.000 4.960 4.892 4.889 4.867 4.864 4.784 4.745 4.702 4.660 4.566 4.563 4.524 4.520 4.327 3.568 3.535 3.505 3.472 3.147 3.114 3.031 2.999 2.922 2.670 2.657 2.648 2.636 2.612 2.595 2.574 2.568 2.549 2.543 2.538 2.529 2.515 2.502 0.969
HN—Bn
Bn—N
O
O
N—Bn
Bn—NH
14.165
1.000
0.486
1.015
1.016
0.557
0.461
0.460
1.023
2.010
2.106
1.016
9 8 7 6 5 4 3 2 1 ppm
NAME 1201100116
EXPNO 69
PROCNO
Date_ 2014121
Time 23.3
INSTRUM spec
PROBHD 5 mm PABBO BB
PULPROG zg3
TD 6553
SOLVENT CDCl
NS 1
DS
SWH 8012.82
FIDRES 0.12226
AQ 4.089496
RG 31.5
DW 62.40
DE 6.5
TE 294.
D1 1.0000000
TD0
======== CHANNEL f1 ==
SFO1 400.152200
NUC1 1
P1 10.4
SI 6553
SF 400.150008
WDW E
SSB
LB 0.3
GB
PC 1.0

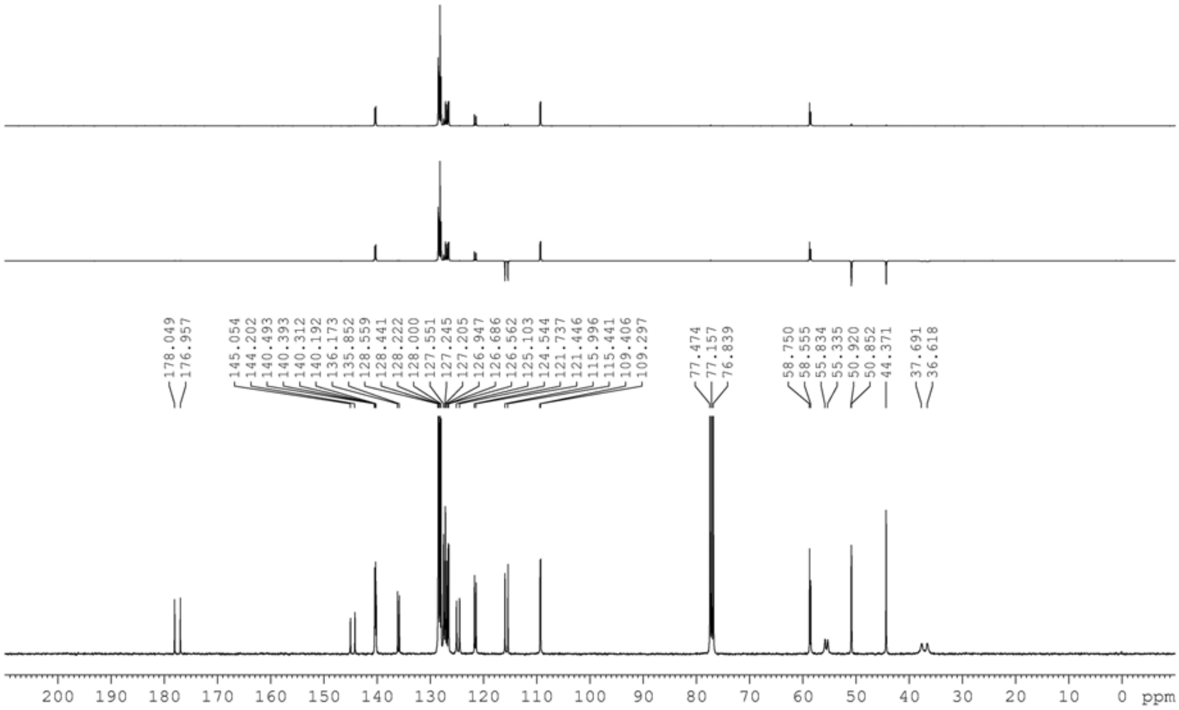
178.049 176.957
145.054 144.202 140.493 140.393 140.312 140.192 136.173 135.852 128.559 128.441 128.222 128.000 127.551 127.245 127.205 126.947 126.686 126.562 125.103 124.544 121.737 121.446 115.996 115.441 109.406 109.297
77.474 77.157 76.839
58.750 58.555 55.834 55.335 50.920 50.852 44.371
37.691 36.618
200 190 180 170 160 150 140 130 120 110 100 90 80 70 60 50 40 30 20 10 0 ppm

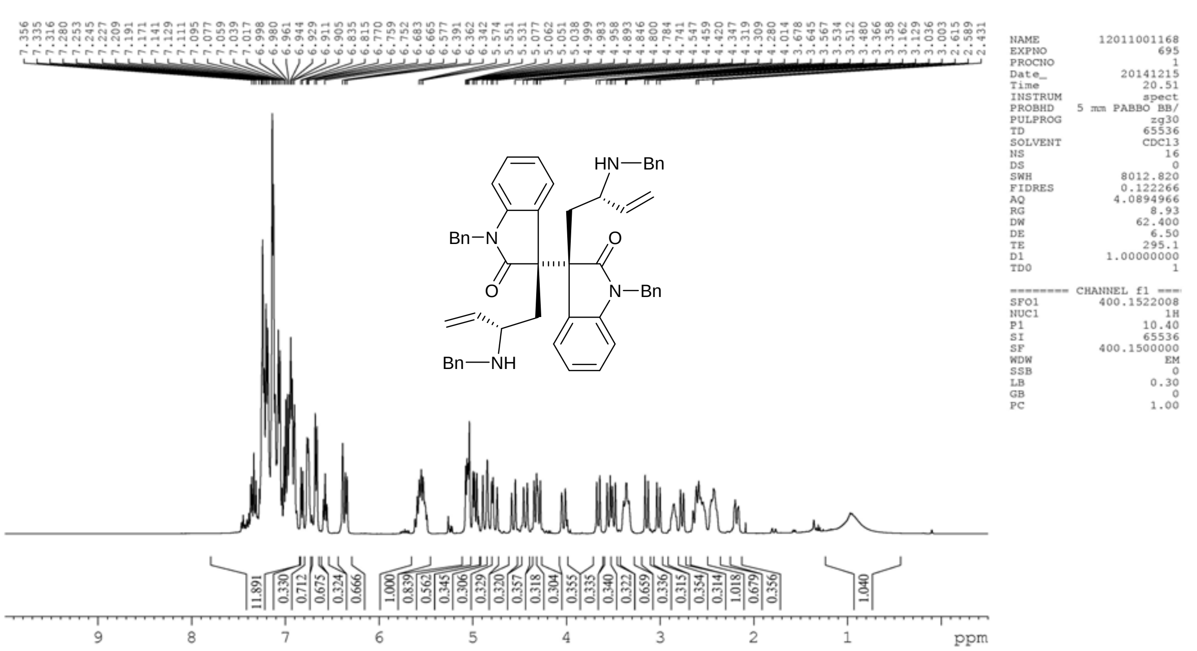
7.356 7.335 7.316 7.280 7.253 7.245 7.227 7.209 7.191 7.171 7.141 7.129 7.111 7.095 7.077 7.059 7.039 7.017 6.998 6.980 6.961 6.944 6.929 6.911 6.905 6.835 6.815 6.770 6.759 6.752 6.683 6.665 6.577 6.391 6.362 6.342 5.574 5.551 5.531 5.077 5.062 5.051 5.038 4.999 4.983 4.958 4.893 4.846 4.800 4.784 4.741 4.547 4.459 4.420 4.347 4.319 4.309 4.280 4.014 3.678 3.645 3.567 3.534 3.512 3.480 3.366 3.358 3.162 3.129 3.036 3.003 2.615 2.589 2.431
HN—Bn
Bn—N
O
O
N—Bn
Bn—NH
11.891 0.330 0.712 0.675 0.324 0.666 1.000 0.839 0.562 0.345 0.306 0.329 0.320 0.357 0.318 0.304 0.355 0.335 0.340 0.322 0.659 0.336 0.315 0.354 0.314 1.018 0.679 0.356 1.040
9 8 7 6 5 4 3 2 1 ppm
NAME 12011001168
EXPNO 695
PROCNO 1
Date_ 20141215
Time 20.51
INSTRUM spect
PROBHD 5 mm PABBO BB/
PULPROG zg30
TD 65536
SOLVENT CDCl3
NS 16
DS 0
SWH 8012.820
FIDRES 0.122266
AQ 4.0894966
RG 8.93
DW 62.400
DE 6.50
TE 295.1
D1 1.00000000
TD0 1
======== CHANNEL f1 ========
SFO1 400.1522008
NUC1 1H
P1 10.40
SI 65536
SF 400.1500000
WDW EM
SSB 0
LB 0.30
GB 0
PC 1.00

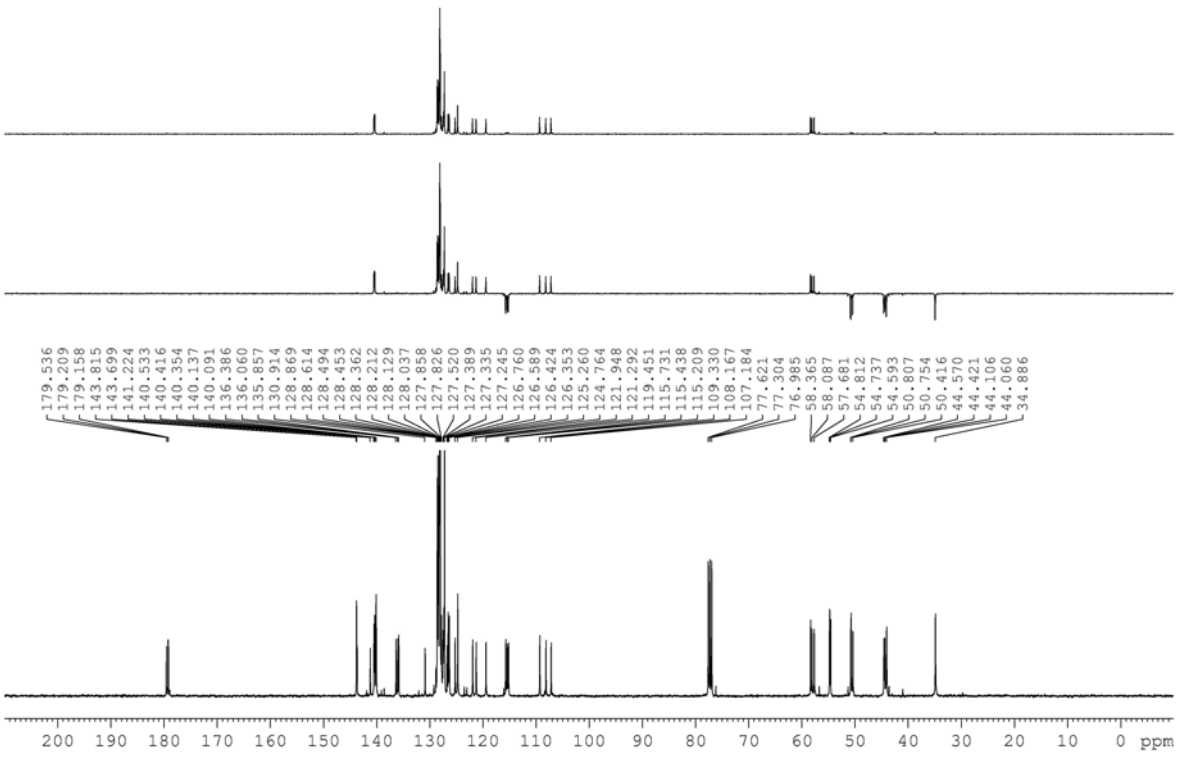
179.536 179.209 179.158 143.815 143.699 141.224 140.533 140.416 140.354 140.137 140.091 136.386 136.060 135.857 130.914 128.869 128.614 128.494 128.453 128.362 128.212 128.129 128.037 127.858 127.826 127.520 127.389 127.335 127.245 126.760 126.589 126.424 126.353 125.260 124.764 121.948 121.292 119.451 115.731 115.438 115.209 109.330 108.167 107.184 77.621 77.304 76.985 58.365 58.087 57.681 54.812 54.737 54.593 50.807 50.754 50.416 44.570 44.421 44.106 44.060 34.886
200 190 180 170 160 150 140 130 120 110 100 90 80 70 60 50 40 30 20 10 0 ppm

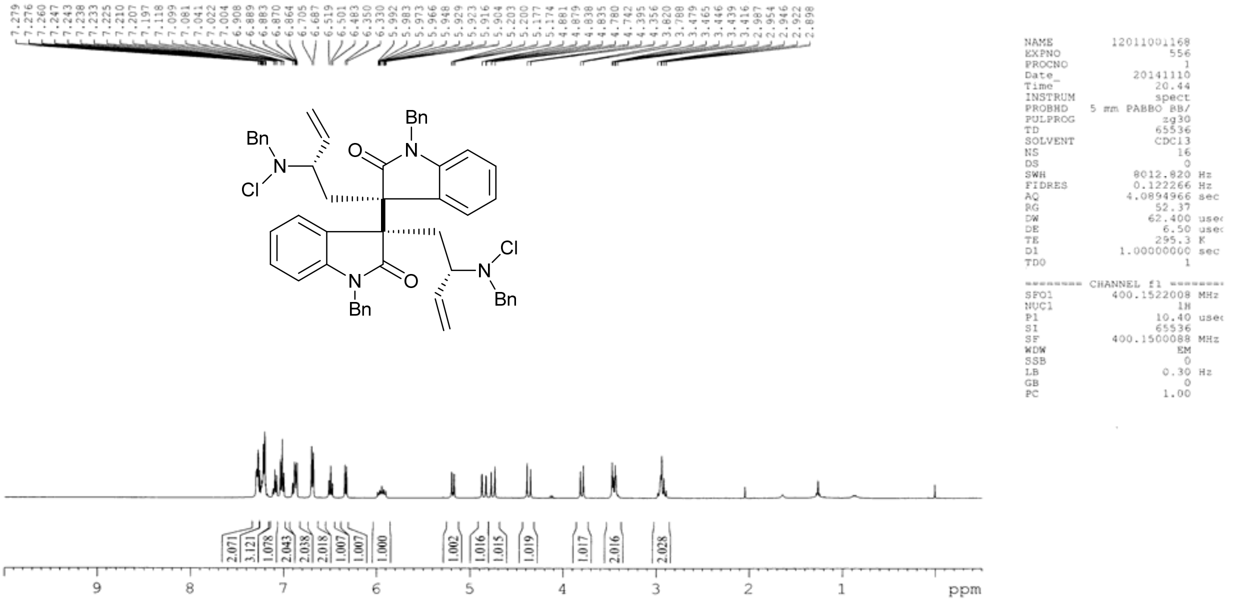

7.279 7.276 7.260 7.247 7.243 7.238 7.233 7.225 7.210 7.207 7.197 7.118 7.099 7.081 7.041 7.022 7.004 6.908 6.889 6.883 6.870 6.864 6.705 6.687 6.519 6.501 6.483 6.350 6.330 5.992 5.983 5.973 5.966 5.948 5.929 5.923 5.916 5.904 5.203 5.200 5.177 5.174 4.881 4.879 4.838 4.835 4.780 4.742 4.395 4.356 3.820 3.780 3.479 3.465 3.446 3.439 3.416 2.987 2.954 2.946 2.922 2.898
Bn
Bn
N
Cl
O
N
O
N
Cl
N
Bn
Bn
NAME 12011001168
EXPNO 556
PROCNO 1
Date_ 20141110
Time 20.44
INSTRUM spect
PROBHD 5 mm PABBO BB/
PULPROG zg30
TD 65536
SOLVENT CDCl3
NS 16
DS 0
SWH 8012.820 Hz
FIDRES 0.122266 Hz
AQ 4.0894966 sec
RG 52.37
DW 62.400 usec
DE 6.50 usec
TE 295.3 K
D1 1.00000000 sec
TD0 1
======== CHANNEL f1 ========
SFO1 400.1522008 MHz
NUC1 1H
P1 10.40 usec
SI 65536
SF 400.1500088 MHz
WDW EM
SSB 0
LB 0.30 Hz
GB 0
PC 1.00
2.071 3.121 1.078 2.043 2.038 2.018 1.007 1.007 1.000 1.002 1.016 1.015 1.019 1.017 2.016 2.028
9 8 7 6 5 4 3 2 1 ppm

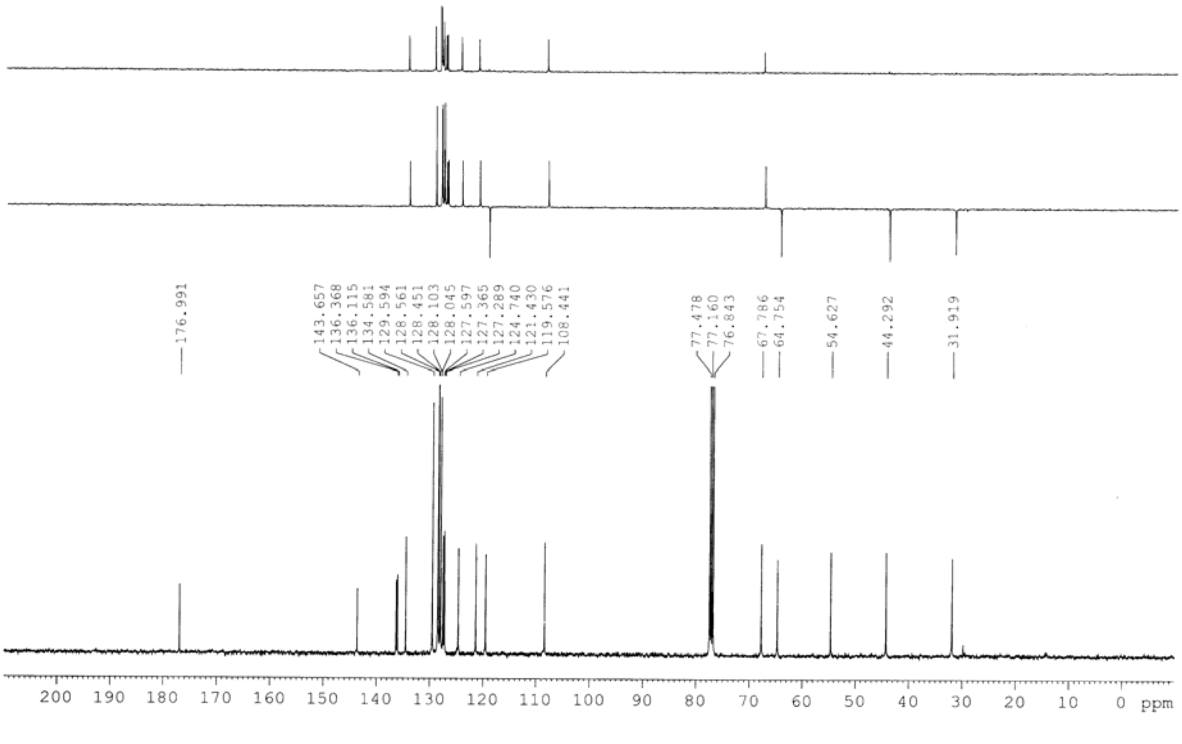

176.991
143.657 136.368 136.115 134.581 129.594 128.561 128.451 128.103 128.045 127.597 127.365 127.289 124.740 121.430 119.576 108.441
77.478 77.160 76.843
67.786 64.754
54.627
44.292
31.919
200 190 180 170 160 150 140 130 120 110 100 90 80 70 60 50 40 30 20 10 0 ppm

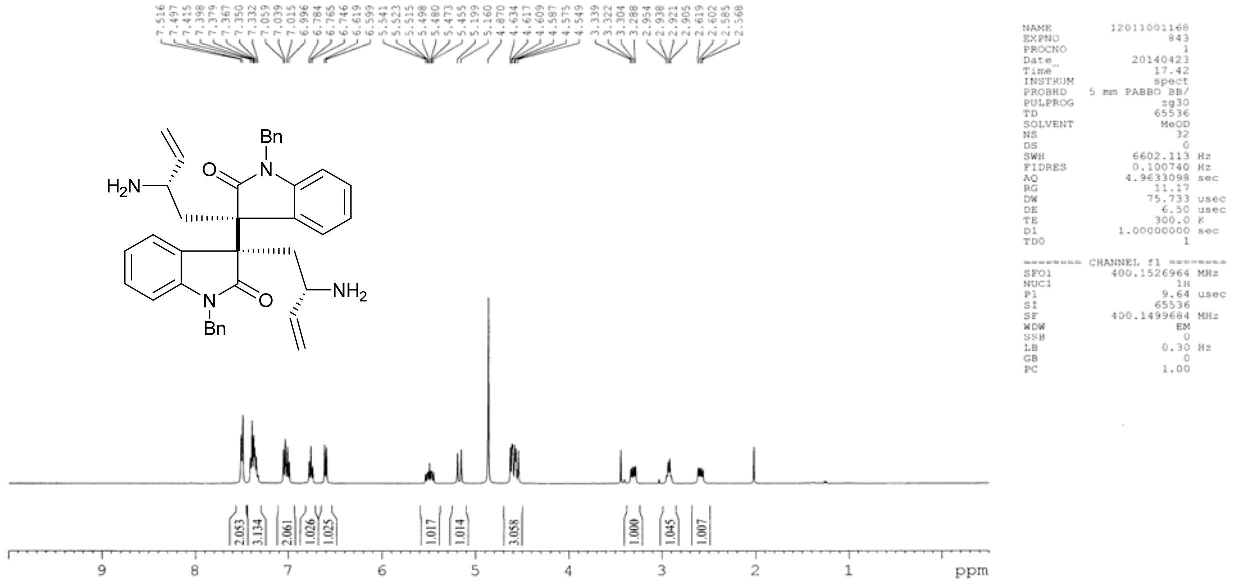
Bn
O
N
H2N
N
O
NH2
Bn
NAME 12011001168
EXPNO 843
PROCNO 1
Date_ 20140423
Time 17.42
INSTRUM spect
PROBHD 5 mm PABBO BB/
PULPROG zg30
TD 65536
SOLVENT MeOD
NS 32
DS 0
SWH 6602.113 Hz
FIDRES 0.100740 Hz
AQ 4.9633098 sec
RG 11.17
DW 75.733 usec
DE 6.50 usec
TE 300.0 K
D1 1.00000000 sec
TD0 1
======== CHANNEL f1 ========
SFO1 400.1526964 MHz
NUC1 1H
P1 9.64 usec
SI 65536
SF 400.1499684 MHz
WDW EM
SSB 0
LB 0.30 Hz
GB 0
PC 1.00
2.053
3.134
2.061
1.026
1.025
1.017
1.014
3.058
1.000
1.045
1.007
9 8 7 6 5 4 3 2 1 ppm

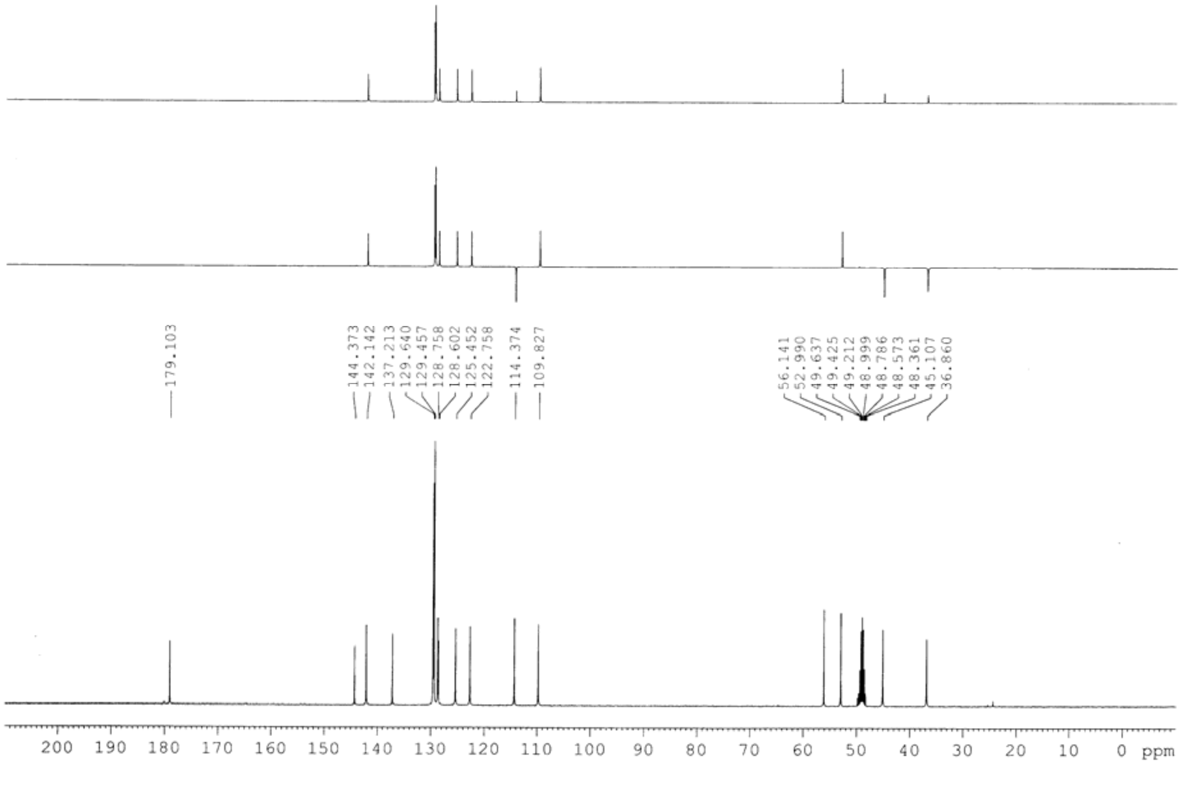
179.103
144.373
142.142
137.213
129.640
129.457
128.758
128.602
125.452
122.758
114.374
109.827
56.141
52.990
49.637
49.425
49.212
48.999
48.786
48.573
48.361
45.107
36.860
200 190 180 170 160 150 140 130 120 110 100 90 80 70 60 50 40 30 20 10 0 ppm

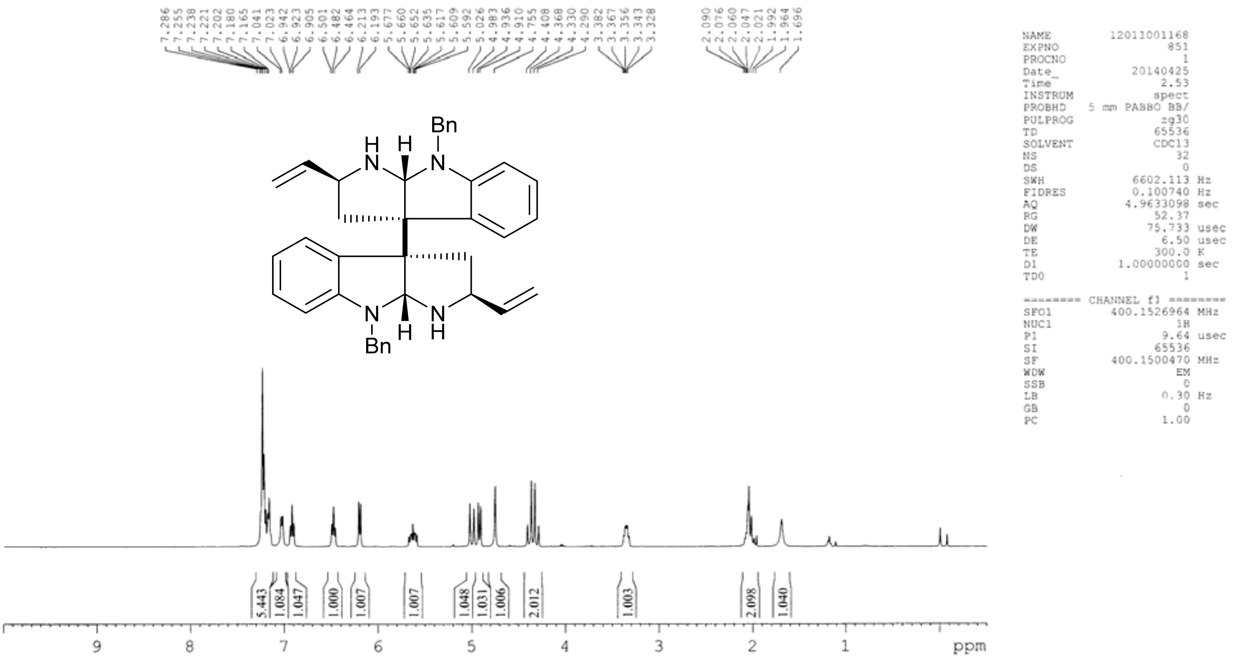
7.286
7.255
7.238
7.221
7.202
7.180
7.165
7.041
7.023
6.942
6.923
6.905
6.501
6.482
6.464
6.213
6.193
5.677
5.660
5.652
5.635
5.617
5.609
5.592
5.026
4.983
4.936
4.910
4.755
4.408
4.368
4.330
4.290
3.382
3.367
3.356
3.343
3.328
2.090
2.076
2.060
2.047
2.021
1.992
1.964
1.696
Bn
H
H
N
N
N
H
H
N
Bn
NAME 12011001168
EXPNO 851
PROCNO 1
Date_ 20140425
Time 2.53
INSTRUM spect
PROBHD 5 mm PABBO BB/
PULPROG zg30
TD 65536
SOLVENT CDCl3
NS 32
DS 0
SWH 6602.113 Hz
FIDRES 0.100740 Hz
AQ 4.9633098 sec
RG 52.37
DW 75.733 usec
DE 6.50 usec
TE 300.0 K
D1 1.00000000 sec
TD0 1
======== CHANNEL f1 ========
SFO1 400.1526964 MHz
NUC1 1H
P1 9.64 usec
SI 65536
SF 400.1500470 MHz
WDW EM
SSB 0
LB 0.30 Hz
GB 0
PC 1.00
5.443
1.084
1.047
1.000
1.007
1.007
1.048
1.031
1.006
2.012
1.003
2.098
1.040
9
8
7
6
5
4
3
2
1
ppm

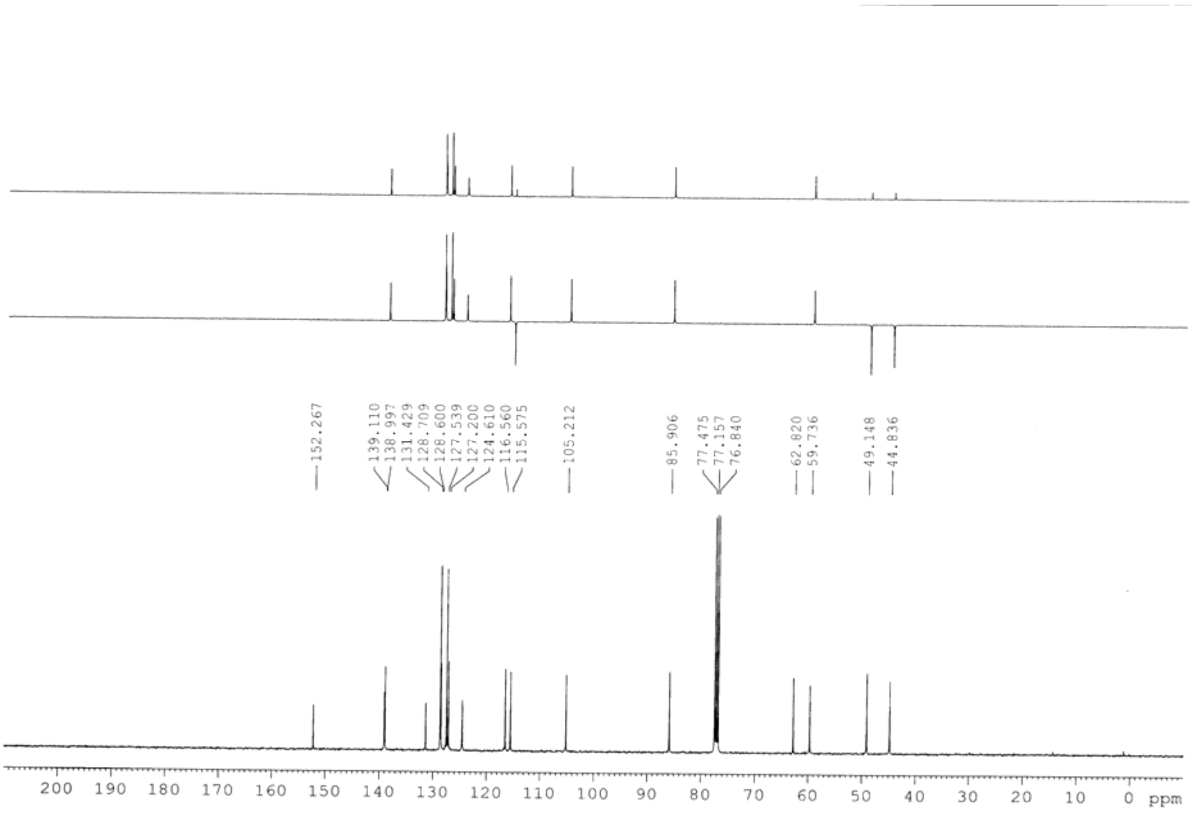
152.267
139.110
138.997
131.429
128.709
128.600
127.539
127.200
124.610
116.560
115.575
105.212
85.906
77.475
77.157
76.840
62.820
59.736
49.148
44.836
200
190
180
170
160
150
140
130
120
110
100
90
80
70
60
50
40
30
20
10
0
ppm

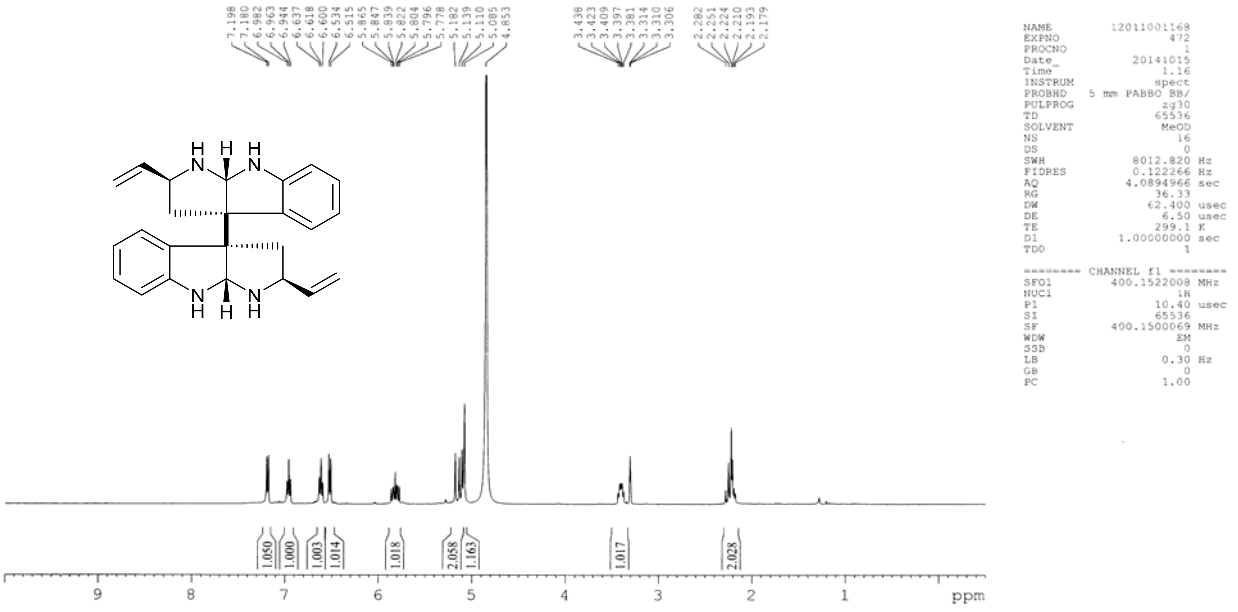
7.198
7.180
6.982
6.963
6.944
6.637
6.618
6.600
6.534
6.515
5.865
5.847
5.839
5.822
5.804
5.796
5.778
5.182
5.139
5.110
5.085
4.853
3.438
3.423
3.409
3.397
3.381
3.314
3.310
3.306
2.282
2.251
2.224
2.210
2.193
2.179
H
N
H
N
H
N
H
H
N
H
1.050
1.000
1.003
1.014
1.018
2.058
1.163
1.017
2.028
9
8
7
6
5
4
3
2
1
ppm
NAME 12011001168
EXPNO 472
PROCNO 1
Date_ 20141015
Time 1.16
INSTRUM spect
PROBHD 5 mm PABBO BB/
PULPROG zg30
TD 65536
SOLVENT MeOD
NS 16
DS 0
SWH 8012.820 Hz
FIDRES 0.122266 Hz
AQ 4.0894966 sec
RG 36.33
DW 62.400 usec
DE 6.50 usec
TE 299.1 K
D1 1.00000000 sec
TD0 1
======== CHANNEL f1 ========
SFO1 400.1522008 MHz
NUC1 1H
P1 10.40 usec
SI 65536
SF 400.1500069 MHz
WDW EM
SSB 0
LB 0.30 Hz
GB 0
PC 1.00

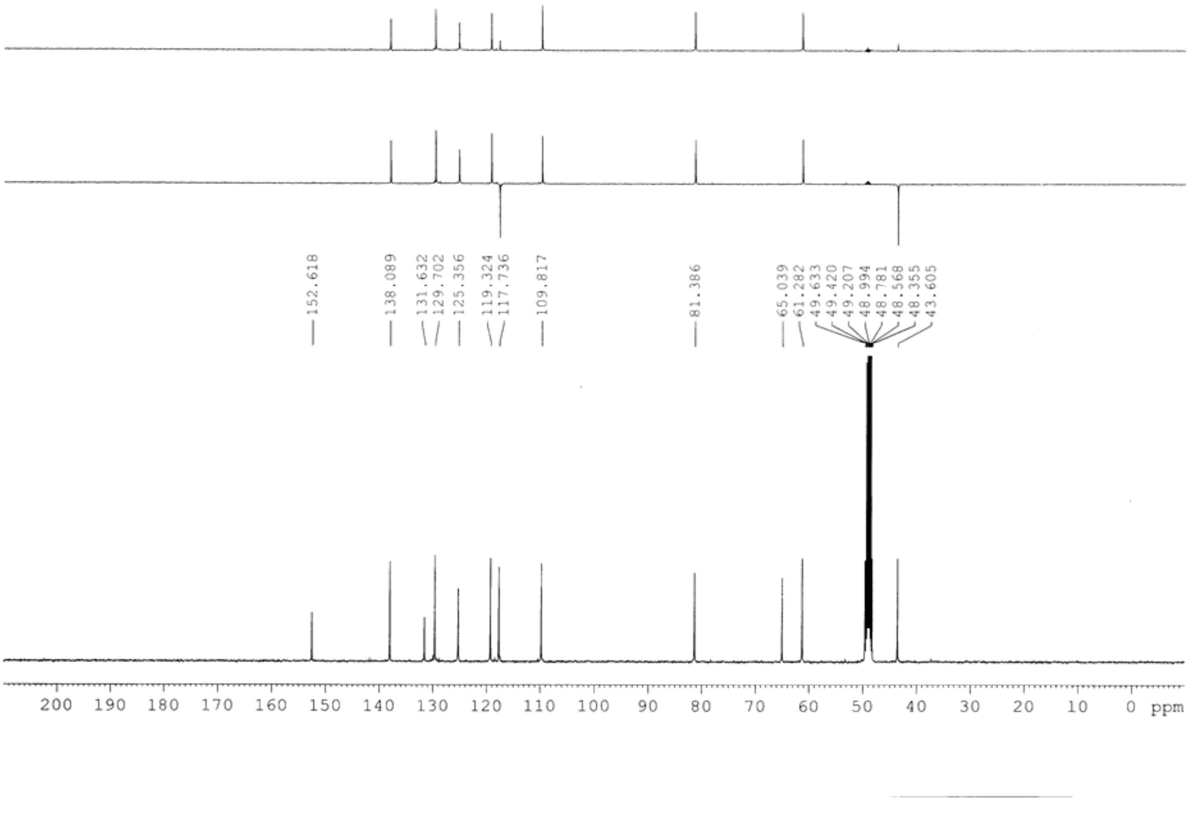
152.618
138.089
131.632
129.702
125.356
119.324
117.736
109.817
81.386
65.039
61.282
49.633
49.420
49.207
48.994
48.781
48.568
48.355
43.605
200
190
180
170
160
150
140
130
120
110
100
90
80
70
60
50
40
30
20
10
0
ppm

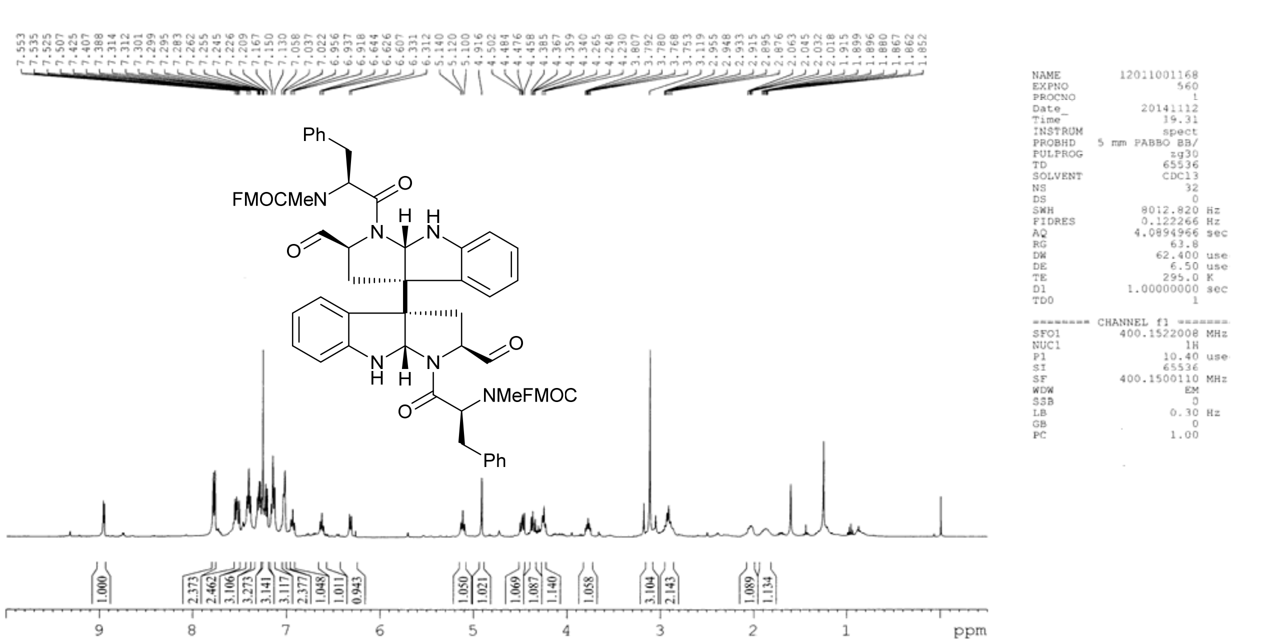
FMOCMeN
NMeFMOC
Ph
NAME 12011001168
EXPNO 560
PROCNO 1
Date_ 20141112
Time 19.31
INSTRUM spect
PROBHD 5 mm PABBO BB/
PULPROG zg30
TD 65536
SOLVENT CDCl3
NS 32
DS 0
SWH 8012.820 Hz
FIDRES 0.122266 Hz
AQ 4.0894966 sec
RG 63.8
DW 62.400 use
DE 6.50 use
TE 295.0 K
D1 1.00000000 sec
TD0 1
======== CHANNEL f1 =======
SFO1 400.1522008 MHz
NUC1 1H
P1 10.40 use
SI 65536
SF 400.1500110 MHz
WDW EM
SSB 0
LB 0.30 Hz
GB 0
PC 1.00
ppm

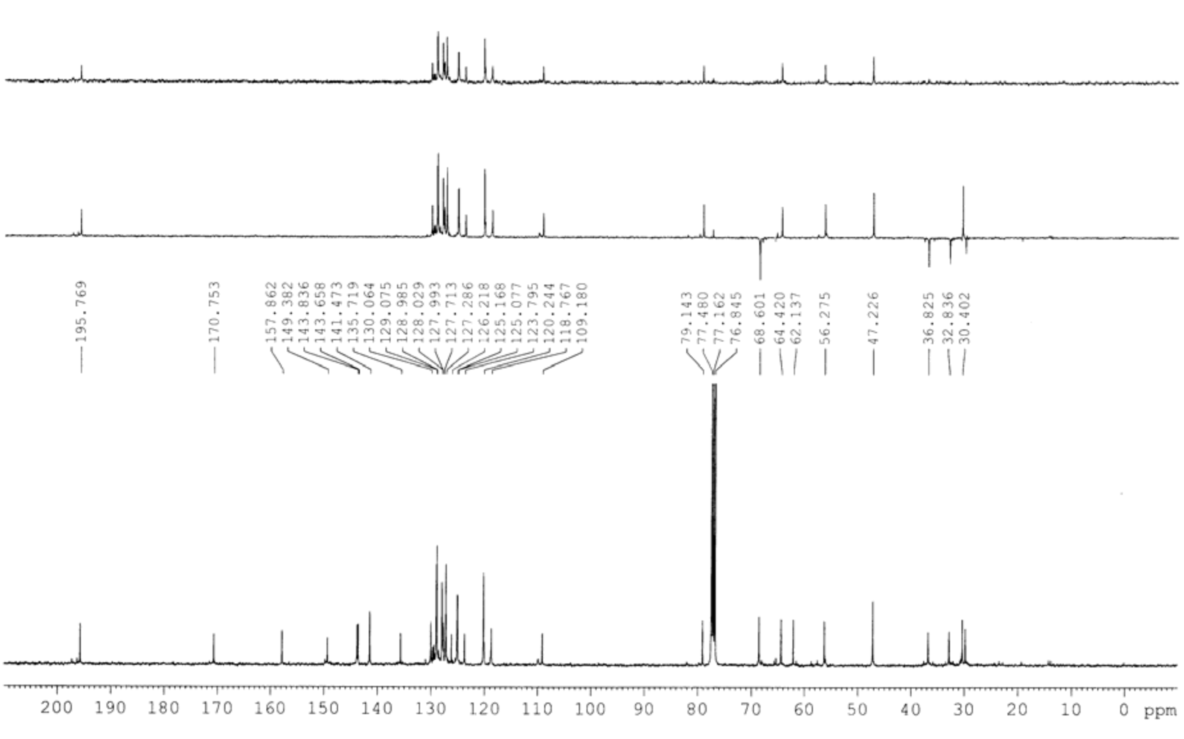
195.769
170.753
157.862
149.382
143.836
143.658
141.473
135.719
130.064
129.075
128.985
128.029
127.993
127.713
127.286
126.218
125.168
125.077
123.795
120.244
118.767
109.180
79.143
77.480
77.162
76.845
68.601
64.420
62.137
56.275
47.226
36.825
32.836
30.402
200 190 180 170 160 150 140 130 120 110 100 90 80 70 60 50 40 30 20 10 0 ppm

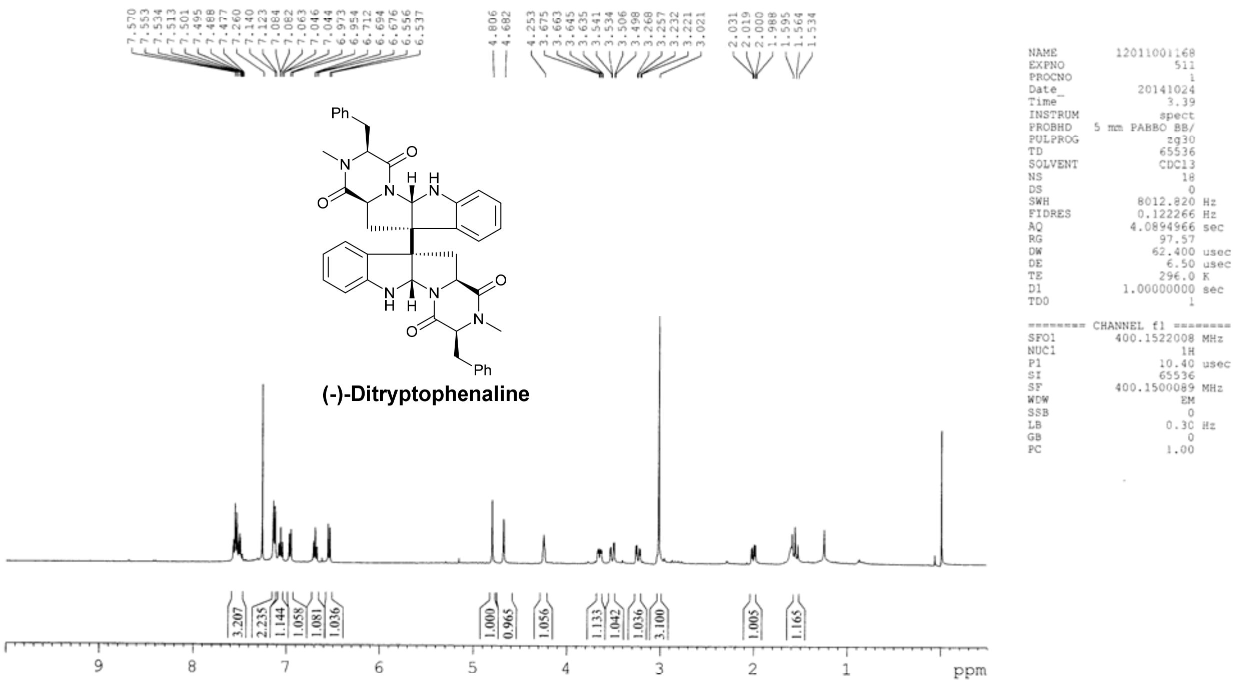
7.570 7.553 7.534 7.513 7.501 7.495 7.488 7.477 7.260 7.140 7.123 7.084 7.082 7.063 7.046 7.044 6.973 6.954 6.712 6.694 6.676 6.556 6.537
4.806 4.682
4.253 3.675 3.663 3.645 3.635 3.541 3.534 3.506 3.498 3.268 3.257 3.232 3.221 3.021
2.031 2.019 2.000 1.988 1.595 1.564 1.534
Ph
O
N
H
O
(-)-Ditryptophenaline
3.207 2.235 1.144 1.058 1.081 1.036 1.000 0.965 1.056 1.133 1.042 1.036 3.100 1.005 1.165
9 8 7 6 5 4 3 2 1 ppm
NAME 12011001168
EXPNO 511
PROCNO 1
Date_ 20141024
Time 3.39
INSTRUM spect
PROBHD 5 mm PABBO BB/
PULPROG zg30
TD 65536
SOLVENT CDCl3
NS 18
DS 0
SWH 8012.820 Hz
FIDRES 0.122266 Hz
AQ 4.0894966 sec
RG 97.57
DW 62.400 usec
DE 6.50 usec
TE 296.0 K
D1 1.00000000 sec
TD0 1
======== CHANNEL f1 ========
SFO1 400.1522008 MHz
NUC1 1H
P1 10.40 usec
SI 65536
SF 400.1500089 MHz
WDW EM
SSB 0
LB 0.30 Hz
GB 0
PC 1.00

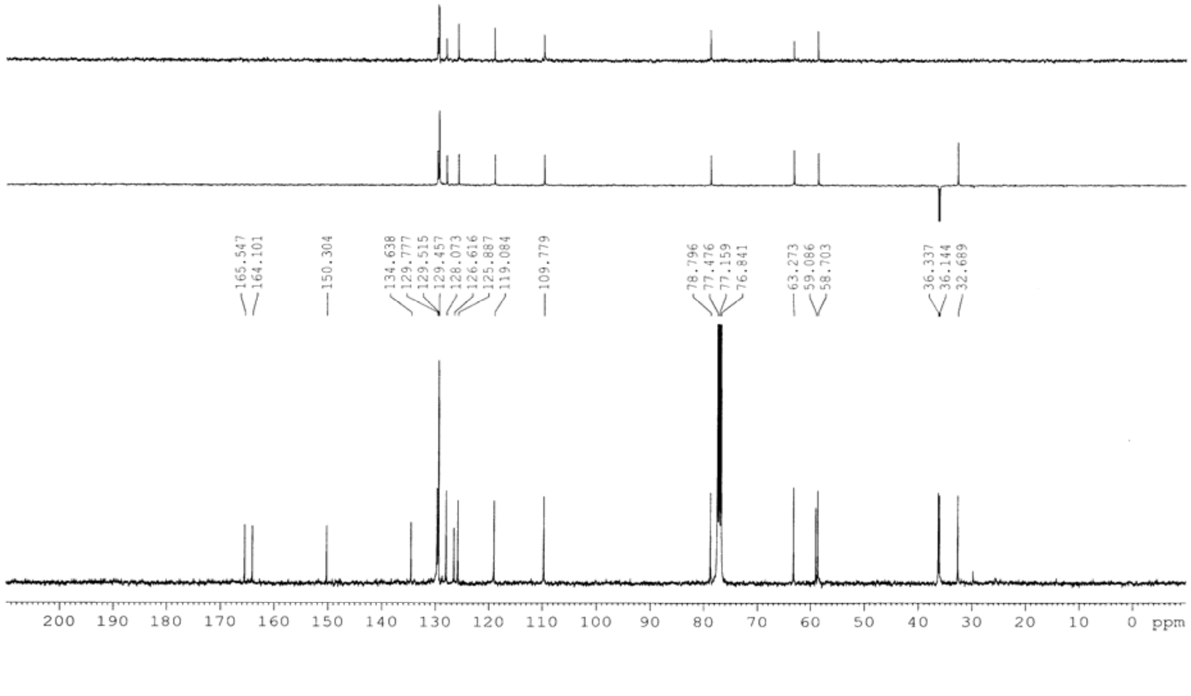
165.547 164.101 150.304 134.638 129.777 129.515 129.457 128.073 126.616 125.887 119.084 109.779 78.796 77.476 77.159 76.841 63.273 59.086 58.703 36.337 36.144 32.689
200 190 180 170 160 150 140 130 120 110 100 90 80 70 60 50 40 30 20 10 0 ppm

Data File C:\HPCHEM\1\VERIFY\DEFAULT.VAL\SXF00305.D Sample Name: zero

```
=====================================================================
Injection Date  : 2013-8-26 18:46:18 下午
Sample Name     : zero                          Location :   -
Acq. Operator   :
Acq. Method     : C:\HPCHEM\1\METHODS\CQ.M
Last changed    : 2013-8-26 18:40:14 下午
                  (modified after loading)
Analysis Method : C:\HPCHEM\1\METHODS\CQ.M
Last changed    : 2013-8-26 20:10:32 下午
                  (modified after loading)
```

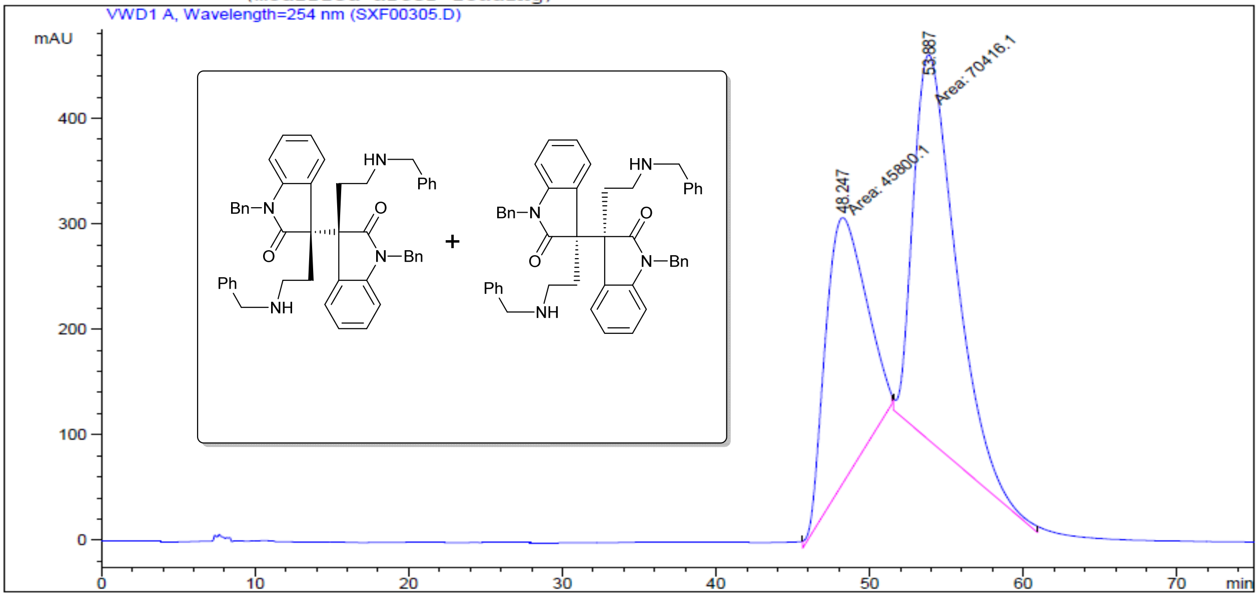


```
=====================================================================
                       Area Percent Report
=====================================================================

Sorted By             :      Signal
Multiplier            :      1.0000
Dilution              :      1.0000

Signal 1: VWD1 A, Wavelength=254 nm
```

Peak #	RetTime [min]	Type	Width [min]	Area mAU *s	Height [mAU]	Area %
1	48.247	MM	3.0300	4.58001e4	251.92842	39.4094
2	53.887	MM	3.2118	7.04161e4	365.40591	60.5906

Totals : 1.16216e5 617.33434

Results obtained with enhanced integrator!

```
=====================================================================
                       *** End of Report ***
```

Data File C:\HPCHEM\1\VERIFY\DEFAULT.VAL\SXF00306.D   Sample Name: zero

```
=====================================================================
Injection Date  : 2013-8-26 20:29:40 下午
Sample Name     : zero                          Location :   -
Acq. Operator   :
Method          : C:\HPCHEM\1\METHODS\CQ.M
Last changed    : 2013-8-26 20:10:32 下午
                  (modified after loading)
```

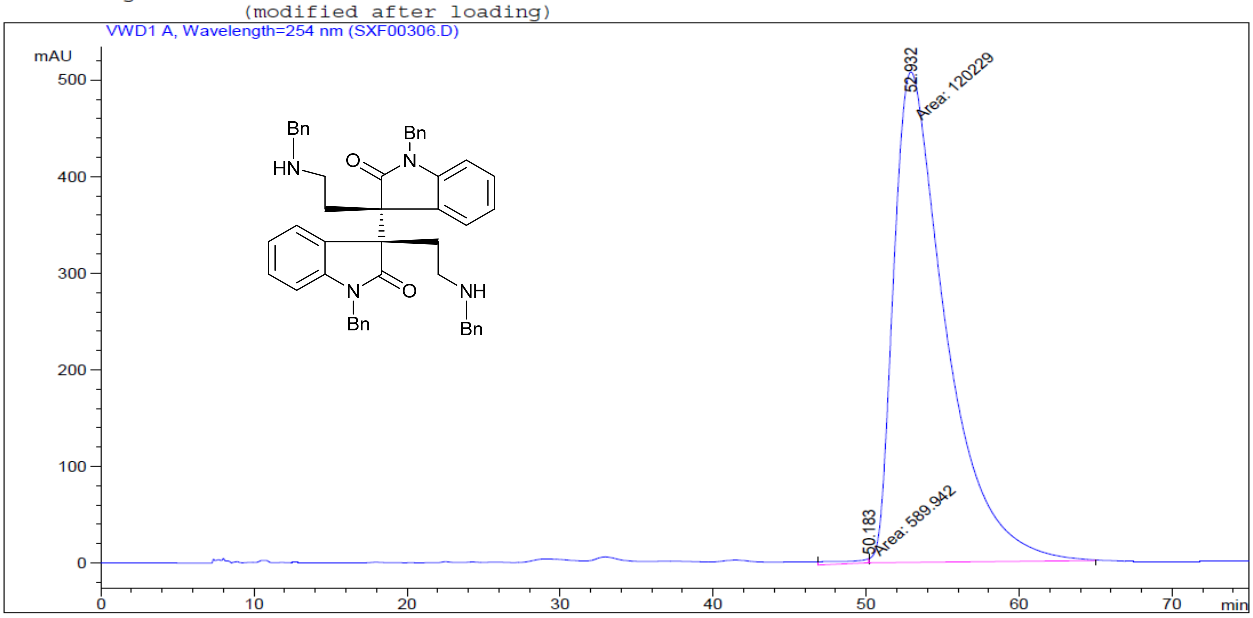


```
=====================================================================
                        Area Percent Report
=====================================================================

Sorted By             :      Signal
Multiplier            :      1.0000
Dilution              :      1.0000

Signal 1: VWD1 A, Wavelength=254 nm
```

Peak #	RetTime [min]	Type	Width [min]	Area mAU *s	Height [mAU]	Area %
1	50.183	MM	2.2697	589.94226	4.33207	0.4883
2	52.932	MM	3.9482	1.20229e5	507.53134	99.5117

Totals : 1.20819e5 511.86342

Results obtained with enhanced integrator!

```
=====================================================================
                        *** End of Report ***
```

===
Injection Date : 2014-4-9 14:57:53 下午
Sample Name : 00011 Location : -
Acq. Operator :
Method : C:\HPCHEM\1\METHODS\ZYY.M
Last changed : 2014-4-9 11:32:11 下午
(modified after loading)

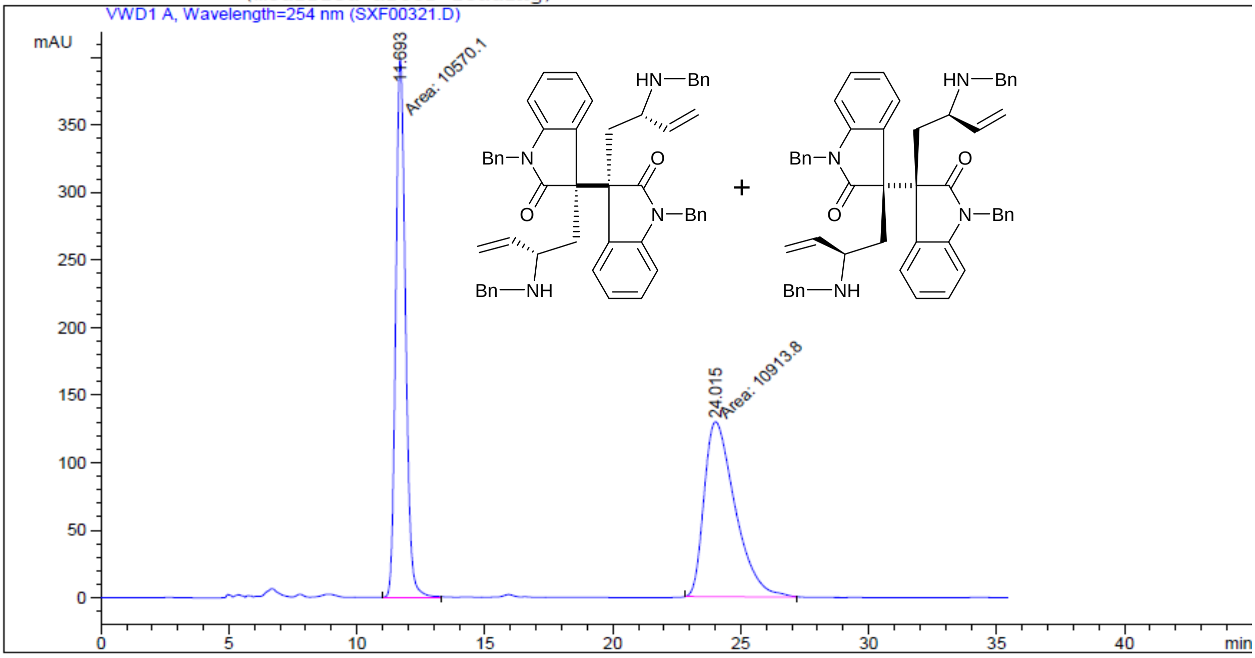

===
Area Percent Report
===

Sorted By : Signal
Multiplier : 1.0000
Dilution : 1.0000

Signal 1: VWD1 A, Wavelength=254 nm

Peak #	RetTime [min]	Type	Width [min]	Area mAU *s	Height [mAU]	Area %
1	11.693	MM	0.4423	1.05701e4	398.32251	49.2003
2	24.015	MM	1.4049	1.09138e4	129.46898	50.7997

Totals : 2.14839e4 527.79149

Results obtained with enhanced integrator!
===
*** End of Report ***

Data File C:\HPCHEM\1\VERIFY\DEFAULT.VAL\SXF00319.D Sample Name: 00011

===

Injection Date : 2014-4-9 13:37:47 下午
Sample Name : 00011 Location : -
Acq. Operator :
Method : C:\HPCHEM\1\METHODS\ZYY.M
Last changed : 2014-4-9 11:32:11 下午
(modified after loading)

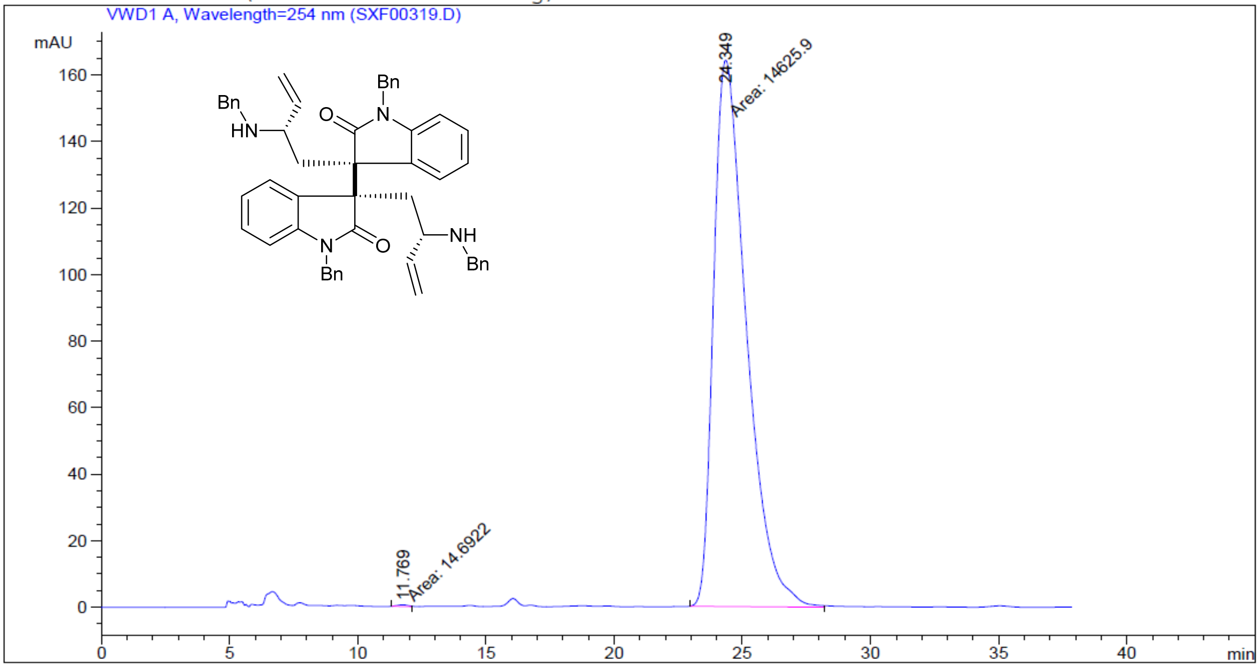

===

Area Percent Report

===

Sorted By : Signal
Multiplier : 1.0000
Dilution : 1.0000

Signal 1: VWD1 A, Wavelength=254 nm

Peak #	RetTime [min]	Type	Width [min]	Area mAU *s	Height [mAU]	Area %
1	11.769	MM	0.5018	14.69220	4.87975e-1	0.1004
2	24.349	MM	1.4844	1.46259e4	164.21399	99.8996
Totals :				1.46406e4	164.70196	

Results obtained with enhanced integrator!

===

*** End of Report ***

Data File C:\HPCHEM\1\VERIFY\DEFAULT.VAL\SXF00414.D Sample Name: 00011

===
Injection Date : 2015-6-3 22:11:24 下午
Sample Name : 00011 Location : -
Acq. Operator :
Method : C:\HPCHEM\1\METHODS\ZJF.M
Last changed : 2015-6-3 20:49:24 下午
(modified after loading)

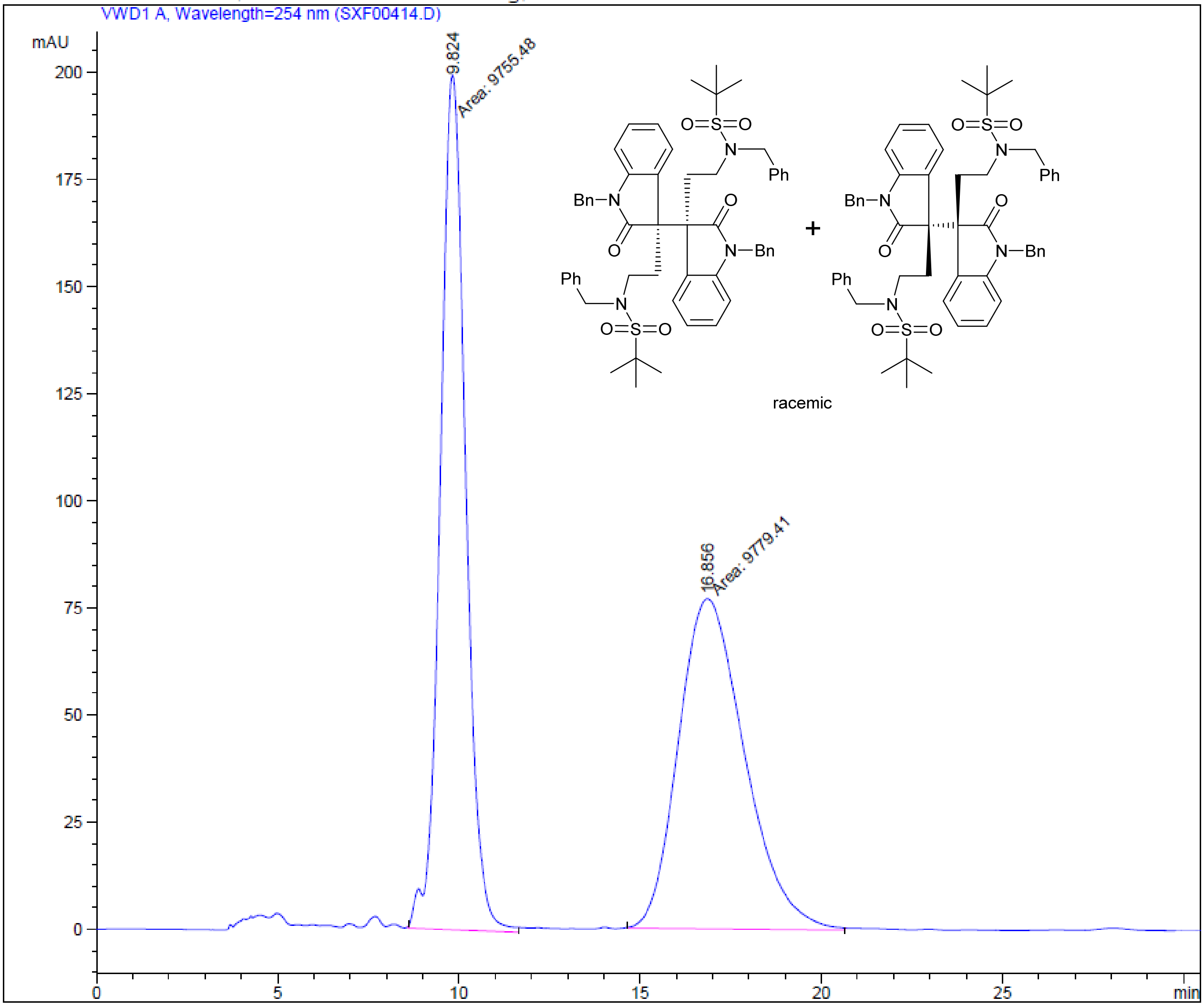

===
Area Percent Report
===

Sorted By : Signal
Multiplier : 1.0000
Dilution : 1.0000

Signal 1: VWD1 A, Wavelength=254 nm

Peak #	RetTime [min]	Type	Width [min]	Area mAU *s	Height [mAU]	Area %
1	9.824	MM	0.8150	9755.48340	199.49382	49.9388
2	16.856	MM	2.1142	9779.41211	77.09438	50.0612

Totals : 1.95349e4 276.58820

Results obtained with enhanced integrator!
===

Instrument 1 2015-6-3 22:41:53 下午 Page 1 of 1

Data File C:\HPCHEM\1\VERIFY\DEFAULT.VAL\SXF00413.D Sample Name: 00011

===

Injection Date : 2015-6-3 21:37:49 下午
Sample Name : 00011 Location : -
Acq. Operator :
Method : C:\HPCHEM\1\METHODS\ZJF.M
Last changed : 2015-6-3 20:49:24 下午
(modified after loading)

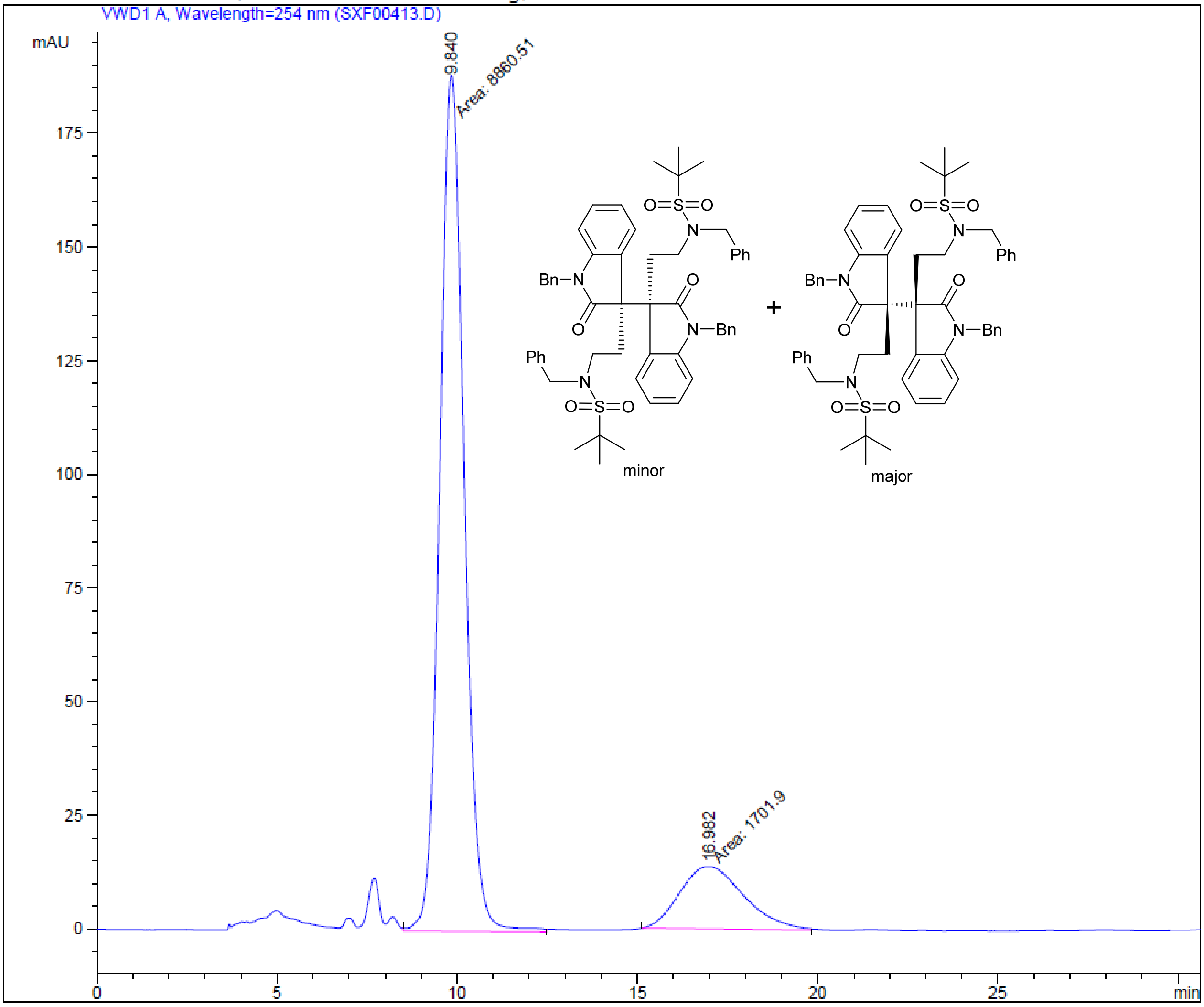

===

Area Percent Report

===

Sorted By : Signal
Multiplier : 1.0000
Dilution : 1.0000

Signal 1: VWD1 A, Wavelength=254 nm

Peak #	RetTime [min]	Type	Width [min]	Area mAU *s	Height [mAU]	Area %
1	9.840	MM	0.7843	8860.50586	188.29802	83.8872
2	16.982	MM	2.0655	1701.90039	13.73279	16.1128

Totals : 1.05624e4 202.03081

Results obtained with enhanced integrator!

===

Data File C:\HPCHEM\1\VERIFY\DEFAULT.VAL\SXF00412.D Sample Name: 00011

===

Injection Date : 2015-6-3 21:04:43 下午
Sample Name : 00011 Location : -
Acq. Operator :
Method : C:\HPCHEM\1\METHODS\ZJF.M
Last changed : 2015-6-3 20:49:24 下午
(modified after loading)

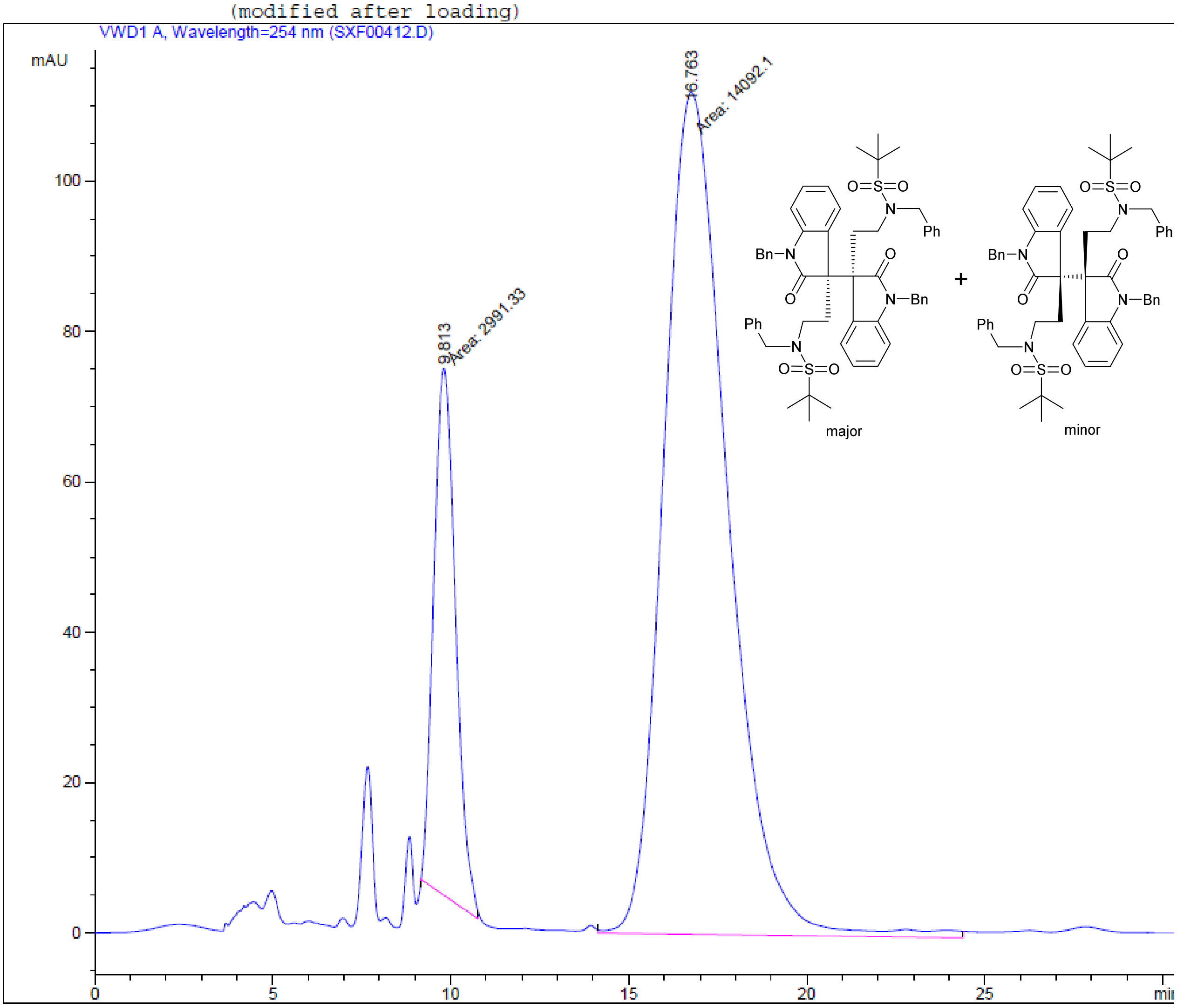

===

Area Percent Report

===

Sorted By : Signal
Multiplier : 1.0000
Dilution : 1.0000

Signal 1: VWD1 A, Wavelength=254 nm

Peak #	RetTime [min]	Type	Width [min]	Area mAU *s	Height [mAU]	Area %
1	9.813	MM	0.7120	2991.33228	70.01850	17.5101
2	16.763	MM	2.0989	1.40921e4	111.90126	82.4899

Totals : 1.70835e4 181.91976

Results obtained with enhanced integrator!

===
